# Glycosylation-related genes mediated prognostic signature contribute to prognostic prediction and treatment options in ovarian cancer: based on bulk and single‑cell RNA sequencing data

**DOI:** 10.1186/s12885-024-11908-4

**Published:** 2024-02-14

**Authors:** Yue You, Qing Yang

**Affiliations:** Department of gynaecology, Shengjing Hospital of China Medical University, Shenyang, China

**Keywords:** Ovarian cancer, Glycosylation, Immunotherapy, scRNA-seq, Immune microenvironment, *CYBRD1*

## Abstract

**Background:**

Ovarian cancer (OC) is a complex disease with significant tumor heterogeneity with the worst prognosis and highest mortality among all gynecological cancers. Glycosylation is a specific post-translational modification that plays an important role in tumor progression, immune escape and metastatic spread. The aim of this work was to identify the major glycosylation-related genes (GRGs) in OC and construct an effective GRGs signature to predict prognosis and immunotherapy.

**Methods:**

AUCell algorithm was used to identify glycosylation-related genes (GRGs) based on the scRNA-seq and bulk RNA-seq data. An effective GRGs signature was conducted using COX and LASSO regression algorithm. The texting dataset and clinical sample data were used to assessed the accuracy of GRGs signature. We evaluated the differences in immune cell infiltration, enrichment of immune checkpoints, immunotherapy response, and gene mutation status among different risk groups. Finally, RT-qPCR, Wound-healing assay, Transwell assay were performed to verify the effect of the *CYBRD1* on OC.

**Results:**

A total of 1187 GRGs were obtained and a GRGs signature including 16 genes was established. The OC patients were divided into high- and low- risk group based on the median riskscore and the patients in high-risk group have poor outcome. We also found that the patients in low-risk group have higher immune cell infiltration, enrichment of immune checkpoints and immunotherapy response. The results of laboratory test showed that *CYBRD1* can promote the invasion, and migration of OC and is closely related to the poor prognosis of OC patients.

**Conclusions:**

Our study established a GRGs signature consisting of 16 genes based on the scRNA-seq and bulk RNA-seq data, which provides a new perspective on the prognosis prediction and treatment strategy for OC.

**Supplementary Information:**

The online version contains supplementary material available at 10.1186/s12885-024-11908-4.

## Background

Ovarian cancer (OC) is a common malignancy of the female reproductive system, ranking third behind cervical and endometrial cancers. In 2020, there will be an estimated 313,959 new cases of ovarian cancer worldwide, with 207,252 deaths [1]. Because the onset is hidden and the diagnosis is often in the advanced stage of the disease, the mortality rate of ovarian cancer is the highest in gynecological tumors. Although there have been great advances in treatment options for ovarian cancer, such as surgery combined with platinum or taxane chemotherapy, recurrence and drug resistance rates remain high, and the 5-year survival rate is only about 40% [2]. Therefore, it is urgent to find new biomarkers or therapeutic targets to improve the survival rate and clinical efficacy for ovarian cancer patients. Recently, researchers have found that the biological behavior of tumors such as proliferation, metabolism, apoptosis are closely related to energy metabolism [3]. Glycosyltransferases (GTs) catalyze the transfer of active monosaccharide donors to carbohydrates to produce a variety of oligosaccharide structures, which play an important role in energy metabolism [4]. Many studies have shown that the abnormal expression of glycosyltransferase is closely related to the malignant degree and prognosis of the tumor [5, 6].

Glycosylation is the process of attaching sugar chains to sugar, lipid or protein molecules under the control of enzymes. Glycosylation represents a unique set of protein modifications that may involve the linking of monosaccharides or even entire oligosaccharides (glycans) to specific amino acids within glycoproteins. Glycosylation occurs primarily in the Golgi apparatus and endoplasmic reticulum and reflects the coordinated action of a complex set of enzymes, organelles, and other factors. The two most common mechanisms by which sugar chains link to lipins and proteins are the glycosylation of O⁃ and N⁃ links [7]. Glycosylation is also one of the epigenetic changes in tumor transformation [8]. Abnormal glycosylation modifications in tumors were first described 50 years ago [9]. So far, many studies have shown that glycosylation modification led by glycosylation genes is closely related to the occurrence and development of tumors [10]. In particular, glycans cannot be synthesized directly from genome-coding templates like proteins and nucleic acids, and their fidelity depends largely on the specificity of glycosyltransferase (GT), which synthesizes complex glycans by adding one sugar at a time. Most eukaryotic GTs locate in the lumen of secretory pathways and modify proteins and lipids during transport to the cell surface or extracellular space [11]. Compared to cells that were not malignantly transformed, tumor cells showed abnormal glycosylation changes, and abnormal glycosylation has been recognized as a universal hallmark of all cancers. Therefore, the in-depth study of glycosylation-related genes (GRGs) may contribute to the prognosis prediction and accurate treatment of tumors.

Single-cell transcriptome sequencing (RNA-seq) allows for quantitative and qualitative analysis of cell composition in complex tissues at the level of individual cells. We can identify commonalities and differences in the cell composition of tumor samples from different sites using the RNA-seq technique [12, 13]. In this study, AUCell algorithm was used to identify GRGs among individual OC cells. Then, a GRGs signature was identified based on the LASSO and COX regression analysis. The patients were divided into high- and low-risk group and the patients in high-risk group have poor prognosis. We also found that the patients in low-risk group have higher immune cell infiltration, enrichment of immune checkpoints and immunotherapy response. Our study provides a new perspective on the prognosis prediction and treatment strategy for OC.

## Materials and methods

### Data processing

We downloaded one mRNAs-seq data (GSE63885) and one single-cell RNA-sequencing dataset (GSE184880) from the Gene Expression Omnibus (GEO, https://www.ncbi.nlm.nih.gov/). Hereby, 75 OC samples were obtained in GSE63885, 12 samples in GSE184880 (5 healthy controls and 7 OC patients). GSE63885 was used as texting dataset. The Robust Multichip Average (RMA) was used to background adjust and quantile-normalize, then log2-transformed. The training dataset (TCGA-OV including RNA sequencing data, somatic mutation data, Copy Number Variation (CNV) data and clinical data) containing 379 patients, was downloaded from the Genomic Data Commons Data Portal (https://portal.gdc.cancer.gov/). Subsequently, two immunotherapy cohorts including GSE78220 (GEO, https://www.ncbi.nlm.nih.gov/) and IMvigor210 (BLCA, http://research-pub.Gene.com/imvigor210corebiologies/) were obtained. The Robust Multichip Average (RMA) was used to background adjust and quantile-normalize, then log2-transformed. The characteristics of the four datasets were presented in Supplementary Table 1.

### Tissue sample collection and cell lines culture

One hundred and fifty OC patients were obtained from Tissue specimen Bank of Shengjing Hospital between 2012 and 2022. All patients were diagnosed by histopathological examination. Inclusion criteria: (1) Meet EOC diagnostic criteria; (2) did not receive any antitumor therapy before admission; (3) Receiving surgical treatment; (4) clinicopathological and follow-up data were complete; (5) Age > 18. Exclusion criteria: (1) Patients with endometriosis or polycystic ovary syndrome; (2) Patients with malignant tumors of other sites; (3) Patients with severe infectious diseases or autoimmune diseases; (4) Pregnant or lactating women. This study was approved by the Ethics Committee of Shengjing Hospital of the China Medical University, and informed consent was obtained from all patients. In addition, all methods were executed in accordance with relevant guidelines and regulations (2023PS175K). Human OC cell lines HO-8910, OVCAR-3 were obtained from Bina Nusantara Computer Club (Beijing, China). The cells were cultured in RPMI-1640 medium with 10% fetal bovine serum at 37 °C and 5% CO2 in an incubator.

### Single-cell sequencing data and glycosylation-related genes acquired and processing

The low-quality cells were filtered for subsequent analysis using R (version 4.1.3) and Seurat (version 4.1.1). Cells with the following conditions are considered low-quality cells: (1) the number of genes is less than 200 or more than 5000; (2) The proportion of mitochondrial gene expression was greater than 20%. We normalized the data using Harmony for the batch de-effect, and then we used the “FindVariableFeatures” function to detect the first 3000 highly variable genes. Principal component analysis (PCA) was then applied to reduce the dimensionality of the scRNA-seq data based on 3000 variable genes. The “RunTSNE” function was applied to perform the t-distributed stochastic neighbor embedding (t-SNE) to analyze the scRNA-seq data. Cell cluster analysis uses the “FindClusters” function (with the “resolution” parameter set to 0.5) and “FindAllMarkers” to identify differentially expressed genes between each group of cells. To identify the marker genes in each group of cells, critical thresholds were used, adjusted for *p* < 0.05,|Log2FC|≥0.5. Eventually, different cell types were annotated based on the marker genes [14, 15]. 185 glycosyltransferase pathway genes were obtained according to previous study [16].

### AUCell

The “AUCell” R package was used to analyze the activity status of gene sets in the scRNA-seq data and was used to assign a GT activity score to each cell line. Based on the area under the curve (AUC) value of the selected GRGs, the gene expression ranking of each cell was used to estimate the percentage of highly expressed gene sets in each cell. The cells were divided into high-GT-AUC and low-GT-AUC groups according to the median AUC value and visualized using the “ggplot2” R software [15]. Single sample gene set enrichment analysis (ssGSEA) was employed to assign GT scores for each TCGA-OV patient. Differential expression analysis was used to screen differentially expressed genes (DEGs) in high and low GRGs_ AUC group for further investigation. Furthermore, we used correlation analysis to look at the genes most connected with GT activity, with the top 150 most associated genes being included for future study. The DEGs and genes discovered through association analysis were the ones that had the greatest effect on GT activity. Next, we applied Gene ontology (GO) and disease (DO) enrichment analysis using ‘clusterProfiler’ package in R software to explore the potential mechanisms of these GRGs [15, 17].

### Construction and validation of a GRGs signature

Univariate analysis was conducted to select the GRGs with prognostic value. Next, the LASSO regression analysis and forward stepwise regression were performed to establish a scoring system to quantify all individuals with OC using the following formula:

$\text{R}\text{i}\text{s}\text{k}\, \text{S}\text{c}\text{o}\text{r}\text{e} = \sum \left(\text{C}\text{o}\text{e}\text{f}\text{i} * \text{E}\text{x}\text{p}\text{r}\text{i}\right)$; here, *i* means the genes, *Coefi* means the coefficient of each gene and *Expri* means the expression level of each gene.

The patients were divided into the high- and low-risk groups after zero-mean normalization. The robustness of the risk scoring system was verified using GSE57495 dataset and clinical sample dataset.

### GSVA Enrichment analysis

Gene set variation analysis (GSVA) enrichment analysis was performed to explore the differential biological mechanism between different risk group using “GSVA” R package. The gene set c2.cp.kegg.v7.4.symbols. gmt and h.all.v2022.1.Hs.symbols. gmt were obtained from the MSigDB database (https://www.gsea-msigdb.org/gsea/msigdb). False discovery rate (FDR) was corrected by Benjamini and Hochberg (BH) method and FDR < 0.05 was regarded as significant difference [18].

### Tumor immunity and immunotherapy

In our study, CIBERSORT algorithm was performed to compare the different levels of tumor immune cell infiltration in the high- and low- risk groups and visualized using the heatmap or histogram. “ESTIMATE” package in R software was used to estimate the ratio of immune matrix components in Immune microenvironment (TME), which was presented in the form of three scores: ImmuneScore, StromalScore and ESTIMATEScore were positively correlated with immunity, matrix and the sum of the two, respectively, which means that the higher the corresponding score, the greater the proportion of corresponding components in TME. In addition, immune checkpoints were composed of various molecules expressed on immune cells that regulate the level of immune activation. Therefore, we compared the expression levels of immune checkpoints in high- and low- risk groups. The potential reactivity to immune checkpoint inhibitors (ICIs) was evaluated using the online Tumor Immune Dysfunction and Exclusion (TIDE) algorithm (http://tide.dfci.harvard.edu/), and the patients with lower TIDE scores are more sensitive to immunotherapy [19]. In addition, Submap was used to calculate correlations between high- and low-risk groups, as well as patients who responded/ non-responded to ICIs, to infer the efficacy of immunotherapy [20].

### Development of potential therapeutic agents

we obtained drug sensitivity data for human cancer cell lines (CCLs) from the Cancer Therapeutics Response Portal (CTRP, https://portals.broadinstitute.org/ctrp) and PRISM Repurposing dataset (https://depmap.org/portal/prism/). The expression data of CCLs were downloaded from the Cancer Cell Line Encyclopedia (CCLE, https://portals.broadinstitute.org/ccle/) database. The CTRP and PRISM datasets have areas under dose-response curves, and a lower AUC value indicates a higher sensitivity to this treatment [21]. In addition, we used the “prorophic” R package to assess the IC50 of four chemotherapy agents (cisplatin, gefitinib, gemcitabine, and paclitaxel) in the high- and low- risk groups. The correlations between GRGs expression and some chemotherapeutic drugs were calculated using Pearson correlation analysis and visualized by ggplot2 [22].

### Quantitative real-time RT-PCR

Total RNA form One hundred and fifty OC patients were obtained from Tissue specimen Bank of Shengjing Hospital was extracted by TRIzol (Invitrogen, USA). Then, the purity and concentration of the RNA samples were measured. Subsequently, the sample RNA was first generated into cDNA by reverse transcription and Real time quantitative polymerase chain reaction (RT-qPCR) was performed. 2-ΔΔCt method was used to calculate the relative expression levels of genes, with GAPDH used as an internal reference. The primer sequences of the genes were presented in Supplementary Table 2.

### Wound-healing assay

A cell suspension containing 10^6 cells was added to the six-well plate to ensure that the cell growth density could reach more than 95% the next day. Mark with a 200 µL gun head and line the bottom of the board according to the scratch. The scratch healing was observed after 48 h culture in the incubator, and 2% serum 1640 was used in the medium.

### Transwell assay

At the stage of cell to logarithmic growth, the cells were starved for 12 h using low-serum medium. Between 20,000 and 50,000 cells are added to each chamber. In a 24-well plate, the upper chamber and lower chamber were cultured using serum-free medium and 1640 medium with 30% serum, respectively. After the top layer cells were removed by PBS, the remaining cells at the bottom layer were fixed by adding 4% paraformaldehyde, and then the crystal violet dye was added for 15 min staining treatment.

### Statistical analysis

The “maftools”R package was used to plot the “oncoplot” based on the somatic mutation data. Correlation analysis was applied using Pearson or Spearman coefficients. T-test or the Mann-Whitney U test was performed to the comparison among groups. Chi-square and Fisher’s exact tests were used to ensure exact test. Kaplan-Meier (K-M) survival curves was plotted to assess prognostic value and the statistical significance was evaluated by log-rank test. All statistical data analyses were conducted using R software (version 4.1.2). The level of significance was set at P < 0.05, and all statistical tests were two-sided.

## Results

### Single-cell RNA sequencing in OC

**Fig. 1:**
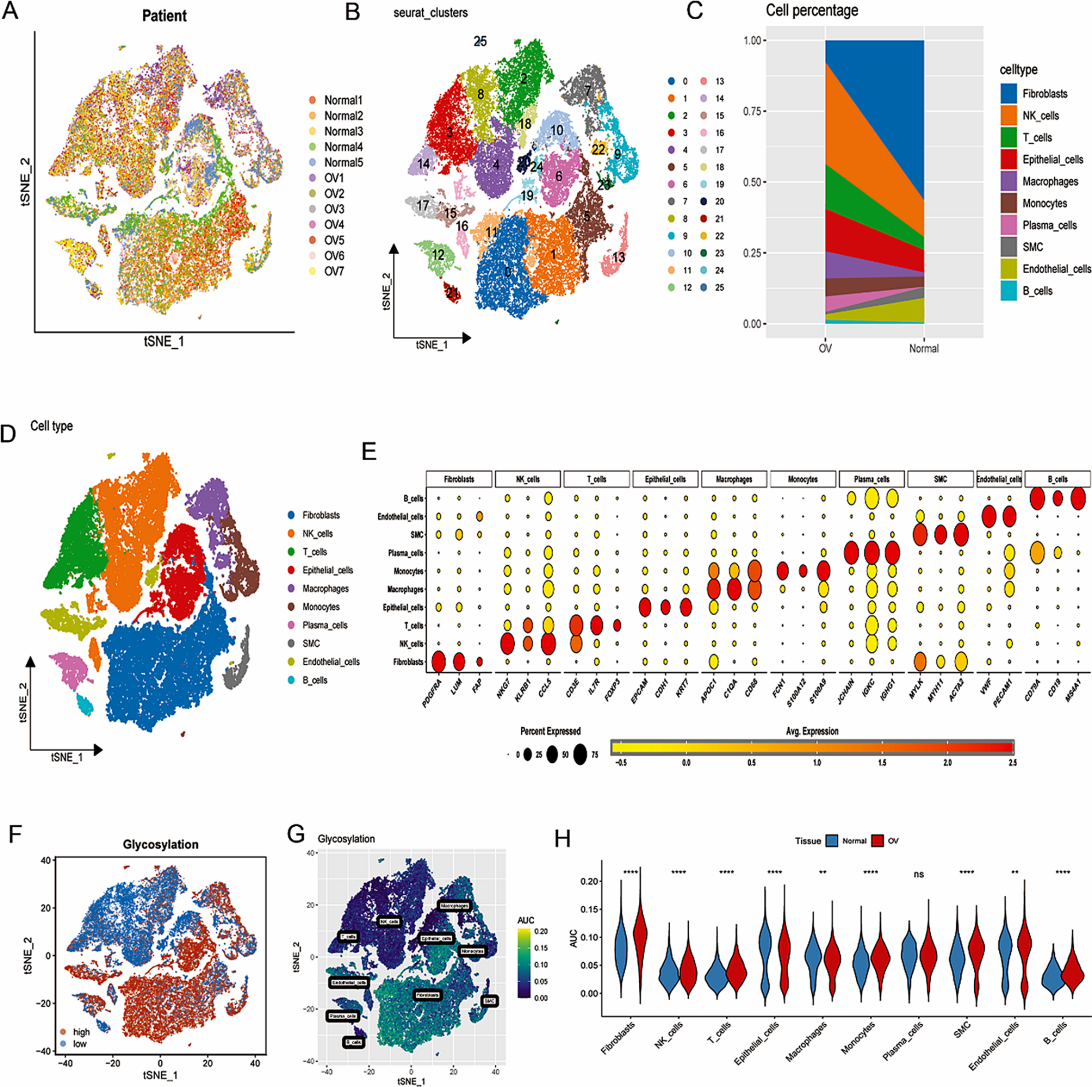
Annotation of single-cell data and GT activity. (**A**) The tSNE plots of cells from 7 OC samples and 5 normal samples. (**B**) The t-SNE plots of 25 clusters from 7 OC samples and 5 normal samples. (**C**) Different proportions of cells in 7 OC samples and 5 normal samples. (**D**) The t-SNE map indicates that 12 samples can be annotated as 10 cell types. (**E**) Bubble diagram plots of marker genes of ten major cell type. (**F**, **G**) All cells were scored according to glycosylation-associated genes (GRGs) and were classified into high- and low- AUC groups. (**H**) The difference in GT activity levels in each cell type between normal and OC samples. *p < 0.05; **p < 0.01; ***p < 0.001. Abbreviation: ns: not significant

**Fig. 2:**
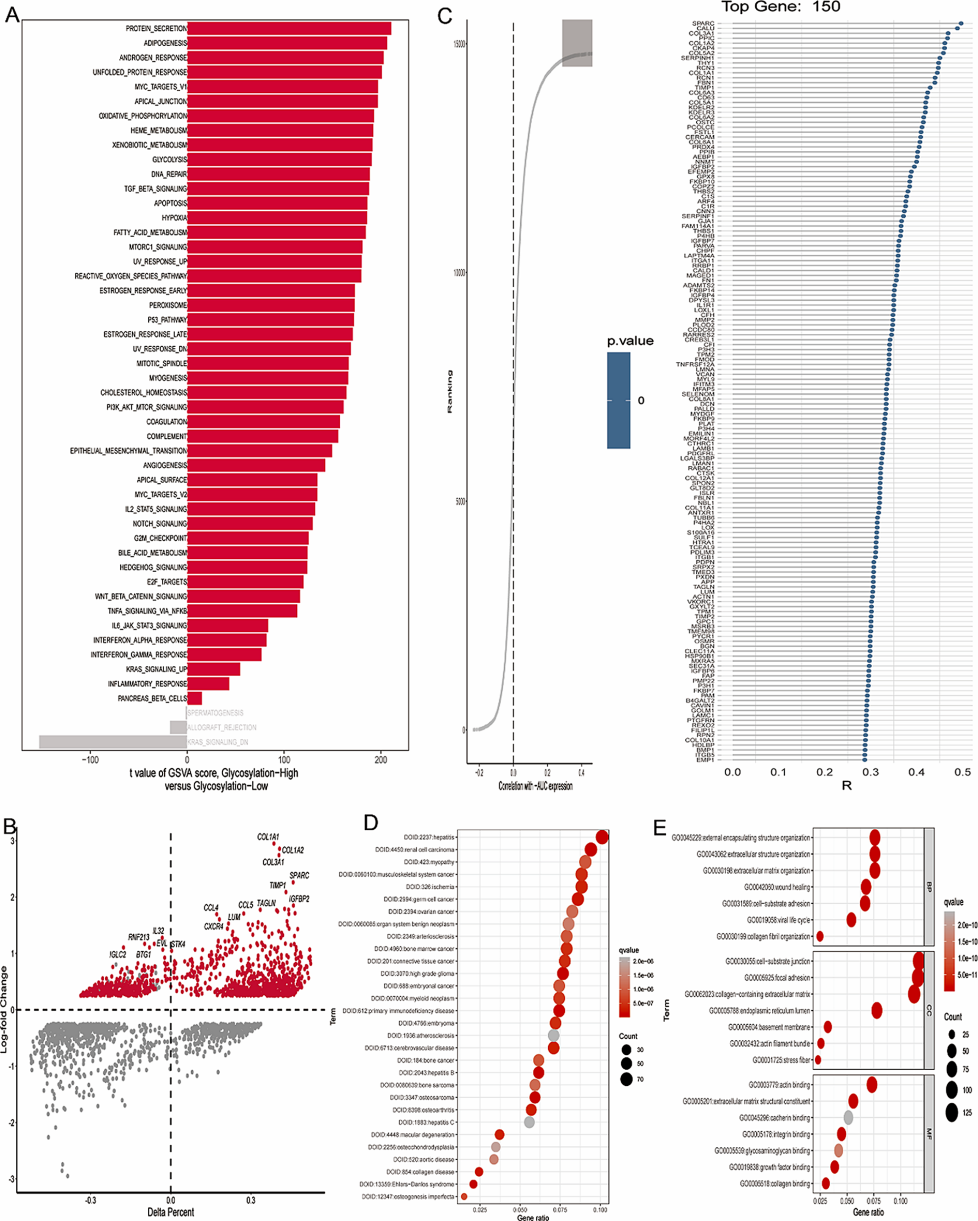
Identification of GTs gene list in OC. (**A**) GSVA indicated the enrichment of hallmark gene sets in different GT AUCell groups. (**B**) Differential expression analysis in high and low GRGs_ AUC group. (**C**) Correlation analysis to select genes have significant correlation with GT-AUCell score. (**D**–**E**) GO and DO functional enrichment analysis of GTs gene

Firstly, we performed quality control on the single cell sequencing data. Some cells were eliminated and the percentage of ribosomal genes, mitochondrial genes, and red blood cell genes were limited to confirm the validity of the cell sample (Supplementary Fig. 1A). There was a significant positive correlation between sequencing depth and total intracellular sequence (*R* = 0.85, Supplementary Fig. 1B). The study included a total of 12 samples (5 normal samples and 7 OC samples). The distribution of cells from different sample sources was uniform, indicating that the batch effect was well corrected (Fig. 1A). Then, the single cell sequencing data was divided into 25 cell clusters using the “FindClusters” function (Fig. 1B). After quality control, dimensionality reduction, integration and clustering of the data, the cells were annotated according to the marker genes of the cells, which could be divided into 10 cell types (Fig. 1D). The bubble map shows the marker genes of different cell types, confirming the accuracy of cell type annotation (Fig. 1E). Figure 1C shows the difference in the proportion of these 10 cell types in OC tissues and normal tissues. Among them, NK cells, T cells, epithelial cells, macrophages, monocytes, plasma cells, occupied a higher proportion in OC tissues compared with normal tissues, while the proportion of B cells, SMC, endothelial cells and fibroblasts were the opposite. The GT activity of each cell was measured using the “AUCell” R package, with higher AUC values indicating higher GT activity (Fig. 1F). Finally, we calculated a corresponding GRGs AUC score for each cell and divided them into high GRGs _AUC group and low GRGs_ AUC group according to the median AUC score (Fig. 1G). Figure 1H demonstrated significant disparities in GT activity levels in in each cell type between normal and OC samples. We performed GSVA analysis to explore the underlying biological mechanisms between high and low GRGs_ AUC group. The results indicated that the high GRGs _AUC group mainly enriched in PROTEIN SECRETION, ADIPOGENESIS, ANDROGEN RESPONSE and UNFOLDED PROTEIN RESPONSE (Fig. 2A, Supplementary Table 3). Next, differential expression analysis screened 1169 DEGs in high and low GRGs_ AUC group (Fig. 2B). Correlation analysis was applied to select the genes associated with GRGs activity and the top 150 genes were screened (Fig. 2C). We integrated these 1169 DEGs with these 150 genes for further study and 1187 genes were obtained. GO and DO functional enrichment analyses were used to explore the potential functions of these genes. DO and GO indicated that these genes were not only associated with glycosylation process but also with ovarian cancer progression (Fig. 2D–E, Supplementary Tables 4 and 5).

### Construction and validation of a GRGs signature

**Fig. 3:**
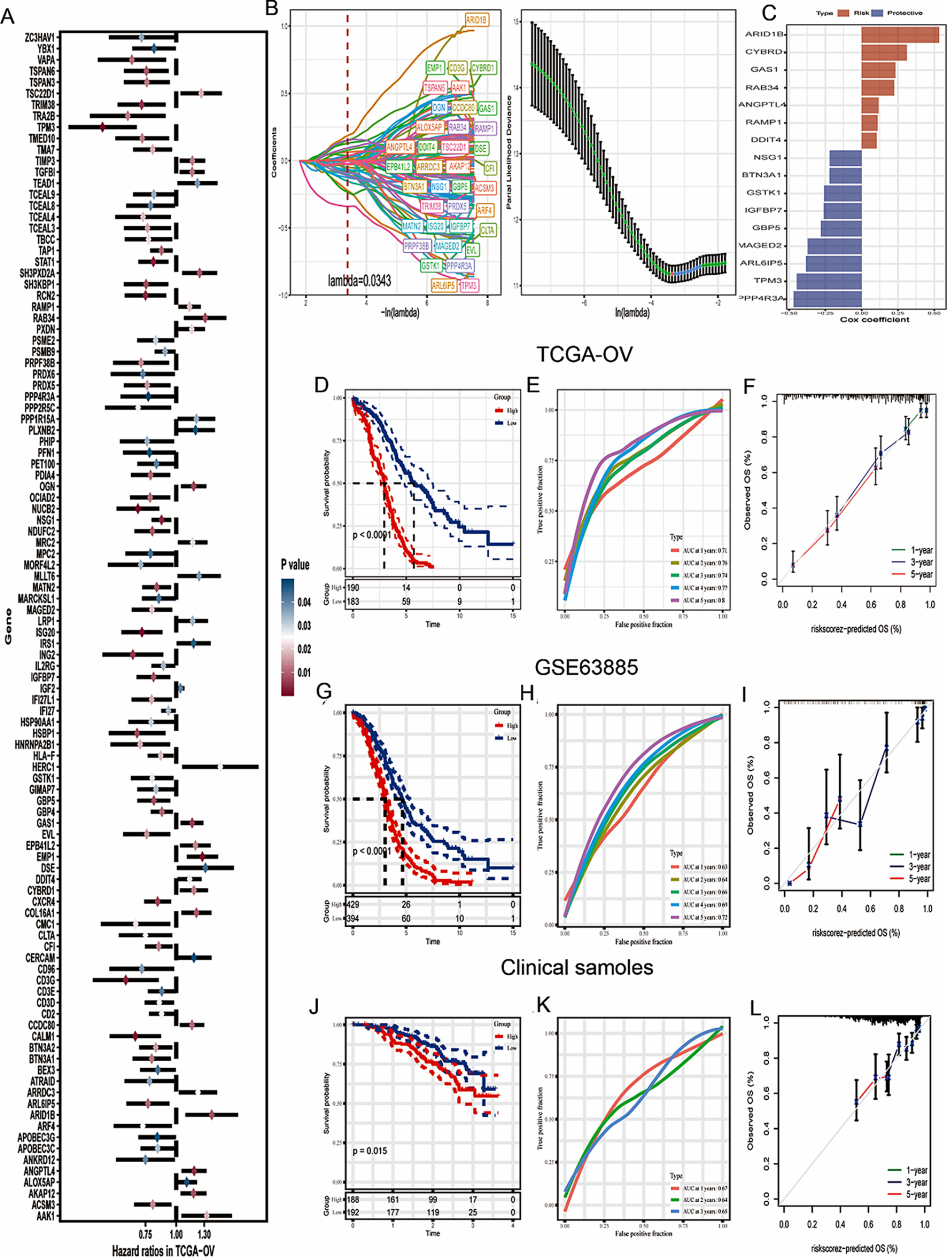
Construction and validation of a GRGs signature. (**A**) Univariate analysis selected genes with significant prognostic value from the GRGs. (**B**) LASSO Cox regression analysis was performed to select genes to conduct a GRGs signature. (**C**) Coefficients of 16 genes in GRGs signature. (**D**) Kaplan-Meier prognostic analysis in TCGA dataset. (**E**) The AUC values at 1, 2, 3, 4, and 5 years for TCGA dataset. (**F**) Calibration curves of 1-, 3-, 5-year in TCGA dataset. (**G**) Kaplan-Meier prognostic analysis in GSE63885 dataset. (**H**) The AUC values at 1, 2, 3, 4, and 5 years for GSE63885 dataset. (**I**) Calibration curves of 1-, 3-, 5-year in GSE63885 dataset. (**J**) Kaplan-Meier prognostic analysis in clinical samples. (**K**) The AUC values at 1, 2, 3, 4, and 5 years for clinical samples. (**L**) Calibration curves of 1-, 3-, 5-year in clinical samples

Univariate analysis selected the 106 genes with significant prognostic value from the 1187 GRGs (*P* < 0.01) (Fig. 3A). Lasso Cox regression analysis was performed to narrow down the number of genes, with 38 genes left as lambda = 0.0343 (Fig. 3B). Finally, we included 16 genes in the risk signature after multivariate Cox regression analysis with stepwise regression method forward stepwise regression (Fig. 3C). Each OC patient obtained a riskscore based on the following formula:

risk score = 0.261 × *expri IGFBP7* − 0.281 ×*expri GBP5* − 0.468 × *expri PPP4R3A* + 0.534 × *expri ARID1B* − 0.258 × *expri GSTK1* − 0.382 × *expri ARL6IP5* + 0.103 × *expri DDIT4* − 0.222 × *expri BTN3A1* − *0.447* × *expri TPM3* − 0.371 × *expri MAGED2* + 0.118 × *expri ANGPTL4* − 0.220 × *expri NSG1* + 0.225 × *expri RAB34* + 0.233 × *expri GAS1* + 0.312 × *expri CYBRD1* + 0.111 × *expri RAMP1* (Supplementary Table 6).

The OC patients in TCGA-OV were classified into high- and low- risk groups. The patients in high-risk group have poor outcome (Fig. 3D). ROC curve was plotted and the areas under the 1, 2, 3, 4 and 5-year were 0.71, 0.76, 0.74, 0.77 and 0.8 respectively (Fig. 3E). Calibration curve at 1-, 3- and 5-year indicated that the predicted OS probability of the model was close to the actual (Fig. 3F). Then, the stability and accuracy of the GRGs signature were successfully verified in GSE63885 and clinical sample data (One hundred and fifty OC patients obtained from Tissue specimen Bank of Shengjing Hospital) (Fig. 3D, G–L). In clinical sample data, we first obtained the expression levels of 16 genes using RT-PCR, and then obtained the riskscore using the same methods mentioned in training dataset. The GSVA algorithm was used to investigate the potential mechanism of poor prognosis in high-risk group. KEGG pathways related to cancer development and progression, such as GLYCOLYSIS GLUCONEOGENESIS, MISMATCH REPAIR, HOMOLOGOUS RECOMBINATION, MTOR SIGNALING PATHWAY, WNT SIGNALING PATHWAY were enriched in high- risk group (Supplementary Fig. 2A). Hallmark pathways such as HALLMARK_TNFA_SIGNALING_VIA_NFKB, HALLMARK_HYPOXIA, HALLMARK_IL6_JAK_STAT3_SIGNALING, HALLMARK_APOPTOSIS and HALLMARK_COMPLEMENT have a strong positive correlation with riskscore (Supplementary Fig. 2B).

### Mutation landscape analysis

**Fig. 4:**
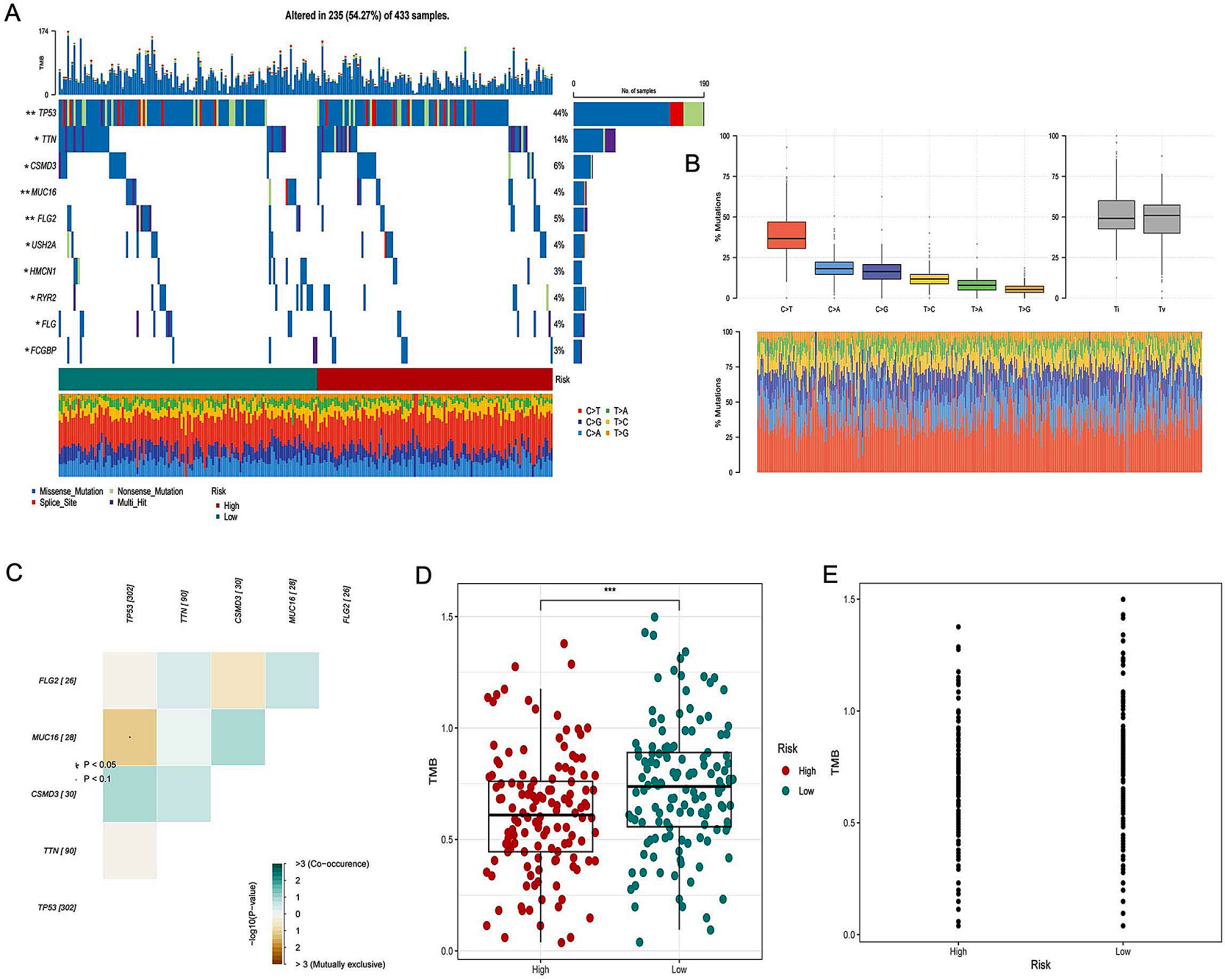
Landscape of OC sample mutation profiles. (**A**) The top 10 mutated genes in TCGA-OV samples. (**B**) Transition and transversion distribution of SNVs in TCGA-OV samples. (**C**) Mutation symbiosis among the top5 mutated genes in TCGA-OV samples. (**D**, **E**) The difference of TMB between high- and low- risk groups. *p < 0.05;**p < 0.05<***p < 0.001

A large number of somatic mutations produce neoantigens, activate CD8 + cytotoxic T cells, and exert T-cell-mediated anti-tumor effects. Therefore, understanding the genetic mutation status of tumors is beneficial to the immunotherapy of patients [23]. First, oncoplot was plotted to show the mutation status of the 16 genes in the GRGs signature based on the TCGA-OV dataset. We found that the mutation frequency of the 16 genes was very low (Supplementary Fig. 3A). Supplementary Fig. 3B indicated that there was no significant mutation symbiosis between these 16 genes and the TOP10 mutant genes in ovarian cancer. Supplementary Fig. 3C displayed mutation frequency of ten common carcinogenic pathways in OC. The copy number variation (CNV) of the 16 genes in TCGA-OV dataset was shown in Supplementary Fig. 3D. Supplementary Fig. 3E indicated that the 16 genes have a strong correlation with aneuploidy score. Moreover, we compared the differential gene mutations in the high- and low- risk groups and showed the top10 differential gene mutations in the oncoplot (Fig. 4A). C > T was the main type of mutation detected in TCGA-OV dataset (Fig. 4B). Figure 4C indicated that there is no mutation symbiosis among the top5 differentially mutated genes (TP53, TTN, CSMD3, MUC16 and FLG2). Tumor mutation burden (TMB) is an important biological marker reflecting the degree of tumor mutation. When the TMB of a tumor sample is high, the mutations that produce immunogenic neoantigens in the mutations also increase. It is easier for the immune system to recognize and remove tumor cells [23]. Figure 4D and E displayed that the patients in the low-risk group had a higher TMB than that in high-risk group, indicating that the patients in low-risk group may be more sensitive to immunotherapy.

### Immune-related characteristics and immunotherapy response assessment

**Fig. 5:**
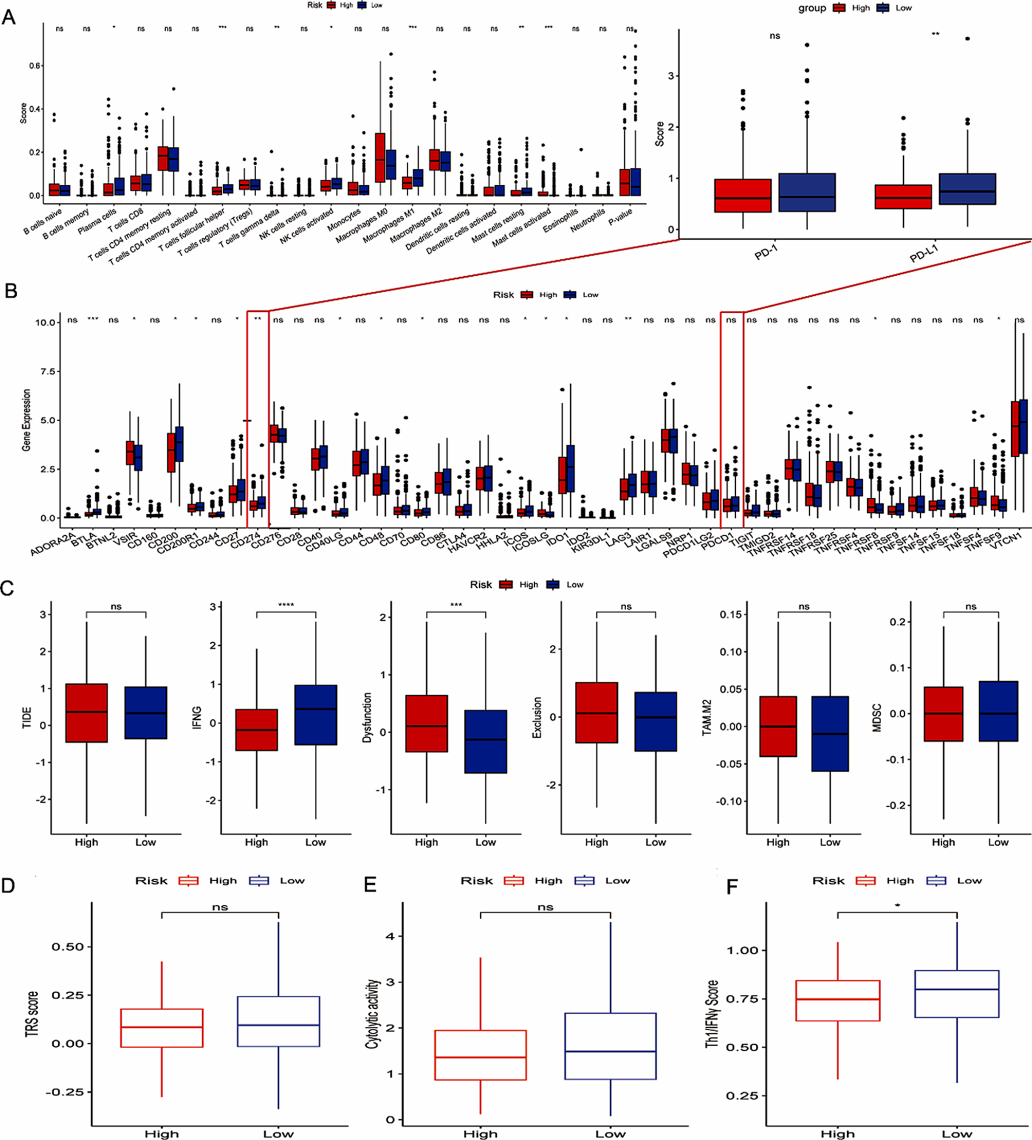
Immune-related characteristics between high- and low- risk groups. (**A**) The differential abundance of 22 types of TIICs between high- and low- risk groups. (**B**) The differential expression level of immune checkpoints between high- and low- risk groups. (**C**) The difference of TIDE score between high- and low- risk groups. (**D**–**F**) The difference of TPS score, cytolytic activity and Th1/IFNy score between high- and low- risk groups. *p < 0.05;**p < 0.05<***p < 0.001

CIBERSORT method was performed to calculate the abundance of tumor-infiltrating immune cells (TIICs) in the TCGA-OV dataset. We found that the infiltration levels of plasma cells, T cells follicular helper, T cells gamma delta, NK cells activated, Macrophages M1, Mast cells resting and Mast cells activated were higher in low-risk group (Fig. 5A). We also found that the majority of immune checkpoints, such as BTLA, CD200, CD200R1, CD27, CD274, CD40LG, CD48, CD80, ICOS, IDO1, LAG3 were higher expressed in low-risk group, while VSIR, ICOSLG, TNFRSF8 and TNFSF9 were strongly expressed in high-risk group (Fig. 5B). The patients in low-risk group have lower TIDE score, suggesting that they may be more benefit from immunotherapy (Fig. 5C). In addition, we also found that TRS score, cytolytic activity and Th1/IFNy score related to inflammatory response were higher in low-risk group (Fig. 5D–F). Correlation analysis showed that the 16 genes in the GRGs signature were strongly correlated with ImmuneScore, StromalScore, ESTIMATEScore, and TIICs (Supplementary Figs. 4, 5).

**Fig. 6:**
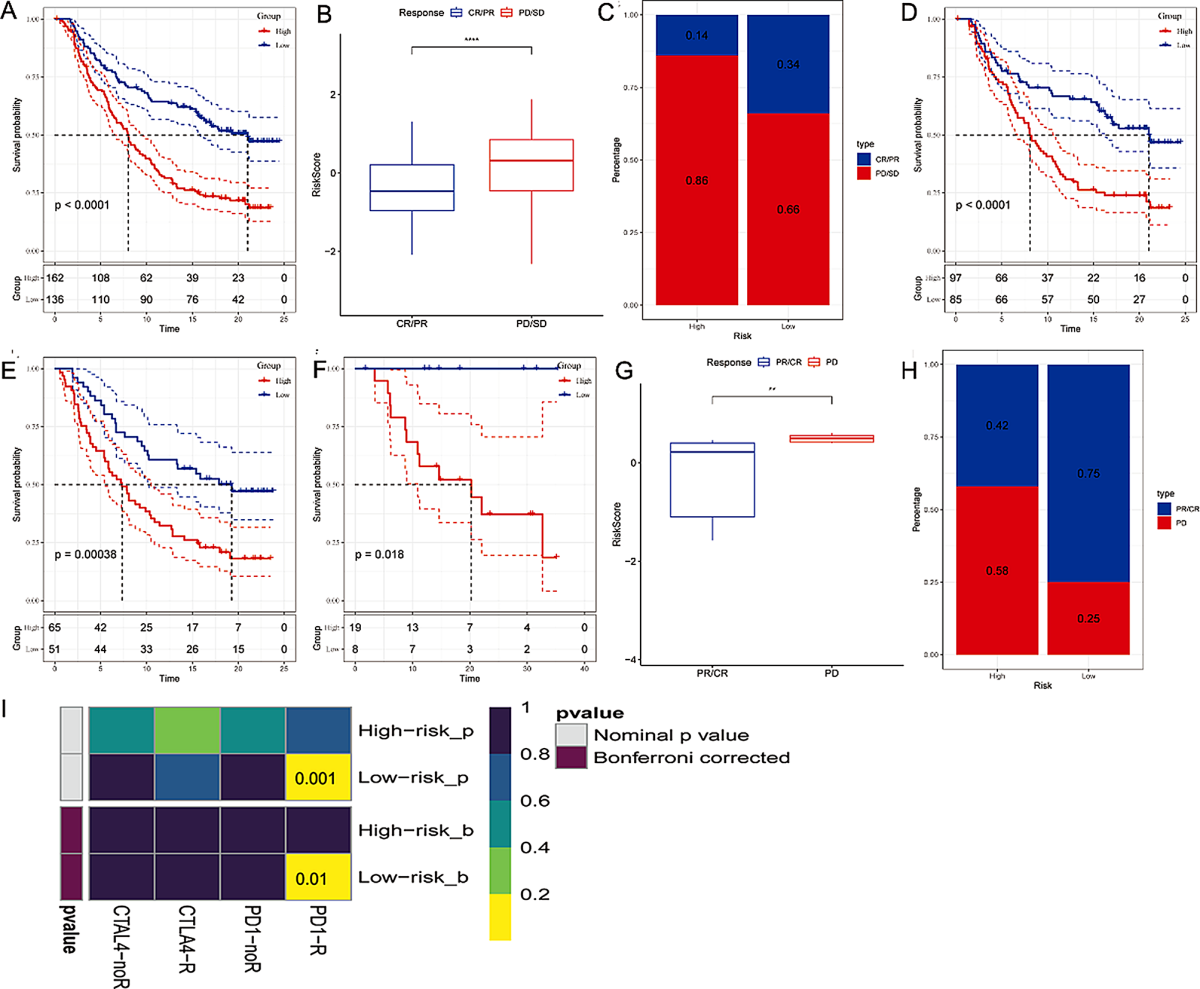
Immunotherapy response assessment. (**A**) Kaplan-Meier prognostic analysis in the IMvigor210 cohort. (**B**) The difference of riskscore between different immunotherapy responses in the IMvigor210 cohort. (**C**) The differential distribution of immunotherapy responses between high- and low- riskscore groups in the IMvigor210 cohort. (**D**) Kaplan-Meier prognostic analysis in the IMvigor210 cohort. (**E**) Kaplan-Meier prognostic analysis in stage I/II patients in the IMvigor210 cohort. (**F**) Kaplan-Meier prognostic analysis in stage III/IV patients in the GSE78220 dataset. (**G**) The difference of riskscore between different immunotherapy responses in the GSE78220 dataset. (**H**) The differential distribution of immunotherapy responses between high- and low- riskscore groups in the GSE78220 dataset. (**I**) Submap analysis indicated the association between the GRGs signature and immunotherapy responses. *p < 0.05;**p < 0.05<***p < 0.001

Considering the ability of the GRGs riskscore to predict immunotherapy sensitivity in OC patients, we validated its predictive stability in immunotherapy datasets: GSE78220 and IMvigor210. In IMvigor210 dataset, UC patients with low- GRGs riskscore have better survival outcomes and more sensitivity to anti-PD-L1 immunotherapy (Fig. 6A–C). In addition, the significant survival differences were not affected by tumor stage (Fig. 6D, E). In the GSE78220 dataset, melanoma patients with low- GRGs riskscore have longer overall survival and higher responsiveness to anti-PD-1 immunotherapy (Fig. 6F–H). Subgraph analysis also showed that patients with low- GRGs riskscore presented better anti-PD-1 immunotherapy responses (Fig. 6I).

### Development of potential therapeutic agents

**Fig. 7:**
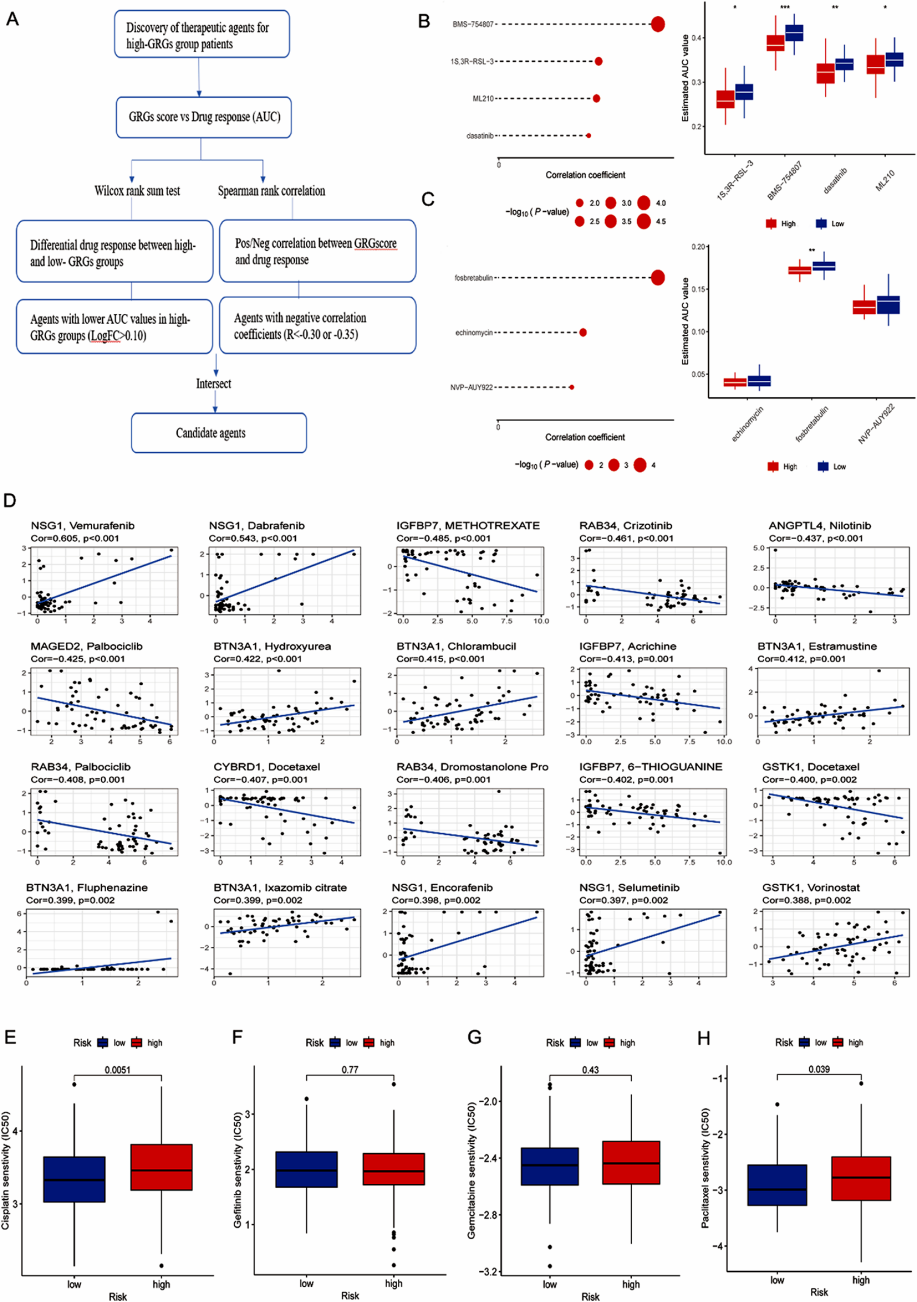
Development of potential therapeutic agents. (**A**) Schematic diagram of identifying agents with higher sensitivity for patients in high-risk group. (**B**) Four CTRP-derived drugs were selected. (**C**) Three PRISM-derived drugs were selected. (**D**) Correlation between 16 GRGs and chemotherapeutic drugs (**E**–**H**) The differential sensitivity of cisplatin, gemcitabine, paclitaxel and gefitinib in high- and low- risk groups

The actual treatment modalities for OC were surgery combined with platinum or taxane chemotherapy. But many people develop resistance to the treatment. Therefore, we predict that the potentially sensitive drugs for both high-risk and low-risk groups are beneficial to the accurate treatment of ovarian cancer patients and improve the long-term survival rate of patients. In our study, two different approaches, CTRP and PRISM, were used to screen potential drugs for high-risk groups of patients (Fig. 7A). We found that four CTRP-derived drugs (including BMS-754,807, 1 S,3R-RSL-3, ML210 and dasatinib) and three PRISM-derived drugs (including fosbretabulin, echinomycin and NVP-AUY922) were selected (Fig. 7B and C). Figure 7D indicated that the 16 GRGs have a strong correlation with some chemotherapy drugs. Additionally, we compared the semi-maximum inhibitory concentrations (IC 50) of four chemotherapy agents in the high- and low- risk groups. We found that the patients in low-risk group have higher sensitivity to cisplatin (*p* = 0.0051, Fig. 7E), gemcitabine (*p* = 0.43, Fig. 7G), and paclitaxel (*p* = 0.039, Fig. 7H), while patients in high- risk group have higher sensitivity to gefitinib (*p* = 0.77, Fig. 7F).

### Validation of the impact of *CYBRD1* in OC

**Fig. 8:**
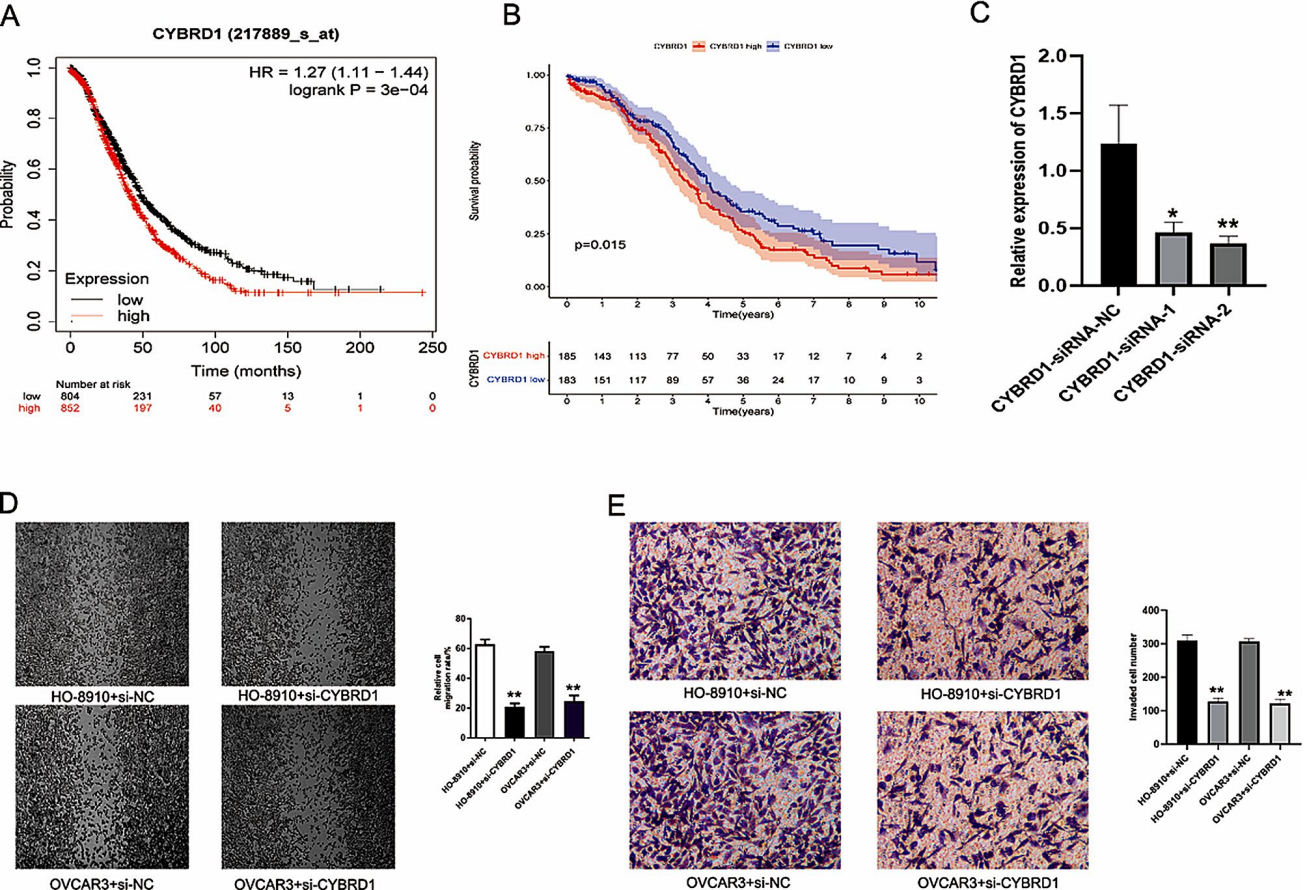
Validation of the impact of CYBRD1 in OC. (**A**, **B**) K–M analysis indicated that patients with low CYBRD1 expression have a longer survival time. (**C**) qRT-PCR to test the interference effect of CYBRD1-siRNAs. (**D**) Wound healing assay. (**E**) Transwell assay. *p < 0.05; **p < 0.05; ***p < 0.001

Among the 16 GRGs, CYBRD1 showed relatively significant carcinogenic capacity. Kaplan–Meier plotter analysis indicated that OC patients with low CYBRD1 expression had longer survival time (Fig. 8A), and we verified this result in 150 clinical samples (Fig. 8B). High expression of CYBRD1 was associated with pathological stage, distant metastasis, lymph node metastasis and other pathological data of ovarian cancer (Supplementary Table 7). Two CYBRD1-silencing lentiviruses were constructed by encapsulation of CYBRD1-specific shrnas, and then transfected into HO-8910 cells to verify the knockdown effect of CYBRD1 at the mRNA level by PCR (Fig. 8C). Wound healing assay and transwell assay indicated that CYBRD1 promotes the migration and invasion capacity of OC cells (Fig. 8D and E). These results of laboratory test showed that CYBRD1 can promote the invasion, and migration of OC and is closely related to the poor prognosis of OC patients.

## Discussion

In recent years, several studies and clinical trials have shown that immunotherapy has made progress in prolonging remission rates and reducing recurrence of OC. Immune processes have important effects on tumor invasion, metastasis, drug resistance and recurrence, and ultimately affect clinical efficacy. Glycosylation is a common post-translational modification of proteins, which transfers sugars to proteins through glycosyltransferase and forms glycosidic bonds with amino acid residues on proteins. Protein glycosylation modification can help immune cells to carry out correct localization and migration [24]. Abnormal glycosylation modification is closely related to tumor genesis, proliferation, invasion, metastasis and immune escape [25, 26].

With the proliferation of medical data and the rapid development of technology and artificial intelligence, the use of big data analysis to build survival prediction models has become an important research topic. Machine learning, a subfield of artificial intelligence, can identify patterns and relationships in the data and provide accurate predictions of future events [27, 28]. Machine learning methods have been used to construct prognostic models for various malignancies, such as lung, liver, breast, and gastrointestinal cancers [29–32]. In the present research, the transcriptome map of OC single cell was constructed and 10 different cell subtypes were obtained based on the data of scRNA-seq. The GT activity of each cell was measured using the “AUCell” R package, 1187 GRGs were obtained using differential expression analysis and correlation analysis. LASSO regression analysis can carry out variable selection and regularization at the same time to improve the prediction accuracy and interpretability of the model [33]. In our study, Lasso regression analysis and multivariate COX were performed to establish a GRGs signature, which was consist of 16 GRGs: IGFBP7, GBP5, PPP4R3A, ARID1B, GSTK1, ARL6IP5, DDIT4, BTN3A1, TPM3, MAGED2, ANGPTL4, NSG1, RAB34, GAS1, CYBRD1 and RAMP1. Finally, the stability and accuracy of the GRGs signature were successfully verified in GSE57495 and clinical sample data. Finally, RT-qPCR, Wound-healing assay, Transwell assay indicated that CYBRD1 can promote the invasion, and migration of OC and is closely related to the poor prognosis of OC patients.

Tumor mutation burden (TMB) is defined as the total number of somatic gene coding errors, base substitution, gene insertion or deletion errors detected per million bases [34]. TMB is associated with the emergence of neoantigens that trigger anti-tumor immunity and can be used to select patients who benefit from immune checkpoint inhibitor (ICB) therapy [35–37]. High TMB indicates that more neoantigens are produced, and T cells released by immune checkpoint inhibitors are more likely to recognize neoantigens, thus achieving the role of attacking and killing tumors. In our study, we found that the patients in the low-risk group have higher TMB, indicating that these patients may be more sensitive to ICB therapy. The GSVA algorithm was used to investigate the potential mechanism in different risk groups. GLYCOLYSIS GLUCONEOGENESIS, MISMATCH REPAIR, HOMOLOGOUS RECOMBINATION, MTOR SIGNALING PATHWAY, WNT SIGNALING PATHWAY considerably enriched in high-risk group, which explains the poor prognosis in the high-risk group. Tumor immune microenvironment (TME) is a complex and dynamic ecosystem with tumor cells, immune cells and supporting cells. Under the influence of chemokines of tumor cells, fibroblasts, or inflammatory cells, immune cells in the bloodstream migrate to the tumor site through a transendothelial process [38, 39]. CIBERSORT method was performed to calculate the abundance of tumor-infiltrating immune cells (TIICs) in the TCGA-OV dataset. We found that the low-risk group had higher infiltration levels of TIICs. The TIDE algorithm is considered to be one of the ways to predict ICB response in cancer. The higher the TIDE score, the worse the ICB response [19]. In this study, TIDE scores in the high-risk group were significantly higher than those in the low-risk group, indicating that the low-risk group had a better ICB response and a better prognosis. In addition, the TRS score, cytolytic activity and Th1/IFNy score related to inflammatory response were higher in low-risk group, which was in line with our expectations. Then, we successfully validated the immunotherapy predictive stability of the GRGs riskscore in immunotherapy datasets: GSE78220 and IMvigor210. Finally, several approaches have successfully selected appropriate chemotherapy agents for patients in high- and low- risk group, providing directions for the precision treatment of OC.

Our research has some advantages. Firstly, we evaluated the predictive value, immunotherapy efficacy, chemotherapy efficacy of the prognostic model for OC patients, providing a basis for the precise treatment of OC patients. Secondly, we incorporated omics information from four dimensions of OC to fully leverage the informative content of each omics dimension. In addition, the current research still has some drawbacks that need to be assessed. Firstly, the limited number of samples involved in scRNA-seq may affect the accuracy and reliability of the results. Secondly, further studies are needed to verify the role of the genes in the development of OC and to fully understand its potential mechanisms and therapeutic value.

## Conclusions

In conclusion, we established and validated a GRGs signature based on the single-cell and bulk RNA-sequencing, which may be used for prognostic prediction and immunotherapy response prediction in OC.

### Electronic supplementary material

Below is the link to the electronic supplementary material.

**Supplementary Figure 1.** Preprocessing of GSE184880 scRNA-seq data. (A) The distribution of gene expression levels, sequencing depth, the percentage of red blood cell genes, the percentage of mitochondrial genes and the percentage of ribosome genes in the 12 samples. (B) Correlation between sequencing depth and gene expression levels, the percentage of mitochondrial genes, the percentage of red blood cell genes, the percentage of ribosome genes.

**Supplementary Figure 2.** GSVA functional enrichment analysis. (A) KEGG pathway gene sets. (B) hallmark gene sets.

**Supplementary Figure 3.** Landscape of 16 GRGs mutation profiles. (A) Mutation landscape of 16 GRGs in TCGA-OV dataset. (B) Mutation symbiosis between these 16 GRGs and the TOP10 mutant genes. (C) The mutation frequency of ten common carcinogenic pathways in OC. (D) The copy number variation (CNV) of the 16 GRGs in TCGA-OV dataset. (E) Correlations between the 16 GRGs and aneuploidy score, homologous recombination defects, fraction altered, number of segments and nonsilent mutation rate.

**Supplementary Figure 4.** The immune infiltrations analysis. (A) Correlations between the 16 GRGs and immune score. (B) Correlations between the 16 GRGs and immune score, stromal score, estimate score. (C) Correlations between 16 GRGs and 22 types of TIICs.

**Supplementary Figure 5.** The immune infiltrations analysis. (A-B) Correlations between 16 GRGs and TIICs. (C) The t-SNE plots indicated the expression of 16 GRGs in 12 samples.

**Supplementary Table 1.** Characteristics of the six datasets.

**Supplementary Table 2.** The primer sequences of 16 GRGs genes.

**Supplementary Table 3.** GSVA analysis to explore the underlying biological mechanisms between high and low GRGs_ AUC group.

**Supplementary Table 4.** GO functional enrichment analyses were used to explore the potential functions of 1187 genes.

**Supplementary Table 5.** DO functional enrichment analyses were used to explore the potential functions of 1187 genes.

**Supplementary Table 6.** 16 genes in the GRGs signature.

**Supplementary Table 7.** Relationship between CYBRD1 expression and tumor characteristics in patients with ovarian cancer. Note?*p<0.05?**p<0.05?***p<0.001

## Data Availability

All data generated or analyzed during this study are included in this published article and its supplementary information files.
