# Supplementary material for: Glycosylation-related genes mediated prognostic signature contribute to prognostic prediction and treatment options in ovarian cancer: based on bulk and single‑cell RNA sequencing data
Source: BMC Cancer. 2024 Feb 14;24:207. doi: 10.1186/s12885-024-11908-4 (PMC10865697; doi:10.1186/s12885-024-11908-4)
Supplement: Supplementary file 1 — Supplementary Figure 1. Preprocessing of GSE184880 scRNA-seq data. (A) The distribution of gene expression levels, sequencing depth, the percentage of red blood cell genes, the percentage of mitochondrial genes and the percentage of ribosome genes in the 12 samples. (B) Correlation between sequencing depth and gene expression levels, the percentage of mitochondrial genes, the percentage of red blood cell genes, the percentage of ribosome genes. [file 12885_2024_11908_MOESM1_ESM.docx]

**
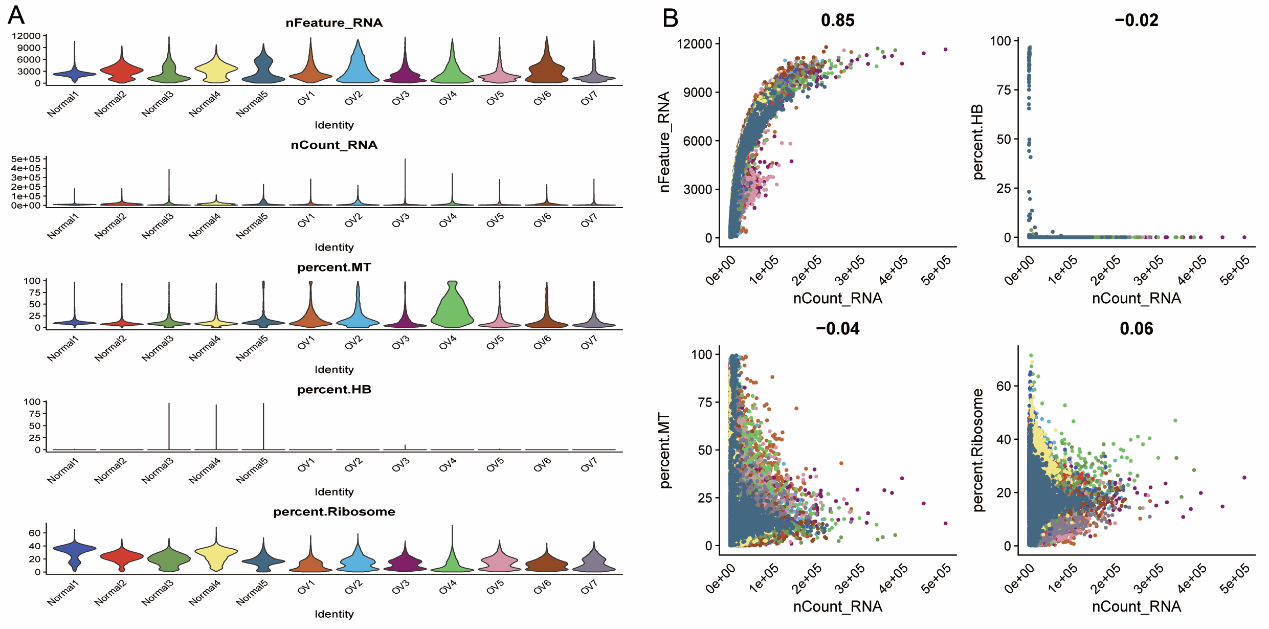
**

Supplementary Figure 1**.** Preprocessing of GSE184880 scRNA-seq data. (A) The distribution of gene expression levels, sequencing depth, the percentage of red blood cell genes, the percentage of mitochondrial genes and the percentage of ribosome genes in the 12 samples. (B) Correlation between sequencing depth and gene expression levels, the percentage of mitochondrial genes, the percentage of red blood cell genes, the percentage of ribosome genes.
