# Supplementary material for: Glycosylation-related genes mediated prognostic signature contribute to prognostic prediction and treatment options in ovarian cancer: based on bulk and single‑cell RNA sequencing data
Source: BMC Cancer. 2024 Feb 14;24:207. doi: 10.1186/s12885-024-11908-4 (PMC10865697; doi:10.1186/s12885-024-11908-4)
Supplement: Supplementary file 2 — Supplementary Figure 2. GSVA functional enrichment analysis. (A) KEGG pathway gene sets. (B) hallmark gene sets. [file 12885_2024_11908_MOESM2_ESM.docx]

**
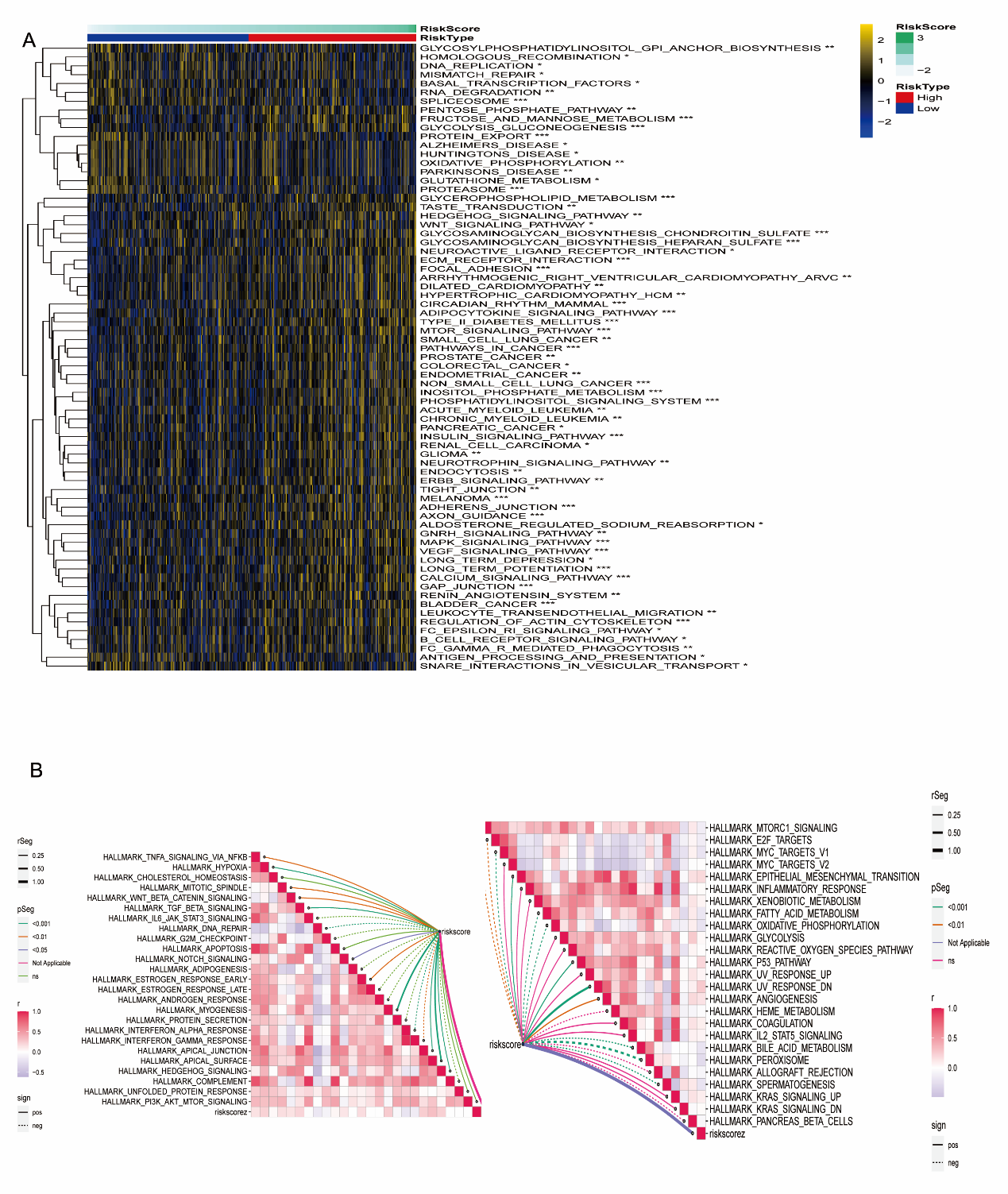
**

Supplementary Figure2**.** GSVA functional enrichment analysis. (A) KEGG pathway gene sets. (B) hallmark gene sets.
