# Supplementary material for: Glycosylation-related genes mediated prognostic signature contribute to prognostic prediction and treatment options in ovarian cancer: based on bulk and single‑cell RNA sequencing data
Source: BMC Cancer. 2024 Feb 14;24:207. doi: 10.1186/s12885-024-11908-4 (PMC10865697; doi:10.1186/s12885-024-11908-4)
Supplement: Supplementary file 3 — Supplementary Figure 3. Landscape of 16 GRGs mutation profiles. (A) Mutation landscape of 16 GRGs in TCGA-OV dataset. (B) Mutation symbiosis between these 16 GRGs and the TOP10 mutant genes. (C) The mutation frequency of ten common carcinogenic pathways in OC. (D) The copy number variation (CNV) of the 16 GRGs in TCGA-OV dataset. (E) Correlations between the 16 GRGs and aneuploidy score, homologous recombination defects, fraction altered, number of segments and nonsilent mutation rate. [file 12885_2024_11908_MOESM3_ESM.docx]

**
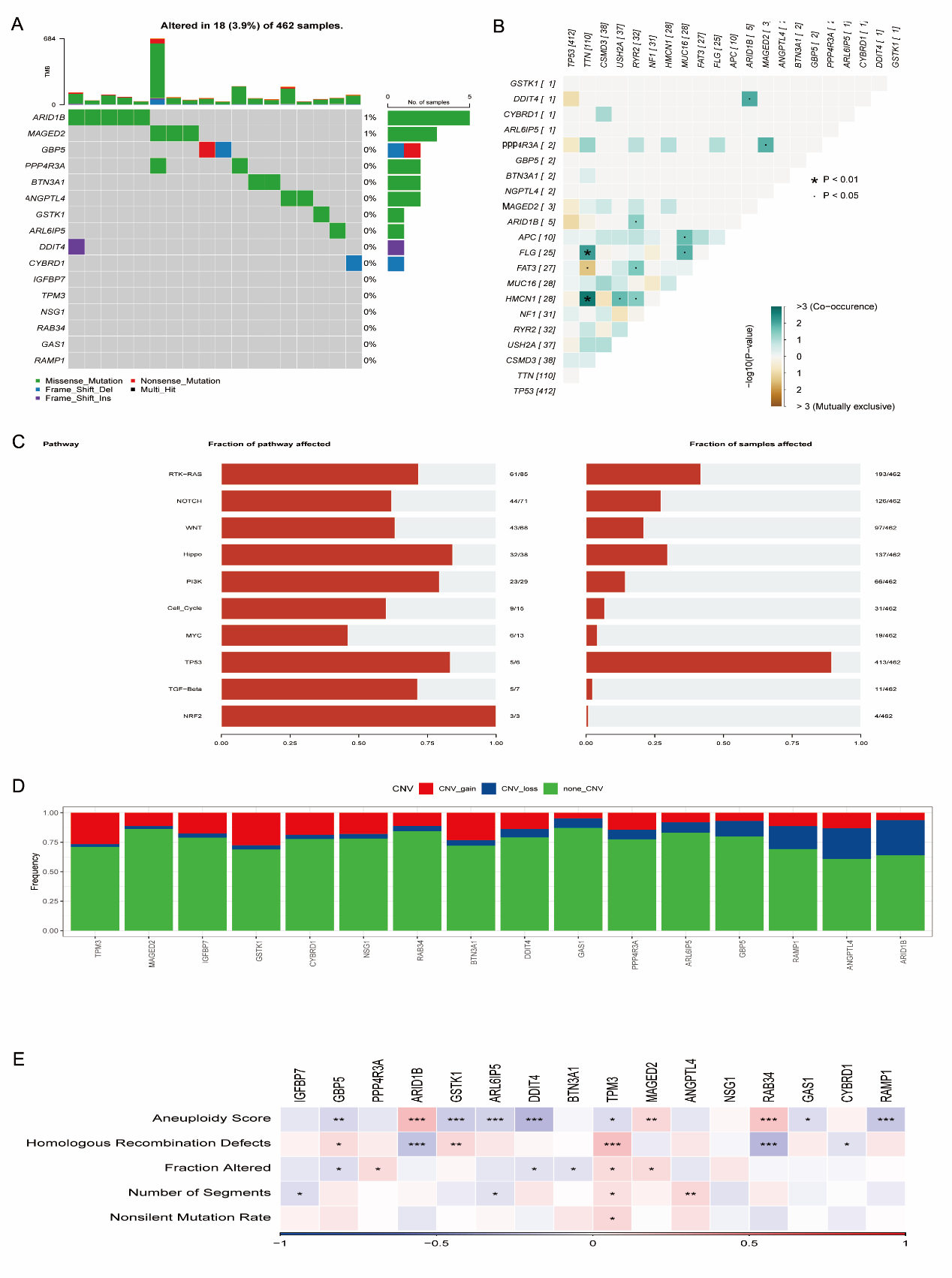
**

Supplementary Figure3**.** Landscape of 16 GRGs mutation profiles. (A) Mutation landscape of 16 GRGs in TCGA-OV dataset. (B) Mutation symbiosis between these 16 GRGs and the TOP10 mutant genes. (C) The mutation frequency of ten common carcinogenic pathways in OC. (D) The copy number variation (CNV) of the 16 GRGs in TCGA-OV dataset. (E) Correlations between the 16 GRGs and aneuploidy score, homologous recombination defects, fraction altered, number of segments and nonsilent mutation rate.
