# Supplementary material for: Glycosylation-related genes mediated prognostic signature contribute to prognostic prediction and treatment options in ovarian cancer: based on bulk and single‑cell RNA sequencing data
Source: BMC Cancer. 2024 Feb 14;24:207. doi: 10.1186/s12885-024-11908-4 (PMC10865697; doi:10.1186/s12885-024-11908-4)
Supplement: Supplementary file 4 — Supplementary Figure 4. The immune infiltrations analysis. (A) Correlations between the 16 GRGs and immune score. (B) Correlations between the 16 GRGs and immune score, stromal score, estimate score. (C) Correlations between 16 GRGs and 22 types of TIICs. [file 12885_2024_11908_MOESM4_ESM.docx]

**
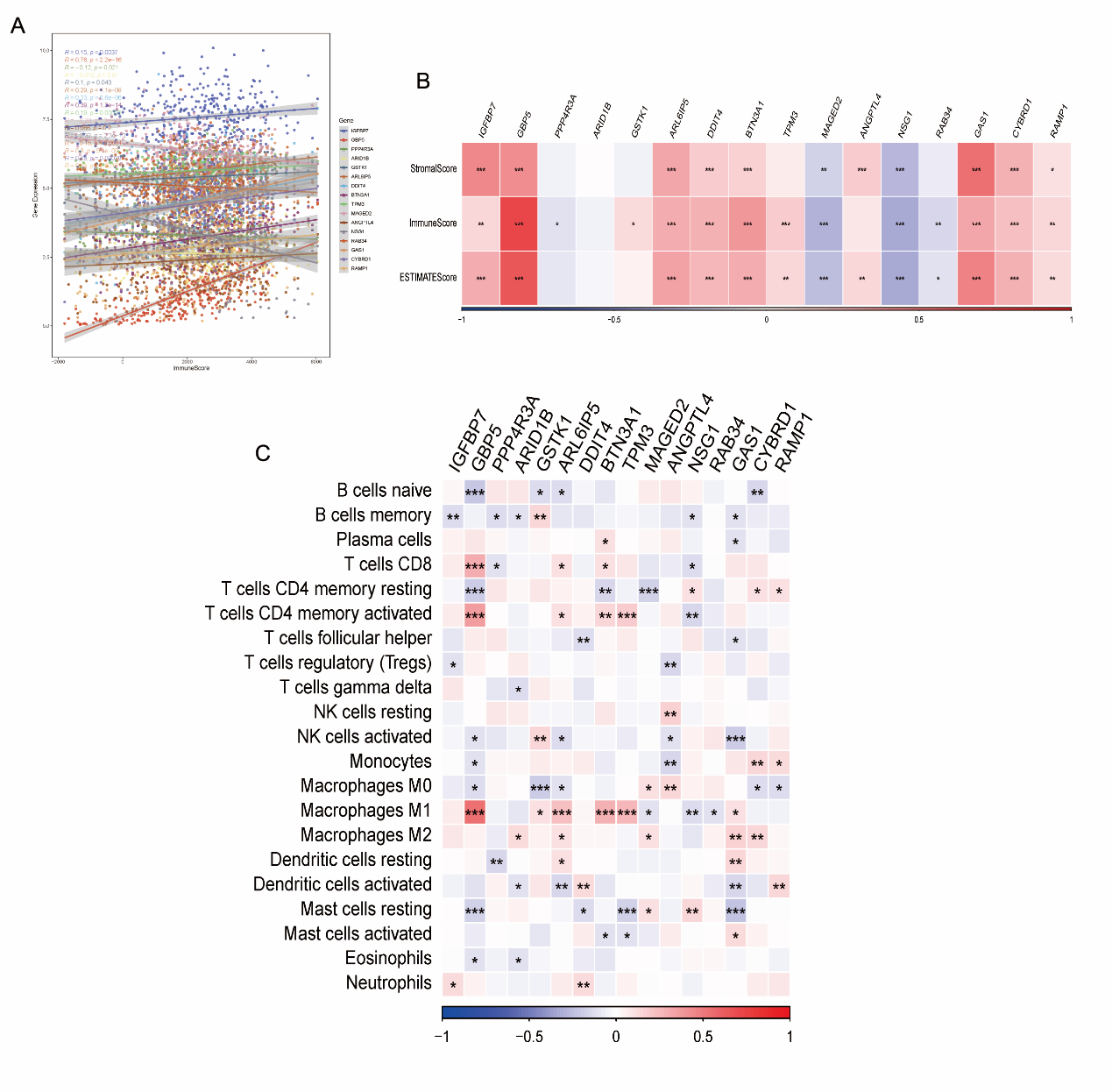
**

Supplementary Figure4**.** The immune infiltrations analysis. (A) Correlations between the 16 GRGs and immune score. (B) Correlations between the 16 GRGs and immune score, stromal score, estimate score. (C) Correlations between 16 GRGs and 22 types of TIICs.
