# Supplementary material for: Glycosylation-related genes mediated prognostic signature contribute to prognostic prediction and treatment options in ovarian cancer: based on bulk and single‑cell RNA sequencing data
Source: BMC Cancer. 2024 Feb 14;24:207. doi: 10.1186/s12885-024-11908-4 (PMC10865697; doi:10.1186/s12885-024-11908-4)
Supplement: Supplementary file 5 — Supplementary Figure 5. The immune infiltrations analysis. (A-B) Correlations between 16 GRGs and TIICs. (C) The t-SNE plots indicated the expression of 16 GRGs in 12 samples. [file 12885_2024_11908_MOESM5_ESM.docx]

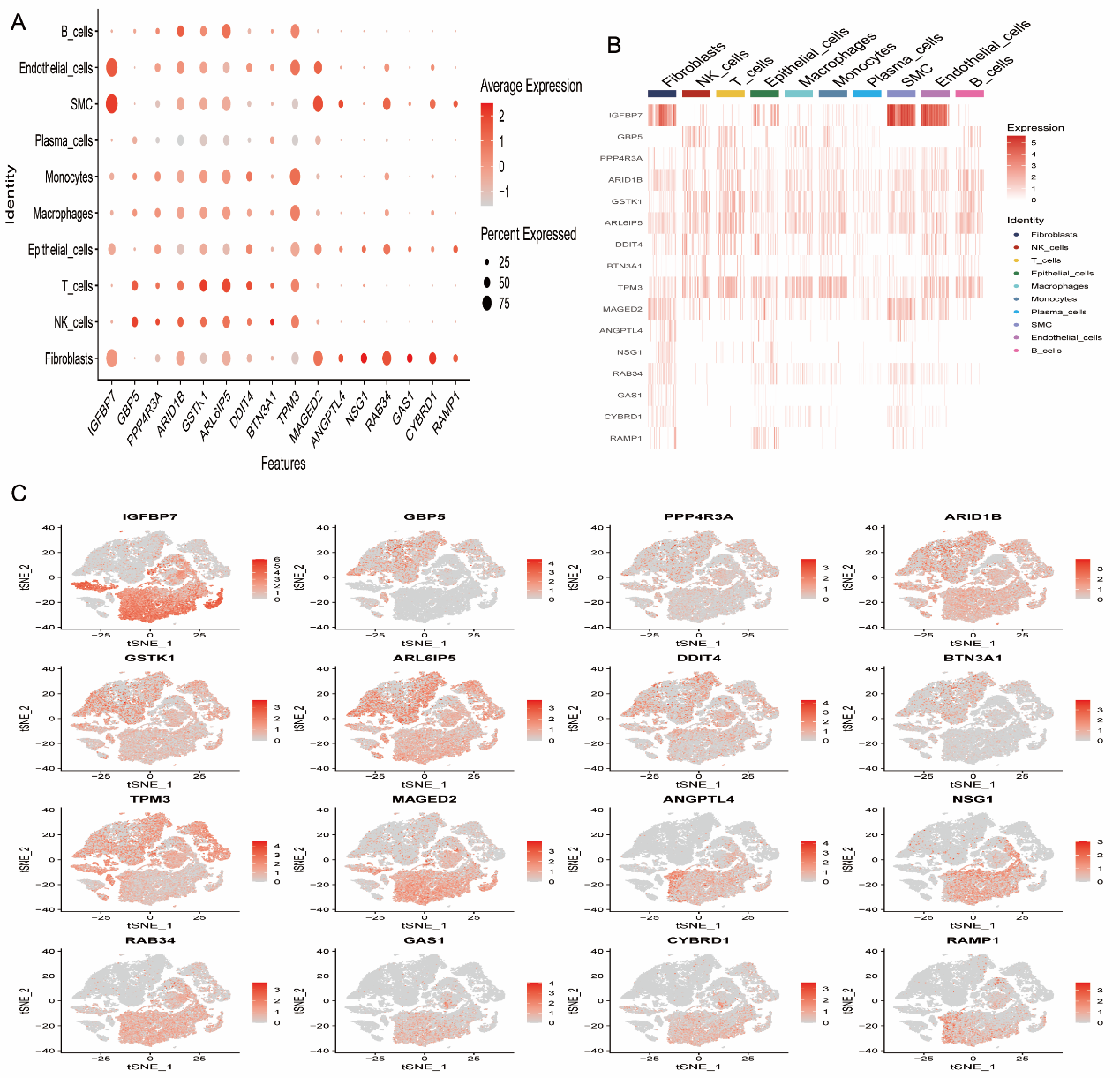


Supplementary Figure5**.** The immune infiltrations analysis. (A-B) Correlations between 16 GRGs and TIICs. (C) The t-SNE plots indicated the expression of 16 GRGs in 12 samples.
