# Supplementary material for: Glycosylation-related genes mediated prognostic signature contribute to prognostic prediction and treatment options in ovarian cancer: based on bulk and single‑cell RNA sequencing data
Source: BMC Cancer. 2024 Feb 14;24:207. doi: 10.1186/s12885-024-11908-4 (PMC10865697; doi:10.1186/s12885-024-11908-4)
Supplement: Supplementary file 6 — Supplementary Table 1. Characteristics of the six datasets. [file 12885_2024_11908_MOESM6_ESM.docx]

Supplementary Table 1. Characteristics of the six datasets.

| **Accession number /Source** | **Cancer** | **Therapy** | **Platform** | **Number of  samples** | **Survival data** |
| --- | --- | --- | --- | --- | --- |
| TCGA-OV | OC | / | Illumina RNAseq | 379 | OS |
| clinical sample | OC | / | Arraystar Human mRNA | 150 | OS |
| GSE63885 | OC | / | Rosetta/Merck Human RSTA Custom Affymetrix 2.0 microarray | 75 | OS |
| GSE184880 | 5 healthy controls and 7 OC patients | / | Illumina NovaSeq 6000 | 12 | - |
| IMvigor210 | BLCA | Anti-PD-L1: atezolizumab | Illumina RNAseq | 348 | OS |
| GSE78220 | Melanoma | Anti-PD-1: pembrolizumab, nivolumab | Illumina HiSeq 2000 (Homo sapiens) | 26 | OS |
