# Supplementary material for: Glycosylation-related genes mediated prognostic signature contribute to prognostic prediction and treatment options in ovarian cancer: based on bulk and single‑cell RNA sequencing data
Source: BMC Cancer. 2024 Feb 14;24:207. doi: 10.1186/s12885-024-11908-4 (PMC10865697; doi:10.1186/s12885-024-11908-4)
Supplement: Supplementary file 7 — Supplementary Table 2. The primer sequences of 16 GRGs genes. [file 12885_2024_11908_MOESM7_ESM.docx]

Supplementary Table 2. The primer sequences of 16 GRGs genes.

| **Gene** | Forward (5′-3′) | Reverse (5′-3′) |
| --- | --- | --- |
| IGFBP7 | 5'- TGCGAGCAAGGTCCTTCCATAG-3' | 5'- GCACTCATATTCTCCAGCATCTTCC-3' |
| GBP5 | 5'- CCTGTAGTTGTGGTAGCGATTGTG-3' | 5'- CGGGTGAGTTTCTTGCCTTGAG-3' |
| PPP4R3A | 5'- CATGGAAGATGGAGAAGCTGTAGTG-3' | 5'- AGATGACTGGCTGGTGAGGTTAG-3' |
| ARID1B | 5'- CTCTCCTGTTGGCTCTCCTGTAG-3' | 5'- TGGCTCATCTGTGGACCGTAAG-3' |
| GSTK1 | 5'- ATCGCAACGCCAAAGGTGAAG-3' | 5'- AGATAGAGAAATCCGAGTCCCAGTG-3' |
| ARL6IP5 | 5'- ATCGTGGTGGTGCTGGTGTTC-3' | 5'- GTGAGTCTGTTGATGCCTTCTTCC-3' |
| DDIT4 | 5'- ACGCACTTGTCTTAGCAGTTCTC-3' | 5'- TCCAGGTAAGCCGTGTCTTCC-3' |
| BTN3A1 | 5'- CTGTAGGTGGTTGTGGAGTTGAC-3' | 5'- GATTGAAGGAAGGCTGGCTCTG-3' |
| TPM3 | 5'- GAAGAAGGCTGCTGATGCTGAG-3' | 5'- GTTCCTCTGTGCGTTCCAAGTC-3' |
| MAGED2 | 5'- GACAATGCCTGCCACTGAGAC-3' | 5'- TCACTGCTGCCATCCTCTTCC-3' |
| ANGPTL4 | 5'- CAAGCCTGCCCGAAGAAAGAG-3' | 5'- GGTTGAAGTCCACTGAGCCATC-3' |
| NSG1 | 5'- AGAGCAAGCCTACTTCGCATTTC-3' | 5'- CTATCAGCACATCACAGAGCAGAC-3' |
| RAB34 | 5'- CTTCCAGTGTGCTTCTCTTCCTTG-3' | 5'- GTTAGGTAGAGGTTGCTGTCATCAC-3' |
| GAS1 | 5'- GGACGACTACTACGATGAGGACTAC-3'， | 5'- CAAGCAGCAGCAGCAAGATGG-3'; |
| CYBRD1 | 5'- CTACTACTGCCAACAAGCCATG-3' | 5'- AAGTCACCTCCAAGTCTCAATTCAC-3' |
| RAMP1 | 5'- GGCAGCATCCTCTACCCCTTC-3' | 5'- CGGCTACTCTGGACTCCTGTG-3' |
| GAPDH | 5'-CAAGGCTGTGGGCAAGGTCATC-3' | 5'-GTGTCGCTGTTGAAGTCAGAGGAG-3' |
