# Supplementary material for: Glycosylation-related genes mediated prognostic signature contribute to prognostic prediction and treatment options in ovarian cancer: based on bulk and single‑cell RNA sequencing data
Source: BMC Cancer. 2024 Feb 14;24:207. doi: 10.1186/s12885-024-11908-4 (PMC10865697; doi:10.1186/s12885-024-11908-4)
Supplement: Supplementary file 8 — Supplementary Table 3. GSVA analysis to explore the underlying biological mechanisms between high and low GRGs_ AUC group. [file 12885_2024_11908_MOESM8_ESM.docx]

Supplementary Table 3. GSVA analysis to explore the underlying biological mechanisms between high and low GRGs_ AUC group.

|  | logFC | AveExpr | t | P.Value | adj.P.Val | B |
| --- | --- | --- | --- | --- | --- | --- |
| ADIPOGENESIS | 0.160896 | 0.201393 | 206.3902 | 0 | 0 | 15360.72 |
| ANDROGEN_RESPONSE | 0.134268 | 0.239966 | 202.872 | 0 | 0 | 14970.51 |
| ANGIOGENESIS | 0.097778 | 0.125771 | 142.5087 | 0 | 0 | 8499.931 |
| APICAL_JUNCTION | 0.103736 | 0.078106 | 196.8053 | 0 | 0 | 14299.19 |
| APICAL_SURFACE | 0.070881 | 0.04295 | 134.5608 | 0 | 0 | 7706.358 |
| APOPTOSIS | 0.113505 | 0.234468 | 186.0501 | 0 | 0 | 13115.74 |
| BILE_ACID_METABOLISM | 0.043718 | 0.104868 | 124.3419 | 0 | 0 | 6718.046 |
| CHOLESTEROL_HOMEOSTASIS | 0.123966 | 0.141286 | 164.528 | 0 | 0 | 10788.66 |
| COAGULATION | 0.093335 | 0.142279 | 157.8025 | 0 | 0 | 10077.54 |
| COMPLEMENT | 0.075276 | 0.201183 | 155.9273 | 0 | 0 | 9880.989 |
| DNA_REPAIR | 0.171035 | 0.159488 | 188.5424 | 0 | 0 | 13389.06 |
| E2F_TARGETS | 0.102106 | 0.112929 | 120.0753 | 0 | 0 | 6317.395 |
| EPITHELIAL_MESENCHYMAL_TRANSITION | 0.143382 | 0.241753 | 149.5936 | 0 | 0 | 9223.244 |
| ESTROGEN_RESPONSE_EARLY | 0.084227 | 0.055676 | 173.2024 | 0 | 0 | 11718.34 |
| ESTROGEN_RESPONSE_LATE | 0.084042 | 0.057377 | 170.9883 | 0 | 0 | 11479.84 |
| FATTY_ACID_METABOLISM | 0.112243 | 0.233125 | 184.3739 | 0 | 0 | 12932.28 |
| G2M_CHECKPOINT | 0.09471 | 0.151778 | 125.3411 | 0 | 0 | 6812.942 |
| GLYCOLYSIS | 0.093128 | 0.205022 | 190.4726 | 0 | 0 | 13601.15 |
| HEDGEHOG_SIGNALING | 0.073784 | 0.049025 | 123.9427 | 0 | 0 | 6680.241 |
| HEME_METABOLISM | 0.093083 | 0.170409 | 192.0156 | 0 | 0 | 13770.94 |
| HYPOXIA | 0.10186 | 0.216405 | 185.7466 | 0 | 0 | 13082.51 |
| IL2_STAT5_SIGNALING | 0.054689 | 0.219674 | 132.1178 | 0 | 0 | 7466.624 |
| IL6_JAK_STAT3_SIGNALING | 0.053437 | 0.166258 | 83.60274 | 0 | 0 | 3258.922 |
| INFLAMMATORY_RESPONSE | 0.021948 | 0.134873 | 43.45711 | 0 | 0 | 916.382 |
| INTERFERON_ALPHA_RESPONSE | 0.074371 | 0.318287 | 81.91813 | 0 | 0 | 3136.434 |
| INTERFERON_GAMMA_RESPONSE | 0.064697 | 0.236713 | 76.76791 | 0 | 0 | 2773.892 |
| KRAS_SIGNALING_DN | -0.03024 | -0.00268 | -152.73 | 0 | 0 | 9547.759 |
| KRAS_SIGNALING_UP | 0.017662 | 0.147754 | 54.72705 | 0 | 0 | 1443.691 |
| MITOTIC_SPINDLE | 0.112348 | 0.137192 | 166.7295 | 0 | 0 | 11023.36 |
| MTORC1_SIGNALING | 0.144094 | 0.268283 | 181.0187 | 0 | 0 | 12566.01 |
| MYC_TARGETS_V1 | 0.226311 | 0.422898 | 197.1017 | 0 | 0 | 14331.94 |
| MYC_TARGETS_V2 | 0.113899 | 0.132148 | 134.3408 | 0 | 0 | 7684.684 |
| MYOGENESIS | 0.090878 | 0.04077 | 166.2473 | 0 | 0 | 10971.88 |
| NOTCH_SIGNALING | 0.083734 | 0.214501 | 129.5157 | 0 | 0 | 7213.596 |
| OXIDATIVE_PHOSPHORYLATION | 0.229034 | 0.327934 | 192.8378 | 0 | 0 | 13861.48 |
| P53_PATHWAY | 0.09424 | 0.174371 | 172.2552 | 0 | 0 | 11616.22 |
| PEROXISOME | 0.084669 | 0.202921 | 172.9274 | 0 | 0 | 11688.68 |
| PI3K_AKT_MTOR_SIGNALING | 0.108404 | 0.21926 | 161.3872 | 0 | 0 | 10455.41 |
| PROTEIN_SECRETION | 0.190623 | 0.279964 | 210.7387 | 0 | 0 | 15843.7 |
| REACTIVE_OXYGEN_SPECIES_PATHWAY | 0.182667 | 0.181014 | 179.8043 | 0 | 0 | 12433.77 |
| TGF_BETA_SIGNALING | 0.164205 | 0.224295 | 187.8334 | 0 | 0 | 13311.25 |
| TNFA_SIGNALING_VIA_NFKB | 0.086927 | 0.237473 | 113.588 | 0 | 0 | 5723.216 |
| UNFOLDED_PROTEIN_RESPONSE | 0.173993 | 0.273036 | 200.9822 | 0 | 0 | 14761.17 |
| UV_RESPONSE_DN | 0.096859 | 0.254326 | 169.0473 | 0 | 0 | 11271.41 |
| UV_RESPONSE_UP | 0.103682 | 0.207553 | 180.1642 | 0 | 0 | 12472.94 |
| WNT_BETA_CATENIN_SIGNALING | 0.064435 | 0.087491 | 116.5077 | 0 | 0 | 5988.306 |
| XENOBIOTIC_METABOLISM | 0.102898 | 0.097634 | 191.4061 | 0 | 0 | 13703.84 |
| ALLOGRAFT_REJECTION | -0.01245 | 0.206012 | -17.526 | 1.46E-68 | 1.52E-68 | 142.7679 |
| PANCREAS_BETA_CELLS | 0.004765 | 0.104059 | 15.31146 | 8.47E-53 | 8.64E-53 | 106.6057 |
| SPERMATOGENESIS | -0.00031 | 0.041323 | -1.48794 | 0.136773 | 0.136773 | -9.23256 |
