# Supplementary material for: Glycosylation-related genes mediated prognostic signature contribute to prognostic prediction and treatment options in ovarian cancer: based on bulk and single‑cell RNA sequencing data
Source: BMC Cancer. 2024 Feb 14;24:207. doi: 10.1186/s12885-024-11908-4 (PMC10865697; doi:10.1186/s12885-024-11908-4)
Supplement: Supplementary file 9 — Supplementary Table 4. GO functional enrichment analyses were used to explore the potential functions of 1187 genes. [file 12885_2024_11908_MOESM9_ESM.docx]

Supplementary Table 4. GO functional enrichment analyses were used to explore the potential functions of 1187 genes.

| ONTOLOGY | ID | Description | pvalue | p.adjust | qvalue |
| --- | --- | --- | --- | --- | --- |
| BP | GO:0030198 | extracellular matrix organization | 2.09E-32 | 7.36E-29 | 5.39E-29 |
| BP | GO:0043062 | extracellular structure organization | 2.67E-32 | 7.36E-29 | 5.39E-29 |
| BP | GO:0045229 | external encapsulating structure organization | 4.36E-32 | 8.01E-29 | 5.86E-29 |
| BP | GO:0031589 | cell-substrate adhesion | 8.38E-21 | 1.16E-17 | 8.46E-18 |
| BP | GO:0030199 | collagen fibril organization | 2.90E-17 | 3.20E-14 | 2.34E-14 |
| BP | GO:0042060 | wound healing | 8.46E-17 | 7.78E-14 | 5.69E-14 |
| BP | GO:0019058 | viral life cycle | 1.25E-15 | 8.71E-13 | 6.38E-13 |
| BP | GO:0007015 | actin filament organization | 1.26E-15 | 8.71E-13 | 6.38E-13 |
| BP | GO:0016032 | viral process | 2.93E-15 | 1.79E-12 | 1.31E-12 |
| BP | GO:0045785 | positive regulation of cell adhesion | 3.88E-15 | 2.05E-12 | 1.50E-12 |
| BP | GO:0032963 | collagen metabolic process | 4.33E-15 | 2.05E-12 | 1.50E-12 |
| BP | GO:0007160 | cell-matrix adhesion | 4.45E-15 | 2.05E-12 | 1.50E-12 |
| BP | GO:0007229 | integrin-mediated signaling pathway | 1.58E-13 | 6.72E-11 | 4.92E-11 |
| BP | GO:1903053 | regulation of extracellular matrix organization | 2.33E-13 | 9.16E-11 | 6.71E-11 |
| BP | GO:0046718 | viral entry into host cell | 1.01E-12 | 3.70E-10 | 2.71E-10 |
| BP | GO:1902903 | regulation of supramolecular fiber organization | 1.64E-12 | 5.67E-10 | 4.15E-10 |
| BP | GO:0071559 | response to transforming growth factor beta | 1.89E-12 | 6.12E-10 | 4.48E-10 |
| BP | GO:0052547 | regulation of peptidase activity | 2.06E-12 | 6.31E-10 | 4.62E-10 |
| BP | GO:0002683 | negative regulation of immune system process | 2.19E-12 | 6.36E-10 | 4.66E-10 |
| BP | GO:0009615 | response to virus | 2.69E-12 | 7.41E-10 | 5.42E-10 |
| BP | GO:0044409 | entry into host | 4.09E-12 | 1.07E-09 | 7.85E-10 |
| BP | GO:0032970 | regulation of actin filament-based process | 5.96E-12 | 1.49E-09 | 1.09E-09 |
| BP | GO:0071560 | cellular response to transforming growth factor beta stimulus | 1.10E-11 | 2.65E-09 | 1.94E-09 |
| BP | GO:0022604 | regulation of cell morphogenesis | 1.44E-11 | 3.32E-09 | 2.43E-09 |
| BP | GO:0044000 | movement in host | 1.79E-11 | 3.95E-09 | 2.89E-09 |
| BP | GO:0032956 | regulation of actin cytoskeleton organization | 1.93E-11 | 4.09E-09 | 2.99E-09 |
| BP | GO:0085029 | extracellular matrix assembly | 2.69E-11 | 5.50E-09 | 4.03E-09 |
| BP | GO:0010810 | regulation of cell-substrate adhesion | 3.03E-11 | 5.96E-09 | 4.36E-09 |
| BP | GO:0022407 | regulation of cell-cell adhesion | 4.07E-11 | 7.74E-09 | 5.66E-09 |
| BP | GO:0090130 | tissue migration | 5.10E-11 | 9.07E-09 | 6.64E-09 |
| BP | GO:2001233 | regulation of apoptotic signaling pathway | 5.10E-11 | 9.07E-09 | 6.64E-09 |
| BP | GO:0051607 | defense response to virus | 6.07E-11 | 1.05E-08 | 7.65E-09 |
| BP | GO:0010631 | epithelial cell migration | 6.61E-11 | 1.10E-08 | 8.08E-09 |
| BP | GO:0140546 | defense response to symbiont | 6.83E-11 | 1.11E-08 | 8.11E-09 |
| BP | GO:0090132 | epithelium migration | 9.08E-11 | 1.43E-08 | 1.05E-08 |
| BP | GO:0000302 | response to reactive oxygen species | 1.23E-10 | 1.88E-08 | 1.38E-08 |
| BP | GO:0051017 | actin filament bundle assembly | 1.27E-10 | 1.89E-08 | 1.39E-08 |
| BP | GO:0051346 | negative regulation of hydrolase activity | 1.38E-10 | 2.00E-08 | 1.46E-08 |
| BP | GO:0001503 | ossification | 1.75E-10 | 2.43E-08 | 1.78E-08 |
| BP | GO:0045936 | negative regulation of phosphate metabolic process | 1.76E-10 | 2.43E-08 | 1.78E-08 |
| BP | GO:0034612 | response to tumor necrosis factor | 1.85E-10 | 2.49E-08 | 1.83E-08 |
| BP | GO:0010563 | negative regulation of phosphorus metabolic process | 1.94E-10 | 2.54E-08 | 1.86E-08 |
| BP | GO:2001236 | regulation of extrinsic apoptotic signaling pathway | 2.36E-10 | 3.02E-08 | 2.21E-08 |
| BP | GO:0061572 | actin filament bundle organization | 2.52E-10 | 3.16E-08 | 2.31E-08 |
| BP | GO:0006979 | response to oxidative stress | 2.79E-10 | 3.42E-08 | 2.51E-08 |
| BP | GO:0050673 | epithelial cell proliferation | 3.54E-10 | 4.25E-08 | 3.11E-08 |
| BP | GO:0051701 | biological process involved in interaction with host | 4.09E-10 | 4.81E-08 | 3.52E-08 |
| BP | GO:0071711 | basement membrane organization | 4.28E-10 | 4.92E-08 | 3.60E-08 |
| BP | GO:0097191 | extrinsic apoptotic signaling pathway | 5.60E-10 | 6.31E-08 | 4.62E-08 |
| BP | GO:0071356 | cellular response to tumor necrosis factor | 5.94E-10 | 6.55E-08 | 4.79E-08 |
| BP | GO:0007179 | transforming growth factor beta receptor signaling pathway | 6.85E-10 | 7.41E-08 | 5.42E-08 |
| BP | GO:0018126 | protein hydroxylation | 6.98E-10 | 7.41E-08 | 5.42E-08 |
| BP | GO:0001666 | response to hypoxia | 8.03E-10 | 8.36E-08 | 6.12E-08 |
| BP | GO:0008360 | regulation of cell shape | 8.67E-10 | 8.86E-08 | 6.48E-08 |
| BP | GO:0007178 | transmembrane receptor protein serine/threonine kinase signaling pathway | 2.08E-09 | 2.09E-07 | 1.53E-07 |
| BP | GO:0150115 | cell-substrate junction organization | 2.15E-09 | 2.12E-07 | 1.55E-07 |
| BP | GO:0001706 | endoderm formation | 2.22E-09 | 2.15E-07 | 1.57E-07 |
| BP | GO:0070482 | response to oxygen levels | 2.27E-09 | 2.15E-07 | 1.57E-07 |
| BP | GO:0001667 | ameboidal-type cell migration | 2.30E-09 | 2.15E-07 | 1.57E-07 |
| BP | GO:2000146 | negative regulation of cell motility | 3.00E-09 | 2.76E-07 | 2.02E-07 |
| BP | GO:0052548 | regulation of endopeptidase activity | 3.21E-09 | 2.90E-07 | 2.12E-07 |
| BP | GO:0036293 | response to decreased oxygen levels | 3.30E-09 | 2.93E-07 | 2.15E-07 |
| BP | GO:0061041 | regulation of wound healing | 3.94E-09 | 3.45E-07 | 2.53E-07 |
| BP | GO:0010632 | regulation of epithelial cell migration | 4.96E-09 | 4.27E-07 | 3.13E-07 |
| BP | GO:0050852 | T cell receptor signaling pathway | 5.54E-09 | 4.70E-07 | 3.44E-07 |
| BP | GO:0007159 | leukocyte cell-cell adhesion | 6.63E-09 | 5.54E-07 | 4.05E-07 |
| BP | GO:0001959 | regulation of cytokine-mediated signaling pathway | 6.78E-09 | 5.54E-07 | 4.05E-07 |
| BP | GO:0042326 | negative regulation of phosphorylation | 6.83E-09 | 5.54E-07 | 4.05E-07 |
| BP | GO:0035987 | endodermal cell differentiation | 7.57E-09 | 6.05E-07 | 4.43E-07 |
| BP | GO:0007596 | blood coagulation | 7.73E-09 | 6.10E-07 | 4.46E-07 |
| BP | GO:0044403 | biological process involved in symbiotic interaction | 7.96E-09 | 6.18E-07 | 4.53E-07 |
| BP | GO:0001819 | positive regulation of cytokine production | 8.90E-09 | 6.82E-07 | 4.99E-07 |
| BP | GO:0010811 | positive regulation of cell-substrate adhesion | 9.58E-09 | 7.23E-07 | 5.29E-07 |
| BP | GO:0032103 | positive regulation of response to external stimulus | 9.70E-09 | 7.23E-07 | 5.29E-07 |
| BP | GO:0060759 | regulation of response to cytokine stimulus | 1.01E-08 | 7.33E-07 | 5.36E-07 |
| BP | GO:0040013 | negative regulation of locomotion | 1.02E-08 | 7.33E-07 | 5.36E-07 |
| BP | GO:0110053 | regulation of actin filament organization | 1.02E-08 | 7.33E-07 | 5.36E-07 |
| BP | GO:0032964 | collagen biosynthetic process | 1.05E-08 | 7.45E-07 | 5.45E-07 |
| BP | GO:0050792 | regulation of viral process | 1.25E-08 | 8.75E-07 | 6.40E-07 |
| BP | GO:1903034 | regulation of response to wounding | 1.40E-08 | 9.65E-07 | 7.06E-07 |
| BP | GO:0050817 | coagulation | 1.42E-08 | 9.65E-07 | 7.06E-07 |
| BP | GO:0030336 | negative regulation of cell migration | 1.55E-08 | 1.04E-06 | 7.62E-07 |
| BP | GO:0007599 | hemostasis | 1.60E-08 | 1.06E-06 | 7.76E-07 |
| BP | GO:0007044 | cell-substrate junction assembly | 1.66E-08 | 1.09E-06 | 7.96E-07 |
| BP | GO:0051604 | protein maturation | 1.68E-08 | 1.09E-06 | 7.96E-07 |
| BP | GO:0050678 | regulation of epithelial cell proliferation | 1.72E-08 | 1.11E-06 | 8.09E-07 |
| BP | GO:0006457 | protein folding | 2.02E-08 | 1.27E-06 | 9.27E-07 |
| BP | GO:0090287 | regulation of cellular response to growth factor stimulus | 2.02E-08 | 1.27E-06 | 9.27E-07 |
| BP | GO:0019079 | viral genome replication | 2.26E-08 | 1.38E-06 | 1.01E-06 |
| BP | GO:0042542 | response to hydrogen peroxide | 2.26E-08 | 1.38E-06 | 1.01E-06 |
| BP | GO:1902905 | positive regulation of supramolecular fiber organization | 2.45E-08 | 1.49E-06 | 1.09E-06 |
| BP | GO:0007162 | negative regulation of cell adhesion | 2.55E-08 | 1.53E-06 | 1.12E-06 |
| BP | GO:0042692 | muscle cell differentiation | 2.92E-08 | 1.73E-06 | 1.27E-06 |
| BP | GO:0050867 | positive regulation of cell activation | 3.03E-08 | 1.78E-06 | 1.30E-06 |
| BP | GO:1903844 | regulation of cellular response to transforming growth factor beta stimulus | 3.18E-08 | 1.85E-06 | 1.35E-06 |
| BP | GO:1903037 | regulation of leukocyte cell-cell adhesion | 3.41E-08 | 1.96E-06 | 1.44E-06 |
| BP | GO:0007492 | endoderm development | 3.62E-08 | 2.06E-06 | 1.51E-06 |
| BP | GO:1903900 | regulation of viral life cycle | 3.74E-08 | 2.10E-06 | 1.54E-06 |
| BP | GO:0032102 | negative regulation of response to external stimulus | 4.06E-08 | 2.26E-06 | 1.66E-06 |
| BP | GO:0043542 | endothelial cell migration | 4.18E-08 | 2.30E-06 | 1.69E-06 |
| BP | GO:0019221 | cytokine-mediated signaling pathway | 4.57E-08 | 2.50E-06 | 1.83E-06 |
| BP | GO:0031099 | regeneration | 5.69E-08 | 3.06E-06 | 2.24E-06 |
| BP | GO:0010466 | negative regulation of peptidase activity | 5.72E-08 | 3.06E-06 | 2.24E-06 |
| BP | GO:0042098 | T cell proliferation | 5.86E-08 | 3.11E-06 | 2.28E-06 |
| BP | GO:0002831 | regulation of response to biotic stimulus | 5.92E-08 | 3.11E-06 | 2.28E-06 |
| BP | GO:0045861 | negative regulation of proteolysis | 7.15E-08 | 3.72E-06 | 2.72E-06 |
| BP | GO:0034446 | substrate adhesion-dependent cell spreading | 7.29E-08 | 3.76E-06 | 2.75E-06 |
| BP | GO:0017015 | regulation of transforming growth factor beta receptor signaling pathway | 7.92E-08 | 4.04E-06 | 2.96E-06 |
| BP | GO:0043254 | regulation of protein-containing complex assembly | 7.99E-08 | 4.04E-06 | 2.96E-06 |
| BP | GO:0045069 | regulation of viral genome replication | 8.43E-08 | 4.20E-06 | 3.07E-06 |
| BP | GO:0045071 | negative regulation of viral genome replication | 8.44E-08 | 4.20E-06 | 3.07E-06 |
| BP | GO:0006898 | receptor-mediated endocytosis | 9.12E-08 | 4.49E-06 | 3.29E-06 |
| BP | GO:0007369 | gastrulation | 9.35E-08 | 4.56E-06 | 3.34E-06 |
| BP | GO:1903317 | regulation of protein maturation | 9.46E-08 | 4.58E-06 | 3.35E-06 |
| BP | GO:0007266 | Rho protein signal transduction | 1.09E-07 | 5.23E-06 | 3.83E-06 |
| BP | GO:0060485 | mesenchyme development | 1.14E-07 | 5.43E-06 | 3.98E-06 |
| BP | GO:0002696 | positive regulation of leukocyte activation | 1.18E-07 | 5.58E-06 | 4.08E-06 |
| BP | GO:0016485 | protein processing | 1.31E-07 | 6.13E-06 | 4.49E-06 |
| BP | GO:0001933 | negative regulation of protein phosphorylation | 1.47E-07 | 6.83E-06 | 5.00E-06 |
| BP | GO:0045862 | positive regulation of proteolysis | 1.49E-07 | 6.87E-06 | 5.03E-06 |
| BP | GO:0002181 | cytoplasmic translation | 1.52E-07 | 6.94E-06 | 5.08E-06 |
| BP | GO:0010951 | negative regulation of endopeptidase activity | 1.61E-07 | 7.27E-06 | 5.32E-06 |
| BP | GO:1901653 | cellular response to peptide | 1.62E-07 | 7.27E-06 | 5.32E-06 |
| BP | GO:0050866 | negative regulation of cell activation | 1.64E-07 | 7.31E-06 | 5.35E-06 |
| BP | GO:0070613 | regulation of protein processing | 1.70E-07 | 7.47E-06 | 5.46E-06 |
| BP | GO:0002832 | negative regulation of response to biotic stimulus | 1.71E-07 | 7.47E-06 | 5.46E-06 |
| BP | GO:0061448 | connective tissue development | 1.73E-07 | 7.53E-06 | 5.51E-06 |
| BP | GO:0002253 | activation of immune response | 1.76E-07 | 7.60E-06 | 5.57E-06 |
| BP | GO:0050818 | regulation of coagulation | 1.85E-07 | 7.89E-06 | 5.77E-06 |
| BP | GO:0048525 | negative regulation of viral process | 1.86E-07 | 7.89E-06 | 5.77E-06 |
| BP | GO:2001234 | negative regulation of apoptotic signaling pathway | 1.97E-07 | 8.31E-06 | 6.08E-06 |
| BP | GO:0050821 | protein stabilization | 2.36E-07 | 9.85E-06 | 7.21E-06 |
| BP | GO:1901342 | regulation of vasculature development | 2.39E-07 | 9.93E-06 | 7.27E-06 |
| BP | GO:0050878 | regulation of body fluid levels | 2.52E-07 | 1.04E-05 | 7.59E-06 |
| BP | GO:0001649 | osteoblast differentiation | 2.57E-07 | 1.05E-05 | 7.68E-06 |
| BP | GO:0002443 | leukocyte mediated immunity | 3.02E-07 | 1.23E-05 | 8.98E-06 |
| BP | GO:0030111 | regulation of Wnt signaling pathway | 3.24E-07 | 1.30E-05 | 9.55E-06 |
| BP | GO:0030193 | regulation of blood coagulation | 3.36E-07 | 1.34E-05 | 9.84E-06 |
| BP | GO:0070661 | leukocyte proliferation | 3.43E-07 | 1.36E-05 | 9.96E-06 |
| BP | GO:1990778 | protein localization to cell periphery | 3.52E-07 | 1.39E-05 | 1.02E-05 |
| BP | GO:0048144 | fibroblast proliferation | 3.56E-07 | 1.39E-05 | 1.02E-05 |
| BP | GO:0045765 | regulation of angiogenesis | 3.72E-07 | 1.45E-05 | 1.06E-05 |
| BP | GO:0048145 | regulation of fibroblast proliferation | 4.16E-07 | 1.61E-05 | 1.18E-05 |
| BP | GO:0010718 | positive regulation of epithelial to mesenchymal transition | 4.19E-07 | 1.61E-05 | 1.18E-05 |
| BP | GO:0018208 | peptidyl-proline modification | 4.27E-07 | 1.62E-05 | 1.19E-05 |
| BP | GO:0002474 | antigen processing and presentation of peptide antigen via MHC class I | 4.49E-07 | 1.70E-05 | 1.24E-05 |
| BP | GO:0016049 | cell growth | 4.54E-07 | 1.70E-05 | 1.25E-05 |
| BP | GO:0050900 | leukocyte migration | 4.65E-07 | 1.73E-05 | 1.27E-05 |
| BP | GO:0002685 | regulation of leukocyte migration | 4.79E-07 | 1.77E-05 | 1.30E-05 |
| BP | GO:0072659 | protein localization to plasma membrane | 4.88E-07 | 1.79E-05 | 1.31E-05 |
| BP | GO:0050863 | regulation of T cell activation | 5.13E-07 | 1.87E-05 | 1.37E-05 |
| BP | GO:1900046 | regulation of hemostasis | 5.19E-07 | 1.88E-05 | 1.38E-05 |
| BP | GO:0019511 | peptidyl-proline hydroxylation | 5.40E-07 | 1.94E-05 | 1.42E-05 |
| BP | GO:0032271 | regulation of protein polymerization | 5.40E-07 | 1.94E-05 | 1.42E-05 |
| BP | GO:0048251 | elastic fiber assembly | 5.66E-07 | 2.02E-05 | 1.47E-05 |
| BP | GO:1903131 | mononuclear cell differentiation | 6.02E-07 | 2.12E-05 | 1.55E-05 |
| BP | GO:0022408 | negative regulation of cell-cell adhesion | 6.04E-07 | 2.12E-05 | 1.55E-05 |
| BP | GO:0022409 | positive regulation of cell-cell adhesion | 6.65E-07 | 2.32E-05 | 1.70E-05 |
| BP | GO:0031032 | actomyosin structure organization | 7.52E-07 | 2.61E-05 | 1.91E-05 |
| BP | GO:2001237 | negative regulation of extrinsic apoptotic signaling pathway | 7.85E-07 | 2.71E-05 | 1.98E-05 |
| BP | GO:0046631 | alpha-beta T cell activation | 7.91E-07 | 2.71E-05 | 1.98E-05 |
| BP | GO:0019882 | antigen processing and presentation | 8.15E-07 | 2.77E-05 | 2.03E-05 |
| BP | GO:0031647 | regulation of protein stability | 8.53E-07 | 2.89E-05 | 2.11E-05 |
| BP | GO:0007517 | muscle organ development | 8.62E-07 | 2.90E-05 | 2.12E-05 |
| BP | GO:0006909 | phagocytosis | 8.76E-07 | 2.93E-05 | 2.14E-05 |
| BP | GO:0001704 | formation of primary germ layer | 9.25E-07 | 3.07E-05 | 2.25E-05 |
| BP | GO:0031638 | zymogen activation | 9.49E-07 | 3.13E-05 | 2.29E-05 |
| BP | GO:0010712 | regulation of collagen metabolic process | 9.59E-07 | 3.15E-05 | 2.30E-05 |
| BP | GO:0010634 | positive regulation of epithelial cell migration | 1.00E-06 | 3.27E-05 | 2.39E-05 |
| BP | GO:0046651 | lymphocyte proliferation | 1.06E-06 | 3.44E-05 | 2.52E-05 |
| BP | GO:0031348 | negative regulation of defense response | 1.10E-06 | 3.56E-05 | 2.60E-05 |
| BP | GO:0045088 | regulation of innate immune response | 1.17E-06 | 3.77E-05 | 2.76E-05 |
| BP | GO:0048762 | mesenchymal cell differentiation | 1.31E-06 | 4.19E-05 | 3.07E-05 |
| BP | GO:0048041 | focal adhesion assembly | 1.35E-06 | 4.27E-05 | 3.13E-05 |
| BP | GO:1903055 | positive regulation of extracellular matrix organization | 1.36E-06 | 4.28E-05 | 3.13E-05 |
| BP | GO:0030865 | cortical cytoskeleton organization | 1.39E-06 | 4.32E-05 | 3.16E-05 |
| BP | GO:0034113 | heterotypic cell-cell adhesion | 1.39E-06 | 4.32E-05 | 3.16E-05 |
| BP | GO:0001837 | epithelial to mesenchymal transition | 1.43E-06 | 4.43E-05 | 3.24E-05 |
| BP | GO:1903039 | positive regulation of leukocyte cell-cell adhesion | 1.44E-06 | 4.44E-05 | 3.25E-05 |
| BP | GO:0150116 | regulation of cell-substrate junction organization | 1.45E-06 | 4.44E-05 | 3.25E-05 |
| BP | GO:0050727 | regulation of inflammatory response | 1.48E-06 | 4.52E-05 | 3.30E-05 |
| BP | GO:0008154 | actin polymerization or depolymerization | 1.49E-06 | 4.52E-05 | 3.30E-05 |
| BP | GO:0030522 | intracellular receptor signaling pathway | 1.73E-06 | 5.21E-05 | 3.82E-05 |
| BP | GO:2001044 | regulation of integrin-mediated signaling pathway | 1.74E-06 | 5.21E-05 | 3.82E-05 |
| BP | GO:0019885 | antigen processing and presentation of endogenous peptide antigen via MHC class I | 1.84E-06 | 5.49E-05 | 4.01E-05 |
| BP | GO:0032943 | mononuclear cell proliferation | 1.89E-06 | 5.62E-05 | 4.11E-05 |
| BP | GO:0034329 | cell junction assembly | 1.95E-06 | 5.74E-05 | 4.20E-05 |
| BP | GO:0042129 | regulation of T cell proliferation | 1.99E-06 | 5.83E-05 | 4.27E-05 |
| BP | GO:0090092 | regulation of transmembrane receptor protein serine/threonine kinase signaling pathway | 2.00E-06 | 5.85E-05 | 4.28E-05 |
| BP | GO:2000116 | regulation of cysteine-type endopeptidase activity | 2.05E-06 | 5.94E-05 | 4.35E-05 |
| BP | GO:0016055 | Wnt signaling pathway | 2.18E-06 | 6.30E-05 | 4.61E-05 |
| BP | GO:0010952 | positive regulation of peptidase activity | 2.38E-06 | 6.84E-05 | 5.00E-05 |
| BP | GO:0198738 | cell-cell signaling by wnt | 2.48E-06 | 7.09E-05 | 5.19E-05 |
| BP | GO:0048545 | response to steroid hormone | 2.58E-06 | 7.34E-05 | 5.37E-05 |
| BP | GO:0001952 | regulation of cell-matrix adhesion | 2.60E-06 | 7.36E-05 | 5.39E-05 |
| BP | GO:1903054 | negative regulation of extracellular matrix organization | 2.66E-06 | 7.42E-05 | 5.43E-05 |
| BP | GO:2001046 | positive regulation of integrin-mediated signaling pathway | 2.66E-06 | 7.42E-05 | 5.43E-05 |
| BP | GO:0048002 | antigen processing and presentation of peptide antigen | 2.66E-06 | 7.42E-05 | 5.43E-05 |
| BP | GO:0051251 | positive regulation of lymphocyte activation | 2.96E-06 | 8.19E-05 | 6.00E-05 |
| BP | GO:0010755 | regulation of plasminogen activation | 3.14E-06 | 8.67E-05 | 6.35E-05 |
| BP | GO:0031349 | positive regulation of defense response | 3.18E-06 | 8.72E-05 | 6.38E-05 |
| BP | GO:0150076 | neuroinflammatory response | 3.21E-06 | 8.76E-05 | 6.41E-05 |
| BP | GO:0032231 | regulation of actin filament bundle assembly | 3.27E-06 | 8.90E-05 | 6.51E-05 |
| BP | GO:0030512 | negative regulation of transforming growth factor beta receptor signaling pathway | 3.30E-06 | 8.93E-05 | 6.54E-05 |
| BP | GO:0010594 | regulation of endothelial cell migration | 3.56E-06 | 9.52E-05 | 6.97E-05 |
| BP | GO:2001238 | positive regulation of extrinsic apoptotic signaling pathway | 3.56E-06 | 9.52E-05 | 6.97E-05 |
| BP | GO:0035909 | aorta morphogenesis | 3.58E-06 | 9.52E-05 | 6.97E-05 |
| BP | GO:0002449 | lymphocyte mediated immunity | 3.60E-06 | 9.52E-05 | 6.97E-05 |
| BP | GO:0002695 | negative regulation of leukocyte activation | 3.61E-06 | 9.52E-05 | 6.97E-05 |
| BP | GO:0033209 | tumor necrosis factor-mediated signaling pathway | 3.80E-06 | 9.97E-05 | 7.30E-05 |
| BP | GO:0097193 | intrinsic apoptotic signaling pathway | 3.87E-06 | 0.000101 | 7.40E-05 |
| BP | GO:0018108 | peptidyl-tyrosine phosphorylation | 3.89E-06 | 0.000101 | 7.40E-05 |
| BP | GO:0010803 | regulation of tumor necrosis factor-mediated signaling pathway | 3.93E-06 | 0.000102 | 7.46E-05 |
| BP | GO:0018212 | peptidyl-tyrosine modification | 4.47E-06 | 0.000115 | 8.43E-05 |
| BP | GO:0001913 | T cell mediated cytotoxicity | 4.52E-06 | 0.000116 | 8.50E-05 |
| BP | GO:1902904 | negative regulation of supramolecular fiber organization | 4.56E-06 | 0.000117 | 8.53E-05 |
| BP | GO:0003158 | endothelium development | 4.62E-06 | 0.000117 | 8.59E-05 |
| BP | GO:0008064 | regulation of actin polymerization or depolymerization | 4.66E-06 | 0.000118 | 8.62E-05 |
| BP | GO:0030099 | myeloid cell differentiation | 4.75E-06 | 0.00012 | 8.75E-05 |
| BP | GO:0050856 | regulation of T cell receptor signaling pathway | 4.90E-06 | 0.000122 | 8.94E-05 |
| BP | GO:1900026 | positive regulation of substrate adhesion-dependent cell spreading | 4.90E-06 | 0.000122 | 8.94E-05 |
| BP | GO:0002483 | antigen processing and presentation of endogenous peptide antigen | 5.15E-06 | 0.000128 | 9.37E-05 |
| BP | GO:0034614 | cellular response to reactive oxygen species | 5.27E-06 | 0.00013 | 9.54E-05 |
| BP | GO:0043434 | response to peptide hormone | 5.36E-06 | 0.000132 | 9.65E-05 |
| BP | GO:0045446 | endothelial cell differentiation | 5.49E-06 | 0.000135 | 9.85E-05 |
| BP | GO:0060537 | muscle tissue development | 5.71E-06 | 0.000139 | 0.000102 |
| BP | GO:0070663 | regulation of leukocyte proliferation | 5.89E-06 | 0.000143 | 0.000105 |
| BP | GO:0071456 | cellular response to hypoxia | 5.93E-06 | 0.000143 | 0.000105 |
| BP | GO:0031579 | membrane raft organization | 6.10E-06 | 0.000146 | 0.000107 |
| BP | GO:0050860 | negative regulation of T cell receptor signaling pathway | 6.10E-06 | 0.000146 | 0.000107 |
| BP | GO:0044089 | positive regulation of cellular component biogenesis | 6.31E-06 | 0.000151 | 0.00011 |
| BP | GO:0030832 | regulation of actin filament length | 6.54E-06 | 0.000156 | 0.000114 |
| BP | GO:0045824 | negative regulation of innate immune response | 6.69E-06 | 0.000158 | 0.000115 |
| BP | GO:1903036 | positive regulation of response to wounding | 6.69E-06 | 0.000158 | 0.000115 |
| BP | GO:0002456 | T cell mediated immunity | 6.72E-06 | 0.000158 | 0.000115 |
| BP | GO:0007265 | Ras protein signal transduction | 7.20E-06 | 0.000168 | 0.000123 |
| BP | GO:0032835 | glomerulus development | 7.21E-06 | 0.000168 | 0.000123 |
| BP | GO:1900024 | regulation of substrate adhesion-dependent cell spreading | 7.42E-06 | 0.000172 | 0.000126 |
| BP | GO:0051235 | maintenance of location | 7.67E-06 | 0.000177 | 0.00013 |
| BP | GO:0051495 | positive regulation of cytoskeleton organization | 7.77E-06 | 0.000178 | 0.000131 |
| BP | GO:0060326 | cell chemotaxis | 8.01E-06 | 0.000183 | 0.000134 |
| BP | GO:0050670 | regulation of lymphocyte proliferation | 8.05E-06 | 0.000183 | 0.000134 |
| BP | GO:0035455 | response to interferon-alpha | 8.16E-06 | 0.000185 | 0.000135 |
| BP | GO:0050729 | positive regulation of inflammatory response | 8.38E-06 | 0.000189 | 0.000139 |
| BP | GO:0043618 | regulation of transcription from RNA polymerase II promoter in response to stress | 8.52E-06 | 0.000192 | 0.00014 |
| BP | GO:0050870 | positive regulation of T cell activation | 8.64E-06 | 0.000194 | 0.000142 |
| BP | GO:1990748 | cellular detoxification | 8.84E-06 | 0.000197 | 0.000144 |
| BP | GO:0032233 | positive regulation of actin filament bundle assembly | 9.08E-06 | 0.0002 | 0.000147 |
| BP | GO:0035904 | aorta development | 9.08E-06 | 0.0002 | 0.000147 |
| BP | GO:0090303 | positive regulation of wound healing | 9.08E-06 | 0.0002 | 0.000147 |
| BP | GO:2001235 | positive regulation of apoptotic signaling pathway | 9.51E-06 | 0.000209 | 0.000153 |
| BP | GO:0033627 | cell adhesion mediated by integrin | 9.78E-06 | 0.000213 | 0.000156 |
| BP | GO:0035023 | regulation of Rho protein signal transduction | 9.78E-06 | 0.000213 | 0.000156 |
| BP | GO:0051348 | negative regulation of transferase activity | 1.04E-05 | 0.000226 | 0.000165 |
| BP | GO:0061077 | chaperone-mediated protein folding | 1.04E-05 | 0.000226 | 0.000165 |
| BP | GO:0010717 | regulation of epithelial to mesenchymal transition | 1.08E-05 | 0.000233 | 0.00017 |
| BP | GO:0032944 | regulation of mononuclear cell proliferation | 1.14E-05 | 0.000244 | 0.000179 |
| BP | GO:0001655 | urogenital system development | 1.17E-05 | 0.00025 | 0.000183 |
| BP | GO:0031639 | plasminogen activation | 1.26E-05 | 0.000268 | 0.000196 |
| BP | GO:0090101 | negative regulation of transmembrane receptor protein serine/threonine kinase signaling pathway | 1.31E-05 | 0.000277 | 0.000203 |
| BP | GO:0034975 | protein folding in endoplasmic reticulum | 1.32E-05 | 0.000277 | 0.000203 |
| BP | GO:0110011 | regulation of basement membrane organization | 1.32E-05 | 0.000277 | 0.000203 |
| BP | GO:0051893 | regulation of focal adhesion assembly | 1.34E-05 | 0.00028 | 0.000205 |
| BP | GO:0090109 | regulation of cell-substrate junction assembly | 1.34E-05 | 0.00028 | 0.000205 |
| BP | GO:0002687 | positive regulation of leukocyte migration | 1.35E-05 | 0.00028 | 0.000205 |
| BP | GO:0007163 | establishment or maintenance of cell polarity | 1.37E-05 | 0.000285 | 0.000209 |
| BP | GO:0051216 | cartilage development | 1.39E-05 | 0.000286 | 0.00021 |
| BP | GO:0043484 | regulation of RNA splicing | 1.46E-05 | 0.000299 | 0.000219 |
| BP | GO:0036294 | cellular response to decreased oxygen levels | 1.46E-05 | 0.000299 | 0.000219 |
| BP | GO:0055001 | muscle cell development | 1.50E-05 | 0.000306 | 0.000224 |
| BP | GO:0010715 | regulation of extracellular matrix disassembly | 1.52E-05 | 0.000309 | 0.000226 |
| BP | GO:0070371 | ERK1 and ERK2 cascade | 1.54E-05 | 0.000312 | 0.000228 |
| BP | GO:0030041 | actin filament polymerization | 1.55E-05 | 0.000312 | 0.000228 |
| BP | GO:2000117 | negative regulation of cysteine-type endopeptidase activity | 1.56E-05 | 0.000313 | 0.000229 |
| BP | GO:0051146 | striated muscle cell differentiation | 1.58E-05 | 0.000317 | 0.000232 |
| BP | GO:0031334 | positive regulation of protein-containing complex assembly | 1.65E-05 | 0.00033 | 0.000241 |
| BP | GO:0030833 | regulation of actin filament polymerization | 1.72E-05 | 0.000343 | 0.000251 |
| BP | GO:0048608 | reproductive structure development | 1.74E-05 | 0.000343 | 0.000251 |
| BP | GO:0071375 | cellular response to peptide hormone stimulus | 1.74E-05 | 0.000343 | 0.000251 |
| BP | GO:0010595 | positive regulation of endothelial cell migration | 1.95E-05 | 0.000384 | 0.000281 |
| BP | GO:0001961 | positive regulation of cytokine-mediated signaling pathway | 2.06E-05 | 0.000404 | 0.000295 |
| BP | GO:0045669 | positive regulation of osteoblast differentiation | 2.09E-05 | 0.000408 | 0.000299 |
| BP | GO:0045185 | maintenance of protein location | 2.10E-05 | 0.000408 | 0.000299 |
| BP | GO:0062197 | cellular response to chemical stress | 2.17E-05 | 0.000421 | 0.000308 |
| BP | GO:0033673 | negative regulation of kinase activity | 2.28E-05 | 0.000441 | 0.000323 |
| BP | GO:0050764 | regulation of phagocytosis | 2.29E-05 | 0.000442 | 0.000323 |
| BP | GO:0061458 | reproductive system development | 2.32E-05 | 0.000444 | 0.000325 |
| BP | GO:0030178 | negative regulation of Wnt signaling pathway | 2.32E-05 | 0.000444 | 0.000325 |
| BP | GO:0050851 | antigen receptor-mediated signaling pathway | 2.39E-05 | 0.000456 | 0.000334 |
| BP | GO:0051492 | regulation of stress fiber assembly | 2.42E-05 | 0.000456 | 0.000334 |
| BP | GO:0060761 | negative regulation of response to cytokine stimulus | 2.42E-05 | 0.000456 | 0.000334 |
| BP | GO:0010955 | negative regulation of protein processing | 2.42E-05 | 0.000456 | 0.000334 |
| BP | GO:1903318 | negative regulation of protein maturation | 2.42E-05 | 0.000456 | 0.000334 |
| BP | GO:0071675 | regulation of mononuclear cell migration | 2.47E-05 | 0.000461 | 0.000338 |
| BP | GO:0097237 | cellular response to toxic substance | 2.47E-05 | 0.000461 | 0.000338 |
| BP | GO:0071453 | cellular response to oxygen levels | 2.56E-05 | 0.000477 | 0.000349 |
| BP | GO:0034599 | cellular response to oxidative stress | 2.61E-05 | 0.000485 | 0.000355 |
| BP | GO:0110020 | regulation of actomyosin structure organization | 2.62E-05 | 0.000485 | 0.000355 |
| BP | GO:0002062 | chondrocyte differentiation | 2.74E-05 | 0.000504 | 0.000369 |
| BP | GO:0044344 | cellular response to fibroblast growth factor stimulus | 2.74E-05 | 0.000504 | 0.000369 |
| BP | GO:0046640 | regulation of alpha-beta T cell proliferation | 2.99E-05 | 0.000547 | 0.000401 |
| BP | GO:0051250 | negative regulation of lymphocyte activation | 3.02E-05 | 0.00055 | 0.000403 |
| BP | GO:1905517 | macrophage migration | 3.02E-05 | 0.00055 | 0.000403 |
| BP | GO:0043534 | blood vessel endothelial cell migration | 3.11E-05 | 0.000562 | 0.000411 |
| BP | GO:0051099 | positive regulation of binding | 3.11E-05 | 0.000562 | 0.000411 |
| BP | GO:1903706 | regulation of hemopoiesis | 3.26E-05 | 0.000588 | 0.00043 |
| BP | GO:0009612 | response to mechanical stimulus | 3.42E-05 | 0.000614 | 0.00045 |
| BP | GO:0051258 | protein polymerization | 3.70E-05 | 0.000663 | 0.000486 |
| BP | GO:0030574 | collagen catabolic process | 3.75E-05 | 0.000667 | 0.000488 |
| BP | GO:0043620 | regulation of DNA-templated transcription in response to stress | 3.75E-05 | 0.000667 | 0.000488 |
| BP | GO:0048732 | gland development | 3.84E-05 | 0.000679 | 0.000497 |
| BP | GO:0072593 | reactive oxygen species metabolic process | 3.84E-05 | 0.000679 | 0.000497 |
| BP | GO:0034101 | erythrocyte homeostasis | 3.88E-05 | 0.000683 | 0.0005 |
| BP | GO:0060840 | artery development | 3.89E-05 | 0.000683 | 0.0005 |
| BP | GO:0050679 | positive regulation of epithelial cell proliferation | 3.96E-05 | 0.000694 | 0.000508 |
| BP | GO:0048844 | artery morphogenesis | 3.99E-05 | 0.000696 | 0.000509 |
| BP | GO:0072001 | renal system development | 4.04E-05 | 0.000702 | 0.000514 |
| BP | GO:0007568 | aging | 4.06E-05 | 0.000705 | 0.000516 |
| BP | GO:0022617 | extracellular matrix disassembly | 4.37E-05 | 0.000755 | 0.000553 |
| BP | GO:0035767 | endothelial cell chemotaxis | 4.40E-05 | 0.000755 | 0.000553 |
| BP | GO:0150117 | positive regulation of cell-substrate junction organization | 4.40E-05 | 0.000755 | 0.000553 |
| BP | GO:0060828 | regulation of canonical Wnt signaling pathway | 4.45E-05 | 0.000762 | 0.000558 |
| BP | GO:0002718 | regulation of cytokine production involved in immune response | 4.49E-05 | 0.000765 | 0.00056 |
| BP | GO:0001960 | negative regulation of cytokine-mediated signaling pathway | 4.50E-05 | 0.000765 | 0.00056 |
| BP | GO:0002274 | myeloid leukocyte activation | 4.51E-05 | 0.000765 | 0.00056 |
| BP | GO:0043409 | negative regulation of MAPK cascade | 4.54E-05 | 0.000768 | 0.000562 |
| BP | GO:2000377 | regulation of reactive oxygen species metabolic process | 4.61E-05 | 0.000778 | 0.000569 |
| BP | GO:0030098 | lymphocyte differentiation | 4.63E-05 | 0.000778 | 0.00057 |
| BP | GO:0050819 | negative regulation of coagulation | 4.66E-05 | 0.000779 | 0.00057 |
| BP | GO:0061900 | glial cell activation | 4.66E-05 | 0.000779 | 0.00057 |
| BP | GO:0030038 | contractile actin filament bundle assembly | 5.01E-05 | 0.000831 | 0.000608 |
| BP | GO:0043149 | stress fiber assembly | 5.01E-05 | 0.000831 | 0.000608 |
| BP | GO:0048024 | regulation of mRNA splicing, via spliceosome | 5.01E-05 | 0.000831 | 0.000608 |
| BP | GO:0009636 | response to toxic substance | 5.04E-05 | 0.000833 | 0.00061 |
| BP | GO:0006029 | proteoglycan metabolic process | 5.18E-05 | 0.000853 | 0.000624 |
| BP | GO:0031663 | lipopolysaccharide-mediated signaling pathway | 5.21E-05 | 0.000856 | 0.000626 |
| BP | GO:0048193 | Golgi vesicle transport | 5.32E-05 | 0.000872 | 0.000638 |
| BP | GO:0043281 | regulation of cysteine-type endopeptidase activity involved in apoptotic process | 5.35E-05 | 0.000873 | 0.000639 |
| BP | GO:0034114 | regulation of heterotypic cell-cell adhesion | 5.40E-05 | 0.000879 | 0.000644 |
| BP | GO:0010769 | regulation of cell morphogenesis involved in differentiation | 5.52E-05 | 0.000892 | 0.000653 |
| BP | GO:0098869 | cellular oxidant detoxification | 5.52E-05 | 0.000892 | 0.000653 |
| BP | GO:0051098 | regulation of binding | 5.56E-05 | 0.000897 | 0.000657 |
| BP | GO:0002367 | cytokine production involved in immune response | 5.69E-05 | 0.000909 | 0.000665 |
| BP | GO:0051897 | positive regulation of protein kinase B signaling | 5.69E-05 | 0.000909 | 0.000665 |
| BP | GO:0071774 | response to fibroblast growth factor | 5.69E-05 | 0.000909 | 0.000665 |
| BP | GO:0036003 | positive regulation of transcription from RNA polymerase II promoter in response to stress | 5.74E-05 | 0.000915 | 0.00067 |
| BP | GO:0001774 | microglial cell activation | 5.78E-05 | 0.000917 | 0.000671 |
| BP | GO:0046633 | alpha-beta T cell proliferation | 5.78E-05 | 0.000917 | 0.000671 |
| BP | GO:0045667 | regulation of osteoblast differentiation | 5.98E-05 | 0.000946 | 0.000692 |
| BP | GO:0010770 | positive regulation of cell morphogenesis involved in differentiation | 6.27E-05 | 0.000987 | 0.000723 |
| BP | GO:0008037 | cell recognition | 6.44E-05 | 0.001012 | 0.000741 |
| BP | GO:0001954 | positive regulation of cell-matrix adhesion | 6.82E-05 | 0.001067 | 0.000781 |
| BP | GO:0001656 | metanephros development | 6.83E-05 | 0.001067 | 0.000781 |
| BP | GO:0002478 | antigen processing and presentation of exogenous peptide antigen | 6.86E-05 | 0.001069 | 0.000782 |
| BP | GO:0006956 | complement activation | 6.95E-05 | 0.00108 | 0.000791 |
| BP | GO:0008630 | intrinsic apoptotic signaling pathway in response to DNA damage | 7.14E-05 | 0.001106 | 0.00081 |
| BP | GO:0048660 | regulation of smooth muscle cell proliferation | 7.17E-05 | 0.001108 | 0.000811 |
| BP | GO:0060760 | positive regulation of response to cytokine stimulus | 7.35E-05 | 0.001132 | 0.000828 |
| BP | GO:0070372 | regulation of ERK1 and ERK2 cascade | 7.41E-05 | 0.001136 | 0.000831 |
| BP | GO:0019883 | antigen processing and presentation of endogenous antigen | 7.41E-05 | 0.001136 | 0.000831 |
| BP | GO:0048147 | negative regulation of fibroblast proliferation | 7.57E-05 | 0.001154 | 0.000845 |
| BP | GO:0050858 | negative regulation of antigen receptor-mediated signaling pathway | 7.57E-05 | 0.001154 | 0.000845 |
| BP | GO:0050680 | negative regulation of epithelial cell proliferation | 7.86E-05 | 0.001194 | 0.000874 |
| BP | GO:0050921 | positive regulation of chemotaxis | 8.17E-05 | 0.001238 | 0.000906 |
| BP | GO:1903978 | regulation of microglial cell activation | 8.39E-05 | 0.001268 | 0.000928 |
| BP | GO:0150077 | regulation of neuroinflammatory response | 8.61E-05 | 0.001296 | 0.000948 |
| BP | GO:0001909 | leukocyte mediated cytotoxicity | 8.62E-05 | 0.001296 | 0.000948 |
| BP | GO:0050854 | regulation of antigen receptor-mediated signaling pathway | 8.67E-05 | 0.0013 | 0.000951 |
| BP | GO:0019884 | antigen processing and presentation of exogenous antigen | 8.70E-05 | 0.001301 | 0.000952 |
| BP | GO:0070997 | neuron death | 9.25E-05 | 0.001379 | 0.001009 |
| BP | GO:0007565 | female pregnancy | 9.30E-05 | 0.001379 | 0.001009 |
| BP | GO:0071478 | cellular response to radiation | 9.30E-05 | 0.001379 | 0.001009 |
| BP | GO:0050732 | negative regulation of peptidyl-tyrosine phosphorylation | 9.79E-05 | 0.001447 | 0.001059 |
| BP | GO:0042026 | protein refolding | 0.0001 | 0.001475 | 0.00108 |
| BP | GO:0050777 | negative regulation of immune response | 0.000101 | 0.00149 | 0.00109 |
| BP | GO:0034109 | homotypic cell-cell adhesion | 0.000102 | 0.00149 | 0.00109 |
| BP | GO:0048659 | smooth muscle cell proliferation | 0.000103 | 0.001505 | 0.001102 |
| BP | GO:0048872 | homeostasis of number of cells | 0.000105 | 0.001535 | 0.001123 |
| BP | GO:0002269 | leukocyte activation involved in inflammatory response | 0.000106 | 0.001536 | 0.001124 |
| BP | GO:0030195 | negative regulation of blood coagulation | 0.000106 | 0.001536 | 0.001124 |
| BP | GO:0001822 | kidney development | 0.000111 | 0.001605 | 0.001175 |
| BP | GO:0001558 | regulation of cell growth | 0.000111 | 0.001608 | 0.001177 |
| BP | GO:0034976 | response to endoplasmic reticulum stress | 0.000113 | 0.001632 | 0.001194 |
| BP | GO:0051651 | maintenance of location in cell | 0.000116 | 0.001666 | 0.001219 |
| BP | GO:0043666 | regulation of phosphoprotein phosphatase activity | 0.000116 | 0.001666 | 0.001219 |
| BP | GO:0002228 | natural killer cell mediated immunity | 0.000117 | 0.001666 | 0.001219 |
| BP | GO:0042102 | positive regulation of T cell proliferation | 0.000117 | 0.001666 | 0.001219 |
| BP | GO:0060070 | canonical Wnt signaling pathway | 0.000118 | 0.001681 | 0.00123 |
| BP | GO:0035633 | maintenance of blood-brain barrier | 0.000125 | 0.001771 | 0.001296 |
| BP | GO:0001765 | membrane raft assembly | 0.000126 | 0.001775 | 0.001299 |
| BP | GO:2000271 | positive regulation of fibroblast apoptotic process | 0.000126 | 0.001775 | 0.001299 |
| BP | GO:0043277 | apoptotic cell clearance | 0.000128 | 0.001795 | 0.001314 |
| BP | GO:1900047 | negative regulation of hemostasis | 0.000128 | 0.001795 | 0.001314 |
| BP | GO:0046578 | regulation of Ras protein signal transduction | 0.00013 | 0.001827 | 0.001337 |
| BP | GO:0006469 | negative regulation of protein kinase activity | 0.000131 | 0.001835 | 0.001343 |
| BP | GO:0070972 | protein localization to endoplasmic reticulum | 0.000135 | 0.001879 | 0.001375 |
| BP | GO:1901654 | response to ketone | 0.000137 | 0.001903 | 0.001392 |
| BP | GO:0002753 | cytoplasmic pattern recognition receptor signaling pathway | 0.000138 | 0.001913 | 0.0014 |
| BP | GO:0007249 | I-kappaB kinase/NF-kappaB signaling | 0.000138 | 0.001913 | 0.0014 |
| BP | GO:0002460 | adaptive immune response based on somatic recombination of immune receptors built from immunoglobulin superfamily domains | 0.000139 | 0.001914 | 0.001401 |
| BP | GO:1903320 | regulation of protein modification by small protein conjugation or removal | 0.000139 | 0.001915 | 0.001402 |
| BP | GO:1901214 | regulation of neuron death | 0.00014 | 0.001918 | 0.001404 |
| BP | GO:0050684 | regulation of mRNA processing | 0.000144 | 0.001976 | 0.001446 |
| BP | GO:0043491 | protein kinase B signaling | 0.000153 | 0.002088 | 0.001528 |
| BP | GO:0044706 | multi-multicellular organism process | 0.000153 | 0.002088 | 0.001528 |
| BP | GO:0051496 | positive regulation of stress fiber assembly | 0.000154 | 0.00209 | 0.00153 |
| BP | GO:0034341 | response to interferon-gamma | 0.000159 | 0.002161 | 0.001582 |
| BP | GO:0050730 | regulation of peptidyl-tyrosine phosphorylation | 0.00016 | 0.002168 | 0.001587 |
| BP | GO:0003012 | muscle system process | 0.000162 | 0.002181 | 0.001596 |
| BP | GO:0043627 | response to estrogen | 0.000162 | 0.002183 | 0.001597 |
| BP | GO:1905521 | regulation of macrophage migration | 0.000163 | 0.002183 | 0.001597 |
| BP | GO:0043122 | regulation of I-kappaB kinase/NF-kappaB signaling | 0.000172 | 0.002302 | 0.001685 |
| BP | GO:0043410 | positive regulation of MAPK cascade | 0.000175 | 0.002338 | 0.001711 |
| BP | GO:0090090 | negative regulation of canonical Wnt signaling pathway | 0.000176 | 0.002344 | 0.001715 |
| BP | GO:0022411 | cellular component disassembly | 0.000177 | 0.002348 | 0.001719 |
| BP | GO:0070431 | nucleotide-binding oligomerization domain containing 2 signaling pathway | 0.000178 | 0.002357 | 0.001725 |
| BP | GO:0072006 | nephron development | 0.000179 | 0.002362 | 0.001728 |
| BP | GO:0032535 | regulation of cellular component size | 0.000181 | 0.002387 | 0.001747 |
| BP | GO:0052372 | modulation by symbiont of entry into host | 0.000184 | 0.002424 | 0.001774 |
| BP | GO:0060191 | regulation of lipase activity | 0.000187 | 0.002454 | 0.001796 |
| BP | GO:0044703 | multi-organism reproductive process | 0.000188 | 0.002458 | 0.001799 |
| BP | GO:0010975 | regulation of neuron projection development | 0.000198 | 0.002594 | 0.001898 |
| BP | GO:0051101 | regulation of DNA binding | 0.0002 | 0.002602 | 0.001904 |
| BP | GO:0031396 | regulation of protein ubiquitination | 0.000203 | 0.002632 | 0.001926 |
| BP | GO:0045766 | positive regulation of angiogenesis | 0.000203 | 0.002632 | 0.001926 |
| BP | GO:1904018 | positive regulation of vasculature development | 0.000203 | 0.002632 | 0.001926 |
| BP | GO:0031532 | actin cytoskeleton reorganization | 0.000208 | 0.002686 | 0.001966 |
| BP | GO:0001935 | endothelial cell proliferation | 0.000212 | 0.002731 | 0.001998 |
| BP | GO:0010720 | positive regulation of cell development | 0.000212 | 0.002731 | 0.001998 |
| BP | GO:0051494 | negative regulation of cytoskeleton organization | 0.000213 | 0.002734 | 0.002001 |
| BP | GO:0098754 | detoxification | 0.000215 | 0.002748 | 0.002011 |
| BP | GO:0034115 | negative regulation of heterotypic cell-cell adhesion | 0.00022 | 0.002786 | 0.002039 |
| BP | GO:0043320 | natural killer cell degranulation | 0.00022 | 0.002786 | 0.002039 |
| BP | GO:0070587 | regulation of cell-cell adhesion involved in gastrulation | 0.00022 | 0.002786 | 0.002039 |
| BP | GO:0097278 | complement-dependent cytotoxicity | 0.00022 | 0.002786 | 0.002039 |
| BP | GO:0002698 | negative regulation of immune effector process | 0.000222 | 0.002804 | 0.002052 |
| BP | GO:0001764 | neuron migration | 0.000228 | 0.002872 | 0.002102 |
| BP | GO:0001906 | cell killing | 0.000239 | 0.003004 | 0.002199 |
| BP | GO:0051896 | regulation of protein kinase B signaling | 0.000239 | 0.003004 | 0.002199 |
| BP | GO:0032273 | positive regulation of protein polymerization | 0.00024 | 0.003008 | 0.002202 |
| BP | GO:0022612 | gland morphogenesis | 0.000246 | 0.003076 | 0.002251 |
| BP | GO:0071677 | positive regulation of mononuclear cell migration | 0.000251 | 0.003138 | 0.002297 |
| BP | GO:0060562 | epithelial tube morphogenesis | 0.000254 | 0.003163 | 0.002315 |
| BP | GO:0042063 | gliogenesis | 0.000255 | 0.003168 | 0.002319 |
| BP | GO:0010763 | positive regulation of fibroblast migration | 0.000261 | 0.003231 | 0.002365 |
| BP | GO:0033631 | cell-cell adhesion mediated by integrin | 0.000261 | 0.003231 | 0.002365 |
| BP | GO:0042176 | regulation of protein catabolic process | 0.000263 | 0.003251 | 0.002379 |
| BP | GO:1903076 | regulation of protein localization to plasma membrane | 0.000268 | 0.003299 | 0.002414 |
| BP | GO:0048771 | tissue remodeling | 0.000269 | 0.0033 | 0.002415 |
| BP | GO:0070301 | cellular response to hydrogen peroxide | 0.000271 | 0.003323 | 0.002432 |
| BP | GO:0010976 | positive regulation of neuron projection development | 0.000281 | 0.003439 | 0.002517 |
| BP | GO:0009408 | response to heat | 0.000288 | 0.003498 | 0.00256 |
| BP | GO:0061437 | renal system vasculature development | 0.000289 | 0.003498 | 0.00256 |
| BP | GO:0061440 | kidney vasculature development | 0.000289 | 0.003498 | 0.00256 |
| BP | GO:0042267 | natural killer cell mediated cytotoxicity | 0.000289 | 0.003498 | 0.00256 |
| BP | GO:0050766 | positive regulation of phagocytosis | 0.000289 | 0.003498 | 0.00256 |
| BP | GO:0002262 | myeloid cell homeostasis | 0.000301 | 0.003629 | 0.002656 |
| BP | GO:1904375 | regulation of protein localization to cell periphery | 0.000301 | 0.00363 | 0.002657 |
| BP | GO:0010921 | regulation of phosphatase activity | 0.000303 | 0.003633 | 0.002658 |
| BP | GO:0031397 | negative regulation of protein ubiquitination | 0.000303 | 0.003633 | 0.002658 |
| BP | GO:0032507 | maintenance of protein location in cell | 0.000304 | 0.003641 | 0.002665 |
| BP | GO:0051043 | regulation of membrane protein ectodomain proteolysis | 0.000306 | 0.003641 | 0.002665 |
| BP | GO:0035304 | regulation of protein dephosphorylation | 0.000306 | 0.003641 | 0.002665 |
| BP | GO:1901888 | regulation of cell junction assembly | 0.000312 | 0.003707 | 0.002713 |
| BP | GO:0071479 | cellular response to ionizing radiation | 0.000332 | 0.003933 | 0.002879 |
| BP | GO:1901796 | regulation of signal transduction by p53 class mediator | 0.000335 | 0.003968 | 0.002904 |
| BP | GO:0014909 | smooth muscle cell migration | 0.000344 | 0.004062 | 0.002973 |
| BP | GO:0008038 | neuron recognition | 0.000347 | 0.004095 | 0.002997 |
| BP | GO:0034605 | cellular response to heat | 0.000353 | 0.00415 | 0.003037 |
| BP | GO:0031953 | negative regulation of protein autophosphorylation | 0.000359 | 0.004201 | 0.003075 |
| BP | GO:0070586 | cell-cell adhesion involved in gastrulation | 0.000359 | 0.004201 | 0.003075 |
| BP | GO:0097529 | myeloid leukocyte migration | 0.000367 | 0.004288 | 0.003138 |
| BP | GO:0030201 | heparan sulfate proteoglycan metabolic process | 0.000373 | 0.004312 | 0.003155 |
| BP | GO:0030866 | cortical actin cytoskeleton organization | 0.000373 | 0.004312 | 0.003155 |
| BP | GO:0032965 | regulation of collagen biosynthetic process | 0.000373 | 0.004312 | 0.003155 |
| BP | GO:1990000 | amyloid fibril formation | 0.000373 | 0.004312 | 0.003155 |
| BP | GO:0150079 | negative regulation of neuroinflammatory response | 0.000373 | 0.004312 | 0.003155 |
| BP | GO:1905475 | regulation of protein localization to membrane | 0.000386 | 0.004439 | 0.003248 |
| BP | GO:2001242 | regulation of intrinsic apoptotic signaling pathway | 0.000386 | 0.004439 | 0.003248 |
| BP | GO:1903321 | negative regulation of protein modification by small protein conjugation or removal | 0.000386 | 0.004439 | 0.003248 |
| BP | GO:0046641 | positive regulation of alpha-beta T cell proliferation | 0.000403 | 0.004617 | 0.003379 |
| BP | GO:0070423 | nucleotide-binding oligomerization domain containing signaling pathway | 0.000403 | 0.004617 | 0.003379 |
| BP | GO:0071900 | regulation of protein serine/threonine kinase activity | 0.000408 | 0.004656 | 0.003407 |
| BP | GO:0031529 | ruffle organization | 0.000422 | 0.004812 | 0.003522 |
| BP | GO:0071383 | cellular response to steroid hormone stimulus | 0.000451 | 0.005126 | 0.003751 |
| BP | GO:0060348 | bone development | 0.000452 | 0.005131 | 0.003755 |
| BP | GO:0045454 | cell redox homeostasis | 0.000453 | 0.005136 | 0.003759 |
| BP | GO:0014812 | muscle cell migration | 0.000463 | 0.005239 | 0.003834 |
| BP | GO:0030239 | myofibril assembly | 0.000469 | 0.005294 | 0.003874 |
| BP | GO:0010927 | cellular component assembly involved in morphogenesis | 0.000482 | 0.005425 | 0.003971 |
| BP | GO:0048661 | positive regulation of smooth muscle cell proliferation | 0.000484 | 0.005442 | 0.003983 |
| BP | GO:0070555 | response to interleukin-1 | 0.00049 | 0.00549 | 0.004018 |
| BP | GO:0043154 | negative regulation of cysteine-type endopeptidase activity involved in apoptotic process | 0.000492 | 0.005497 | 0.004023 |
| BP | GO:0043903 | regulation of biological process involved in symbiotic interaction | 0.000492 | 0.005497 | 0.004023 |
| BP | GO:0010038 | response to metal ion | 0.000499 | 0.005566 | 0.004073 |
| BP | GO:0030324 | lung development | 0.000506 | 0.005624 | 0.004116 |
| BP | GO:0033002 | muscle cell proliferation | 0.000511 | 0.005669 | 0.004149 |
| BP | GO:2001185 | regulation of CD8-positive, alpha-beta T cell activation | 0.000518 | 0.005724 | 0.004189 |
| BP | GO:0071214 | cellular response to abiotic stimulus | 0.000519 | 0.005724 | 0.004189 |
| BP | GO:0104004 | cellular response to environmental stimulus | 0.000519 | 0.005724 | 0.004189 |
| BP | GO:0060541 | respiratory system development | 0.00052 | 0.005724 | 0.004189 |
| BP | GO:0035872 | nucleotide-binding domain, leucine rich repeat containing receptor signaling pathway | 0.000525 | 0.005732 | 0.004195 |
| BP | GO:0044346 | fibroblast apoptotic process | 0.000525 | 0.005732 | 0.004195 |
| BP | GO:0051894 | positive regulation of focal adhesion assembly | 0.000525 | 0.005732 | 0.004195 |
| BP | GO:0060343 | trabecula formation | 0.000525 | 0.005732 | 0.004195 |
| BP | GO:0055002 | striated muscle cell development | 0.000539 | 0.005873 | 0.004298 |
| BP | GO:0030101 | natural killer cell activation | 0.000541 | 0.005886 | 0.004307 |
| BP | GO:2000403 | positive regulation of lymphocyte migration | 0.000548 | 0.005942 | 0.004349 |
| BP | GO:0032386 | regulation of intracellular transport | 0.000548 | 0.005942 | 0.004349 |
| BP | GO:0031960 | response to corticosteroid | 0.000553 | 0.00597 | 0.004369 |
| BP | GO:0002357 | defense response to tumor cell | 0.000555 | 0.00597 | 0.004369 |
| BP | GO:0035437 | maintenance of protein localization in endoplasmic reticulum | 0.000555 | 0.00597 | 0.004369 |
| BP | GO:0072537 | fibroblast activation | 0.000555 | 0.00597 | 0.004369 |
| BP | GO:0048678 | response to axon injury | 0.000557 | 0.005979 | 0.004376 |
| BP | GO:1904035 | regulation of epithelial cell apoptotic process | 0.000571 | 0.006112 | 0.004473 |
| BP | GO:0002711 | positive regulation of T cell mediated immunity | 0.000572 | 0.006113 | 0.004474 |
| BP | GO:0031623 | receptor internalization | 0.000586 | 0.006243 | 0.004569 |
| BP | GO:0032355 | response to estradiol | 0.000586 | 0.006243 | 0.004569 |
| BP | GO:0043123 | positive regulation of I-kappaB kinase/NF-kappaB signaling | 0.000588 | 0.006248 | 0.004573 |
| BP | GO:0002429 | immune response-activating cell surface receptor signaling pathway | 0.000597 | 0.00632 | 0.004625 |
| BP | GO:0002757 | immune response-activating signal transduction | 0.000597 | 0.00632 | 0.004625 |
| BP | GO:0071347 | cellular response to interleukin-1 | 0.000632 | 0.006675 | 0.004885 |
| BP | GO:0043433 | negative regulation of DNA-binding transcription factor activity | 0.000633 | 0.006678 | 0.004887 |
| BP | GO:0031346 | positive regulation of cell projection organization | 0.000647 | 0.006812 | 0.004985 |
| BP | GO:0070665 | positive regulation of leukocyte proliferation | 0.00065 | 0.006826 | 0.004996 |
| BP | GO:0002347 | response to tumor cell | 0.000658 | 0.006899 | 0.005049 |
| BP | GO:0010875 | positive regulation of cholesterol efflux | 0.000673 | 0.007009 | 0.00513 |
| BP | GO:0032967 | positive regulation of collagen biosynthetic process | 0.000673 | 0.007009 | 0.00513 |
| BP | GO:0036037 | CD8-positive, alpha-beta T cell activation | 0.000673 | 0.007009 | 0.00513 |
| BP | GO:0042730 | fibrinolysis | 0.000673 | 0.007009 | 0.00513 |
| BP | GO:0030323 | respiratory tube development | 0.000681 | 0.007066 | 0.005171 |
| BP | GO:0010639 | negative regulation of organelle organization | 0.000682 | 0.007066 | 0.005171 |
| BP | GO:0048511 | rhythmic process | 0.000695 | 0.007195 | 0.005266 |
| BP | GO:0048259 | regulation of receptor-mediated endocytosis | 0.000698 | 0.007213 | 0.005279 |
| BP | GO:0055093 | response to hyperoxia | 0.000705 | 0.007252 | 0.005307 |
| BP | GO:2000269 | regulation of fibroblast apoptotic process | 0.000705 | 0.007252 | 0.005307 |
| BP | GO:0030595 | leukocyte chemotaxis | 0.000717 | 0.007363 | 0.005389 |
| BP | GO:0050671 | positive regulation of lymphocyte proliferation | 0.000757 | 0.007761 | 0.00568 |
| BP | GO:0031102 | neuron projection regeneration | 0.000763 | 0.007782 | 0.005695 |
| BP | GO:0061614 | miRNA transcription | 0.000763 | 0.007782 | 0.005695 |
| BP | GO:1904036 | negative regulation of epithelial cell apoptotic process | 0.000763 | 0.007782 | 0.005695 |
| BP | GO:0015918 | sterol transport | 0.000773 | 0.007868 | 0.005758 |
| BP | GO:0048588 | developmental cell growth | 0.000777 | 0.007897 | 0.005779 |
| BP | GO:0097581 | lamellipodium organization | 0.000783 | 0.007941 | 0.005812 |
| BP | GO:0014911 | positive regulation of smooth muscle cell migration | 0.000785 | 0.007948 | 0.005816 |
| BP | GO:1902105 | regulation of leukocyte differentiation | 0.000787 | 0.00795 | 0.005818 |
| BP | GO:0048146 | positive regulation of fibroblast proliferation | 0.000797 | 0.008038 | 0.005882 |
| BP | GO:0018158 | protein oxidation | 0.000823 | 0.008198 | 0.006 |
| BP | GO:0031665 | negative regulation of lipopolysaccharide-mediated signaling pathway | 0.000823 | 0.008198 | 0.006 |
| BP | GO:0070278 | extracellular matrix constituent secretion | 0.000823 | 0.008198 | 0.006 |
| BP | GO:0098760 | response to interleukin-7 | 0.000823 | 0.008198 | 0.006 |
| BP | GO:0098761 | cellular response to interleukin-7 | 0.000823 | 0.008198 | 0.006 |
| BP | GO:1902043 | positive regulation of extrinsic apoptotic signaling pathway via death domain receptors | 0.000823 | 0.008198 | 0.006 |
| BP | GO:1903038 | negative regulation of leukocyte cell-cell adhesion | 0.000823 | 0.008198 | 0.006 |
| BP | GO:0050920 | regulation of chemotaxis | 0.00083 | 0.008244 | 0.006034 |
| BP | GO:0071674 | mononuclear cell migration | 0.000847 | 0.008399 | 0.006147 |
| BP | GO:0010714 | positive regulation of collagen metabolic process | 0.000854 | 0.008438 | 0.006176 |
| BP | GO:1903319 | positive regulation of protein maturation | 0.000854 | 0.008438 | 0.006176 |
| BP | GO:0050869 | negative regulation of B cell activation | 0.000858 | 0.008464 | 0.006195 |
| BP | GO:0001818 | negative regulation of cytokine production | 0.000876 | 0.008625 | 0.006312 |
| BP | GO:0034340 | response to type I interferon | 0.000904 | 0.00889 | 0.006506 |
| BP | GO:0046635 | positive regulation of alpha-beta T cell activation | 0.000909 | 0.00892 | 0.006528 |
| BP | GO:0046634 | regulation of alpha-beta T cell activation | 0.000935 | 0.009161 | 0.006704 |
| BP | GO:0016525 | negative regulation of angiogenesis | 0.000971 | 0.009482 | 0.006939 |
| BP | GO:0032946 | positive regulation of mononuclear cell proliferation | 0.000971 | 0.009482 | 0.006939 |
| BP | GO:0051090 | regulation of DNA-binding transcription factor activity | 0.001008 | 0.009819 | 0.007186 |
| BP | GO:0008585 | female gonad development | 0.001013 | 0.009837 | 0.007199 |
| BP | GO:1901655 | cellular response to ketone | 0.001013 | 0.009837 | 0.007199 |
| BP | GO:0018205 | peptidyl-lysine modification | 0.001022 | 0.009898 | 0.007244 |
| BP | GO:0002688 | regulation of leukocyte chemotaxis | 0.001023 | 0.009898 | 0.007244 |
| BP | GO:0035924 | cellular response to vascular endothelial growth factor stimulus | 0.001029 | 0.009937 | 0.007273 |
| BP | GO:0072331 | signal transduction by p53 class mediator | 0.00103 | 0.009937 | 0.007273 |
| BP | GO:0030100 | regulation of endocytosis | 0.001037 | 0.009987 | 0.007309 |
| BP | GO:0034110 | regulation of homotypic cell-cell adhesion | 0.00104 | 0.009992 | 0.007312 |
| BP | GO:0010592 | positive regulation of lamellipodium assembly | 0.00107 | 0.010244 | 0.007497 |
| BP | GO:0072012 | glomerulus vasculature development | 0.00107 | 0.010244 | 0.007497 |
| BP | GO:0031103 | axon regeneration | 0.001077 | 0.010277 | 0.007521 |
| BP | GO:0042743 | hydrogen peroxide metabolic process | 0.001077 | 0.010277 | 0.007521 |
| BP | GO:0046596 | regulation of viral entry into host cell | 0.0011 | 0.010481 | 0.00767 |
| BP | GO:0030218 | erythrocyte differentiation | 0.001117 | 0.010616 | 0.00777 |
| BP | GO:0001841 | neural tube formation | 0.001118 | 0.010616 | 0.00777 |
| BP | GO:0008625 | extrinsic apoptotic signaling pathway via death domain receptors | 0.001135 | 0.010759 | 0.007874 |
| BP | GO:2000181 | negative regulation of blood vessel morphogenesis | 0.001141 | 0.010797 | 0.007902 |
| BP | GO:0001885 | endothelial cell development | 0.001148 | 0.010822 | 0.00792 |
| BP | GO:0031100 | animal organ regeneration | 0.001148 | 0.010822 | 0.00792 |
| BP | GO:0060337 | type I interferon signaling pathway | 0.001162 | 0.010934 | 0.008002 |
| BP | GO:0070374 | positive regulation of ERK1 and ERK2 cascade | 0.001164 | 0.010942 | 0.008008 |
| BP | GO:0001771 | immunological synapse formation | 0.001176 | 0.010975 | 0.008032 |
| BP | GO:0032905 | transforming growth factor beta1 production | 0.001176 | 0.010975 | 0.008032 |
| BP | GO:0072075 | metanephric mesenchyme development | 0.001176 | 0.010975 | 0.008032 |
| BP | GO:0072148 | epithelial cell fate commitment | 0.001176 | 0.010975 | 0.008032 |
| BP | GO:0045807 | positive regulation of endocytosis | 0.001201 | 0.011191 | 0.00819 |
| BP | GO:0007039 | protein catabolic process in the vacuole | 0.001228 | 0.011405 | 0.008347 |
| BP | GO:0030449 | regulation of complement activation | 0.001228 | 0.011405 | 0.008347 |
| BP | GO:0030183 | B cell differentiation | 0.001235 | 0.011426 | 0.008362 |
| BP | GO:1901343 | negative regulation of vasculature development | 0.001235 | 0.011426 | 0.008362 |
| BP | GO:0071346 | cellular response to interferon-gamma | 0.001237 | 0.011426 | 0.008362 |
| BP | GO:0002369 | T cell cytokine production | 0.001251 | 0.011465 | 0.00839 |
| BP | GO:0002724 | regulation of T cell cytokine production | 0.001251 | 0.011465 | 0.00839 |
| BP | GO:0040036 | regulation of fibroblast growth factor receptor signaling pathway | 0.001251 | 0.011465 | 0.00839 |
| BP | GO:0043516 | regulation of DNA damage response, signal transduction by p53 class mediator | 0.001251 | 0.011465 | 0.00839 |
| BP | GO:0048009 | insulin-like growth factor receptor signaling pathway | 0.001251 | 0.011465 | 0.00839 |
| BP | GO:0007519 | skeletal muscle tissue development | 0.001267 | 0.011592 | 0.008484 |
| BP | GO:0060538 | skeletal muscle organ development | 0.001284 | 0.011704 | 0.008566 |
| BP | GO:0030308 | negative regulation of cell growth | 0.001288 | 0.011704 | 0.008566 |
| BP | GO:0002697 | regulation of immune effector process | 0.001288 | 0.011704 | 0.008566 |
| BP | GO:0006986 | response to unfolded protein | 0.001288 | 0.011704 | 0.008566 |
| BP | GO:0061383 | trabecula morphogenesis | 0.001292 | 0.011722 | 0.008579 |
| BP | GO:0045685 | regulation of glial cell differentiation | 0.001308 | 0.01185 | 0.008672 |
| BP | GO:0033157 | regulation of intracellular protein transport | 0.001321 | 0.011898 | 0.008708 |
| BP | GO:0030204 | chondroitin sulfate metabolic process | 0.001327 | 0.011898 | 0.008708 |
| BP | GO:0042744 | hydrogen peroxide catabolic process | 0.001327 | 0.011898 | 0.008708 |
| BP | GO:0050820 | positive regulation of coagulation | 0.001327 | 0.011898 | 0.008708 |
| BP | GO:0071480 | cellular response to gamma radiation | 0.001327 | 0.011898 | 0.008708 |
| BP | GO:1901798 | positive regulation of signal transduction by p53 class mediator | 0.001327 | 0.011898 | 0.008708 |
| BP | GO:0002690 | positive regulation of leukocyte chemotaxis | 0.001331 | 0.011915 | 0.00872 |
| BP | GO:0002064 | epithelial cell development | 0.001349 | 0.01206 | 0.008826 |
| BP | GO:1902106 | negative regulation of leukocyte differentiation | 0.001357 | 0.012109 | 0.008862 |
| BP | GO:0014910 | regulation of smooth muscle cell migration | 0.001414 | 0.012597 | 0.009219 |
| BP | GO:0010332 | response to gamma radiation | 0.001432 | 0.012722 | 0.009311 |
| BP | GO:0043388 | positive regulation of DNA binding | 0.001432 | 0.012722 | 0.009311 |
| BP | GO:0045637 | regulation of myeloid cell differentiation | 0.001438 | 0.012756 | 0.009336 |
| BP | GO:0061564 | axon development | 0.001456 | 0.012891 | 0.009434 |
| BP | GO:0071357 | cellular response to type I interferon | 0.00147 | 0.012994 | 0.00951 |
| BP | GO:0046545 | development of primary female sexual characteristics | 0.001491 | 0.013115 | 0.009598 |
| BP | GO:0032373 | positive regulation of sterol transport | 0.001496 | 0.013115 | 0.009598 |
| BP | GO:0032376 | positive regulation of cholesterol transport | 0.001496 | 0.013115 | 0.009598 |
| BP | GO:0045730 | respiratory burst | 0.001496 | 0.013115 | 0.009598 |
| BP | GO:0097242 | amyloid-beta clearance | 0.001496 | 0.013115 | 0.009598 |
| BP | GO:0010212 | response to ionizing radiation | 0.001514 | 0.013237 | 0.009687 |
| BP | GO:0033135 | regulation of peptidyl-serine phosphorylation | 0.001514 | 0.013237 | 0.009687 |
| BP | GO:1903828 | negative regulation of protein localization | 0.001562 | 0.013633 | 0.009977 |
| BP | GO:1903829 | positive regulation of protein localization | 0.001585 | 0.013813 | 0.010109 |
| BP | GO:0030301 | cholesterol transport | 0.001617 | 0.014023 | 0.010262 |
| BP | GO:0001916 | positive regulation of T cell mediated cytotoxicity | 0.001629 | 0.014023 | 0.010262 |
| BP | GO:0006620 | post-translational protein targeting to endoplasmic reticulum membrane | 0.00163 | 0.014023 | 0.010262 |
| BP | GO:0034112 | positive regulation of homotypic cell-cell adhesion | 0.00163 | 0.014023 | 0.010262 |
| BP | GO:0034375 | high-density lipoprotein particle remodeling | 0.00163 | 0.014023 | 0.010262 |
| BP | GO:0048268 | clathrin coat assembly | 0.00163 | 0.014023 | 0.010262 |
| BP | GO:1901201 | regulation of extracellular matrix assembly | 0.00163 | 0.014023 | 0.010262 |
| BP | GO:1905906 | regulation of amyloid fibril formation | 0.00163 | 0.014023 | 0.010262 |
| BP | GO:0030177 | positive regulation of Wnt signaling pathway | 0.001639 | 0.01407 | 0.010297 |
| BP | GO:0048008 | platelet-derived growth factor receptor signaling pathway | 0.001643 | 0.01407 | 0.010297 |
| BP | GO:0061005 | cell differentiation involved in kidney development | 0.001643 | 0.01407 | 0.010297 |
| BP | GO:0050864 | regulation of B cell activation | 0.001667 | 0.014255 | 0.010432 |
| BP | GO:0043550 | regulation of lipid kinase activity | 0.001679 | 0.014336 | 0.010492 |
| BP | GO:0030168 | platelet activation | 0.00171 | 0.014536 | 0.010638 |
| BP | GO:0035303 | regulation of dephosphorylation | 0.00171 | 0.014536 | 0.010638 |
| BP | GO:0009895 | negative regulation of catabolic process | 0.00171 | 0.014536 | 0.010638 |
| BP | GO:0034644 | cellular response to UV | 0.001747 | 0.014765 | 0.010805 |
| BP | GO:0071229 | cellular response to acid chemical | 0.001747 | 0.014765 | 0.010805 |
| BP | GO:0002291 | T cell activation via T cell receptor contact with antigen bound to MHC molecule on antigen presenting cell | 0.001756 | 0.014765 | 0.010805 |
| BP | GO:0018401 | peptidyl-proline hydroxylation to 4-hydroxy-L-proline | 0.001756 | 0.014765 | 0.010805 |
| BP | GO:0034356 | NAD biosynthesis via nicotinamide riboside salvage pathway | 0.001756 | 0.014765 | 0.010805 |
| BP | GO:0072203 | cell proliferation involved in metanephros development | 0.001756 | 0.014765 | 0.010805 |
| BP | GO:1901203 | positive regulation of extracellular matrix assembly | 0.001756 | 0.014765 | 0.010805 |
| BP | GO:0010762 | regulation of fibroblast migration | 0.001776 | 0.014845 | 0.010864 |
| BP | GO:0043001 | Golgi to plasma membrane protein transport | 0.001776 | 0.014845 | 0.010864 |
| BP | GO:0048246 | macrophage chemotaxis | 0.001776 | 0.014845 | 0.010864 |
| BP | GO:0140467 | integrated stress response signaling | 0.001776 | 0.014845 | 0.010864 |
| BP | GO:0060249 | anatomical structure homeostasis | 0.001796 | 0.01499 | 0.01097 |
| BP | GO:0002720 | positive regulation of cytokine production involved in immune response | 0.001843 | 0.015333 | 0.011221 |
| BP | GO:0043299 | leukocyte degranulation | 0.001843 | 0.015333 | 0.011221 |
| BP | GO:0007566 | embryo implantation | 0.001877 | 0.015597 | 0.011414 |
| BP | GO:2000401 | regulation of lymphocyte migration | 0.001895 | 0.01572 | 0.011505 |
| BP | GO:0051960 | regulation of nervous system development | 0.00195 | 0.016154 | 0.011822 |
| BP | GO:2001243 | negative regulation of intrinsic apoptotic signaling pathway | 0.001971 | 0.016304 | 0.011932 |
| BP | GO:0061099 | negative regulation of protein tyrosine kinase activity | 0.001983 | 0.016373 | 0.011982 |
| BP | GO:0072202 | cell differentiation involved in metanephros development | 0.002006 | 0.016543 | 0.012107 |
| BP | GO:1902041 | regulation of extrinsic apoptotic signaling pathway via death domain receptors | 0.002034 | 0.016749 | 0.012257 |
| BP | GO:0071230 | cellular response to amino acid stimulus | 0.002057 | 0.016907 | 0.012373 |
| BP | GO:0046660 | female sex differentiation | 0.00209 | 0.01712 | 0.012529 |
| BP | GO:0001936 | regulation of endothelial cell proliferation | 0.00209 | 0.01712 | 0.012529 |
| BP | GO:0003007 | heart morphogenesis | 0.002095 | 0.01712 | 0.012529 |
| BP | GO:0072210 | metanephric nephron development | 0.002097 | 0.01712 | 0.012529 |
| BP | GO:0035051 | cardiocyte differentiation | 0.002098 | 0.01712 | 0.012529 |
| BP | GO:1901216 | positive regulation of neuron death | 0.002142 | 0.017426 | 0.012753 |
| BP | GO:0008406 | gonad development | 0.002144 | 0.017426 | 0.012753 |
| BP | GO:0010633 | negative regulation of epithelial cell migration | 0.002145 | 0.017426 | 0.012753 |
| BP | GO:0042113 | B cell activation | 0.002149 | 0.017432 | 0.012758 |
| BP | GO:0050767 | regulation of neurogenesis | 0.002172 | 0.017591 | 0.012874 |
| BP | GO:0010224 | response to UV-B | 0.0022 | 0.017661 | 0.012925 |
| BP | GO:0038065 | collagen-activated signaling pathway | 0.0022 | 0.017661 | 0.012925 |
| BP | GO:0060192 | negative regulation of lipase activity | 0.0022 | 0.017661 | 0.012925 |
| BP | GO:0070593 | dendrite self-avoidance | 0.0022 | 0.017661 | 0.012925 |
| BP | GO:0072224 | metanephric glomerulus development | 0.0022 | 0.017661 | 0.012925 |
| BP | GO:0090594 | inflammatory response to wounding | 0.0022 | 0.017661 | 0.012925 |
| BP | GO:0050808 | synapse organization | 0.002243 | 0.017982 | 0.01316 |
| BP | GO:0032272 | negative regulation of protein polymerization | 0.00229 | 0.018335 | 0.013418 |
| BP | GO:0002526 | acute inflammatory response | 0.002341 | 0.018691 | 0.013679 |
| BP | GO:1903707 | negative regulation of hemopoiesis | 0.002341 | 0.018691 | 0.013679 |
| BP | GO:0030850 | prostate gland development | 0.002346 | 0.0187 | 0.013686 |
| BP | GO:1904019 | epithelial cell apoptotic process | 0.002356 | 0.018752 | 0.013724 |
| BP | GO:0001843 | neural tube closure | 0.002365 | 0.0188 | 0.013759 |
| BP | GO:2000045 | regulation of G1/S transition of mitotic cell cycle | 0.002387 | 0.018932 | 0.013855 |
| BP | GO:0071353 | cellular response to interleukin-4 | 0.002393 | 0.018932 | 0.013855 |
| BP | GO:0032922 | circadian regulation of gene expression | 0.002396 | 0.018932 | 0.013855 |
| BP | GO:0070527 | platelet aggregation | 0.002396 | 0.018932 | 0.013855 |
| BP | GO:1902893 | regulation of miRNA transcription | 0.002428 | 0.019161 | 0.014023 |
| BP | GO:0071604 | transforming growth factor beta production | 0.002461 | 0.019368 | 0.014175 |
| BP | GO:2000249 | regulation of actin cytoskeleton reorganization | 0.002461 | 0.019368 | 0.014175 |
| BP | GO:0002819 | regulation of adaptive immune response | 0.002496 | 0.019613 | 0.014353 |
| BP | GO:0019835 | cytolysis | 0.002513 | 0.019659 | 0.014387 |
| BP | GO:0043567 | regulation of insulin-like growth factor receptor signaling pathway | 0.002513 | 0.019659 | 0.014387 |
| BP | GO:1903077 | negative regulation of protein localization to plasma membrane | 0.002513 | 0.019659 | 0.014387 |
| BP | GO:0046209 | nitric oxide metabolic process | 0.002545 | 0.019827 | 0.01451 |
| BP | GO:0048708 | astrocyte differentiation | 0.002545 | 0.019827 | 0.01451 |
| BP | GO:0051149 | positive regulation of muscle cell differentiation | 0.002545 | 0.019827 | 0.01451 |
| BP | GO:0009266 | response to temperature stimulus | 0.002549 | 0.019827 | 0.014511 |
| BP | GO:0010950 | positive regulation of endopeptidase activity | 0.002584 | 0.020052 | 0.014675 |
| BP | GO:0045926 | negative regulation of growth | 0.002585 | 0.020052 | 0.014675 |
| BP | GO:0043535 | regulation of blood vessel endothelial cell migration | 0.002589 | 0.020054 | 0.014676 |
| BP | GO:0006892 | post-Golgi vesicle-mediated transport | 0.002604 | 0.020122 | 0.014726 |
| BP | GO:0060606 | tube closure | 0.002608 | 0.020122 | 0.014726 |
| BP | GO:0007548 | sex differentiation | 0.00261 | 0.020122 | 0.014726 |
| BP | GO:0001894 | tissue homeostasis | 0.002613 | 0.020122 | 0.014726 |
| BP | GO:0002768 | immune response-regulating cell surface receptor signaling pathway | 0.00263 | 0.020122 | 0.014726 |
| BP | GO:0019065 | receptor-mediated endocytosis of virus by host cell | 0.002634 | 0.020122 | 0.014726 |
| BP | GO:0032060 | bleb assembly | 0.002634 | 0.020122 | 0.014726 |
| BP | GO:0032836 | glomerular basement membrane development | 0.002634 | 0.020122 | 0.014726 |
| BP | GO:0048505 | regulation of timing of cell differentiation | 0.002634 | 0.020122 | 0.014726 |
| BP | GO:1990440 | positive regulation of transcription from RNA polymerase II promoter in response to endoplasmic reticulum stress | 0.002634 | 0.020122 | 0.014726 |
| BP | GO:0120032 | regulation of plasma membrane bounded cell projection assembly | 0.002657 | 0.020275 | 0.014838 |
| BP | GO:0001838 | embryonic epithelial tube formation | 0.002674 | 0.020372 | 0.014909 |
| BP | GO:0010939 | regulation of necrotic cell death | 0.002694 | 0.0205 | 0.015003 |
| BP | GO:0002700 | regulation of production of molecular mediator of immune response | 0.002719 | 0.020658 | 0.015119 |
| BP | GO:0000381 | regulation of alternative mRNA splicing, via spliceosome | 0.002748 | 0.020795 | 0.015219 |
| BP | GO:0043030 | regulation of macrophage activation | 0.002748 | 0.020795 | 0.015219 |
| BP | GO:0061951 | establishment of protein localization to plasma membrane | 0.002748 | 0.020795 | 0.015219 |
| BP | GO:0035966 | response to topologically incorrect protein | 0.002787 | 0.021062 | 0.015414 |
| BP | GO:2001057 | reactive nitrogen species metabolic process | 0.002822 | 0.021296 | 0.015585 |
| BP | GO:0045137 | development of primary sexual characteristics | 0.002855 | 0.021512 | 0.015743 |
| BP | GO:0045687 | positive regulation of glial cell differentiation | 0.002874 | 0.021625 | 0.015826 |
| BP | GO:0009314 | response to radiation | 0.002888 | 0.021634 | 0.015832 |
| BP | GO:0050868 | negative regulation of T cell activation | 0.002896 | 0.021634 | 0.015832 |
| BP | GO:0061138 | morphogenesis of a branching epithelium | 0.002899 | 0.021634 | 0.015832 |
| BP | GO:0003159 | morphogenesis of an endothelium | 0.002902 | 0.021634 | 0.015832 |
| BP | GO:0030206 | chondroitin sulfate biosynthetic process | 0.002902 | 0.021634 | 0.015832 |
| BP | GO:0061154 | endothelial tube morphogenesis | 0.002902 | 0.021634 | 0.015832 |
| BP | GO:0097067 | cellular response to thyroid hormone stimulus | 0.002902 | 0.021634 | 0.015832 |
| BP | GO:0042246 | tissue regeneration | 0.002998 | 0.022288 | 0.016311 |
| BP | GO:0072678 | T cell migration | 0.002998 | 0.022288 | 0.016311 |
| BP | GO:0060491 | regulation of cell projection assembly | 0.003007 | 0.022323 | 0.016337 |
| BP | GO:0071887 | leukocyte apoptotic process | 0.003022 | 0.022402 | 0.016395 |
| BP | GO:0006890 | retrograde vesicle-mediated transport, Golgi to endoplasmic reticulum | 0.003083 | 0.022684 | 0.016601 |
| BP | GO:0034381 | plasma lipoprotein particle clearance | 0.003083 | 0.022684 | 0.016601 |
| BP | GO:0048260 | positive regulation of receptor-mediated endocytosis | 0.003083 | 0.022684 | 0.016601 |
| BP | GO:0032481 | positive regulation of type I interferon production | 0.003101 | 0.022684 | 0.016601 |
| BP | GO:0002726 | positive regulation of T cell cytokine production | 0.003109 | 0.022684 | 0.016601 |
| BP | GO:0006929 | substrate-dependent cell migration | 0.003109 | 0.022684 | 0.016601 |
| BP | GO:0010954 | positive regulation of protein processing | 0.003109 | 0.022684 | 0.016601 |
| BP | GO:0035994 | response to muscle stretch | 0.003109 | 0.022684 | 0.016601 |
| BP | GO:0046629 | gamma-delta T cell activation | 0.003109 | 0.022684 | 0.016601 |
| BP | GO:0060333 | interferon-gamma-mediated signaling pathway | 0.003109 | 0.022684 | 0.016601 |
| BP | GO:1905048 | regulation of metallopeptidase activity | 0.003109 | 0.022684 | 0.016601 |
| BP | GO:1905523 | positive regulation of macrophage migration | 0.003109 | 0.022684 | 0.016601 |
| BP | GO:0045931 | positive regulation of mitotic cell cycle | 0.003134 | 0.022834 | 0.016711 |
| BP | GO:0010001 | glial cell differentiation | 0.00315 | 0.022917 | 0.016772 |
| BP | GO:0032088 | negative regulation of NF-kappaB transcription factor activity | 0.003153 | 0.022917 | 0.016772 |
| BP | GO:0051056 | regulation of small GTPase mediated signal transduction | 0.003159 | 0.022929 | 0.01678 |
| BP | GO:0016331 | morphogenesis of embryonic epithelium | 0.003213 | 0.023282 | 0.017039 |
| BP | GO:0140694 | non-membrane-bounded organelle assembly | 0.003216 | 0.023282 | 0.017039 |
| BP | GO:0032612 | interleukin-1 production | 0.003387 | 0.024453 | 0.017896 |
| BP | GO:0032652 | regulation of interleukin-1 production | 0.003387 | 0.024453 | 0.017896 |
| BP | GO:0001763 | morphogenesis of a branching structure | 0.003394 | 0.024465 | 0.017905 |
| BP | GO:0002719 | negative regulation of cytokine production involved in immune response | 0.003406 | 0.024465 | 0.017905 |
| BP | GO:0050654 | chondroitin sulfate proteoglycan metabolic process | 0.003406 | 0.024465 | 0.017905 |
| BP | GO:2000406 | positive regulation of T cell migration | 0.003406 | 0.024465 | 0.017905 |
| BP | GO:0051384 | response to glucocorticoid | 0.003439 | 0.024669 | 0.018054 |
| BP | GO:0019216 | regulation of lipid metabolic process | 0.003455 | 0.02475 | 0.018113 |
| BP | GO:0000768 | syncytium formation by plasma membrane fusion | 0.00349 | 0.024901 | 0.018224 |
| BP | GO:0071384 | cellular response to corticosteroid stimulus | 0.00349 | 0.024901 | 0.018224 |
| BP | GO:0140253 | cell-cell fusion | 0.00349 | 0.024901 | 0.018224 |
| BP | GO:1902743 | regulation of lamellipodium organization | 0.003513 | 0.025038 | 0.018324 |
| BP | GO:0002455 | humoral immune response mediated by circulating immunoglobulin | 0.00356 | 0.025335 | 0.018541 |
| BP | GO:2001056 | positive regulation of cysteine-type endopeptidase activity | 0.003699 | 0.026292 | 0.019242 |
| BP | GO:0043536 | positive regulation of blood vessel endothelial cell migration | 0.003717 | 0.026333 | 0.019272 |
| BP | GO:2000242 | negative regulation of reproductive process | 0.003717 | 0.026333 | 0.019272 |
| BP | GO:0061298 | retina vasculature development in camera-type eye | 0.003754 | 0.026333 | 0.019272 |
| BP | GO:0072074 | kidney mesenchyme development | 0.003754 | 0.026333 | 0.019272 |
| BP | GO:0097284 | hepatocyte apoptotic process | 0.003754 | 0.026333 | 0.019272 |
| BP | GO:0001867 | complement activation, lectin pathway | 0.003771 | 0.026333 | 0.019272 |
| BP | GO:0032908 | regulation of transforming growth factor beta1 production | 0.003771 | 0.026333 | 0.019272 |
| BP | GO:0038063 | collagen-activated tyrosine kinase receptor signaling pathway | 0.003771 | 0.026333 | 0.019272 |
| BP | GO:0040034 | regulation of development, heterochronic | 0.003771 | 0.026333 | 0.019272 |
| BP | GO:0043589 | skin morphogenesis | 0.003771 | 0.026333 | 0.019272 |
| BP | GO:0072182 | regulation of nephron tubule epithelial cell differentiation | 0.003771 | 0.026333 | 0.019272 |
| BP | GO:0097084 | vascular associated smooth muscle cell development | 0.003771 | 0.026333 | 0.019272 |
| BP | GO:1904350 | regulation of protein catabolic process in the vacuole | 0.003771 | 0.026333 | 0.019272 |
| BP | GO:0007260 | tyrosine phosphorylation of STAT protein | 0.003804 | 0.026333 | 0.019272 |
| BP | GO:0032092 | positive regulation of protein binding | 0.003804 | 0.026333 | 0.019272 |
| BP | GO:0030325 | adrenal gland development | 0.003805 | 0.026333 | 0.019272 |
| BP | GO:0036296 | response to increased oxygen levels | 0.003805 | 0.026333 | 0.019272 |
| BP | GO:0044342 | type B pancreatic cell proliferation | 0.003805 | 0.026333 | 0.019272 |
| BP | GO:0071549 | cellular response to dexamethasone stimulus | 0.003805 | 0.026333 | 0.019272 |
| BP | GO:1904376 | negative regulation of protein localization to cell periphery | 0.003805 | 0.026333 | 0.019272 |
| BP | GO:2000737 | negative regulation of stem cell differentiation | 0.003805 | 0.026333 | 0.019272 |
| BP | GO:1903311 | regulation of mRNA metabolic process | 0.003822 | 0.02642 | 0.019335 |
| BP | GO:0090288 | negative regulation of cellular response to growth factor stimulus | 0.003857 | 0.026601 | 0.019467 |
| BP | GO:0062208 | positive regulation of pattern recognition receptor signaling pathway | 0.003858 | 0.026601 | 0.019467 |
| BP | GO:0030217 | T cell differentiation | 0.003876 | 0.02669 | 0.019533 |
| BP | GO:0002573 | myeloid leukocyte differentiation | 0.003894 | 0.026779 | 0.019598 |
| BP | GO:0045444 | fat cell differentiation | 0.003961 | 0.02721 | 0.019913 |
| BP | GO:0071385 | cellular response to glucocorticoid stimulus | 0.00399 | 0.027307 | 0.019984 |
| BP | GO:1900087 | positive regulation of G1/S transition of mitotic cell cycle | 0.00399 | 0.027307 | 0.019984 |
| BP | GO:2000107 | negative regulation of leukocyte apoptotic process | 0.00399 | 0.027307 | 0.019984 |
| BP | GO:0048261 | negative regulation of receptor-mediated endocytosis | 0.004021 | 0.027408 | 0.020058 |
| BP | GO:0060323 | head morphogenesis | 0.004021 | 0.027408 | 0.020058 |
| BP | GO:0070670 | response to interleukin-4 | 0.004021 | 0.027408 | 0.020058 |
| BP | GO:0042116 | macrophage activation | 0.004025 | 0.027408 | 0.020058 |
| BP | GO:0006936 | muscle contraction | 0.00412 | 0.028017 | 0.020504 |
| BP | GO:0006809 | nitric oxide biosynthetic process | 0.004124 | 0.028017 | 0.020504 |
| BP | GO:0014020 | primary neural tube formation | 0.004143 | 0.028109 | 0.020571 |
| BP | GO:0001892 | embryonic placenta development | 0.004187 | 0.028372 | 0.020764 |
| BP | GO:0031345 | negative regulation of cell projection organization | 0.004202 | 0.028438 | 0.020812 |
| BP | GO:0002244 | hematopoietic progenitor cell differentiation | 0.00425 | 0.028727 | 0.021024 |
| BP | GO:0010517 | regulation of phospholipase activity | 0.004381 | 0.029544 | 0.021621 |
| BP | GO:0030837 | negative regulation of actin filament polymerization | 0.004381 | 0.029544 | 0.021621 |
| BP | GO:0097178 | ruffle assembly | 0.004439 | 0.029894 | 0.021877 |
| BP | GO:0071901 | negative regulation of protein serine/threonine kinase activity | 0.004511 | 0.03034 | 0.022204 |
| BP | GO:0045806 | negative regulation of endocytosis | 0.004516 | 0.03034 | 0.022204 |
| BP | GO:0030048 | actin filament-based movement | 0.004574 | 0.030674 | 0.022449 |
| BP | GO:0031333 | negative regulation of protein-containing complex assembly | 0.004577 | 0.030674 | 0.022449 |
| BP | GO:1901879 | regulation of protein depolymerization | 0.0046 | 0.030709 | 0.022474 |
| BP | GO:0030194 | positive regulation of blood coagulation | 0.00461 | 0.030709 | 0.022474 |
| BP | GO:0031664 | regulation of lipopolysaccharide-mediated signaling pathway | 0.00461 | 0.030709 | 0.022474 |
| BP | GO:0060765 | regulation of androgen receptor signaling pathway | 0.00461 | 0.030709 | 0.022474 |
| BP | GO:1900048 | positive regulation of hemostasis | 0.00461 | 0.030709 | 0.022474 |
| BP | GO:0031330 | negative regulation of cellular catabolic process | 0.004637 | 0.030855 | 0.022581 |
| BP | GO:0010923 | negative regulation of phosphatase activity | 0.004717 | 0.031237 | 0.02286 |
| BP | GO:0044319 | wound healing, spreading of cells | 0.004717 | 0.031237 | 0.02286 |
| BP | GO:0090505 | epiboly involved in wound healing | 0.004717 | 0.031237 | 0.02286 |
| BP | GO:1902745 | positive regulation of lamellipodium organization | 0.004717 | 0.031237 | 0.02286 |
| BP | GO:0051051 | negative regulation of transport | 0.00473 | 0.031282 | 0.022893 |
| BP | GO:0002396 | MHC protein complex assembly | 0.00477 | 0.031397 | 0.022978 |
| BP | GO:0002501 | peptide antigen assembly with MHC protein complex | 0.00477 | 0.031397 | 0.022978 |
| BP | GO:0002716 | negative regulation of natural killer cell mediated immunity | 0.00477 | 0.031397 | 0.022978 |
| BP | GO:0032495 | response to muramyl dipeptide | 0.00477 | 0.031397 | 0.022978 |
| BP | GO:0045732 | positive regulation of protein catabolic process | 0.004815 | 0.031656 | 0.023167 |
| BP | GO:0002699 | positive regulation of immune effector process | 0.004858 | 0.031861 | 0.023317 |
| BP | GO:0006470 | protein dephosphorylation | 0.004858 | 0.031861 | 0.023317 |
| BP | GO:0030203 | glycosaminoglycan metabolic process | 0.004871 | 0.031912 | 0.023354 |
| BP | GO:0032640 | tumor necrosis factor production | 0.0049 | 0.031989 | 0.023411 |
| BP | GO:0032680 | regulation of tumor necrosis factor production | 0.0049 | 0.031989 | 0.023411 |
| BP | GO:0050728 | negative regulation of inflammatory response | 0.0049 | 0.031989 | 0.023411 |
| BP | GO:1900180 | regulation of protein localization to nucleus | 0.004919 | 0.032073 | 0.023472 |
| BP | GO:0071496 | cellular response to external stimulus | 0.004926 | 0.032078 | 0.023476 |
| BP | GO:0002709 | regulation of T cell mediated immunity | 0.005044 | 0.032745 | 0.023964 |
| BP | GO:0061045 | negative regulation of wound healing | 0.005046 | 0.032745 | 0.023964 |
| BP | GO:0070373 | negative regulation of ERK1 and ERK2 cascade | 0.005046 | 0.032745 | 0.023964 |
| BP | GO:0030521 | androgen receptor signaling pathway | 0.005084 | 0.032837 | 0.024032 |
| BP | GO:0046677 | response to antibiotic | 0.005084 | 0.032837 | 0.024032 |
| BP | GO:0097028 | dendritic cell differentiation | 0.005084 | 0.032837 | 0.024032 |
| BP | GO:1902895 | positive regulation of miRNA transcription | 0.005084 | 0.032837 | 0.024032 |
| BP | GO:0008347 | glial cell migration | 0.005094 | 0.032864 | 0.024051 |
| BP | GO:0045445 | myoblast differentiation | 0.005143 | 0.033049 | 0.024187 |
| BP | GO:0051147 | regulation of muscle cell differentiation | 0.005169 | 0.033049 | 0.024187 |
| BP | GO:0043568 | positive regulation of insulin-like growth factor receptor signaling pathway | 0.005201 | 0.033049 | 0.024187 |
| BP | GO:0045916 | negative regulation of complement activation | 0.005201 | 0.033049 | 0.024187 |
| BP | GO:0070424 | regulation of nucleotide-binding oligomerization domain containing signaling pathway | 0.005201 | 0.033049 | 0.024187 |
| BP | GO:0075509 | endocytosis involved in viral entry into host cell | 0.005201 | 0.033049 | 0.024187 |
| BP | GO:0097048 | dendritic cell apoptotic process | 0.005201 | 0.033049 | 0.024187 |
| BP | GO:1900115 | extracellular regulation of signal transduction | 0.005201 | 0.033049 | 0.024187 |
| BP | GO:1900116 | extracellular negative regulation of signal transduction | 0.005201 | 0.033049 | 0.024187 |
| BP | GO:1903265 | positive regulation of tumor necrosis factor-mediated signaling pathway | 0.005201 | 0.033049 | 0.024187 |
| BP | GO:1905907 | negative regulation of amyloid fibril formation | 0.005201 | 0.033049 | 0.024187 |
| BP | GO:2000668 | regulation of dendritic cell apoptotic process | 0.005201 | 0.033049 | 0.024187 |
| BP | GO:2001198 | regulation of dendritic cell differentiation | 0.005201 | 0.033049 | 0.024187 |
| BP | GO:0055007 | cardiac muscle cell differentiation | 0.005254 | 0.03335 | 0.024407 |
| BP | GO:0000082 | G1/S transition of mitotic cell cycle | 0.005407 | 0.034282 | 0.025089 |
| BP | GO:0006949 | syncytium formation | 0.005443 | 0.034472 | 0.025228 |
| BP | GO:0090504 | epiboly | 0.0055 | 0.034723 | 0.025411 |
| BP | GO:0010586 | miRNA metabolic process | 0.005522 | 0.034723 | 0.025411 |
| BP | GO:0055006 | cardiac cell development | 0.005522 | 0.034723 | 0.025411 |
| BP | GO:0043405 | regulation of MAP kinase activity | 0.005525 | 0.034723 | 0.025411 |
| BP | GO:0006622 | protein targeting to lysosome | 0.005533 | 0.034723 | 0.025411 |
| BP | GO:0010575 | positive regulation of vascular endothelial growth factor production | 0.005533 | 0.034723 | 0.025411 |
| BP | GO:0010804 | negative regulation of tumor necrosis factor-mediated signaling pathway | 0.005533 | 0.034723 | 0.025411 |
| BP | GO:0097066 | response to thyroid hormone | 0.005533 | 0.034723 | 0.025411 |
| BP | GO:0032388 | positive regulation of intracellular transport | 0.005629 | 0.035286 | 0.025823 |
| BP | GO:0033619 | membrane protein proteolysis | 0.005728 | 0.035863 | 0.026246 |
| BP | GO:0010171 | body morphogenesis | 0.005799 | 0.036142 | 0.02645 |
| BP | GO:0035305 | negative regulation of dephosphorylation | 0.005799 | 0.036142 | 0.02645 |
| BP | GO:0035850 | epithelial cell differentiation involved in kidney development | 0.005799 | 0.036142 | 0.02645 |
| BP | GO:0048483 | autonomic nervous system development | 0.005799 | 0.036142 | 0.02645 |
| BP | GO:0043393 | regulation of protein binding | 0.005958 | 0.037093 | 0.027146 |
| BP | GO:1903206 | negative regulation of hydrogen peroxide-induced cell death | 0.005967 | 0.037108 | 0.027157 |
| BP | GO:0001890 | placenta development | 0.006006 | 0.037262 | 0.02727 |
| BP | GO:0098876 | vesicle-mediated transport to the plasma membrane | 0.006006 | 0.037262 | 0.02727 |
| BP | GO:0046777 | protein autophosphorylation | 0.00603 | 0.037375 | 0.027353 |
| BP | GO:0003206 | cardiac chamber morphogenesis | 0.006092 | 0.037674 | 0.027572 |
| BP | GO:0043244 | regulation of protein-containing complex disassembly | 0.006092 | 0.037674 | 0.027572 |
| BP | GO:0014068 | positive regulation of phosphatidylinositol 3-kinase signaling | 0.006123 | 0.037823 | 0.02768 |
| BP | GO:0034504 | protein localization to nucleus | 0.006154 | 0.037972 | 0.027789 |
| BP | GO:0030279 | negative regulation of ossification | 0.006376 | 0.039207 | 0.028693 |
| BP | GO:0033028 | myeloid cell apoptotic process | 0.006376 | 0.039207 | 0.028693 |
| BP | GO:0071634 | regulation of transforming growth factor beta production | 0.006376 | 0.039207 | 0.028693 |
| BP | GO:0045010 | actin nucleation | 0.006421 | 0.039441 | 0.028865 |
| BP | GO:0035710 | CD4-positive, alpha-beta T cell activation | 0.006499 | 0.039875 | 0.029182 |
| BP | GO:0008203 | cholesterol metabolic process | 0.006515 | 0.039887 | 0.029191 |
| BP | GO:0072175 | epithelial tube formation | 0.006515 | 0.039887 | 0.029191 |
| BP | GO:0071706 | tumor necrosis factor superfamily cytokine production | 0.006584 | 0.03993 | 0.029223 |
| BP | GO:1903555 | regulation of tumor necrosis factor superfamily cytokine production | 0.006584 | 0.03993 | 0.029223 |
| BP | GO:0034067 | protein localization to Golgi apparatus | 0.006585 | 0.03993 | 0.029223 |
| BP | GO:0045940 | positive regulation of steroid metabolic process | 0.006585 | 0.03993 | 0.029223 |
| BP | GO:0060325 | face morphogenesis | 0.006585 | 0.03993 | 0.029223 |
| BP | GO:0070293 | renal absorption | 0.006585 | 0.03993 | 0.029223 |
| BP | GO:1903205 | regulation of hydrogen peroxide-induced cell death | 0.006585 | 0.03993 | 0.029223 |
| BP | GO:0072091 | regulation of stem cell proliferation | 0.006586 | 0.03993 | 0.029223 |
| BP | GO:2000404 | regulation of T cell migration | 0.006587 | 0.03993 | 0.029223 |
| BP | GO:0021915 | neural tube development | 0.006667 | 0.040368 | 0.029543 |
| BP | GO:0051881 | regulation of mitochondrial membrane potential | 0.006696 | 0.040456 | 0.029607 |
| BP | GO:0016064 | immunoglobulin mediated immune response | 0.006697 | 0.040456 | 0.029607 |
| BP | GO:0000380 | alternative mRNA splicing, via spliceosome | 0.006726 | 0.040456 | 0.029607 |
| BP | GO:0001570 | vasculogenesis | 0.006726 | 0.040456 | 0.029607 |
| BP | GO:0031060 | regulation of histone methylation | 0.006726 | 0.040456 | 0.029607 |
| BP | GO:0072332 | intrinsic apoptotic signaling pathway by p53 class mediator | 0.006726 | 0.040456 | 0.029607 |
| BP | GO:0035791 | platelet-derived growth factor receptor-beta signaling pathway | 0.006952 | 0.041321 | 0.03024 |
| BP | GO:0043374 | CD8-positive, alpha-beta T cell differentiation | 0.006952 | 0.041321 | 0.03024 |
| BP | GO:0043558 | regulation of translational initiation in response to stress | 0.006952 | 0.041321 | 0.03024 |
| BP | GO:0043922 | negative regulation by host of viral transcription | 0.006952 | 0.041321 | 0.03024 |
| BP | GO:0045059 | positive thymic T cell selection | 0.006952 | 0.041321 | 0.03024 |
| BP | GO:0048711 | positive regulation of astrocyte differentiation | 0.006952 | 0.041321 | 0.03024 |
| BP | GO:0048715 | negative regulation of oligodendrocyte differentiation | 0.006952 | 0.041321 | 0.03024 |
| BP | GO:0051764 | actin crosslink formation | 0.006952 | 0.041321 | 0.03024 |
| BP | GO:0071801 | regulation of podosome assembly | 0.006952 | 0.041321 | 0.03024 |
| BP | GO:0072160 | nephron tubule epithelial cell differentiation | 0.006952 | 0.041321 | 0.03024 |
| BP | GO:1905049 | negative regulation of metallopeptidase activity | 0.006952 | 0.041321 | 0.03024 |
| BP | GO:0002705 | positive regulation of leukocyte mediated immunity | 0.006973 | 0.041405 | 0.030302 |
| BP | GO:0030518 | intracellular steroid hormone receptor signaling pathway | 0.007009 | 0.041574 | 0.030426 |
| BP | GO:2000106 | regulation of leukocyte apoptotic process | 0.007176 | 0.042429 | 0.031052 |
| BP | GO:0006893 | Golgi to plasma membrane transport | 0.007177 | 0.042429 | 0.031052 |
| BP | GO:0045599 | negative regulation of fat cell differentiation | 0.007177 | 0.042429 | 0.031052 |
| BP | GO:0048568 | embryonic organ development | 0.007358 | 0.043333 | 0.031713 |
| BP | GO:0032516 | positive regulation of phosphoprotein phosphatase activity | 0.007361 | 0.043333 | 0.031713 |
| BP | GO:0071404 | cellular response to low-density lipoprotein particle stimulus | 0.007361 | 0.043333 | 0.031713 |
| BP | GO:0072111 | cell proliferation involved in kidney development | 0.007361 | 0.043333 | 0.031713 |
| BP | GO:0042509 | regulation of tyrosine phosphorylation of STAT protein | 0.007373 | 0.043336 | 0.031715 |
| BP | GO:0008217 | regulation of blood pressure | 0.007377 | 0.043336 | 0.031715 |
| BP | GO:0033344 | cholesterol efflux | 0.007401 | 0.043386 | 0.031752 |
| BP | GO:2000379 | positive regulation of reactive oxygen species metabolic process | 0.007401 | 0.043386 | 0.031752 |
| BP | GO:0032479 | regulation of type I interferon production | 0.007438 | 0.043509 | 0.031842 |
| BP | GO:0032606 | type I interferon production | 0.007438 | 0.043509 | 0.031842 |
| BP | GO:0007520 | myoblast fusion | 0.007455 | 0.043559 | 0.031879 |
| BP | GO:0002263 | cell activation involved in immune response | 0.00752 | 0.043895 | 0.032124 |
| BP | GO:0010039 | response to iron ion | 0.007774 | 0.045155 | 0.033046 |
| BP | GO:0033688 | regulation of osteoblast proliferation | 0.007774 | 0.045155 | 0.033046 |
| BP | GO:0060390 | regulation of SMAD protein signal transduction | 0.007774 | 0.045155 | 0.033046 |
| BP | GO:1900027 | regulation of ruffle assembly | 0.007774 | 0.045155 | 0.033046 |
| BP | GO:0016125 | sterol metabolic process | 0.007777 | 0.045155 | 0.033046 |
| BP | GO:0050769 | positive regulation of neurogenesis | 0.007802 | 0.045229 | 0.033101 |
| BP | GO:1903035 | negative regulation of response to wounding | 0.007806 | 0.045229 | 0.033101 |
| BP | GO:0019724 | B cell mediated immunity | 0.007844 | 0.045353 | 0.033191 |
| BP | GO:1901215 | negative regulation of neuron death | 0.007844 | 0.045353 | 0.033191 |
| BP | GO:0032496 | response to lipopolysaccharide | 0.007857 | 0.045381 | 0.033211 |
| BP | GO:0016050 | vesicle organization | 0.007922 | 0.045707 | 0.033451 |
| BP | GO:0010977 | negative regulation of neuron projection development | 0.007967 | 0.045923 | 0.033608 |
| BP | GO:0009100 | glycoprotein metabolic process | 0.008001 | 0.046056 | 0.033706 |
| BP | GO:0003205 | cardiac chamber development | 0.008007 | 0.046056 | 0.033706 |
| BP | GO:0006958 | complement activation, classical pathway | 0.008043 | 0.046213 | 0.033821 |
| BP | GO:0055013 | cardiac muscle cell development | 0.008069 | 0.046312 | 0.033893 |
| BP | GO:0034968 | histone lysine methylation | 0.008091 | 0.046395 | 0.033954 |
| BP | GO:0042698 | ovulation cycle | 0.008162 | 0.046657 | 0.034146 |
| BP | GO:0070265 | necrotic cell death | 0.008162 | 0.046657 | 0.034146 |
| BP | GO:1901880 | negative regulation of protein depolymerization | 0.008162 | 0.046657 | 0.034146 |
| BP | GO:0007409 | axonogenesis | 0.008227 | 0.046978 | 0.034381 |
| BP | GO:0035148 | tube formation | 0.00828 | 0.047229 | 0.034564 |
| BP | GO:0031062 | positive regulation of histone methylation | 0.008405 | 0.047661 | 0.034881 |
| BP | GO:0032692 | negative regulation of interleukin-1 production | 0.008405 | 0.047661 | 0.034881 |
| BP | GO:0032873 | negative regulation of stress-activated MAPK cascade | 0.008405 | 0.047661 | 0.034881 |
| BP | GO:0034332 | adherens junction organization | 0.008405 | 0.047661 | 0.034881 |
| BP | GO:0070303 | negative regulation of stress-activated protein kinase signaling cascade | 0.008405 | 0.047661 | 0.034881 |
| BP | GO:0001914 | regulation of T cell mediated cytotoxicity | 0.008433 | 0.047661 | 0.034881 |
| BP | GO:0006458 | 'de novo' protein folding | 0.008433 | 0.047661 | 0.034881 |
| BP | GO:0072595 | maintenance of protein localization in organelle | 0.008433 | 0.047661 | 0.034881 |
| BP | GO:1901031 | regulation of response to reactive oxygen species | 0.008433 | 0.047661 | 0.034881 |
| BP | GO:1903510 | mucopolysaccharide metabolic process | 0.008479 | 0.04787 | 0.035033 |
| BP | GO:0055123 | digestive system development | 0.008505 | 0.04797 | 0.035106 |
| BP | GO:0098727 | maintenance of cell number | 0.00864 | 0.048678 | 0.035625 |
| BP | GO:0014013 | regulation of gliogenesis | 0.008686 | 0.048888 | 0.035778 |
| BP | GO:0002708 | positive regulation of lymphocyte mediated immunity | 0.008735 | 0.049016 | 0.035872 |
| BP | GO:0032609 | interferon-gamma production | 0.008735 | 0.049016 | 0.035872 |
| BP | GO:0032649 | regulation of interferon-gamma production | 0.008735 | 0.049016 | 0.035872 |
| BP | GO:0008589 | regulation of smoothened signaling pathway | 0.008814 | 0.049357 | 0.036121 |
| BP | GO:0097006 | regulation of plasma lipoprotein particle levels | 0.008814 | 0.049357 | 0.036121 |
| BP | GO:0030166 | proteoglycan biosynthetic process | 0.00889 | 0.049684 | 0.036361 |
| BP | GO:0045428 | regulation of nitric oxide biosynthetic process | 0.00889 | 0.049684 | 0.036361 |
| BP | GO:0035739 | CD4-positive, alpha-beta T cell proliferation | 0.008967 | 0.049732 | 0.036396 |
| BP | GO:0046794 | transport of virus | 0.008967 | 0.049732 | 0.036396 |
| BP | GO:0050650 | chondroitin sulfate proteoglycan biosynthetic process | 0.008967 | 0.049732 | 0.036396 |
| BP | GO:0072243 | metanephric nephron epithelium development | 0.008967 | 0.049732 | 0.036396 |
| BP | GO:1901032 | negative regulation of response to reactive oxygen species | 0.008967 | 0.049732 | 0.036396 |
| BP | GO:2000561 | regulation of CD4-positive, alpha-beta T cell proliferation | 0.008967 | 0.049732 | 0.036396 |
| BP | GO:0030032 | lamellipodium assembly | 0.008982 | 0.049732 | 0.036396 |
| BP | GO:1903578 | regulation of ATP metabolic process | 0.008982 | 0.049732 | 0.036396 |
| BP | GO:0033689 | negative regulation of osteoblast proliferation | 0.009052 | 0.049732 | 0.036396 |
| BP | GO:0034384 | high-density lipoprotein particle clearance | 0.009052 | 0.049732 | 0.036396 |
| BP | GO:0042532 | negative regulation of tyrosine phosphorylation of STAT protein | 0.009052 | 0.049732 | 0.036396 |
| BP | GO:0045725 | positive regulation of glycogen biosynthetic process | 0.009052 | 0.049732 | 0.036396 |
| BP | GO:0060766 | negative regulation of androgen receptor signaling pathway | 0.009052 | 0.049732 | 0.036396 |
| BP | GO:0070262 | peptidyl-serine dephosphorylation | 0.009052 | 0.049732 | 0.036396 |
| BP | GO:0070831 | basement membrane assembly | 0.009052 | 0.049732 | 0.036396 |
| BP | GO:0090280 | positive regulation of calcium ion import | 0.009052 | 0.049732 | 0.036396 |
| BP | GO:2001028 | positive regulation of endothelial cell chemotaxis | 0.009052 | 0.049732 | 0.036396 |
| BP | GO:0031116 | positive regulation of microtubule polymerization | 0.009111 | 0.049807 | 0.036451 |
| BP | GO:0035456 | response to interferon-beta | 0.009111 | 0.049807 | 0.036451 |
| BP | GO:0048710 | regulation of astrocyte differentiation | 0.009111 | 0.049807 | 0.036451 |
| BP | GO:0051085 | chaperone cofactor-dependent protein refolding | 0.009111 | 0.049807 | 0.036451 |
| BP | GO:1990776 | response to angiotensin | 0.009111 | 0.049807 | 0.036451 |
| BP | GO:0032869 | cellular response to insulin stimulus | 0.009196 | 0.050223 | 0.036755 |
| BP | GO:0090263 | positive regulation of canonical Wnt signaling pathway | 0.009368 | 0.051061 | 0.037368 |
| BP | GO:0090398 | cellular senescence | 0.009368 | 0.051061 | 0.037368 |
| BP | GO:0002821 | positive regulation of adaptive immune response | 0.009379 | 0.051073 | 0.037377 |
| BP | GO:0061462 | protein localization to lysosome | 0.009443 | 0.051371 | 0.037595 |
| BP | GO:0071222 | cellular response to lipopolysaccharide | 0.009616 | 0.052218 | 0.038215 |
| BP | GO:0006623 | protein targeting to vacuole | 0.009628 | 0.052218 | 0.038215 |
| BP | GO:0010591 | regulation of lamellipodium assembly | 0.009628 | 0.052218 | 0.038215 |
| BP | GO:0097696 | receptor signaling pathway via STAT | 0.009666 | 0.052376 | 0.038331 |
| BP | GO:0021885 | forebrain cell migration | 0.009855 | 0.053184 | 0.038922 |
| BP | GO:0030330 | DNA damage response, signal transduction by p53 class mediator | 0.009863 | 0.053184 | 0.038922 |
| BP | GO:0048662 | negative regulation of smooth muscle cell proliferation | 0.009863 | 0.053184 | 0.038922 |
| BP | GO:0050688 | regulation of defense response to virus | 0.009863 | 0.053184 | 0.038922 |
| BP | GO:2000628 | regulation of miRNA metabolic process | 0.009863 | 0.053184 | 0.038922 |
| BP | GO:0002702 | positive regulation of production of molecular mediator of immune response | 0.009908 | 0.053373 | 0.039061 |
| BP | GO:0060993 | kidney morphogenesis | 0.009959 | 0.053595 | 0.039223 |
| BP | GO:0046661 | male sex differentiation | 0.010123 | 0.054424 | 0.03983 |
| BP | GO:0002237 | response to molecule of bacterial origin | 0.010406 | 0.055891 | 0.040904 |
| BP | GO:0030838 | positive regulation of actin filament polymerization | 0.010574 | 0.056566 | 0.041397 |
| BP | GO:0006022 | aminoglycan metabolic process | 0.010581 | 0.056566 | 0.041397 |
| BP | GO:0045577 | regulation of B cell differentiation | 0.010604 | 0.056566 | 0.041397 |
| BP | GO:0090279 | regulation of calcium ion import | 0.010604 | 0.056566 | 0.041397 |
| BP | GO:1902253 | regulation of intrinsic apoptotic signaling pathway by p53 class mediator | 0.010604 | 0.056566 | 0.041397 |
| BP | GO:1902992 | negative regulation of amyloid precursor protein catabolic process | 0.010604 | 0.056566 | 0.041397 |
| BP | GO:1905476 | negative regulation of protein localization to membrane | 0.010604 | 0.056566 | 0.041397 |
| BP | GO:0007259 | receptor signaling pathway via JAK-STAT | 0.010716 | 0.056943 | 0.041673 |
| BP | GO:0007040 | lysosome organization | 0.010771 | 0.056943 | 0.041673 |
| BP | GO:0080171 | lytic vacuole organization | 0.010771 | 0.056943 | 0.041673 |
| BP | GO:0030278 | regulation of ossification | 0.01078 | 0.056943 | 0.041673 |
| BP | GO:0048675 | axon extension | 0.01078 | 0.056943 | 0.041673 |
| BP | GO:1901989 | positive regulation of cell cycle phase transition | 0.01078 | 0.056943 | 0.041673 |
| BP | GO:0002040 | sprouting angiogenesis | 0.010788 | 0.056943 | 0.041673 |
| BP | GO:0018279 | protein N-linked glycosylation via asparagine | 0.010798 | 0.056943 | 0.041673 |
| BP | GO:0036010 | protein localization to endosome | 0.010798 | 0.056943 | 0.041673 |
| BP | GO:0040037 | negative regulation of fibroblast growth factor receptor signaling pathway | 0.010798 | 0.056943 | 0.041673 |
| BP | GO:0061484 | hematopoietic stem cell homeostasis | 0.010798 | 0.056943 | 0.041673 |
| BP | GO:2001026 | regulation of endothelial cell chemotaxis | 0.010798 | 0.056943 | 0.041673 |
| BP | GO:0031952 | regulation of protein autophosphorylation | 0.010941 | 0.057208 | 0.041868 |
| BP | GO:0032691 | negative regulation of interleukin-1 beta production | 0.010941 | 0.057208 | 0.041868 |
| BP | GO:0048713 | regulation of oligodendrocyte differentiation | 0.010941 | 0.057208 | 0.041868 |
| BP | GO:0060428 | lung epithelium development | 0.010941 | 0.057208 | 0.041868 |
| BP | GO:0071548 | response to dexamethasone | 0.010941 | 0.057208 | 0.041868 |
| BP | GO:0009410 | response to xenobiotic stimulus | 0.010949 | 0.057208 | 0.041868 |
| BP | GO:0008584 | male gonad development | 0.010952 | 0.057208 | 0.041868 |
| BP | GO:0045598 | regulation of fat cell differentiation | 0.010952 | 0.057208 | 0.041868 |
| BP | GO:0106106 | cold-induced thermogenesis | 0.010952 | 0.057208 | 0.041868 |
| BP | GO:0120161 | regulation of cold-induced thermogenesis | 0.010952 | 0.057208 | 0.041868 |
| BP | GO:0006959 | humoral immune response | 0.011127 | 0.058065 | 0.042494 |
| BP | GO:1990845 | adaptive thermogenesis | 0.011197 | 0.058379 | 0.042724 |
| BP | GO:0051962 | positive regulation of nervous system development | 0.011219 | 0.058438 | 0.042768 |
| BP | GO:0008543 | fibroblast growth factor receptor signaling pathway | 0.011371 | 0.059116 | 0.043263 |
| BP | GO:1900182 | positive regulation of protein localization to nucleus | 0.011371 | 0.059116 | 0.043263 |
| BP | GO:0042159 | lipoprotein catabolic process | 0.011526 | 0.059419 | 0.043485 |
| BP | GO:0043517 | positive regulation of DNA damage response, signal transduction by p53 class mediator | 0.011526 | 0.059419 | 0.043485 |
| BP | GO:0044406 | adhesion of symbiont to host | 0.011526 | 0.059419 | 0.043485 |
| BP | GO:0051014 | actin filament severing | 0.011526 | 0.059419 | 0.043485 |
| BP | GO:0051044 | positive regulation of membrane protein ectodomain proteolysis | 0.011526 | 0.059419 | 0.043485 |
| BP | GO:0061469 | regulation of type B pancreatic cell proliferation | 0.011526 | 0.059419 | 0.043485 |
| BP | GO:0061684 | chaperone-mediated autophagy | 0.011526 | 0.059419 | 0.043485 |
| BP | GO:0070875 | positive regulation of glycogen metabolic process | 0.011526 | 0.059419 | 0.043485 |
| BP | GO:2000696 | regulation of epithelial cell differentiation involved in kidney development | 0.011526 | 0.059419 | 0.043485 |
| BP | GO:0061326 | renal tubule development | 0.011632 | 0.059819 | 0.043778 |
| BP | GO:0046546 | development of primary male sexual characteristics | 0.011643 | 0.059819 | 0.043778 |
| BP | GO:0030510 | regulation of BMP signaling pathway | 0.011669 | 0.059819 | 0.043778 |
| BP | GO:0042177 | negative regulation of protein catabolic process | 0.011669 | 0.059819 | 0.043778 |
| BP | GO:0120034 | positive regulation of plasma membrane bounded cell projection assembly | 0.011669 | 0.059819 | 0.043778 |
| BP | GO:1901222 | regulation of NIK/NF-kappaB signaling | 0.011669 | 0.059819 | 0.043778 |
| BP | GO:0030834 | regulation of actin filament depolymerization | 0.011802 | 0.060335 | 0.044156 |
| BP | GO:0030857 | negative regulation of epithelial cell differentiation | 0.011802 | 0.060335 | 0.044156 |
| BP | GO:0031113 | regulation of microtubule polymerization | 0.011802 | 0.060335 | 0.044156 |
| BP | GO:0045860 | positive regulation of protein kinase activity | 0.011829 | 0.060414 | 0.044214 |
| BP | GO:1902806 | regulation of cell cycle G1/S phase transition | 0.011857 | 0.060501 | 0.044277 |
| BP | GO:0048857 | neural nucleus development | 0.012018 | 0.061213 | 0.044798 |
| BP | GO:0080164 | regulation of nitric oxide metabolic process | 0.012018 | 0.061213 | 0.044798 |
| BP | GO:0010765 | positive regulation of sodium ion transport | 0.012262 | 0.061937 | 0.045328 |
| BP | GO:0035025 | positive regulation of Rho protein signal transduction | 0.012262 | 0.061937 | 0.045328 |
| BP | GO:0035308 | negative regulation of protein dephosphorylation | 0.012262 | 0.061937 | 0.045328 |
| BP | GO:0036336 | dendritic cell migration | 0.012262 | 0.061937 | 0.045328 |
| BP | GO:0036474 | cell death in response to hydrogen peroxide | 0.012262 | 0.061937 | 0.045328 |
| BP | GO:0045648 | positive regulation of erythrocyte differentiation | 0.012262 | 0.061937 | 0.045328 |
| BP | GO:0055094 | response to lipoprotein particle | 0.012262 | 0.061937 | 0.045328 |
| BP | GO:0060563 | neuroepithelial cell differentiation | 0.012262 | 0.061937 | 0.045328 |
| BP | GO:0090322 | regulation of superoxide metabolic process | 0.012262 | 0.061937 | 0.045328 |
| BP | GO:1902652 | secondary alcohol metabolic process | 0.012368 | 0.062246 | 0.045554 |
| BP | GO:0006509 | membrane protein ectodomain proteolysis | 0.012379 | 0.062246 | 0.045554 |
| BP | GO:0016601 | Rac protein signal transduction | 0.012379 | 0.062246 | 0.045554 |
| BP | GO:0042771 | intrinsic apoptotic signaling pathway in response to DNA damage by p53 class mediator | 0.012379 | 0.062246 | 0.045554 |
| BP | GO:0045214 | sarcomere organization | 0.012379 | 0.062246 | 0.045554 |
| BP | GO:0006165 | nucleoside diphosphate phosphorylation | 0.012546 | 0.062913 | 0.046042 |
| BP | GO:0034620 | cellular response to unfolded protein | 0.012546 | 0.062913 | 0.046042 |
| BP | GO:1901992 | positive regulation of mitotic cell cycle phase transition | 0.012546 | 0.062913 | 0.046042 |
| BP | GO:0007034 | vacuolar transport | 0.012571 | 0.062978 | 0.04609 |
| BP | GO:0002833 | positive regulation of response to biotic stimulus | 0.012664 | 0.063389 | 0.046391 |
| BP | GO:0001911 | negative regulation of leukocyte mediated cytotoxicity | 0.01287 | 0.064011 | 0.046846 |
| BP | GO:0018196 | peptidyl-asparagine modification | 0.01287 | 0.064011 | 0.046846 |
| BP | GO:0021801 | cerebral cortex radial glia-guided migration | 0.01287 | 0.064011 | 0.046846 |
| BP | GO:0022030 | telencephalon glial cell migration | 0.01287 | 0.064011 | 0.046846 |
| BP | GO:0032816 | positive regulation of natural killer cell activation | 0.01287 | 0.064011 | 0.046846 |
| BP | GO:0046716 | muscle cell cellular homeostasis | 0.01287 | 0.064011 | 0.046846 |
| BP | GO:2000679 | positive regulation of transcription regulatory region DNA binding | 0.01287 | 0.064011 | 0.046846 |
| BP | GO:0051402 | neuron apoptotic process | 0.0129 | 0.064013 | 0.046848 |
| BP | GO:0030968 | endoplasmic reticulum unfolded protein response | 0.012905 | 0.064013 | 0.046848 |
| BP | GO:0032729 | positive regulation of interferon-gamma production | 0.012905 | 0.064013 | 0.046848 |
| BP | GO:0010959 | regulation of metal ion transport | 0.013103 | 0.064791 | 0.047417 |
| BP | GO:0032868 | response to insulin | 0.013124 | 0.064791 | 0.047417 |
| BP | GO:0010761 | fibroblast migration | 0.013132 | 0.064791 | 0.047417 |
| BP | GO:0043525 | positive regulation of neuron apoptotic process | 0.013132 | 0.064791 | 0.047417 |
| BP | GO:1904645 | response to amyloid-beta | 0.013132 | 0.064791 | 0.047417 |
| BP | GO:2000648 | positive regulation of stem cell proliferation | 0.013132 | 0.064791 | 0.047417 |
| BP | GO:0043280 | positive regulation of cysteine-type endopeptidase activity involved in apoptotic process | 0.013181 | 0.064956 | 0.047537 |
| BP | GO:0032984 | protein-containing complex disassembly | 0.013208 | 0.064956 | 0.047537 |
| BP | GO:0010812 | negative regulation of cell-substrate adhesion | 0.013224 | 0.064956 | 0.047537 |
| BP | GO:0031343 | positive regulation of cell killing | 0.013224 | 0.064956 | 0.047537 |
| BP | GO:0060389 | pathway-restricted SMAD protein phosphorylation | 0.013224 | 0.064956 | 0.047537 |
| BP | GO:0046849 | bone remodeling | 0.013367 | 0.065598 | 0.048007 |
| BP | GO:0033674 | positive regulation of kinase activity | 0.013502 | 0.066203 | 0.04845 |
| BP | GO:0030835 | negative regulation of actin filament depolymerization | 0.013949 | 0.068211 | 0.04992 |
| BP | GO:0031295 | T cell costimulation | 0.013949 | 0.068211 | 0.04992 |
| BP | GO:1904646 | cellular response to amyloid-beta | 0.013949 | 0.068211 | 0.04992 |
| CC | GO:0005925 | focal adhesion | 1.21E-57 | 7.47E-55 | 5.26E-55 |
| CC | GO:0030055 | cell-substrate junction | 3.17E-57 | 9.73E-55 | 6.85E-55 |
| CC | GO:0062023 | collagen-containing extracellular matrix | 1.00E-52 | 2.05E-50 | 1.44E-50 |
| CC | GO:0005788 | endoplasmic reticulum lumen | 5.04E-35 | 7.76E-33 | 5.46E-33 |
| CC | GO:0005604 | basement membrane | 3.73E-19 | 4.58E-17 | 3.22E-17 |
| CC | GO:0032432 | actin filament bundle | 1.24E-15 | 1.27E-13 | 8.97E-14 |
| CC | GO:0001725 | stress fiber | 3.41E-14 | 2.62E-12 | 1.85E-12 |
| CC | GO:0097517 | contractile actin filament bundle | 3.41E-14 | 2.62E-12 | 1.85E-12 |
| CC | GO:0031252 | cell leading edge | 6.23E-14 | 4.26E-12 | 3.00E-12 |
| CC | GO:0034774 | secretory granule lumen | 2.30E-13 | 1.42E-11 | 9.97E-12 |
| CC | GO:0060205 | cytoplasmic vesicle lumen | 3.41E-13 | 1.91E-11 | 1.34E-11 |
| CC | GO:0031983 | vesicle lumen | 4.41E-13 | 2.26E-11 | 1.59E-11 |
| CC | GO:0042641 | actomyosin | 5.81E-13 | 2.75E-11 | 1.93E-11 |
| CC | GO:0030667 | secretory granule membrane | 9.00E-13 | 3.95E-11 | 2.78E-11 |
| CC | GO:0005775 | vacuolar lumen | 3.77E-12 | 1.53E-10 | 1.08E-10 |
| CC | GO:0043202 | lysosomal lumen | 3.99E-12 | 1.53E-10 | 1.08E-10 |
| CC | GO:0030027 | lamellipodium | 4.49E-12 | 1.62E-10 | 1.14E-10 |
| CC | GO:0098857 | membrane microdomain | 5.21E-12 | 1.78E-10 | 1.25E-10 |
| CC | GO:0005884 | actin filament | 5.71E-12 | 1.85E-10 | 1.30E-10 |
| CC | GO:0045121 | membrane raft | 1.54E-11 | 4.73E-10 | 3.33E-10 |
| CC | GO:0101002 | ficolin-1-rich granule | 1.80E-11 | 5.26E-10 | 3.70E-10 |
| CC | GO:0031091 | platelet alpha granule | 2.66E-11 | 7.44E-10 | 5.24E-10 |
| CC | GO:0030139 | endocytic vesicle | 3.37E-11 | 9.00E-10 | 6.33E-10 |
| CC | GO:0031093 | platelet alpha granule lumen | 6.68E-11 | 1.71E-09 | 1.20E-09 |
| CC | GO:0005581 | collagen trimer | 2.98E-10 | 7.17E-09 | 5.05E-09 |
| CC | GO:0009897 | external side of plasma membrane | 3.03E-10 | 7.17E-09 | 5.05E-09 |
| CC | GO:0005938 | cell cortex | 3.33E-10 | 7.46E-09 | 5.25E-09 |
| CC | GO:0043292 | contractile fiber | 3.40E-10 | 7.46E-09 | 5.25E-09 |
| CC | GO:0030016 | myofibril | 3.71E-10 | 7.88E-09 | 5.54E-09 |
| CC | GO:0042470 | melanosome | 4.08E-10 | 8.10E-09 | 5.70E-09 |
| CC | GO:0048770 | pigment granule | 4.08E-10 | 8.10E-09 | 5.70E-09 |
| CC | GO:0098644 | complex of collagen trimers | 4.28E-10 | 8.23E-09 | 5.79E-09 |
| CC | GO:0022626 | cytosolic ribosome | 7.50E-10 | 1.40E-08 | 9.83E-09 |
| CC | GO:0005793 | endoplasmic reticulum-Golgi intermediate compartment | 1.51E-09 | 2.72E-08 | 1.92E-08 |
| CC | GO:0030017 | sarcomere | 4.90E-09 | 8.62E-08 | 6.06E-08 |
| CC | GO:0140534 | endoplasmic reticulum protein-containing complex | 1.07E-08 | 1.83E-07 | 1.28E-07 |
| CC | GO:0030666 | endocytic vesicle membrane | 1.61E-08 | 2.67E-07 | 1.88E-07 |
| CC | GO:0098636 | protein complex involved in cell adhesion | 1.68E-08 | 2.72E-07 | 1.92E-07 |
| CC | GO:0030662 | coated vesicle membrane | 2.69E-08 | 4.24E-07 | 2.98E-07 |
| CC | GO:0005583 | fibrillar collagen trimer | 4.43E-08 | 6.64E-07 | 4.67E-07 |
| CC | GO:0098643 | banded collagen fibril | 4.43E-08 | 6.64E-07 | 4.67E-07 |
| CC | GO:0030135 | coated vesicle | 6.27E-08 | 9.17E-07 | 6.45E-07 |
| CC | GO:0045335 | phagocytic vesicle | 7.45E-08 | 1.07E-06 | 7.50E-07 |
| CC | GO:1904813 | ficolin-1-rich granule lumen | 1.20E-07 | 1.68E-06 | 1.18E-06 |
| CC | GO:0030175 | filopodium | 1.56E-07 | 2.14E-06 | 1.50E-06 |
| CC | GO:0030670 | phagocytic vesicle membrane | 2.38E-07 | 3.18E-06 | 2.24E-06 |
| CC | GO:0031904 | endosome lumen | 2.76E-07 | 3.62E-06 | 2.54E-06 |
| CC | GO:0030134 | COPII-coated ER to Golgi transport vesicle | 3.24E-07 | 4.15E-06 | 2.92E-06 |
| CC | GO:0002102 | podosome | 3.99E-07 | 5.00E-06 | 3.52E-06 |
| CC | GO:0030864 | cortical actin cytoskeleton | 4.40E-07 | 5.30E-06 | 3.73E-06 |
| CC | GO:0033116 | endoplasmic reticulum-Golgi intermediate compartment membrane | 4.40E-07 | 5.30E-06 | 3.73E-06 |
| CC | GO:0098858 | actin-based cell projection | 4.78E-07 | 5.65E-06 | 3.98E-06 |
| CC | GO:0031674 | I band | 5.07E-07 | 5.88E-06 | 4.13E-06 |
| CC | GO:0072562 | blood microparticle | 6.64E-07 | 7.56E-06 | 5.32E-06 |
| CC | GO:0042383 | sarcolemma | 7.14E-07 | 7.99E-06 | 5.62E-06 |
| CC | GO:0022627 | cytosolic small ribosomal subunit | 8.38E-07 | 9.21E-06 | 6.48E-06 |
| CC | GO:0001726 | ruffle | 1.00E-06 | 1.08E-05 | 7.62E-06 |
| CC | GO:0030018 | Z disc | 1.16E-06 | 1.23E-05 | 8.62E-06 |
| CC | GO:0005840 | ribosome | 1.19E-06 | 1.24E-05 | 8.71E-06 |
| CC | GO:0031258 | lamellipodium membrane | 1.57E-06 | 1.61E-05 | 1.14E-05 |
| CC | GO:0005766 | primary lysosome | 2.36E-06 | 2.34E-05 | 1.65E-05 |
| CC | GO:0042582 | azurophil granule | 2.36E-06 | 2.34E-05 | 1.65E-05 |
| CC | GO:0031527 | filopodium membrane | 2.87E-06 | 2.81E-05 | 1.97E-05 |
| CC | GO:0098637 | protein complex involved in cell-matrix adhesion | 4.66E-06 | 4.48E-05 | 3.15E-05 |
| CC | GO:0070820 | tertiary granule | 6.66E-06 | 6.30E-05 | 4.44E-05 |
| CC | GO:0005765 | lysosomal membrane | 7.16E-06 | 6.57E-05 | 4.63E-05 |
| CC | GO:0098852 | lytic vacuole membrane | 7.16E-06 | 6.57E-05 | 4.63E-05 |
| CC | GO:0031256 | leading edge membrane | 8.73E-06 | 7.90E-05 | 5.56E-05 |
| CC | GO:0030863 | cortical cytoskeleton | 1.05E-05 | 9.36E-05 | 6.58E-05 |
| CC | GO:0042105 | alpha-beta T cell receptor complex | 1.23E-05 | 0.000108 | 7.60E-05 |
| CC | GO:0019897 | extrinsic component of plasma membrane | 1.56E-05 | 0.000135 | 9.48E-05 |
| CC | GO:0035577 | azurophil granule membrane | 1.81E-05 | 0.000155 | 0.000109 |
| CC | GO:0005774 | vacuolar membrane | 2.10E-05 | 0.000177 | 0.000125 |
| CC | GO:0031941 | filamentous actin | 2.20E-05 | 0.000178 | 0.000126 |
| CC | GO:0071556 | integral component of lumenal side of endoplasmic reticulum membrane | 2.20E-05 | 0.000178 | 0.000126 |
| CC | GO:0098553 | lumenal side of endoplasmic reticulum membrane | 2.20E-05 | 0.000178 | 0.000126 |
| CC | GO:0071682 | endocytic vesicle lumen | 2.49E-05 | 0.000199 | 0.00014 |
| CC | GO:0005796 | Golgi lumen | 2.92E-05 | 0.000231 | 0.000162 |
| CC | GO:0101003 | ficolin-1-rich granule membrane | 3.22E-05 | 0.00025 | 0.000176 |
| CC | GO:0008305 | integrin complex | 4.00E-05 | 0.000308 | 0.000216 |
| CC | GO:0001527 | microfibril | 4.14E-05 | 0.000314 | 0.000221 |
| CC | GO:0045177 | apical part of cell | 4.23E-05 | 0.000317 | 0.000223 |
| CC | GO:0012507 | ER to Golgi transport vesicle membrane | 4.61E-05 | 0.000342 | 0.00024 |
| CC | GO:0009898 | cytoplasmic side of plasma membrane | 4.95E-05 | 0.000362 | 0.000255 |
| CC | GO:0005901 | caveola | 5.47E-05 | 0.000396 | 0.000279 |
| CC | GO:0008250 | oligosaccharyltransferase complex | 6.89E-05 | 0.000492 | 0.000346 |
| CC | GO:0044853 | plasma membrane raft | 7.01E-05 | 0.000494 | 0.000347 |
| CC | GO:0044391 | ribosomal subunit | 7.07E-05 | 0.000494 | 0.000347 |
| CC | GO:0005844 | polysome | 7.68E-05 | 0.000531 | 0.000374 |
| CC | GO:0015935 | small ribosomal subunit | 0.000103 | 0.000704 | 0.000495 |
| CC | GO:0098562 | cytoplasmic side of membrane | 0.000105 | 0.000707 | 0.000497 |
| CC | GO:0042827 | platelet dense granule | 0.000111 | 0.000742 | 0.000522 |
| CC | GO:0098576 | lumenal side of membrane | 0.000114 | 0.000747 | 0.000525 |
| CC | GO:0055038 | recycling endosome membrane | 0.000114 | 0.000747 | 0.000525 |
| CC | GO:0030137 | COPI-coated vesicle | 0.000155 | 0.001002 | 0.000705 |
| CC | GO:0036019 | endolysosome | 0.000161 | 0.001029 | 0.000724 |
| CC | GO:0031253 | cell projection membrane | 0.000164 | 0.001038 | 0.00073 |
| CC | GO:0005905 | clathrin-coated pit | 0.000168 | 0.001052 | 0.00074 |
| CC | GO:0034663 | endoplasmic reticulum chaperone complex | 0.000208 | 0.00129 | 0.000907 |
| CC | GO:0005770 | late endosome | 0.000247 | 0.001516 | 0.001067 |
| CC | GO:0030867 | rough endoplasmic reticulum membrane | 0.000284 | 0.001729 | 0.001216 |
| CC | GO:0044754 | autolysosome | 0.000339 | 0.002045 | 0.001439 |
| CC | GO:0030663 | COPI-coated vesicle membrane | 0.000349 | 0.002075 | 0.00146 |
| CC | GO:0030133 | transport vesicle | 0.000351 | 0.002075 | 0.00146 |
| CC | GO:0042611 | MHC protein complex | 0.000375 | 0.002197 | 0.001545 |
| CC | GO:0032587 | ruffle membrane | 0.000382 | 0.002216 | 0.001559 |
| CC | GO:0022625 | cytosolic large ribosomal subunit | 0.000445 | 0.002558 | 0.0018 |
| CC | GO:0005798 | Golgi-associated vesicle | 0.000495 | 0.002818 | 0.001983 |
| CC | GO:0044194 | cytolytic granule | 0.000525 | 0.002964 | 0.002085 |
| CC | GO:0005791 | rough endoplasmic reticulum | 0.000565 | 0.003159 | 0.002222 |
| CC | GO:0005912 | adherens junction | 0.000572 | 0.003168 | 0.002228 |
| CC | GO:0030669 | clathrin-coated endocytic vesicle membrane | 0.000721 | 0.003959 | 0.002785 |
| CC | GO:0005902 | microvillus | 0.000778 | 0.004179 | 0.00294 |
| CC | GO:0031089 | platelet dense granule lumen | 0.000779 | 0.004179 | 0.00294 |
| CC | GO:0034358 | plasma lipoprotein particle | 0.000793 | 0.004179 | 0.00294 |
| CC | GO:1990777 | lipoprotein particle | 0.000793 | 0.004179 | 0.00294 |
| CC | GO:0005771 | multivesicular body | 0.000795 | 0.004179 | 0.00294 |
| CC | GO:0005769 | early endosome | 0.000819 | 0.004266 | 0.003001 |
| CC | GO:0001772 | immunological synapse | 0.000856 | 0.004423 | 0.003111 |
| CC | GO:0019898 | extrinsic component of membrane | 0.001222 | 0.006265 | 0.004407 |
| CC | GO:0032994 | protein-lipid complex | 0.001385 | 0.00704 | 0.004953 |
| CC | GO:0031234 | extrinsic component of cytoplasmic side of plasma membrane | 0.001452 | 0.007322 | 0.005151 |
| CC | GO:0045334 | clathrin-coated endocytic vesicle | 0.001742 | 0.008708 | 0.006126 |
| CC | GO:0035579 | specific granule membrane | 0.001928 | 0.009562 | 0.006727 |
| CC | GO:0042581 | specific granule | 0.001973 | 0.009706 | 0.006828 |
| CC | GO:0016010 | dystrophin-associated glycoprotein complex | 0.002086 | 0.010181 | 0.007162 |
| CC | GO:0005767 | secondary lysosome | 0.002754 | 0.013306 | 0.009361 |
| CC | GO:0098791 | Golgi apparatus subcompartment | 0.002769 | 0.013306 | 0.009361 |
| CC | GO:0016607 | nuclear speck | 0.003322 | 0.015837 | 0.011141 |
| CC | GO:0030665 | clathrin-coated vesicle membrane | 0.003514 | 0.016622 | 0.011693 |
| CC | GO:0034364 | high-density lipoprotein particle | 0.003585 | 0.01683 | 0.011839 |
| CC | GO:0045178 | basal part of cell | 0.003724 | 0.017352 | 0.012207 |
| CC | GO:0031672 | A band | 0.003763 | 0.017401 | 0.012241 |
| CC | GO:0031901 | early endosome membrane | 0.004043 | 0.018556 | 0.013054 |
| CC | GO:0036464 | cytoplasmic ribonucleoprotein granule | 0.004204 | 0.019149 | 0.013471 |
| CC | GO:0001891 | phagocytic cup | 0.004346 | 0.019509 | 0.013724 |
| CC | GO:0032585 | multivesicular body membrane | 0.004346 | 0.019509 | 0.013724 |
| CC | GO:0090575 | RNA polymerase II transcription regulator complex | 0.004427 | 0.01973 | 0.013879 |
| CC | GO:0030136 | clathrin-coated vesicle | 0.004694 | 0.020699 | 0.014561 |
| CC | GO:0030660 | Golgi-associated vesicle membrane | 0.004712 | 0.020699 | 0.014561 |
| CC | GO:0043197 | dendritic spine | 0.005364 | 0.023396 | 0.016458 |
| CC | GO:0035578 | azurophil granule lumen | 0.005499 | 0.023815 | 0.016753 |
| CC | GO:0044309 | neuron spine | 0.005701 | 0.024518 | 0.017248 |
| CC | GO:0030176 | integral component of endoplasmic reticulum membrane | 0.005916 | 0.025266 | 0.017774 |
| CC | GO:0005790 | smooth endoplasmic reticulum | 0.006215 | 0.026361 | 0.018544 |
| CC | GO:0005795 | Golgi stack | 0.006509 | 0.027419 | 0.019289 |
| CC | GO:0030132 | clathrin coat of coated pit | 0.006662 | 0.027872 | 0.019607 |
| CC | GO:0014704 | intercalated disc | 0.00695 | 0.028701 | 0.02019 |
| CC | GO:0016324 | apical plasma membrane | 0.006997 | 0.028701 | 0.02019 |
| CC | GO:0090665 | glycoprotein complex | 0.007 | 0.028701 | 0.02019 |
| CC | GO:0042788 | polysomal ribosome | 0.007342 | 0.029902 | 0.021035 |
| CC | GO:0031902 | late endosome membrane | 0.007393 | 0.029912 | 0.021042 |
| CC | GO:0044291 | cell-cell contact zone | 0.007528 | 0.03026 | 0.021287 |
| CC | GO:0031985 | Golgi cisterna | 0.008493 | 0.033852 | 0.023814 |
| CC | GO:0031430 | M band | 0.008532 | 0.033852 | 0.023814 |
| CC | GO:0031227 | intrinsic component of endoplasmic reticulum membrane | 0.009556 | 0.037546 | 0.026412 |
| CC | GO:0035770 | ribonucleoprotein granule | 0.009585 | 0.037546 | 0.026412 |
| CC | GO:0005801 | cis-Golgi network | 0.010929 | 0.042504 | 0.0299 |
| CC | GO:0032154 | cleavage furrow | 0.011029 | 0.042504 | 0.0299 |
| CC | GO:0043034 | costamere | 0.011058 | 0.042504 | 0.0299 |
| CC | GO:1904724 | tertiary granule lumen | 0.012279 | 0.046903 | 0.032994 |
| CC | GO:0030496 | midbody | 0.012357 | 0.046912 | 0.033001 |
| CC | GO:0016363 | nuclear matrix | 0.012784 | 0.048232 | 0.03393 |
| CC | GO:0005681 | spliceosomal complex | 0.016129 | 0.060484 | 0.042549 |
| CC | GO:0097197 | tetraspanin-enriched microdomain | 0.016442 | 0.061082 | 0.042969 |
| CC | GO:0043209 | myelin sheath | 0.016487 | 0.061082 | 0.042969 |
| CC | GO:0055037 | recycling endosome | 0.018655 | 0.068701 | 0.048329 |
| CC | GO:0098978 | glutamatergic synapse | 0.019198 | 0.070279 | 0.049439 |
| MF | GO:0005201 | extracellular matrix structural constituent | 4.68E-31 | 4.07E-28 | 3.23E-28 |
| MF | GO:0005178 | integrin binding | 1.24E-22 | 5.39E-20 | 4.27E-20 |
| MF | GO:0005518 | collagen binding | 2.34E-22 | 6.79E-20 | 5.38E-20 |
| MF | GO:0019838 | growth factor binding | 7.99E-20 | 1.74E-17 | 1.38E-17 |
| MF | GO:0003779 | actin binding | 3.03E-19 | 5.27E-17 | 4.17E-17 |
| MF | GO:0005539 | glycosaminoglycan binding | 1.35E-12 | 1.96E-10 | 1.55E-10 |
| MF | GO:0045296 | cadherin binding | 2.44E-12 | 3.03E-10 | 2.40E-10 |
| MF | GO:0030020 | extracellular matrix structural constituent conferring tensile strength | 5.26E-12 | 5.72E-10 | 4.53E-10 |
| MF | GO:0004857 | enzyme inhibitor activity | 3.25E-11 | 3.14E-09 | 2.49E-09 |
| MF | GO:0051015 | actin filament binding | 7.32E-11 | 6.37E-09 | 5.05E-09 |
| MF | GO:0008201 | heparin binding | 5.86E-10 | 4.64E-08 | 3.67E-08 |
| MF | GO:0050840 | extracellular matrix binding | 2.32E-09 | 1.69E-07 | 1.34E-07 |
| MF | GO:0061134 | peptidase regulator activity | 1.73E-08 | 1.16E-06 | 9.20E-07 |
| MF | GO:0048407 | platelet-derived growth factor binding | 2.40E-08 | 1.49E-06 | 1.18E-06 |
| MF | GO:1901681 | sulfur compound binding | 4.37E-08 | 2.54E-06 | 2.01E-06 |
| MF | GO:0019955 | cytokine binding | 1.22E-07 | 6.63E-06 | 5.25E-06 |
| MF | GO:0008307 | structural constituent of muscle | 6.46E-07 | 3.30E-05 | 2.62E-05 |
| MF | GO:0043394 | proteoglycan binding | 7.09E-07 | 3.43E-05 | 2.72E-05 |
| MF | GO:0060090 | molecular adaptor activity | 7.68E-07 | 3.52E-05 | 2.79E-05 |
| MF | GO:0016504 | peptidase activator activity | 1.56E-06 | 6.78E-05 | 5.37E-05 |
| MF | GO:0140678 | molecular function inhibitor activity | 2.34E-06 | 9.68E-05 | 7.67E-05 |
| MF | GO:0030674 | protein-macromolecule adaptor activity | 2.49E-06 | 9.74E-05 | 7.72E-05 |
| MF | GO:0001618 | virus receptor activity | 2.57E-06 | 9.74E-05 | 7.72E-05 |
| MF | GO:0140272 | exogenous protein binding | 3.11E-06 | 0.000113 | 8.93E-05 |
| MF | GO:0031994 | insulin-like growth factor I binding | 3.55E-06 | 0.000124 | 9.79E-05 |
| MF | GO:0061135 | endopeptidase regulator activity | 5.52E-06 | 0.000185 | 0.000147 |
| MF | GO:0005520 | insulin-like growth factor binding | 7.11E-06 | 0.000229 | 0.000182 |
| MF | GO:0001968 | fibronectin binding | 8.47E-06 | 0.000263 | 0.000209 |
| MF | GO:0002020 | protease binding | 9.37E-06 | 0.000281 | 0.000223 |
| MF | GO:0031418 | L-ascorbic acid binding | 1.12E-05 | 0.000325 | 0.000258 |
| MF | GO:0003714 | transcription corepressor activity | 1.16E-05 | 0.000326 | 0.000258 |
| MF | GO:0004860 | protein kinase inhibitor activity | 1.46E-05 | 0.000396 | 0.000314 |
| MF | GO:0004601 | peroxidase activity | 1.76E-05 | 0.000465 | 0.000368 |
| MF | GO:0044389 | ubiquitin-like protein ligase binding | 1.99E-05 | 0.000509 | 0.000403 |
| MF | GO:0042277 | peptide binding | 2.06E-05 | 0.000512 | 0.000406 |
| MF | GO:0098631 | cell adhesion mediator activity | 2.18E-05 | 0.000527 | 0.000417 |
| MF | GO:0043236 | laminin binding | 2.49E-05 | 0.000585 | 0.000464 |
| MF | GO:0030021 | extracellular matrix structural constituent conferring compression resistance | 2.56E-05 | 0.000585 | 0.000464 |
| MF | GO:0016684 | oxidoreductase activity, acting on peroxide as acceptor | 2.66E-05 | 0.000593 | 0.00047 |
| MF | GO:0019210 | kinase inhibitor activity | 2.93E-05 | 0.000638 | 0.000506 |
| MF | GO:0004866 | endopeptidase inhibitor activity | 3.06E-05 | 0.000648 | 0.000514 |
| MF | GO:0031543 | peptidyl-proline dioxygenase activity | 3.21E-05 | 0.000664 | 0.000526 |
| MF | GO:0033218 | amide binding | 3.28E-05 | 0.000665 | 0.000527 |
| MF | GO:0070325 | lipoprotein particle receptor binding | 3.42E-05 | 0.000676 | 0.000536 |
| MF | GO:0043548 | phosphatidylinositol 3-kinase binding | 4.62E-05 | 0.000894 | 0.000708 |
| MF | GO:0051082 | unfolded protein binding | 5.12E-05 | 0.000968 | 0.000767 |
| MF | GO:0030414 | peptidase inhibitor activity | 5.92E-05 | 0.001094 | 0.000867 |
| MF | GO:0031625 | ubiquitin protein ligase binding | 6.03E-05 | 0.001094 | 0.000867 |
| MF | GO:0050431 | transforming growth factor beta binding | 7.35E-05 | 0.001305 | 0.001034 |
| MF | GO:0048029 | monosaccharide binding | 8.67E-05 | 0.001508 | 0.001195 |
| MF | GO:0019902 | phosphatase binding | 0.00012 | 0.002045 | 0.001621 |
| MF | GO:0051371 | muscle alpha-actinin binding | 0.000148 | 0.002411 | 0.001911 |
| MF | GO:0003735 | structural constituent of ribosome | 0.000152 | 0.002411 | 0.001911 |
| MF | GO:0005522 | profilin binding | 0.000155 | 0.002411 | 0.001911 |
| MF | GO:0042608 | T cell receptor binding | 0.000155 | 0.002411 | 0.001911 |
| MF | GO:0016209 | antioxidant activity | 0.000155 | 0.002411 | 0.001911 |
| MF | GO:0046332 | SMAD binding | 0.000212 | 0.003233 | 0.002562 |
| MF | GO:0004602 | glutathione peroxidase activity | 0.000218 | 0.003273 | 0.002594 |
| MF | GO:0005159 | insulin-like growth factor receptor binding | 0.000225 | 0.003324 | 0.002634 |
| MF | GO:0097493 | structural molecule activity conferring elasticity | 0.000269 | 0.003905 | 0.003095 |
| MF | GO:0042605 | peptide antigen binding | 0.000274 | 0.003912 | 0.0031 |
| MF | GO:0140416 | transcription regulator inhibitor activity | 0.000297 | 0.004174 | 0.003308 |
| MF | GO:0140297 | DNA-binding transcription factor binding | 0.000338 | 0.004664 | 0.003697 |
| MF | GO:0050750 | low-density lipoprotein particle receptor binding | 0.000398 | 0.005413 | 0.00429 |
| MF | GO:0050699 | WW domain binding | 0.00049 | 0.006558 | 0.005197 |
| MF | GO:0043028 | cysteine-type endopeptidase regulator activity involved in apoptotic process | 0.000512 | 0.00675 | 0.005349 |
| MF | GO:0001540 | amyloid-beta binding | 0.000531 | 0.006891 | 0.005461 |
| MF | GO:0019903 | protein phosphatase binding | 0.000642 | 0.008209 | 0.006505 |
| MF | GO:0005200 | structural constituent of cytoskeleton | 0.000667 | 0.008414 | 0.006668 |
| MF | GO:0019887 | protein kinase regulator activity | 0.0007 | 0.0087 | 0.006895 |
| MF | GO:0019199 | transmembrane receptor protein kinase activity | 0.000836 | 0.010248 | 0.008122 |
| MF | GO:0050681 | nuclear androgen receptor binding | 0.000872 | 0.010531 | 0.008346 |
| MF | GO:0019207 | kinase regulator activity | 0.000933 | 0.011121 | 0.008813 |
| MF | GO:0042805 | actinin binding | 0.001137 | 0.01337 | 0.010595 |
| MF | GO:0030246 | carbohydrate binding | 0.001243 | 0.01442 | 0.011427 |
| MF | GO:0003755 | peptidyl-prolyl cis-trans isomerase activity | 0.001651 | 0.018902 | 0.01498 |
| MF | GO:0017134 | fibroblast growth factor binding | 0.001975 | 0.021762 | 0.017247 |
| MF | GO:0035035 | histone acetyltransferase binding | 0.001975 | 0.021762 | 0.017247 |
| MF | GO:0008191 | metalloendopeptidase inhibitor activity | 0.001976 | 0.021762 | 0.017247 |
| MF | GO:0015026 | coreceptor activity | 0.002036 | 0.022143 | 0.017548 |
| MF | GO:0004175 | endopeptidase activity | 0.002144 | 0.02303 | 0.018251 |
| MF | GO:0061629 | RNA polymerase II-specific DNA-binding transcription factor binding | 0.002343 | 0.024756 | 0.019619 |
| MF | GO:0044183 | protein folding chaperone | 0.002362 | 0.024756 | 0.019619 |
| MF | GO:0016667 | oxidoreductase activity, acting on a sulfur group of donors | 0.00258 | 0.026722 | 0.021177 |
| MF | GO:0070064 | proline-rich region binding | 0.002662 | 0.026927 | 0.021339 |
| MF | GO:0140693 | molecular condensate scaffold activity | 0.002662 | 0.026927 | 0.021339 |
| MF | GO:0016859 | cis-trans isomerase activity | 0.002749 | 0.027485 | 0.021782 |
| MF | GO:0051020 | GTPase binding | 0.002861 | 0.028288 | 0.022418 |
| MF | GO:0004714 | transmembrane receptor protein tyrosine kinase activity | 0.002932 | 0.028663 | 0.022715 |
| MF | GO:0008022 | protein C-terminus binding | 0.003054 | 0.029193 | 0.023135 |
| MF | GO:0016248 | channel inhibitor activity | 0.003055 | 0.029193 | 0.023135 |
| MF | GO:0034713 | type I transforming growth factor beta receptor binding | 0.003087 | 0.029193 | 0.023135 |
| MF | GO:0003756 | protein disulfide isomerase activity | 0.003504 | 0.032434 | 0.025704 |
| MF | GO:0016864 | intramolecular oxidoreductase activity, transposing S-S bonds | 0.003504 | 0.032434 | 0.025704 |
| MF | GO:0051213 | dioxygenase activity | 0.00378 | 0.034617 | 0.027434 |
| MF | GO:0051087 | chaperone binding | 0.004196 | 0.037851 | 0.029997 |
| MF | GO:1990841 | promoter-specific chromatin binding | 0.004223 | 0.037851 | 0.029997 |
| MF | GO:0016922 | nuclear receptor binding | 0.004264 | 0.037851 | 0.029997 |
| MF | GO:0017166 | vinculin binding | 0.004411 | 0.038388 | 0.030422 |
| MF | GO:0044325 | transmembrane transporter binding | 0.004412 | 0.038388 | 0.030422 |
| MF | GO:0098632 | cell-cell adhesion mediator activity | 0.004673 | 0.040171 | 0.031835 |
| MF | GO:0023026 | MHC class II protein complex binding | 0.00471 | 0.040171 | 0.031835 |
| MF | GO:0019888 | protein phosphatase regulator activity | 0.00498 | 0.04206 | 0.033332 |
| MF | GO:0051393 | alpha-actinin binding | 0.005694 | 0.04707 | 0.037303 |
| MF | GO:0004197 | cysteine-type endopeptidase activity | 0.00573 | 0.04707 | 0.037303 |
| MF | GO:0016505 | peptidase activator activity involved in apoptotic process | 0.005735 | 0.04707 | 0.037303 |
| MF | GO:0008236 | serine-type peptidase activity | 0.006068 | 0.048901 | 0.038754 |
| MF | GO:0004859 | phospholipase inhibitor activity | 0.00607 | 0.048901 | 0.038754 |
| MF | GO:0019843 | rRNA binding | 0.006598 | 0.052662 | 0.041734 |
| MF | GO:0046966 | nuclear thyroid hormone receptor binding | 0.006819 | 0.053935 | 0.042743 |
| MF | GO:0003823 | antigen binding | 0.007442 | 0.058331 | 0.046227 |
| MF | GO:0017171 | serine hydrolase activity | 0.007642 | 0.059359 | 0.047042 |
| MF | GO:0032036 | myosin heavy chain binding | 0.008098 | 0.060734 | 0.048132 |
| MF | GO:0140313 | molecular sequestering activity | 0.008098 | 0.060734 | 0.048132 |
| MF | GO:0071813 | lipoprotein particle binding | 0.008098 | 0.060734 | 0.048132 |
| MF | GO:0071814 | protein-lipid complex binding | 0.008098 | 0.060734 | 0.048132 |
| MF | GO:0005525 | GTP binding | 0.008175 | 0.060789 | 0.048175 |
