# Supplementary material for: Glycosylation-related genes mediated prognostic signature contribute to prognostic prediction and treatment options in ovarian cancer: based on bulk and single‑cell RNA sequencing data
Source: BMC Cancer. 2024 Feb 14;24:207. doi: 10.1186/s12885-024-11908-4 (PMC10865697; doi:10.1186/s12885-024-11908-4)
Supplement: Supplementary file 10 — Supplementary Table 5. DO functional enrichment analyses were used to explore the potential functions of 1187 genes. [file 12885_2024_11908_MOESM10_ESM.docx]

Supplementary Table 5. DO functional enrichment analyses were used to explore the potential functions of 1187 genes.

| ID | Description | GeneRatio | BgRatio | pvalue | p.adjust | qvalue | geneID |
| --- | --- | --- | --- | --- | --- | --- | --- |
| DOID:13359 | Ehlers-Danlos syndrome | 18/860 | 26/10312 | 2.65E-14 | 2.55E-11 | 1.74E-11 | COL3A1/COL1A2/COL5A2/COL1A1/COL5A1/AEBP1/C1S/C1R/ADAMTS2/FKBP14/PLOD2/DCN/LOX/LUM/TGFB1/TNXB/PLOD1/DSE |
| DOID:2237 | hepatitis | 87/860 | 452/10312 | 4.54E-14 | 2.55E-11 | 1.74E-11 | COL1A1/THBS1/IL1R1/MMP2/LMAN1/FAP/GOLM1/CCL5/CCL4/CXCR4/PTPRC/PRF1/LCK/PTPN22/B2M/HLA-E/HLA-A/HLA-B/HLA-C/TMSB4X/EIF2AK2/ITGAL/DDX5/IL10RA/DDX6/STAT1/APOBEC3G/PSMB9/NFKBIA/CCL3/HLA-DPB1/MX1/ISG20/IRF3/MAF/IRF9/TNFRSF1B/JAK1/SH3KBP1/IRF1/MCL1/TAP1/HSP90AA1/JUN/ISG15/SOCS1/BCL2/TGFB1/DNAJB1/APOE/IGF1/VIM/ENG/APOC1/CD59/CD81/HSPA5/APOA1/GSTP1/CXCL12/MMP14/EPHX1/LAMB2/IGF2/VCAM1/NR4A1/FNDC3B/DLC1/TJP1/NPM1/JAG1/MYC/CDKN1A/CLU/SOCS3/LDLR/SCARB1/TNFRSF1A/NR2F1/PLAUR/IRS1/B4GALT1/LTBR/CTNNB1/EGR3/HMOX1/NFE2L2 |
| DOID:2043 | hepatitis B | 53/860 | 224/10312 | 1.56E-12 | 5.85E-10 | 3.99E-10 | IL1R1/MMP2/LMAN1/CCL5/CCL4/CXCR4/PRF1/IL2RB/HLA-A/HLA-C/TMSB4X/EIF2AK2/DOCK8/IL32/STAT1/APOBEC3G/SAMHD1/PSMB9/NFKBIA/HLA-DPB1/MX1/IRF3/IRF9/HSP90AA1/JUN/SOCS1/BCL2/TGFB1/CCND2/DNAJB1/APOE/CD59/HSPA5/APOA1/GSTP1/MMP14/IGF2/NR4A1/FNDC3B/DLC1/NPM1/JAG1/MYC/CDKN1A/SOCS3/TNFRSF1A/NR2F1/PLAUR/IRS1/CTNNB1/EGR3/HMOX1/NFE2L2 |
| DOID:3347 | osteosarcoma | 51/860 | 233/10312 | 9.02E-11 | 2.45E-08 | 1.67E-08 | COL1A1/FBN1/TIMP1/EFEMP2/SERPINF1/FN1/MMP2/DCN/ITGB1/PDPN/LUM/HSP90B1/CCL5/CXCR4/S100A4/HLA-A/TMSB4X/CD44/BUB3/ATM/JUN/DUSP1/BCL2/TRIM22/ACTA2/CAV1/CDH11/IGF1/GSTM3/ANXA2/AKAP12/PDGFRA/ANXA5/GSTP1/CTSB/CXCL12/PRDX2/RPL7A/TGFBI/GADD45A/IGF2/IMPDH2/CD9/EGR1/NPM1/CDKN1A/ITGAV/APEX1/FTL/CTNNB1/S100A6 |
| DOID:3070 | high grade glioma | 66/860 | 347/10312 | 1.09E-10 | 2.45E-08 | 1.67E-08 | IGFBP2/GJA1/CALD1/IGFBP4/MMP2/DCN/ITGB1/PDPN/APP/LAMC1/S100A4/DOK2/MDM4/ADAR/NFKBIA/CCND3/ANXA1/ATM/JUN/BCL2/PIK3R1/CFLAR/S100A13/CAV1/LAMA4/LGALS1/SERPING1/IGF1/MDK/GSTM3/EFEMP1/HSPA5/IL6ST/CALR/GSTP1/CTSB/CXCL12/MMP14/NRP2/LGALS3/NRP1/PDGFRB/FGFR1/CAV2/INSR/IGF2/PRDX6/RHOB/RPL5/GPNMB/PTTG1IP/FSCN1/MYC/CDKN1A/RAC1/SPINT2/BST2/ITGAV/PLAUR/MAP1LC3A/IL13RA1/CEBPB/CDKN1C/CTSH/AXL/STMN1 |
| DOID:4450 | renal cell carcinoma | 81/860 | 472/10312 | 1.67E-10 | 3.14E-08 | 2.14E-08 | SPARC/TIMP1/NNMT/IGFBP2/THBS1/IGFBP7/FN1/IGFBP4/MMP2/ITGB1/PDPN/TIMP2/IGFBP6/CXCR4/CD3E/B2M/HLA-A/CD44/TXNIP/IL10RA/SRSF7/STAT1/PSMB9/LNPEP/TNFRSF1B/CCND3/JAK1/ANXA1/PFN1/NR3C1/JUN/DUSP1/BCL2/PIK3R1/TGFB1/APOE/MGP/SFRP1/CAV1/LAMA4/COL18A1/IGF1/ENG/APOC1/ANXA2/PDGFRA/LRP1/HSPA5/TIMP3/CD151/GAS6/GSTP1/CXCL12/MMP14/IGFBP5/ANGPTL4/NRP1/PMEPA1/PDGFRB/FGFR1/INSR/BSG/RACK1/ST3GAL4/NME1/DLC1/COMT/JAG1/FSCN1/MYC/CDKN1A/EPAS1/CLU/SPINT2/BST2/TNFRSF1A/PLAUR/CTNNB1/AXL/NFE2L2/CTNNA1 |
| DOID:612 | primary immunodeficiency disease | 64/860 | 348/10312 | 8.97E-10 | 1.44E-07 | 9.84E-08 | CD63/CFH/CFI/CCL5/CXCR4/PTPRC/CD3D/TRAC/CD3G/CORO1A/IKZF3/CD3E/IL2RG/PRF1/RAC2/CD247/IKZF1/LCK/B2M/IL2RB/ITGB2/ITGAL/DOCK8/GLS/IVNS1ABP/CYBA/LCP2/HEXIM1/STAT1/APOBEC3G/ORAI1/ADAR/LRBA/TNFAIP3/REL/KMT2A/IRF9/IRF7/TNFRSF1B/SH3KBP1/ARPC1B/CIB1/MSN/TAPBP/TAP1/CTSC/ATM/ISG15/PIK3R1/ARHGEF1/APOE/C7/C3/CAV1/FCGRT/CD81/APOA1/IL6ST/CXCL12/PPP2CB/MYC/BST2/TNFRSF1A/PLAUR |
| DOID:854 | collagen disease | 21/860 | 57/10312 | 2.04E-09 | 2.88E-07 | 1.96E-07 | COL3A1/COL1A2/COL5A2/COL1A1/FBN1/COL5A1/AEBP1/C1S/C1R/ADAMTS2/FKBP14/PLOD2/DCN/LOX/LUM/NFKBIA/TGFB1/COL4A1/TNXB/PLOD1/DSE |
| DOID:2994 | germ cell cancer | 74/860 | 439/10312 | 2.47E-09 | 3.09E-07 | 2.11E-07 | PRDX4/IGFBP2/GJA1/CALD1/MMP2/ITGB1/PDPN/IL2RG/RUNX3/CD247/ARHGDIB/B2M/IL2RB/FMNL1/EMB/MECP2/RBL2/ARF6/JAK1/IRF1/ARPC1B/MCL1/HSP90AA1/ATM/JUN/BCL2/PIK3R1/XIST/CCND2/APOE/RBP1/MGP/SFRP1/CAV1/LGALS1/IGF1/SFRP4/MDK/CD59/PDGFRA/KRT19/LRP1/HSPA5/IL6ST/TM4SF1/CXCL12/MMP14/LGALS3/RTN4/PEBP1/INSR/RPL7A/ITGA6/SGK1/IGF2/ID4/RACK1/RHOB/KLF4/RPL5/NME1/ID1/GPNMB/MYC/CDKN1A/BCAM/ATF3/MTUS1/PLAUR/IRS1/MCFD2/CTNNB1/CDKN1C/AXL |
| DOID:8398 | osteoarthritis | 49/860 | 243/10312 | 4.05E-09 | 4.26E-07 | 2.91E-07 | COL1A1/EFEMP2/FN1/IL1R1/MMP2/COL11A1/P4HA2/SULF1/HTRA1/TIMP2/FAP/CCL5/CD247/MALAT1/B2M/CD53/ITGB2/CYBA/TNFAIP3/MAF/CALM1/TNFRSF1B/BCL2/PIK3R1/TGFB1/CAV1/SULF2/IGF1/FHL2/VIM/ENG/HSPA5/APOA1/CTSB/CXCL12/FGFR1/DDR2/IGF2/RHOB/EGR1/ADAMTS5/JAG1/SPON1/EPAS1/CRYAB/PRDX5/P4HA1/ERRFI1/HMOX1 |
| DOID:6713 | cerebrovascular disease | 61/860 | 337/10312 | 4.28E-09 | 4.26E-07 | 2.91E-07 | COL1A2/TIMP1/CD63/SERPINF1/CFH/MMP2/LMNA/VCAN/PLAT/CTSK/LOX/APP/LUM/TIMP2/B2M/RNF213/ALOX5AP/CCM2/CYBA/NFKBIA/ANXA1/NR3C1/UCP2/BCL2/TGFB1/APOE/ACTA2/SERPINE2/COL4A1/C3/CST3/SERPING1/IGF1/MDK/A2M/VIM/HSPG2/ENG/PDGFRA/TIMP3/GSTP1/CTSB/MMP14/PAWR/PDGFRB/DYNLL1/IGF2/VCAM1/ACTG1/EGR1/BNIP3L/ALDH2/LDLR/TNFRSF1A/PROS1/APEX1/HNMT/BNIP3/HMOX1/NFE2L2/RAMP1 |
| DOID:4448 | macular degeneration | 32/860 | 124/10312 | 4.55E-09 | 4.26E-07 | 2.91E-07 | TIMP1/IGFBP2/SERPINF1/LOXL1/CFH/MMP2/CFI/HTRA1/TIMP2/HLA-B/TRA2B/RORA/APOE/C3/CFD/CST3/SERPING1/IGF1/GSTM3/FBLN5/EFEMP1/ANXA5/TIMP3/GSTP1/GPX3/PDGFRB/ATP6AP2/CDKN1A/SCARB1/CRYAB/NFE2L2/CTNNA1 |
| DOID:326 | ischemia | 76/860 | 464/10312 | 5.56E-09 | 4.81E-07 | 3.28E-07 | THY1/COL1A1/SERPINF1/GJA1/THBS1/MMP2/LMNA/PLAT/CXCR4/CREM/WNK1/IL32/CD44/ARID1B/GABPB1/MECP2/CCL3/MBP/SF1/HNRNPA2B1/PPP1R15A/PRKACB/JAK1/ANXA1/HSPA1B/GRK2/NR3C1/UCP2/CTSC/DUSP1/SOCS1/BCL2/TGFB1/APOE/C3/CAV1/CST3/BEX3/IGF1/MDK/AKAP12/ANXA5/HSPA5/APOA1/CTSL/IL6ST/GAS6/CTSB/CXCL12/MMP14/PAWR/ANGPTL4/LGALS3/ADAMTS1/MYH10/PTGIS/FGFR1/TXN/SGK1/RACK1/LGMN/TJP1/MFGE8/LDHA/LTBP1/EPAS1/CLU/TNFRSF1A/ITGAV/APEX1/MAP1LC3A/PON2/MCFD2/AXL/BNIP3/HMOX1 |
| DOID:0060100 | musculoskeletal system cancer | 76/860 | 468/10312 | 8.20E-09 | 6.21E-07 | 4.23E-07 | COL1A1/FBN1/TIMP1/EFEMP2/SERPINF1/CALD1/FN1/MMP2/CREB3L1/DCN/CTSK/ITGB1/PDPN/LUM/HSP90B1/IGFBP6/CCL5/CXCR4/S100A4/HLA-A/TMSB4X/CD44/BUB3/RBPJ/ID2/MCL1/ATM/JUN/DUSP1/FUS/BCL2/TGFB1/TRIM22/ACTA2/CAV1/LGALS1/CDH11/IGF1/GSTM3/GRN/ANXA2/AKAP12/PDGFRA/LRP1/ANXA5/TM4SF1/GSTP1/CTSB/CXCL12/PRDX2/LGALS3/APOD/PDGFRB/FGFR1/RPL7A/TGFBI/GADD45A/IGF2/IMPDH2/CD9/ID1/EGR1/FBLIM1/NPM1/FSCN1/MYC/CDKN1A/TNFRSF1A/YAP1/ITGAV/PLAUR/APEX1/FTL/CTNNB1/DMD/S100A6 |
| DOID:201 | connective tissue cancer | 67/860 | 392/10312 | 8.28E-09 | 6.21E-07 | 4.23E-07 | COL1A1/FBN1/TIMP1/EFEMP2/SERPINF1/FN1/MMP2/CREB3L1/DCN/CTSK/ITGB1/PDPN/LUM/HSP90B1/CCL5/CXCR4/S100A4/HLA-A/TMSB4X/CD44/BUB3/RBPJ/ID2/MCL1/ATM/JUN/DUSP1/FUS/BCL2/TRIM22/ACTA2/CAV1/CDH11/IGF1/GSTM3/ANXA2/AKAP12/PDGFRA/LRP1/ANXA5/TM4SF1/GSTP1/CTSB/CXCL12/PRDX2/LGALS3/APOD/PDGFRB/RPL7A/TGFBI/GADD45A/IGF2/IMPDH2/CD9/ID1/EGR1/NPM1/MYC/CDKN1A/TNFRSF1A/YAP1/ITGAV/PLAUR/APEX1/FTL/CTNNB1/S100A6 |
| DOID:4960 | bone marrow cancer | 68/860 | 401/10312 | 8.87E-09 | 6.24E-07 | 4.25E-07 | COL1A1/THBS1/FN1/MMP2/ITGB1/BMP1/CCL4/CXCR4/PTPRC/B2M/SRGN/HLA-A/HLA-B/DDX5/TANK/CD44/NFKBIA/CCL3/MAF/CCND3/DAZAP2/ARPC1B/MCL1/HSP90AA1/JUN/SOCS1/BCL2/TGFB1/CCND2/SAMD9L/CYLD/CFLAR/APOE/SFRP1/IGLC2/SULF2/COL18A1/IGF1/SFRP4/ENG/APOC1/AKAP12/PDGFRA/LRP1/CALR/GSTP1/CXCL12/MMP14/EPHX1/PDGFRB/FGFR1/VCAM1/CD9/ID1/EGR1/NPM1/LDHB/JAG1/LDHA/MYC/RAC1/SOCS3/ITGAV/PLAUR/APEX1/CTNNB1/CEBPB/BNIP3 |
| DOID:4766 | embryoma | 62/860 | 352/10312 | 9.47E-09 | 6.26E-07 | 4.27E-07 | PRDX4/IGFBP2/CALD1/MMP2/ITGB1/PDPN/IL2RG/RUNX3/CD247/ARHGDIB/B2M/IL2RB/FMNL1/MECP2/RBL2/ARF6/JAK1/IRF1/ARPC1B/MCL1/HSP90AA1/ATM/JUN/BCL2/PIK3R1/APOE/RBP1/SFRP1/CAV1/LGALS1/IGF1/SFRP4/MDK/KRT19/LRP1/HSPA5/IL6ST/TM4SF1/CXCL12/MMP14/LGALS3/RTN4/PEBP1/RPL7A/ITGA6/SGK1/IGF2/ID4/RACK1/RHOB/RPL5/ID1/GPNMB/MYC/CDKN1A/BCAM/ATF3/MTUS1/PLAUR/IRS1/CDKN1C/AXL |
| DOID:0070004 | myeloid neoplasm | 64/860 | 371/10312 | 1.25E-08 | 7.84E-07 | 5.35E-07 | COL1A1/THBS1/FN1/MMP2/ITGB1/BMP1/CCL4/CXCR4/PTPRC/B2M/SRGN/HLA-A/HLA-B/DDX5/TANK/CD44/NFKBIA/CCL3/MAF/CCND3/DAZAP2/MCL1/HSP90AA1/JUN/SOCS1/BCL2/TGFB1/CCND2/CYLD/CFLAR/APOE/SFRP1/IGLC2/SULF2/COL18A1/IGF1/SFRP4/ENG/APOC1/AKAP12/LRP1/CALR/GSTP1/CXCL12/MMP14/EPHX1/PDGFRB/FGFR1/VCAM1/CD9/EGR1/NPM1/LDHB/JAG1/LDHA/MYC/RAC1/SOCS3/ITGAV/PLAUR/APEX1/CTNNB1/CEBPB/BNIP3 |
| DOID:12347 | osteogenesis imperfecta | 13/860 | 25/10312 | 1.74E-08 | 1.03E-06 | 7.04E-07 | SPARC/COL1A2/SERPINH1/COL1A1/KDELR2/PPIB/FKBP10/SERPINF1/P4HB/CREB3L1/P3H1/BMP1/CRTAP |
| DOID:184 | bone cancer | 53/860 | 287/10312 | 2.28E-08 | 1.28E-06 | 8.74E-07 | COL1A1/FBN1/TIMP1/EFEMP2/SERPINF1/FN1/MMP2/DCN/ITGB1/PDPN/LUM/HSP90B1/CCL5/CXCR4/S100A4/HLA-A/TMSB4X/CD44/BUB3/ATM/JUN/DUSP1/BCL2/TRIM22/ACTA2/CAV1/CDH11/IGF1/GSTM3/ANXA2/AKAP12/PDGFRA/ANXA5/GSTP1/CTSB/CXCL12/PRDX2/LGALS3/PDGFRB/RPL7A/TGFBI/GADD45A/IGF2/IMPDH2/CD9/EGR1/NPM1/CDKN1A/ITGAV/APEX1/FTL/CTNNB1/S100A6 |
| DOID:2349 | arteriosclerosis | 68/860 | 411/10312 | 2.46E-08 | 1.29E-06 | 8.82E-07 | FN1/CFH/MMP2/TNFRSF12A/LMNA/PALLD/PLAT/LOX/ITGB5/CCL5/B2M/IL32/ALOX5AP/CYBA/KLF2/HSPA1A/TNFRSF14/UCP2/RORA/SOCS1/SAMD9/TGFB1/APOE/ACTA2/MGP/COL4A2/C3/CAV1/LGALS1/CST3/IGF1/VIM/HSPG2/APOC1/GRN/CD59/LRP1/CPE/APOA1/IL6ST/GAS6/GSTP1/CTSB/PLTP/CXCL12/MMP14/RTN4/BSG/TXN/VCAM1/SCARB2/EGR1/ALDH2/EPAS1/CLU/SOCS3/LDLR/SCARB1/TNFRSF1A/ITGAV/PLAUR/PON2/LTBR/CDKN1C/ADAM9/HMOX1/NFE2L2/RAMP1 |
| DOID:688 | embryonal cancer | 64/860 | 378/10312 | 2.63E-08 | 1.29E-06 | 8.82E-07 | PRDX4/IGFBP2/CALD1/MMP2/ITGB1/PDPN/IL2RG/RUNX3/CD247/ARHGDIB/B2M/IL2RB/FMNL1/EMB/MECP2/RBL2/ARF6/JAK1/IRF1/ARPC1B/MCL1/HSP90AA1/ATM/JUN/BCL2/PIK3R1/CCND2/APOE/RBP1/SFRP1/CAV1/LGALS1/IGF1/SFRP4/MDK/KRT19/LRP1/HSPA5/IL6ST/TM4SF1/CXCL12/MMP14/LGALS3/RTN4/PEBP1/RPL7A/ITGA6/SGK1/IGF2/ID4/RACK1/RHOB/RPL5/ID1/GPNMB/MYC/CDKN1A/BCAM/ATF3/MTUS1/PLAUR/IRS1/CDKN1C/AXL |
| DOID:423 | myopathy | 78/860 | 498/10312 | 2.64E-08 | 1.29E-06 | 8.82E-07 | COL1A2/TIMP1/COL6A3/COL6A2/COL6A1/THBS1/MMP2/TPM2/FMOD/LMNA/COL12A1/PDLIM3/APP/TIMP2/GPC1/CAVIN1/PTPN22/HLA-E/HLA-A/HLA-B/HLA-C/EIF2AK2/ATXN7/HLA-F/YTHDC1/LPIN1/ORAI1/SYNE1/JUND/LPIN2/HNRNPA2B1/MBNL1/TNFRSF1B/UTRN/CBLB/TRA2B/NR3C1/RAB27A/BCL2/CELF2/TGFB1/TPM3/SMCHD1/DNAJB1/SYNE2/CFLAR/APOE/COL4A1/CST3/COL15A1/COL18A1/IGF1/HSPG2/GSN/DSTN/SDC2/CTSL/CTNNAL1/INSR/PLS3/SSPN/SLC25A6/LAMA2/CDKN1A/OBSL1/CLU/LAMP2/CRYAB/TNFRSF1A/HSPB6/PDLIM7/MAP1LC3A/SLC25A5/B4GALT1/PDLIM5/SGCB/MBNL2/DMD |
| DOID:0080639 | bone sarcoma | 51/860 | 275/10312 | 3.65E-08 | 1.69E-06 | 1.15E-06 | COL1A1/FBN1/TIMP1/EFEMP2/SERPINF1/FN1/MMP2/DCN/ITGB1/PDPN/LUM/HSP90B1/CCL5/CXCR4/S100A4/HLA-A/TMSB4X/CD44/BUB3/ATM/JUN/DUSP1/BCL2/TRIM22/ACTA2/CAV1/CDH11/IGF1/GSTM3/ANXA2/AKAP12/PDGFRA/ANXA5/GSTP1/CTSB/CXCL12/PRDX2/RPL7A/TGFBI/GADD45A/IGF2/IMPDH2/CD9/EGR1/NPM1/CDKN1A/ITGAV/APEX1/FTL/CTNNB1/S100A6 |
| DOID:2394 | ovarian cancer | 71/860 | 441/10312 | 3.75E-08 | 1.69E-06 | 1.15E-06 | SPARC/THY1/TIMP1/IGFBP2/EFEMP2/THBS1/MMP2/VCAN/ITGB1/PDPN/CXCR4/RUNX3/S100A4/CD44/MDM4/CASP4/TSC22D3/NFKBIA/RBL2/ETS1/TNFRSF1B/UTRN/CCND3/JAK1/MCL1/DUSP1/SOCS1/BCL2/TGFB1/SLC2A3/CFLAR/CAV1/IGF1/FHL2/ENG/GRN/CD59/LAPTM4B/CALR/GSTP1/MMP14/WWTR1/TSC22D1/PEBP1/BSG/TXN/CAMK2N1/IGF2/CD9/NME1/ID1/COMT/JAG1/HYAL2/MYC/CDKN1A/SPON1/YBX1/TNFRSF1A/MTUS1/YAP1/ITGAV/PLAUR/APEX1/ALDH1A1/RHOC/CTNNB1/STMN1/NFE2L2/S100A6/WFDC2 |
| DOID:0060085 | organ system benign neoplasm | 69/860 | 426/10312 | 4.62E-08 | 2.00E-06 | 1.36E-06 | SERPINH1/COL1A1/COL6A3/COL5A1/GJA1/MMP2/LMNA/VCAN/PLAT/LAMB1/CTSK/FBLN1/LOX/ITGB1/HLA-A/CD44/TNRC6B/RBL2/CCND3/ANXA1/MCL1/JUN/BCL2/TGFB1/CCND2/RBP1/CAV1/IGF1/A2M/ENG/ANXA2/TNXB/PDGFRA/CALR/TM4SF1/GSTP1/GPX3/CXCL12/MMP14/LGALS3/NRP1/PMEPA1/PDGFRB/PTGIS/FGFR1/BSG/TXN/IGF2/RHOB/SELENOP/KLF4/NME1/TUBA1A/EGR1/COMT/FSCN1/CDKN1A/BCAM/RAC1/EPAS1/CLU/TNFRSF1A/YAP1/PLAUR/PRDX1/CTNNB1/CEBPB/CDKN1C/S100A6 |
| DOID:520 | aortic disease | 29/860 | 116/10312 | 5.16E-08 | 2.15E-06 | 1.47E-06 | FBN1/TIMP1/EFEMP2/FN1/MMP2/MFAP5/PLAT/LOX/ITGB1/TIMP2/PRF1/CD44/APOE/ACTA2/MYLK/FBLN5/CD59/TIMP3/APOA1/IGKC/MMP14/RTN4/VCAM1/ACTG1/EGR1/ITGAV/PRDX1/LTBP4/HMOX1 |
| DOID:2256 | osteochondrodysplasia | 30/860 | 124/10312 | 6.76E-08 | 2.72E-06 | 1.85E-06 | SPARC/COL1A2/SERPINH1/COL1A1/FBN1/KDELR2/PPIB/FKBP10/SERPINF1/P4HB/FN1/CREB3L1/CTSK/COL11A1/BGN/P3H1/COL10A1/BMP1/TGFB1/IGF1/SFRP4/HSPG2/FGFR1/RAB13/DDR2/PAPSS2/CRTAP/GPC6/LIFR/CEBPB |
| DOID:1936 | atherosclerosis | 61/860 | 364/10312 | 8.29E-08 | 3.14E-06 | 2.14E-06 | FN1/CFH/MMP2/TNFRSF12A/PALLD/PLAT/ITGB5/CCL5/IL32/ALOX5AP/CYBA/KLF2/HSPA1A/TNFRSF14/UCP2/RORA/SOCS1/SAMD9/TGFB1/APOE/ACTA2/MGP/COL4A2/CAV1/LGALS1/CST3/IGF1/VIM/HSPG2/APOC1/GRN/CD59/LRP1/CPE/APOA1/IL6ST/GAS6/GSTP1/CTSB/PLTP/CXCL12/MMP14/RTN4/BSG/TXN/VCAM1/SCARB2/ALDH2/CLU/SOCS3/LDLR/SCARB1/TNFRSF1A/ITGAV/PLAUR/PON2/LTBR/CDKN1C/ADAM9/HMOX1/NFE2L2 |
| DOID:1883 | hepatitis C | 48/860 | 258/10312 | 8.37E-08 | 3.14E-06 | 2.14E-06 | THBS1/FAP/CCL5/CCL4/PTPRC/LCK/PTPN22/HLA-E/HLA-B/HLA-C/EIF2AK2/ITGAL/DDX5/IL10RA/DDX6/STAT1/SAMHD1/NFKBIA/MX1/ISG20/MAF/IRF9/JAK1/IRF1/MCL1/TAP1/ISG15/SOCS1/TGFB1/APOE/IGF1/VIM/ENG/APOC1/CD81/APOA1/EPHX1/LAMB2/IGF2/TJP1/MYC/CDKN1A/SOCS3/LDLR/SCARB1/IRS1/HMOX1/NFE2L2 |
| DOID:2348 | arteriosclerotic cardiovascular disease | 61/860 | 365/10312 | 9.18E-08 | 3.33E-06 | 2.27E-06 | FN1/CFH/MMP2/TNFRSF12A/PALLD/PLAT/ITGB5/CCL5/IL32/ALOX5AP/CYBA/KLF2/HSPA1A/TNFRSF14/UCP2/RORA/SOCS1/SAMD9/TGFB1/APOE/ACTA2/MGP/COL4A2/CAV1/LGALS1/CST3/IGF1/VIM/HSPG2/APOC1/GRN/CD59/LRP1/CPE/APOA1/IL6ST/GAS6/GSTP1/CTSB/PLTP/CXCL12/MMP14/RTN4/BSG/TXN/VCAM1/SCARB2/ALDH2/CLU/SOCS3/LDLR/SCARB1/TNFRSF1A/ITGAV/PLAUR/PON2/LTBR/CDKN1C/ADAM9/HMOX1/NFE2L2 |
| DOID:8893 | psoriasis | 24/860 | 87/10312 | 9.88E-08 | 3.47E-06 | 2.37E-06 | CFH/CFI/PTPN22/ITGB2/HLA-C/IL32/PSMB9/TNFRSF1B/ANXA1/TAP1/DUSP1/TGFB1/APOE/CAV1/IGF1/GSTP1/COMT/CDKN1A/EPAS1/CLU/TNFRSF1A/FABP5/LTBR/IL13RA1 |
| DOID:3627 | aortic aneurysm | 28/860 | 113/10312 | 1.07E-07 | 3.66E-06 | 2.50E-06 | FBN1/TIMP1/EFEMP2/FN1/MMP2/MFAP5/PLAT/LOX/ITGB1/TIMP2/PRF1/CD44/APOE/ACTA2/MYLK/FBLN5/CD59/TIMP3/APOA1/IGKC/MMP14/RTN4/ACTG1/EGR1/ITGAV/PRDX1/LTBP4/HMOX1 |
| DOID:2007 | degeneration of macula and posterior pole | 25/860 | 94/10312 | 1.18E-07 | 3.89E-06 | 2.65E-06 | TIMP1/IGFBP2/SERPINF1/LOXL1/CFH/CFI/HTRA1/TIMP2/HLA-B/RORA/APOE/C3/CFD/CST3/SERPING1/IGF1/FBLN5/EFEMP1/ANXA5/TIMP3/GSTP1/GPX3/PDGFRB/ATP6AP2/SCARB1 |
| DOID:10873 | Kuhnt-Junius degeneration | 14/860 | 33/10312 | 1.29E-07 | 4.10E-06 | 2.80E-06 | IGFBP2/SERPINF1/LOXL1/CFH/CFI/HTRA1/RORA/APOE/C3/SERPING1/IGF1/ANXA5/GSTP1/PDGFRB |
| DOID:9538 | multiple myeloma | 53/860 | 302/10312 | 1.31E-07 | 4.10E-06 | 2.80E-06 | COL1A1/MMP2/ITGB1/CCL4/CXCR4/PTPRC/B2M/SRGN/HLA-A/HLA-B/DDX5/TANK/CD44/NFKBIA/CCL3/MAF/CCND3/DAZAP2/MCL1/HSP90AA1/JUN/SOCS1/BCL2/TGFB1/CCND2/CYLD/CFLAR/APOE/SFRP1/IGLC2/SULF2/COL18A1/IGF1/SFRP4/ENG/APOC1/LRP1/GSTP1/CXCL12/EPHX1/VCAM1/CD9/EGR1/NPM1/JAG1/MYC/RAC1/ITGAV/PLAUR/APEX1/CTNNB1/CEBPB/BNIP3 |
| DOID:2151 | malignant ovarian surface epithelial-stromal neoplasm | 55/860 | 321/10312 | 1.73E-07 | 5.12E-06 | 3.49E-06 | THY1/TIMP1/IGFBP2/THBS1/MMP2/VCAN/ITGB1/PDPN/CXCR4/RUNX3/S100A4/CD44/MDM4/TSC22D3/NFKBIA/RBL2/ETS1/TNFRSF1B/CCND3/MCL1/DUSP1/SOCS1/BCL2/TGFB1/SLC2A3/CFLAR/CAV1/FHL2/ENG/GRN/LAPTM4B/GSTP1/MMP14/TSC22D1/BSG/TXN/CAMK2N1/IGF2/NME1/JAG1/HYAL2/MYC/CDKN1A/SPON1/YBX1/TNFRSF1A/MTUS1/ITGAV/ALDH1A1/RHOC/CTNNB1/STMN1/NFE2L2/S100A6/WFDC2 |
| DOID:4001 | ovarian carcinoma | 55/860 | 321/10312 | 1.73E-07 | 5.12E-06 | 3.49E-06 | THY1/TIMP1/IGFBP2/THBS1/MMP2/VCAN/ITGB1/PDPN/CXCR4/RUNX3/S100A4/CD44/MDM4/TSC22D3/NFKBIA/RBL2/ETS1/TNFRSF1B/CCND3/MCL1/DUSP1/SOCS1/BCL2/TGFB1/SLC2A3/CFLAR/CAV1/FHL2/ENG/GRN/LAPTM4B/GSTP1/MMP14/TSC22D1/BSG/TXN/CAMK2N1/IGF2/NME1/JAG1/HYAL2/MYC/CDKN1A/SPON1/YBX1/TNFRSF1A/MTUS1/ITGAV/ALDH1A1/RHOC/CTNNB1/STMN1/NFE2L2/S100A6/WFDC2 |
| DOID:11934 | head and neck cancer | 49/860 | 276/10312 | 2.74E-07 | 7.92E-06 | 5.40E-06 | TIMP1/GJA1/MMP2/ITGB1/CXCR4/RUNX3/CD44/TXNIP/STAT1/DDIT4/SH3KBP1/TAP1/BCL2/TGFB1/SLC2A3/CFLAR/APOE/CAV1/TCF21/COL18A1/IGF1/MDK/KRT19/MRC2/IFI27/TIMP3/IL6ST/GSTP1/CXCL12/EPHX1/CAV2/CTTN/DDR2/KLF4/JAG1/LDHA/MYC/CDKN1A/ALDH2/RAC1/EPAS1/SOCS3/CRYAB/MTUS1/ING2/ALDH1A1/CTNNB1/SPECC1/ENO1 |
| DOID:8692 | myeloid leukemia | 50/860 | 285/10312 | 3.02E-07 | 8.50E-06 | 5.80E-06 | IGFBP2/MMP2/PLAT/CXCR4/BTG1/RUNX3/CD96/B2M/ITGAL/NSD1/CHORDC1/KAT6A/SAMHD1/CCL3/SENP7/NSD3/KMT2A/CBLB/MCL1/KMT2E/JUN/SOCS1/BCL2/TGFB1/USP15/CFLAR/CAV1/IGF1/CEBPD/PDGFRA/SDC2/CALR/GSTP1/CXCL12/EPHX1/NRP2/NECTIN2/NRP1/FERMT2/FGFR1/IGF2/NPM1/LPP/HES1/MYC/CDKN1A/SOCS3/ITGAV/PLAUR/HMOX1 |
| DOID:526 | human immunodeficiency virus infectious disease | 42/860 | 222/10312 | 3.39E-07 | 9.24E-06 | 6.31E-06 | CCL5/CCL4/CXCR4/PTPRC/IL2RG/PRF1/LCK/B2M/IL2RB/HLA-A/ITGB2/HLA-B/HLA-C/IL32/CYBA/IL10RA/APOBEC3G/CCL3/IRF3/TNFRSF1B/IRF1/NR3C1/KMT2E/ATM/SOCS1/BCL2/TGFB1/TRIM22/C3/CAV1/LRP1/HSPA5/GPX3/CXCL12/IGF2/VCAM1/YBX1/SOCS3/BST2/TNFRSF1A/PLAUR/CEBPB |
| DOID:11054 | urinary bladder cancer | 45/860 | 246/10312 | 3.53E-07 | 9.24E-06 | 6.31E-06 | SPARC/CKAP4/IGFBP2/THBS1/FN1/MMP2/ITGB1/TIMP2/CXCR4/RUNX3/ARHGDIB/CD44/HSPA1A/ARL6IP5/CCND3/ANXA1/IRF1/DUSP1/SOCS1/BCL2/TGFB1/CFLAR/CAV1/VIM/TIMP3/CALR/GSTP1/CTSB/SOX4/EPHX1/ANGPTL4/LGALS3/IGF2/ID4/CD9/NME1/ID1/COMT/MYC/CDKN1A/PLAUR/APEX1/CTNNB1/SMTN/CTNNA1 |
| DOID:3459 | breast carcinoma | 74/860 | 493/10312 | 3.53E-07 | 9.24E-06 | 6.31E-06 | SPARC/IGFBP2/MAGED1/MMP2/DCN/ITGB1/HSP90B1/CCL5/CXCR4/RUNX3/RBM5/CD44/STAT1/PSMB9/CCNDBP1/TNFRSF1B/CCND3/ANXA1/HSP90AA1/ATM/ISG15/SOCS1/BCL2/TGFB1/CCND2/CAV1/CD55/IGF1/VIM/ENG/CD59/ANXA2/PDGFRA/KRT19/TIMP3/GSTP1/CXCL12/MMP14/IGFBP5/EPHX1/LGALS3/NRP1/ADAMTS1/PDGFRB/CAV2/BSG/GADD45A/TXN/IGF2/PRDX6/RACK1/CD9/NME1/EGR1/COMT/NPM1/FSCN1/MYC/CDKN1A/YBX1/RAC1/CLU/SOCS3/MTUS1/CTNND1/ITGAV/PLAUR/LOXL2/APEX1/RHOC/CTNNB1/ENO1/NFE2L2/CTNNA1 |
| DOID:5041 | esophageal cancer | 46/860 | 255/10312 | 3.98E-07 | 1.02E-05 | 6.94E-06 | MMP2/HTRA1/PDPN/HSP90B1/FAP/CXCR4/RUNX3/S100A4/HLA-A/HLA-B/CD44/RSRC2/PSMB9/TNFAIP3/ARL6IP5/ID2/TAP1/ATM/SOCS1/BCL2/TGFB1/SFRP1/CAV1/IGF1/MDK/AKAP12/HSPA5/CALR/GSTP1/CTSB/GPX3/COL14A1/MMP14/PBX1/CAV2/IGF2/ID1/EGR1/MYC/CDKN1A/BST2/PRDX1/RHOC/CDKN1C/HMOX1/CTNNA1 |
| DOID:2152 | ovary epithelial cancer | 56/860 | 338/10312 | 4.20E-07 | 1.05E-05 | 7.17E-06 | SPARC/THY1/TIMP1/IGFBP2/THBS1/MMP2/VCAN/ITGB1/PDPN/CXCR4/RUNX3/S100A4/CD44/MDM4/TSC22D3/NFKBIA/RBL2/ETS1/TNFRSF1B/CCND3/MCL1/DUSP1/SOCS1/BCL2/TGFB1/SLC2A3/CFLAR/CAV1/FHL2/ENG/GRN/LAPTM4B/GSTP1/MMP14/TSC22D1/BSG/TXN/CAMK2N1/IGF2/NME1/JAG1/HYAL2/MYC/CDKN1A/SPON1/YBX1/TNFRSF1A/MTUS1/ITGAV/ALDH1A1/RHOC/CTNNB1/STMN1/NFE2L2/S100A6/WFDC2 |
| DOID:10534 | stomach cancer | 65/860 | 416/10312 | 4.44E-07 | 1.09E-05 | 7.40E-06 | SPARC/COL1A2/IGFBP2/MMP2/CFI/LGALS3BP/LOX/ITGB1/TAGLN/CCL5/CCL4/CXCR4/RUNX3/CD247/RPS27/IL32/CD44/TXNIP/ADAR/TNFAIP3/ARL6IP5/CBLB/JAK1/ANXA1/IRF1/ARPC1B/KLF6/MCL1/ATM/JUN/BCL2/TGFB1/CCND2/CFLAR/SFRP1/LGALS1/RNASE1/KRT19/HSPA5/IL6ST/CALR/ID3/GPX3/CXCL12/MMP14/PMEPA1/PEBP1/DDR2/ID4/SELENOP/KLF4/ID1/DLC1/ASPH/MYC/CDKN1A/BST2/PLAUR/APEX1/PTPRG/RHOC/CTNNB1/BNIP3/HMOX1/S100A6 |
| DOID:3213 | demyelinating disease | 46/860 | 259/10312 | 6.31E-07 | 1.51E-05 | 1.03E-05 | COL1A1/TIMP1/IGFBP2/PLAT/CCL5/PTPRC/PRF1/PTPN22/HLA-A/ITGB2/HLA-B/HLA-C/IL32/PSMB9/ITGA1/HLA-DPB1/MX1/MBP/ICAM3/IRF1/GRK2/NR3C1/APOE/C7/C3/CFD/CAV1/CST3/GSTM3/A2M/GRN/IFI27/APOA1/GSTP1/CTSB/CXCL12/NECTIN2/RTN4/IGF2/JAG1/MYC/CRYAB/TNFRSF1A/AXL/HMOX1/STMN1 |
| DOID:1542 | head and neck carcinoma | 47/860 | 269/10312 | 7.77E-07 | 1.82E-05 | 1.24E-05 | TIMP1/GJA1/MMP2/ITGB1/CXCR4/RUNX3/CD44/STAT1/DDIT4/SH3KBP1/TAP1/BCL2/TGFB1/SLC2A3/CFLAR/APOE/CAV1/TCF21/COL18A1/IGF1/MDK/KRT19/MRC2/IFI27/TIMP3/IL6ST/GSTP1/CXCL12/EPHX1/CAV2/CTTN/DDR2/KLF4/LDHA/MYC/CDKN1A/ALDH2/RAC1/EPAS1/SOCS3/CRYAB/MTUS1/ING2/ALDH1A1/CTNNB1/SPECC1/ENO1 |
| DOID:13089 | intracranial arterial disease | 16/860 | 48/10312 | 8.39E-07 | 1.89E-05 | 1.29E-05 | COL1A2/TIMP1/MMP2/VCAN/CTSK/LOX/TIMP2/RNF213/BCL2/ACTA2/CST3/ENG/TIMP3/CTSB/MMP14/ACTG1 |
| DOID:3527 | cerebral arterial disease | 16/860 | 48/10312 | 8.39E-07 | 1.89E-05 | 1.29E-05 | COL1A2/TIMP1/MMP2/VCAN/CTSK/LOX/TIMP2/RNF213/BCL2/ACTA2/CST3/ENG/TIMP3/CTSB/MMP14/ACTG1 |
| DOID:10941 | intracranial aneurysm | 13/860 | 33/10312 | 1.02E-06 | 2.25E-05 | 1.54E-05 | COL1A2/TIMP1/MMP2/VCAN/CTSK/LOX/TIMP2/BCL2/CST3/ENG/TIMP3/CTSB/MMP14 |
| DOID:5844 | myocardial infarction | 68/860 | 453/10312 | 1.08E-06 | 2.33E-05 | 1.59E-05 | SPARC/COL3A1/COL1A1/THBS2/GJA1/THBS1/FN1/CFH/MMP2/VCAN/DCN/PLAT/ITGB1/CCL5/CXCR4/PTPRC/PRF1/ALOX5AP/CYBA/NFKBIA/VAMP8/TNFRSF1B/PSMB8/TNFRSF14/LRRFIP1/SOCS1/TGFB1/APOE/MGP/C7/COL4A1/C3/CST3/SERPING1/IGF1/MDK/GSN/ENG/CD59/LRP1/ANXA5/TIMP3/APOA1/IL6ST/CTSB/CXCL12/MMP14/EPHX1/DAB2/ADAMTS1/PEBP1/PTGIS/TXN/VCAM1/YBX1/ALDH2/EPAS1/CLU/LAMP2/TNFRSF1A/PLAUR/MAP1LC3A/CDKN1C/BNIP3/LRPAP1/HMOX1/NFE2L2/RAMP1 |
| DOID:3069 | malignant astrocytoma | 35/860 | 177/10312 | 1.11E-06 | 2.36E-05 | 1.61E-05 | IGFBP2/IGFBP4/MMP2/ITGB1/PDPN/APP/LAMC1/S100A4/DOK2/ADAR/NFKBIA/ANXA1/ATM/BCL2/PIK3R1/S100A13/LGALS1/SERPING1/GSTP1/CTSB/CXCL12/LGALS3/PDGFRB/IGF2/RHOB/RPL5/PTTG1IP/MYC/CDKN1A/BST2/ITGAV/PLAUR/MAP1LC3A/CDKN1C/CTSH |
| DOID:9884 | muscular dystrophy | 37/860 | 193/10312 | 1.21E-06 | 2.53E-05 | 1.72E-05 | TIMP1/COL6A3/COL6A2/COL6A1/MMP2/LMNA/COL12A1/PDLIM3/TIMP2/GPC1/CAVIN1/HLA-A/HLA-B/YTHDC1/SYNE1/HNRNPA2B1/MBNL1/UTRN/NR3C1/RAB27A/BCL2/CELF2/TPM3/SMCHD1/DNAJB1/SYNE2/CFLAR/COL4A1/HSPG2/CTNNAL1/INSR/SSPN/LAMA2/OBSL1/SGCB/MBNL2/DMD |
| DOID:219 | colon cancer | 62/860 | 403/10312 | 1.42E-06 | 2.89E-05 | 1.97E-05 | SERPINF1/MMP2/LMNA/IFITM3/DCN/PALLD/LGALS3BP/LOX/ITGB1/HSP90B1/PMP22/GZMA/PRF1/LCP1/TMSB4X/IL32/CD44/HSPA1A/STAT1/ITGA1/CCL3/TNFAIP3/TNFRSF1B/MCL1/HSP90AA1/ATM/JUN/BCL2/CELF2/TGFB1/CCND2/CFLAR/DDX17/CAV1/IGF1/MDK/SDC2/TIMP3/APOA1/CALR/CXCL12/MMP14/NECTIN2/LGALS3/NRP1/PMEPA1/CAV2/ITGA6/RPL29/DLC1/ASPH/FSCN1/MYC/CDKN1A/RAC1/TNFRSF1A/MTUS1/PLAUR/PRDX1/IRS1/CTNNB1/HMOX1 |
| DOID:2316 | brain ischemia | 52/860 | 318/10312 | 1.64E-06 | 3.25E-05 | 2.22E-05 | SERPINF1/GJA1/MMP2/LMNA/PLAT/CREM/WNK1/MECP2/MBP/HNRNPA2B1/PPP1R15A/PRKACB/JAK1/ANXA1/HSPA1B/NR3C1/UCP2/CTSC/DUSP1/SOCS1/BCL2/TGFB1/APOE/C3/CAV1/CST3/BEX3/IGF1/MDK/AKAP12/HSPA5/CTSL/IL6ST/CTSB/CXCL12/MMP14/PAWR/LGALS3/ADAMTS1/PTGIS/FGFR1/SGK1/RACK1/TJP1/LTBP1/CLU/TNFRSF1A/APEX1/MAP1LC3A/MCFD2/BNIP3/HMOX1 |
| DOID:1107 | esophageal carcinoma | 40/860 | 219/10312 | 1.65E-06 | 3.25E-05 | 2.22E-05 | MMP2/HTRA1/PDPN/HSP90B1/FAP/CXCR4/RUNX3/S100A4/HLA-A/HLA-B/CD44/PSMB9/TNFAIP3/ARL6IP5/ID2/TAP1/ATM/SOCS1/BCL2/TGFB1/SFRP1/CAV1/MDK/AKAP12/HSPA5/CALR/GSTP1/CTSB/GPX3/COL14A1/MMP14/PBX1/CAV2/ID1/EGR1/MYC/CDKN1A/BST2/PRDX1/RHOC |
| DOID:4905 | pancreatic carcinoma | 52/860 | 319/10312 | 1.81E-06 | 3.51E-05 | 2.40E-05 | SPARC/THBS1/MMP2/PALLD/PLAT/FAP/CXCR4/HLA-A/GLIPR2/HNRNPA2B1/JAK1/ANXA1/MCL1/JUN/BCL2/TGFB1/CFLAR/C3/CAV1/LGALS1/IGF1/QSOX1/APOC1/CD59/ANXA2/AKAP12/EFEMP1/ANXA5/CD151/CALR/GSTP1/CXCL12/MMP14/NRP2/PEBP1/ITGA6/KLF10/NME1/FSCN1/MYC/CDKN1A/RAC1/EPAS1/SOCS3/AKR7A2/CTNND1/LIFR/BTF3/RHOC/CTNNB1/ADAM9/CTNNA1 |
| DOID:11476 | osteoporosis | 29/860 | 136/10312 | 1.89E-06 | 3.60E-05 | 2.46E-05 | SPARC/COL1A2/COL1A1/THBS1/CTSK/VKORC1/CXCR4/IL32/CD44/TSC22D3/STAT1/ITGA1/TNFRSF1B/ARHGEF3/BCL2/TGFB1/APOE/CST3/IGF1/GSTP1/PLS3/ITGA6/IGF2/PLOD1/ID4/ALDH7A1/PDLIM4/MYC/IRS1 |
| DOID:0080011 | bone resorption disease | 29/860 | 137/10312 | 2.21E-06 | 4.05E-05 | 2.76E-05 | SPARC/COL1A2/COL1A1/THBS1/CTSK/VKORC1/CXCR4/IL32/CD44/TSC22D3/STAT1/ITGA1/TNFRSF1B/ARHGEF3/BCL2/TGFB1/APOE/CST3/IGF1/GSTP1/PLS3/ITGA6/IGF2/PLOD1/ID4/ALDH7A1/PDLIM4/MYC/IRS1 |
| DOID:2377 | multiple sclerosis | 43/860 | 246/10312 | 2.27E-06 | 4.05E-05 | 2.76E-05 | COL1A1/TIMP1/PLAT/CCL5/PTPRC/PRF1/PTPN22/HLA-A/HLA-B/HLA-C/IL32/PSMB9/ITGA1/HLA-DPB1/MX1/MBP/ICAM3/IRF1/GRK2/NR3C1/APOE/C7/C3/CFD/CAV1/CST3/GSTM3/A2M/GRN/IFI27/APOA1/GSTP1/CTSB/CXCL12/NECTIN2/RTN4/IGF2/JAG1/CRYAB/TNFRSF1A/AXL/HMOX1/STMN1 |
| DOID:1575 | rheumatic disease | 42/860 | 238/10312 | 2.31E-06 | 4.05E-05 | 2.76E-05 | COL3A1/COL1A2/COL1A1/RCN1/FBN1/TIMP1/THBS1/IL1R1/CFH/MMP2/PLOD2/LMNA/ITGB5/CCL4/CXCR4/PTPRC/CD247/PTPN22/TMSB4X/CCL3/HLA-DPB1/MX1/TNFAIP3/ICAM3/TAP1/TGFB1/C3/CAV1/COL15A1/IGF1/ENG/PDGFRA/ANXA5/MMP14/PDGFRB/INSR/NDN/RBFOX2/CLU/ITGAV/PLAUR/IL13RA1 |
| DOID:418 | systemic scleroderma | 42/860 | 238/10312 | 2.31E-06 | 4.05E-05 | 2.76E-05 | COL3A1/COL1A2/COL1A1/RCN1/FBN1/TIMP1/THBS1/IL1R1/CFH/MMP2/PLOD2/LMNA/ITGB5/CCL4/CXCR4/PTPRC/CD247/PTPN22/TMSB4X/CCL3/HLA-DPB1/MX1/TNFAIP3/ICAM3/TAP1/TGFB1/C3/CAV1/COL15A1/IGF1/ENG/PDGFRA/ANXA5/MMP14/PDGFRB/INSR/NDN/RBFOX2/CLU/ITGAV/PLAUR/IL13RA1 |
| DOID:419 | scleroderma | 42/860 | 238/10312 | 2.31E-06 | 4.05E-05 | 2.76E-05 | COL3A1/COL1A2/COL1A1/RCN1/FBN1/TIMP1/THBS1/IL1R1/CFH/MMP2/PLOD2/LMNA/ITGB5/CCL4/CXCR4/PTPRC/CD247/PTPN22/TMSB4X/CCL3/HLA-DPB1/MX1/TNFAIP3/ICAM3/TAP1/TGFB1/C3/CAV1/COL15A1/IGF1/ENG/PDGFRA/ANXA5/MMP14/PDGFRB/INSR/NDN/RBFOX2/CLU/ITGAV/PLAUR/IL13RA1 |
| DOID:4362 | cervical cancer | 35/860 | 187/10312 | 4.15E-06 | 7.17E-05 | 4.89E-05 | SPARC/SERPINH1/EFEMP2/MMP2/PDPN/TIMP2/CXCR4/HLA-B/ADAR/HLA-DPB1/IFITM1/KMT2A/CCND3/TAP1/KMT2E/IFI16/JUN/BCL2/SLC2A3/CFLAR/CD55/PDGFRA/KRT19/IFI27/IL6ST/CXCL12/MMP14/BSG/CD9/MYC/CDKN1A/RAC1/SOCS3/APEX1/CEBPB |
| DOID:9588 | encephalitis | 18/860 | 66/10312 | 4.69E-06 | 7.99E-05 | 5.45E-05 | PPIB/APP/EIF2AK2/IL32/STAT1/NFKBIA/CCL3/MBP/ISG15/CFLAR/APOE/GRN/IL6ST/APOD/CLDN11/VCAM1/TJP1/NPM1 |
| DOID:0080599 | Coronavirus infectious disease | 29/860 | 143/10312 | 5.45E-06 | 9.14E-05 | 6.24E-05 | THBS1/IL1R1/CFH/MMP2/IFITM3/PLAT/CCL5/CCL4/CD3E/IL2RG/IL2RB/HLA-B/HLA-C/STAT1/CCL3/MX1/IRF3/CCND3/ICAM3/TGFB1/C3/SERPING1/IGF1/A2M/LGALS3/CD9/RAB31/FTL/FTH1 |
| DOID:1781 | thyroid gland cancer | 42/860 | 247/10312 | 6.12E-06 | 0.000101 | 6.90E-05 | TIMP1/THBS1/FN1/MMP2/DCN/CXCR4/RUNX3/S100A4/HLA-C/PSMB9/ZFP36L2/ATM/BCL2/TGFB1/TPM3/IGF1/KRT19/LRP1/CTSB/LGALS3/PEBP1/BSG/TXN/ITGA6/CAMK2N1/RHOB/NUPR1/ID1/RPL15/RPL7/FSCN1/MYC/CDKN1A/PLAUR/APEX1/PRDX1/NDUFC2/CTNNB1/CEBPB/CDKN1C/DAD1/S100A6 |
| DOID:3963 | thyroid gland carcinoma | 40/860 | 231/10312 | 6.46E-06 | 0.000105 | 7.19E-05 | TIMP1/THBS1/FN1/MMP2/DCN/CXCR4/RUNX3/S100A4/HLA-C/PSMB9/ZFP36L2/ATM/BCL2/TGFB1/TPM3/IGF1/KRT19/LRP1/CTSB/LGALS3/PEBP1/BSG/TXN/ITGA6/CAMK2N1/RHOB/NUPR1/RPL15/RPL7/FSCN1/MYC/CDKN1A/APEX1/PRDX1/NDUFC2/CTNNB1/CEBPB/CDKN1C/DAD1/S100A6 |
| DOID:3068 | glioblastoma | 27/860 | 130/10312 | 7.17E-06 | 0.000114 | 7.75E-05 | IGFBP2/IGFBP4/MMP2/PDPN/APP/LAMC1/S100A4/NFKBIA/ATM/BCL2/PIK3R1/LGALS1/SERPING1/CTSB/CXCL12/LGALS3/PDGFRB/IGF2/RHOB/RPL5/MYC/CDKN1A/ITGAV/PLAUR/MAP1LC3A/CDKN1C/CTSH |
| DOID:14004 | thoracic aortic aneurysm | 12/860 | 33/10312 | 7.17E-06 | 0.000114 | 7.75E-05 | TIMP1/FN1/MMP2/MFAP5/LOX/TIMP2/ACTA2/MYLK/TIMP3/MMP14/RTN4/ACTG1 |
| DOID:0060084 | cell type benign neoplasm | 68/860 | 480/10312 | 8.39E-06 | 0.000131 | 8.94E-05 | COL1A1/COL5A1/THBS2/THBS1/CALD1/MMP2/VCAN/LAMB1/COL11A1/ITGB1/IGFBP6/CXCR4/S100A4/CD44/NCOR1/STAT1/LNPEP/MBP/CCND3/ANXA1/JUN/SOCS1/BCL2/TGFB1/AKAP13/CFLAR/RBP1/OGN/SFRP1/MEG3/CAV1/LGALS1/CDH11/IGF1/SFRP4/MATN2/GSN/TCF4/TNXB/PDGFRA/KRT19/EFEMP1/CTSL/IL6ST/GSTP1/CTSB/GPX3/CXCL12/EPHX1/IGF2/SELENOP/NME1/EGR1/COMT/FSCN1/MYC/CDKN1A/ALDH2/CLU/TNFRSF1A/PRDX1/CTNNB1/SLC9A3R2/CEBPB/STRAP/HMOX1/NFE2L2/CTNNA1 |
| DOID:6000 | congestive heart failure | 54/860 | 355/10312 | 9.60E-06 | 0.000148 | 0.000101 | TIMP1/FSTL1/GJA1/THBS1/MMP2/LMNA/MYL9/LOX/TPM1/TIMP2/CCL5/HLA-B/CYBA/OGT/HSPA1A/STAT1/PPP1R2/TNFRSF1B/HSPA1B/GRK2/NR3C1/UCP2/DUSP1/BCL2/TGFB1/CFLAR/APOE/CAV1/LAMA4/CST3/IGF1/MDK/ENG/TIMP3/APOA1/IL6ST/CALR/MMP14/LGALS3/RTN4/BSG/DYNLL1/VCAM1/NUPR1/ID1/TJP1/RAC1/TNFRSF1A/FTH1/BNIP3/HMOX1/NFE2L2/GNAQ/RAMP1 |
| DOID:104 | bacterial infectious disease | 54/860 | 359/10312 | 1.33E-05 | 0.000203 | 0.000138 | TIMP1/SERPINF1/CFH/ITGB1/HSP90B1/CCL5/CCL4/CXCR4/GZMA/PRF1/CD247/LCK/PTPN22/HLA-A/ITGB2/HLA-B/ITGAL/DOCK8/IL32/CYBA/STAT1/PSMB9/NFKBIA/CCL3/HLA-DPB1/IRF3/MAF/TNFRSF1B/CBLB/PSMB8/ARF6/IRF1/MCL1/HSPA1B/TAP1/GRK2/JUN/SOCS1/BCL2/TGFB1/APOE/C3/SERPING1/IGF1/A2M/VIM/VCAM1/ATF3/SOCS3/TNFRSF1A/PLAUR/NPDC1/HMOX1/NFE2L2 |
| DOID:1115 | sarcoma | 36/860 | 205/10312 | 1.35E-05 | 0.000203 | 0.000138 | THBS2/THBS1/MMP2/CREB3L1/CXCR4/MALAT1/MDM4/KMT2A/ID2/MCL1/FUS/BCL2/CAV1/IGF1/MDK/VIM/ENG/PDGFRA/LRP1/ANGPTL4/ADAMTS1/PDGFRB/FGFR1/GADD45A/IGF2/ID1/NPM1/JAG1/MYC/CDKN1A/ALDH2/RAC1/PLAUR/ALDH1A1/CTNNB1/AXL |
| DOID:1380 | endometrial cancer | 37/860 | 214/10312 | 1.48E-05 | 0.000219 | 0.000149 | SPARC/TIMP1/EFEMP2/MMP2/ITGB1/TIMP2/RUNX3/MALAT1/S100A4/TNFRSF1B/CCND3/JAK1/IRF1/ATM/JUN/BCL2/PIK3R1/CFLAR/CD55/MDK/CD59/PDGFRA/GPX3/MMP14/PDGFRB/BSG/CD9/COMT/MYC/EPAS1/BST2/TNFRSF1A/PLAUR/APEX1/FTL/CTNNB1/ERRFI1 |
| DOID:3319 | lymphangioleiomyomatosis | 9/860 | 21/10312 | 2.19E-05 | 0.00032 | 0.000219 | CTSK/PDPN/CXCR4/CD44/STAT1/TIMP3/BSG/PLAUR/CTNNB1 |
| DOID:10871 | age related macular degeneration | 19/860 | 80/10312 | 2.27E-05 | 0.000328 | 0.000223 | SERPINF1/LOXL1/CFH/CFI/HTRA1/HLA-B/RORA/APOE/C3/CFD/CST3/SERPING1/FBLN5/EFEMP1/TIMP3/GSTP1/GPX3/ATP6AP2/SCARB1 |
| DOID:363 | uterine cancer | 37/860 | 219/10312 | 2.51E-05 | 0.000357 | 0.000244 | SPARC/TIMP1/EFEMP2/MMP2/ITGB1/TIMP2/RUNX3/MALAT1/S100A4/TNFRSF1B/CCND3/JAK1/IRF1/ATM/JUN/BCL2/PIK3R1/CFLAR/CD55/MDK/CD59/PDGFRA/GPX3/MMP14/PDGFRB/BSG/CD9/COMT/MYC/EPAS1/BST2/TNFRSF1A/PLAUR/APEX1/FTL/CTNNB1/ERRFI1 |
| DOID:2893 | cervix carcinoma | 27/860 | 140/10312 | 2.95E-05 | 0.000413 | 0.000281 | SPARC/SERPINH1/EFEMP2/MMP2/PDPN/TIMP2/CXCR4/ADAR/IFITM1/CCND3/IFI16/JUN/BCL2/SLC2A3/CFLAR/KRT19/IFI27/IL6ST/CXCL12/MMP14/CD9/MYC/CDKN1A/RAC1/SOCS3/APEX1/CEBPB |
| DOID:9120 | amyloidosis | 13/860 | 43/10312 | 2.97E-05 | 0.000413 | 0.000281 | MMP2/APP/OSMR/B2M/ICAM3/APOE/CST3/GSN/APOA1/CALR/ITM2B/GPNMB/TNFRSF1A |
| DOID:9119 | acute myeloid leukemia | 36/860 | 216/10312 | 4.34E-05 | 0.000587 | 0.0004 | IGFBP2/MMP2/PLAT/CXCR4/BTG1/RUNX3/CD96/B2M/ITGAL/NSD1/KAT6A/SAMHD1/CCL3/SENP7/KMT2A/CBLB/KMT2E/SOCS1/BCL2/CFLAR/CAV1/IGF1/CEBPD/CALR/GSTP1/CXCL12/EPHX1/NRP1/FERMT2/FGFR1/NPM1/LPP/MYC/ITGAV/PLAUR/HMOX1 |
| DOID:3748 | esophagus squamous cell carcinoma | 27/860 | 143/10312 | 4.36E-05 | 0.000587 | 0.0004 | MMP2/HTRA1/FAP/CXCR4/RUNX3/PSMB9/TNFAIP3/ARL6IP5/ID2/TAP1/ATM/SOCS1/BCL2/CAV1/MDK/CALR/GSTP1/GPX3/COL14A1/MMP14/PBX1/CAV2/ID1/CDKN1A/BST2/PRDX1/RHOC |
| DOID:127 | leiomyoma | 24/860 | 120/10312 | 4.38E-05 | 0.000587 | 0.0004 | COL1A1/COL5A1/MMP2/VCAN/LAMB1/IGFBP6/S100A4/CD44/LNPEP/BCL2/TGFB1/RBP1/SFRP1/CAV1/LGALS1/IGF1/IGF2/EGR1/COMT/FSCN1/MYC/CDKN1A/TNFRSF1A/CEBPB |
| DOID:3376 | bone osteosarcoma | 14/860 | 51/10312 | 4.91E-05 | 0.00065 | 0.000443 | EFEMP2/ITGB1/LUM/BUB3/ATM/JUN/BCL2/ACTA2/CAV1/IGF1/CXCL12/IMPDH2/CDKN1A/S100A6 |
| DOID:635 | acquired immunodeficiency syndrome | 19/860 | 85/10312 | 5.59E-05 | 0.000732 | 0.000499 | CCL5/CCL4/CXCR4/HLA-A/HLA-B/HLA-C/CYBA/IL10RA/TNFRSF1B/NR3C1/BCL2/LRP1/CXCL12/IGF2/YBX1/BST2/TNFRSF1A/PLAUR/CEBPB |
| DOID:5517 | stomach carcinoma | 34/860 | 202/10312 | 5.73E-05 | 0.000741 | 0.000505 | SPARC/COL1A2/IGFBP2/MMP2/LOX/ITGB1/TAGLN/CCL4/RUNX3/CD247/CD44/TNFAIP3/CBLB/ANXA1/IRF1/JUN/BCL2/TGFB1/CCND2/LGALS1/HSPA5/CXCL12/PMEPA1/PEBP1/ID4/SELENOP/KLF4/MYC/CDKN1A/APEX1/RHOC/CTNNB1/BNIP3/HMOX1 |
| DOID:3454 | brain infarction | 36/860 | 220/10312 | 6.44E-05 | 0.000824 | 0.000562 | SERPINF1/CFH/PLAT/APP/ALOX5AP/NFKBIA/ANXA1/NR3C1/BCL2/TGFB1/APOE/SERPINE2/C3/CST3/SERPING1/MDK/A2M/VIM/PDGFRA/CTSB/PAWR/PDGFRB/DYNLL1/IGF2/EGR1/BNIP3L/ALDH2/LDLR/TNFRSF1A/PROS1/APEX1/HNMT/BNIP3/HMOX1/NFE2L2/RAMP1 |
| DOID:5082 | liver cirrhosis | 51/860 | 354/10312 | 7.38E-05 | 0.000933 | 0.000637 | SERPINH1/COL1A1/FBN1/SERPINF1/FN1/MMP2/ITGB1/CCL5/PTPN22/B2M/ITGAL/GLS/ADAR/CENPC/IRF7/GRK2/NR3C1/ATM/BCL2/RGS2/TGFB1/CCND2/APOE/RBP1/CAV1/IGF1/MATN2/ENG/ANXA2/AKAP12/KRT19/APOA1/GSTP1/CXCL12/LGALS3/PDIA3/CAV2/DDR2/IGF2/VCAM1/EGR1/MYC/CDKN1A/ALDH2/RAC1/PLAUR/MAP1LC3A/CTNNB1/HMOX1/SLC40A1/NFE2L2 |
| DOID:0080006 | bone development disease | 38/860 | 239/10312 | 7.71E-05 | 0.000963 | 0.000657 | SPARC/COL1A2/SERPINH1/COL1A1/FBN1/KDELR2/PPIB/FKBP10/SERPINF1/GJA1/P4HB/FN1/CREB3L1/TPM2/CTSK/COL11A1/BGN/P3H1/COL10A1/BMP1/TCIRG1/JUN/TGFB1/IGF1/SFRP4/HSPG2/PDGFRA/EPHX1/FGFR1/RAB13/DDR2/PAPSS2/CRTAP/GPC6/POR/LIFR/CTNNB1/CEBPB |
| DOID:5409 | lung small cell carcinoma | 21/860 | 102/10312 | 8.36E-05 | 0.001033 | 0.000705 | IGFBP2/MMP2/ITGB1/CXCR4/RBM5/CD44/IL10RA/NR3C1/ATM/BCL2/PIK3R1/MARCKSL1/CAV1/CXCL12/LGALS3/CD9/DLC1/CDKN1A/PLAUR/RHOC/CEBPB |
| DOID:229 | female reproductive system disease | 58/860 | 421/10312 | 8.69E-05 | 0.001059 | 0.000722 | SERPINH1/TIMP1/SERPINF1/IL1R1/MMP2/RARRES2/CFI/LMNA/PLAT/LAMB1/PDPN/ACTN1/LAMC1/CCL5/MALAT1/PTPN22/IL2RB/IL32/NCOR1/LPIN1/NFKBIA/LNPEP/TNFRSF1B/NR3C1/BCL2/PIK3R1/TGFB1/S100A13/C3/STAR/CST3/IGF1/MDK/APOC1/PDGFRA/PGRMC1/IL6ST/GSTP1/EMP2/CTSB/CXCL12/MMP14/PDGFRB/INSR/IGF2/EGR1/COMT/CDKN1A/SOCS3/ITGAV/PLAUR/FOXL2/IRS1/NFIC/CTNNB1/AXL/HMOX1/GNAQ |
| DOID:11832 | visual epilepsy | 25/860 | 133/10312 | 8.93E-05 | 0.001059 | 0.000722 | GJA1/APP/CCL5/HSPA1A/MECP2/TNFRSF1B/NR3C1/DUSP1/SLC38A1/C7/MARCKSL1/BEX3/MARCKS/HSPA5/IL6ST/PAWR/MAP1B/RTN4/ADAMTS1/FGFR1/TUBA1A/EGR1/TNFRSF1A/APEX1/NFE2L2 |
| DOID:8857 | lupus erythematosus | 33/860 | 198/10312 | 8.93E-05 | 0.001059 | 0.000722 | CFH/ITGB1/CCL5/CCL4/PTPRC/IKZF3/PRF1/CD247/PTPN22/IL10RA/HSPA1A/MYO9B/SAMHD1/MECP2/ADAR/TNFRSF1B/JAK1/TAP1/JUN/BCL2/APOE/C3/VIM/APOA1/CXCL12/GADD45A/VCAM1/CDKN1A/RPL12/CLU/LDLR/TNFRSF1A/CEBPB |
| DOID:50 | thyroid gland disease | 37/860 | 232/10312 | 8.94E-05 | 0.001059 | 0.000722 | COL1A2/COL1A1/IGFBP2/PLOD2/PAM/CCL5/PTPRC/PTPN22/HLA-A/HLA-B/NCOR1/MBP/CCND3/ANXA1/UCP2/BCL2/TGFB1/SLC2A3/APOE/STAR/CAV1/LGALS1/IGF1/VIM/GSN/ANXA2/KRT19/ANXA5/APOA1/CTSL/LGALS3/MAP1B/FGFR1/PLOD1/EGR1/LRPAP1/HMOX1 |
| DOID:438 | autoimmune disease of the nervous system | 17/860 | 74/10312 | 9.48E-05 | 0.001104 | 0.000753 | MMP2/PMP22/PTPRC/PTPN22/HLA-E/IL2RB/IL32/NR3C1/TGFB1/APOE/C3/LGALS1/CST3/CD59/ANXA5/IL6ST/CTSB |
| DOID:8469 | influenza | 23/860 | 118/10312 | 9.52E-05 | 0.001104 | 0.000753 | CCL5/HLA-A/HLA-B/EIF2AK2/IL32/STAT1/APOBEC3G/ADAR/CCL3/MX1/IRF3/IFITM1/IRF7/ISG15/SOCS1/PIK3R1/TGFB1/ANXA2/TXN/ST3GAL4/ATP1B1/SOCS3/BST2 |
| DOID:230 | lateral sclerosis | 22/860 | 111/10312 | 0.000104 | 0.001185 | 0.000808 | TIMP1/P4HB/MMP2/LOX/APP/LAMC1/SETX/TNFRSF1B/RNF19A/FUS/APOE/CST3/IGF1/ENG/GRN/TIMP3/GSTP1/RTN4/TNFRSF1A/APEX1/PON2/VAPA |
| DOID:5520 | head and neck squamous cell carcinoma | 34/860 | 208/10312 | 0.000104 | 0.001185 | 0.000808 | TIMP1/GJA1/MMP2/ITGB1/CXCR4/CD44/STAT1/DDIT4/SH3KBP1/TAP1/BCL2/TGFB1/CFLAR/CAV1/MDK/MRC2/IFI27/IL6ST/GSTP1/CXCL12/CAV2/CTTN/DDR2/KLF4/MYC/CDKN1A/ALDH2/RAC1/SOCS3/CRYAB/MTUS1/ING2/ALDH1A1/CTNNB1 |
| DOID:4607 | biliary tract cancer | 41/860 | 269/10312 | 0.000108 | 0.001212 | 0.000827 | THBS1/MMP2/ANTXR1/CXCR4/RUNX3/TNFAIP3/IFITM1/ANXA1/MCL1/HSP90AA1/DUSP1/BCL2/TGFB1/CFLAR/APOE/CAV1/VIM/ANXA2/KRT19/LAPTM4B/APOA1/CD151/CALR/GSTP1/GPX3/CXCL12/PAWR/LGALS3/DDR2/NME1/TJP1/ASPH/FSCN1/MYC/CDKN1A/SOCS3/LDLR/MTUS1/CTNND1/RHOC/LRPAP1 |
| DOID:0080600 | COVID-19 | 18/860 | 82/10312 | 0.000112 | 0.001246 | 0.00085 | THBS1/IL1R1/CFH/MMP2/IFITM3/CCL5/CCL4/IL2RG/IL2RB/HLA-B/HLA-C/CCL3/C3/SERPING1/A2M/LGALS3/FTL/FTH1 |
| DOID:9074 | systemic lupus erythematosus | 32/860 | 192/10312 | 0.000114 | 0.001248 | 0.000851 | CFH/ITGB1/CCL5/CCL4/PTPRC/IKZF3/PRF1/CD247/PTPN22/IL10RA/HSPA1A/MYO9B/MECP2/ADAR/TNFRSF1B/JAK1/TAP1/JUN/BCL2/APOE/C3/VIM/APOA1/CXCL12/GADD45A/VCAM1/CDKN1A/RPL12/CLU/LDLR/TNFRSF1A/CEBPB |
| DOID:2527 | nephrosis | 28/860 | 159/10312 | 0.000114 | 0.001248 | 0.000851 | IL1R1/CFH/CFI/PDPN/CD247/NFKBIA/ITSN2/MAF/PSMB8/SH3KBP1/NR3C1/TGFB1/APOE/STAR/LGALS1/A2M/VIM/CD59/ANXA5/APOA1/CTSL/GSTP1/EMP2/GPX3/LAMB2/CDKN1A/CLU/KANK2 |
| DOID:10952 | nephritis | 45/860 | 306/10312 | 0.00012 | 0.00129 | 0.00088 | SPARC/COL3A1/COL1A1/FBN1/FN1/CFH/MMP2/ITGB1/CCL5/PRF1/IKZF1/ITGB2/ITGAL/ALOX5AP/CCL3/JUND/TNFRSF1B/PFN1/NR3C1/DUSP1/RGS2/TGFB1/CCND2/APOE/C7/C3/A2M/CD59/ANXA2/ANXA5/GAS6/MMP14/LGALS3/FGFR1/SGK1/VCAM1/NR4A1/CDKN1A/LTBP1/CLU/TNFRSF1A/ACSM3/B4GALT1/AXL/LRPAP1 |
| DOID:0080005 | bone remodeling disease | 33/860 | 201/10312 | 0.00012 | 0.00129 | 0.00088 | SPARC/COL1A2/COL1A1/GJA1/THBS1/CTSK/COL12A1/VKORC1/CXCR4/IL32/CD44/TSC22D3/STAT1/ITGA1/TCIRG1/KMT2A/TNFRSF1B/ARHGEF3/BCL2/TGFB1/APOE/CST3/IGF1/GSTP1/PLS3/ITGA6/IGF2/PLOD1/ID4/ALDH7A1/PDLIM4/MYC/IRS1 |
| DOID:224 | transient cerebral ischemia | 33/860 | 202/10312 | 0.000133 | 0.001408 | 0.000961 | SERPINF1/GJA1/MMP2/LMNA/PLAT/CREM/WNK1/MECP2/MBP/HNRNPA2B1/PPP1R15A/JAK1/UCP2/DUSP1/SOCS1/BCL2/APOE/C3/CAV1/BEX3/IGF1/HSPA5/CXCL12/PAWR/ADAMTS1/FGFR1/SGK1/RACK1/LTBP1/TNFRSF1A/MAP1LC3A/MCFD2/BNIP3 |
| DOID:3082 | interstitial lung disease | 39/860 | 254/10312 | 0.000134 | 0.001413 | 0.000964 | SPARC/COL1A2/COL1A1/TIMP1/AEBP1/MMP2/LMNA/CTSK/CCL5/CXCR4/STAT1/MECP2/TNFRSF1B/ANXA1/TAP1/NR3C1/SOCS1/TGFB1/CFLAR/C3/CAV1/COL18A1/IGF1/PDGFRA/TIMP3/APOA1/IL6ST/GSTP1/CXCL12/IGFBP5/EPHX1/SGK1/IGF2/EGR1/TNFRSF1A/CTNNB1/HMOX1/SLC40A1/NFE2L2 |
| DOID:0060099 | musculoskeletal system benign neoplasm | 14/860 | 56/10312 | 0.000149 | 0.001551 | 0.001058 | SERPINH1/COL6A3/MMP2/CTSK/HLA-A/BCL2/RBP1/CAV1/PDGFRB/BSG/CDKN1A/CLU/CTNNB1/CEBPB |
| DOID:3565 | meningioma | 18/860 | 84/10312 | 0.000155 | 0.0016 | 0.001092 | SPARC/MMP2/LAMB1/FAP/JAK1/ATM/BCL2/MEG3/CAV1/GSTM3/ENG/TIMP3/CTSL/GADD45A/CDKN1A/YWHAE/YAP1/PLAUR |
| DOID:4045 | muscle cancer | 22/860 | 115/10312 | 0.000178 | 0.001824 | 0.001244 | CALD1/MMP2/IGFBP6/CXCR4/MCL1/ATM/BCL2/TGFB1/LGALS1/GRN/PDGFRA/CXCL12/LGALS3/FGFR1/IGF2/FBLIM1/FSCN1/MYC/CDKN1A/APEX1/CTNNB1/DMD |
| DOID:552 | pneumonia | 28/860 | 164/10312 | 0.000197 | 0.001998 | 0.001363 | COL3A1/COL1A1/MMP2/PLAT/CTSK/PDPN/CCL5/CCL4/CXCR4/ITGB2/IL32/NFKBIA/TNFRSF1B/TAP1/GRK2/ATM/TGFB1/CFLAR/APOE/MYLK/C3/IL6ST/CXCL12/IGFBP5/TXN/TNFRSF1A/IL13RA1/HMOX1 |
| DOID:0050338 | primary bacterial infectious disease | 43/860 | 295/10312 | 0.000206 | 0.002069 | 0.001411 | SERPINF1/ITGB1/HSP90B1/CCL5/CCL4/CXCR4/GZMA/PRF1/CD247/LCK/PTPN22/HLA-A/HLA-B/ITGAL/IL32/CYBA/PSMB9/NFKBIA/CCL3/HLA-DPB1/IRF3/MAF/TNFRSF1B/CBLB/PSMB8/ARF6/IRF1/MCL1/TAP1/JUN/SOCS1/BCL2/TGFB1/APOE/C3/IGF1/VIM/ATF3/SOCS3/TNFRSF1A/PLAUR/NPDC1/NFE2L2 |
| DOID:8719 | in situ carcinoma | 25/860 | 140/10312 | 0.000209 | 0.002077 | 0.001417 | MMP2/PDPN/TIMP2/IGFBP6/RUNX3/S100A4/ANXA1/ATM/SOCS1/BCL2/CCND2/MGP/SFRP1/CAV1/IGF1/PDGFRA/LRP1/GSTP1/CDKN1A/SOCS3/APEX1/CTNNB1/ADAM9/BNIP3/CTNNA1 |
| DOID:7693 | abdominal aortic aneurysm | 17/860 | 79/10312 | 0.000222 | 0.002175 | 0.001484 | TIMP1/MMP2/PLAT/LOX/PRF1/CD44/APOE/FBLN5/CD59/APOA1/IGKC/MMP14/EGR1/ITGAV/PRDX1/LTBP4/HMOX1 |
| DOID:2596 | larynx cancer | 14/860 | 58/10312 | 0.000222 | 0.002175 | 0.001484 | THBS2/MMP2/CXCR4/CCND3/SOCS1/BCL2/CAV1/LGALS1/GSTM3/KRT19/CXCL12/MYC/CDKN1A/FOXL2 |
| DOID:0050624 | gastrointestinal system benign neoplasm | 23/860 | 125/10312 | 0.000235 | 0.002275 | 0.001552 | COL1A1/FBLN1/LOX/CD44/JUN/BCL2/TGFB1/CCND2/A2M/TNXB/PDGFRA/GSTP1/GPX3/CXCL12/PMEPA1/PTGIS/SELENOP/KLF4/FSCN1/CDKN1A/RAC1/TNFRSF1A/PRDX1 |
| DOID:3620 | central nervous system cancer | 31/860 | 191/10312 | 0.000237 | 0.002275 | 0.001552 | SPARC/COL1A2/MMP2/LAMB1/APP/FAP/RAC2/JAK1/ATM/BCL2/MEG3/CAV1/GSTM3/ENG/PDGFRA/HSPA5/TIMP3/CTSL/CXCL12/MMP14/GADD45A/IGF2/HES1/MYC/CDKN1A/YWHAE/RAC1/HES4/YAP1/PLAUR/CTNNB1 |
| DOID:4248 | coronary stenosis | 11/860 | 39/10312 | 0.000242 | 0.002305 | 0.001572 | MMP2/LOX/ITGB2/ITGAL/CYBA/APOE/IGF1/MDK/MYC/ITGAV/HMOX1 |
| DOID:1520 | colon carcinoma | 34/860 | 218/10312 | 0.000263 | 0.002488 | 0.001697 | SERPINF1/LMNA/DCN/PALLD/LOX/HSP90B1/PMP22/GZMA/PRF1/TMSB4X/CD44/HSPA1A/STAT1/TNFRSF1B/BCL2/TGFB1/CFLAR/CAV1/SDC2/TIMP3/APOA1/CALR/MMP14/NECTIN2/LGALS3/NRP1/ITGA6/ASPH/MYC/CDKN1A/RAC1/TNFRSF1A/CTNNB1/HMOX1 |
| DOID:399 | tuberculosis | 32/860 | 201/10312 | 0.000271 | 0.002539 | 0.001732 | ITGB1/CCL5/CCL4/CXCR4/GZMA/PRF1/CD247/PTPN22/HLA-A/HLA-B/ITGAL/IL32/CYBA/NFKBIA/CCL3/HLA-DPB1/MAF/TNFRSF1B/IRF1/MCL1/TAP1/JUN/SOCS1/BCL2/TGFB1/APOE/C3/VIM/ATF3/SOCS3/PLAUR/NFE2L2 |
| DOID:13223 | uterine fibroid | 12/860 | 46/10312 | 0.000285 | 0.002653 | 0.00181 | COL1A1/COL5A1/MMP2/VCAN/LAMB1/TNRC6B/BCL2/TGFB1/IGF1/EGR1/COMT/CEBPB |
| DOID:2355 | anemia | 45/860 | 319/10312 | 0.000313 | 0.00289 | 0.001971 | COL1A1/ITGB1/CLEC11A/CCL5/PRF1/HLA-E/RPS29/HLA-A/HLA-B/RPS27/EIF2AK2/ARID1B/PPP1R15A/TNFRSF1B/MSN/HSP90AA1/ATM/TGFB1/APOE/CD55/IGF1/MDK/A2M/CEBPD/CD59/CALR/GSTP1/RPS7/PRDX2/EPHX1/CYB5A/VCAM1/RPL5/EGR1/CYB5R3/NPM1/HES1/RPL15/BCAM/TNFRSF1A/FTL/TWSG1/RPS17/SLC40A1/RPL18 |
| DOID:0060086 | female reproductive organ benign neoplasm | 14/860 | 60/10312 | 0.000325 | 0.002965 | 0.002023 | COL1A1/COL5A1/MMP2/LMNA/VCAN/LAMB1/TNRC6B/BCL2/TGFB1/IGF1/EGR1/COMT/CEBPB/CDKN1C |
| DOID:576 | proteinuria | 37/860 | 247/10312 | 0.000327 | 0.002965 | 0.002023 | THBS1/IL1R1/CFH/CFI/PDPN/CXCR4/CD247/B2M/STAT1/NFKBIA/ITSN2/MAF/PSMB8/SH3KBP1/NR3C1/TGFB1/APOE/C3/STAR/LGALS1/CD55/A2M/VIM/ENG/CD59/ANXA5/APOA1/CTSL/ENAH/GSTP1/EMP2/GPX3/LAMB2/CDKN1A/CLU/KANK2/HMOX1 |
| DOID:0060071 | pre-malignant neoplasm | 28/860 | 169/10312 | 0.00033 | 0.00297 | 0.002026 | MMP2/PDPN/TIMP2/IGFBP6/RUNX3/S100A4/NFKBIA/ANXA1/ATM/SOCS1/BCL2/TGFB1/CCND2/MGP/SFRP1/CAV1/IGF1/PDGFRA/LRP1/GSTP1/INSR/CDKN1A/SOCS3/APEX1/CTNNB1/ADAM9/BNIP3/CTNNA1 |
| DOID:2526 | prostate adenocarcinoma | 12/860 | 47/10312 | 0.000355 | 0.003164 | 0.002158 | MMP2/ITGB1/PAM/STAT1/NFKBIA/JUN/PDGFRA/INSR/CAMK2N1/CD9/MYC/CLU |
| DOID:450 | myotonic disease | 8/860 | 23/10312 | 0.000357 | 0.003164 | 0.002158 | PDLIM3/MBNL1/CELF2/TPM3/HSPG2/INSR/MBNL2/DMD |
| DOID:2921 | glomerulonephritis | 36/860 | 240/10312 | 0.000381 | 0.003352 | 0.002287 | SPARC/COL3A1/COL1A1/FBN1/FN1/CFH/MMP2/ITGB1/PRF1/ALOX5AP/CCL3/JUND/TNFRSF1B/PFN1/NR3C1/RGS2/TGFB1/APOE/C7/C3/A2M/CD59/ANXA5/GAS6/MMP14/FGFR1/SGK1/VCAM1/NR4A1/LTBP1/CLU/TNFRSF1A/ACSM3/B4GALT1/AXL/LRPAP1 |
| DOID:1074 | kidney failure | 49/860 | 360/10312 | 0.000404 | 0.003523 | 0.002403 | COL3A1/COL1A1/SERPINF1/FN1/CFH/MMP2/PLAT/CCL5/B2M/ITGB2/ATXN7/CYBA/LPIN1/HSPA1A/CCL3/TNFRSF1B/ICAM3/ANXA1/NR3C1/UCP2/BCL2/TGFB1/APOE/C3/CST3/AKAP12/APOA1/IL6ST/GSTP1/LGALS3/INSR/VCAM1/NR4A1/ATP6AP2/COMT/HES1/MYC/CDKN1A/ALDH2/EPAS1/CLU/TNFRSF1A/PLAUR/PROS1/IRS1/AXL/BNIP3/HMOX1/NFE2L2 |
| DOID:3770 | pulmonary fibrosis | 31/860 | 198/10312 | 0.000452 | 0.00391 | 0.002667 | SPARC/COL1A2/COL1A1/TIMP1/AEBP1/MMP2/LMNA/CCL5/CXCR4/STAT1/MECP2/ANXA1/SOCS1/TGFB1/CFLAR/CAV1/COL18A1/IGF1/PDGFRA/TIMP3/APOA1/IL6ST/GSTP1/CXCL12/IGFBP5/SGK1/IGF2/EGR1/CTNNB1/HMOX1/NFE2L2 |
| DOID:0050622 | reproductive organ benign neoplasm | 14/860 | 62/10312 | 0.000466 | 0.003971 | 0.002709 | COL1A1/COL5A1/MMP2/LMNA/VCAN/LAMB1/TNRC6B/BCL2/TGFB1/IGF1/EGR1/COMT/CEBPB/CDKN1C |
| DOID:4971 | myelofibrosis | 14/860 | 62/10312 | 0.000466 | 0.003971 | 0.002709 | THBS1/FN1/BMP1/CXCR4/TGFB1/ENG/CALR/CXCL12/MMP14/NPM1/LDHB/LDHA/SOCS3/BNIP3 |
| DOID:1184 | nephrotic syndrome | 23/860 | 131/10312 | 0.000474 | 0.004009 | 0.002734 | IL1R1/CFI/CD247/NFKBIA/ITSN2/MAF/PSMB8/SH3KBP1/NR3C1/TGFB1/APOE/LGALS1/A2M/ANXA5/APOA1/CTSL/GSTP1/EMP2/GPX3/LAMB2/CDKN1A/CLU/KANK2 |
| DOID:12689 | acoustic neuroma | 8/860 | 24/10312 | 0.000497 | 0.004172 | 0.002846 | CD44/TGFB1/CAV1/IGF1/PDGFRA/FGFR1/CDKN1A/RAC1 |
| DOID:1967 | leiomyosarcoma | 10/860 | 36/10312 | 0.000528 | 0.00437 | 0.002981 | CALD1/MMP2/BCL2/LGALS1/GRN/LGALS3/FBLIM1/FSCN1/MYC/CTNNB1 |
| DOID:4230 | smooth muscle cancer | 10/860 | 36/10312 | 0.000528 | 0.00437 | 0.002981 | CALD1/MMP2/BCL2/LGALS1/GRN/LGALS3/FBLIM1/FSCN1/MYC/CTNNB1 |
| DOID:3526 | cerebral infarction | 31/860 | 200/10312 | 0.000539 | 0.004427 | 0.003019 | SERPINF1/PLAT/APP/ALOX5AP/NFKBIA/ANXA1/NR3C1/BCL2/TGFB1/APOE/SERPINE2/C3/CST3/SERPING1/MDK/A2M/VIM/PDGFRA/PAWR/PDGFRB/DYNLL1/EGR1/BNIP3L/LDLR/TNFRSF1A/PROS1/APEX1/BNIP3/HMOX1/NFE2L2/RAMP1 |
| DOID:2671 | transitional cell carcinoma | 17/860 | 85/10312 | 0.000552 | 0.004503 | 0.003071 | TIMP1/MMP2/TIMP2/CD44/REL/ANXA1/BCL2/CCND2/CAV1/APOA1/CTSB/MMP14/BSG/CD9/CTNNB1/CTSH/CTNNA1 |
| DOID:6432 | pulmonary hypertension | 33/860 | 218/10312 | 0.000558 | 0.004516 | 0.00308 | COL1A1/GJA1/CCDC80/CAVIN1/CCL5/CXCR4/HLA-A/HLA-B/EIF2AK2/ALOX5AP/CYBA/JUN/BCL2/TGFB1/APOE/COL4A1/CAV1/VIM/CXCL12/PDIA3/PTGIS/FGFR1/VCAM1/ID1/EGR1/HYAL2/MYC/CDKN1A/EPAS1/CLU/CTNND1/IL13RA1/HMOX1 |
| DOID:1686 | glaucoma | 27/860 | 167/10312 | 0.000621 | 0.004967 | 0.003388 | SPARC/FBN1/GJA1/LOXL1/CFH/COL8A1/TXNIP/TAP1/BCL2/TGFB1/LYST/CCND2/APOE/SFRP1/CAV1/BEX3/COL18A1/HSPA5/EEF2/CAV2/TXN/LTBP2/CDKN1A/CLU/LDLR/TNFRSF1A/MAP1LC3A |
| DOID:2621 | autonomic nervous system neoplasm | 51/860 | 386/10312 | 0.000627 | 0.004967 | 0.003388 | IGFBP2/MMP2/LGALS3BP/ITGB1/PDPN/APP/CXCR4/MALAT1/S100A4/NSD1/ALOX5AP/CD44/ARID1B/KLF2/ID2/ATM/JUN/DUSP1/BCL2/PIK3R1/LGALS1/CD55/IGF1/FHL2/MDK/VIM/HSPA5/ID3/GSTP1/CXCL12/MMP14/PAWR/IGFBP5/DHCR24/TGFBI/IGF2/ITM2B/KLF4/NME1/ATP1B1/ID1/EGR1/ASPH/COMT/MYC/CDKN1A/EPAS1/CLU/CTNNB1/CDKN1C/HMOX1 |
| DOID:769 | neuroblastoma | 51/860 | 386/10312 | 0.000627 | 0.004967 | 0.003388 | IGFBP2/MMP2/LGALS3BP/ITGB1/PDPN/APP/CXCR4/MALAT1/S100A4/NSD1/ALOX5AP/CD44/ARID1B/KLF2/ID2/ATM/JUN/DUSP1/BCL2/PIK3R1/LGALS1/CD55/IGF1/FHL2/MDK/VIM/HSPA5/ID3/GSTP1/CXCL12/MMP14/PAWR/IGFBP5/DHCR24/TGFBI/IGF2/ITM2B/KLF4/NME1/ATP1B1/ID1/EGR1/ASPH/COMT/MYC/CDKN1A/EPAS1/CLU/CTNNB1/CDKN1C/HMOX1 |
| DOID:0080524 | thyroid gland adenocarcinoma | 24/860 | 142/10312 | 0.000639 | 0.004995 | 0.003407 | TIMP1/THBS1/FN1/CXCR4/RUNX3/PSMB9/ZFP36L2/ATM/TGFB1/TPM3/KRT19/LGALS3/TXN/CAMK2N1/RPL15/RPL7/MYC/CDKN1A/PRDX1/NDUFC2/CTNNB1/CEBPB/DAD1/S100A6 |
| DOID:0080525 | differentiated thyroid gland carcinoma | 24/860 | 142/10312 | 0.000639 | 0.004995 | 0.003407 | TIMP1/THBS1/FN1/CXCR4/RUNX3/PSMB9/ZFP36L2/ATM/TGFB1/TPM3/KRT19/LGALS3/TXN/CAMK2N1/RPL15/RPL7/MYC/CDKN1A/PRDX1/NDUFC2/CTNNB1/CEBPB/DAD1/S100A6 |
| DOID:332 | amyotrophic lateral sclerosis | 33/860 | 220/10312 | 0.000658 | 0.005102 | 0.00348 | TIMP1/IGFBP2/SERPINF1/P4HB/MMP2/LOX/APP/LAMC1/EIF2AK2/SETX/BPTF/TNFRSF1B/RNF19A/PFN1/FUS/APOE/C3/CST3/IGF1/ENG/GRN/TIMP3/GSTP1/CTSB/LGALS3/RTN4/FGFR1/IGF2/TNFRSF1A/APEX1/PON2/VAPA/NFE2L2 |
| DOID:4251 | conjunctival disease | 10/860 | 37/10312 | 0.000671 | 0.005168 | 0.003525 | MMP2/CCL5/LCK/STAT1/IRF1/C3/GSTP1/CLU/LAMP2/SOCS3 |
| DOID:3451 | skin carcinoma | 16/860 | 79/10312 | 0.000689 | 0.005271 | 0.003595 | THBS1/MMP2/CCL5/RUNX3/DDX5/CD44/MCL1/CFLAR/IGF1/KRT19/CXCL12/MMP14/CDKN1A/ITGAV/CTNNB1/AXL |
| DOID:2945 | severe acute respiratory syndrome | 14/860 | 65/10312 | 0.000773 | 0.005875 | 0.004007 | IL1R1/PLAT/CCL5/CD3E/HLA-B/STAT1/MX1/IRF3/CCND3/ICAM3/TGFB1/IGF1/CD9/RAB31 |
| DOID:0060095 | uterine benign neoplasm | 13/860 | 58/10312 | 0.000794 | 0.005992 | 0.004087 | COL1A1/COL5A1/MMP2/VCAN/LAMB1/TNRC6B/BCL2/TGFB1/IGF1/EGR1/COMT/CEBPB/CDKN1C |
| DOID:349 | systemic mastocytosis | 10/860 | 39/10312 | 0.001051 | 0.007879 | 0.005375 | MMP2/CCL5/CXCR4/KMT2A/DUSP1/TGFB1/PDGFRA/KLF4/MYC/TNFRSF1A |
| DOID:10286 | prostate carcinoma | 28/860 | 182/10312 | 0.001104 | 0.008153 | 0.005561 | COL1A1/MMP2/ITGB1/PAM/TXNIP/HSPA1A/STAT1/NFKBIA/ATM/JUN/BCL2/CFLAR/MGP/CAV1/IGF1/VIM/PDGFRA/GSTP1/MMP14/INSR/CAMK2N1/CD9/NME1/EGR1/DLC1/COMT/MYC/CLU |
| DOID:3146 | lipid metabolism disorder | 28/860 | 182/10312 | 0.001104 | 0.008153 | 0.005561 | FN1/CCL5/ALOX5AP/TXNIP/LPIN1/LPIN2/IRF9/HSPA1B/TGFB1/APOE/C3/STAR/CD55/IGF1/APOC1/LRP1/CPE/APOA1/MMP14/DHCR24/FKBP1A/VCAM1/LDLR/POR/PLAUR/IRS1/PON2/HADHB |
| DOID:11723 | Duchenne muscular dystrophy | 11/860 | 46/10312 | 0.001122 | 0.008153 | 0.005561 | TIMP1/MMP2/TIMP2/GPC1/HLA-A/HLA-B/UTRN/NR3C1/HSPG2/CTNNAL1/DMD |
| DOID:350 | mastocytosis | 11/860 | 46/10312 | 0.001122 | 0.008153 | 0.005561 | MMP2/CCL5/CXCR4/KMT2A/DUSP1/BCL2/TGFB1/PDGFRA/KLF4/MYC/TNFRSF1A |
| DOID:11722 | myotonic dystrophy type 1 | 7/860 | 21/10312 | 0.001123 | 0.008153 | 0.005561 | PDLIM3/MBNL1/CELF2/TPM3/INSR/MBNL2/DMD |
| DOID:4606 | bile duct cancer | 31/860 | 209/10312 | 0.001142 | 0.008233 | 0.005616 | THBS1/MMP2/CXCR4/RUNX3/TNFAIP3/ANXA1/MCL1/HSP90AA1/DUSP1/BCL2/TGFB1/APOE/CAV1/VIM/ANXA2/KRT19/LAPTM4B/APOA1/CD151/GSTP1/CXCL12/PAWR/LGALS3/TJP1/ASPH/FSCN1/CDKN1A/SOCS3/LDLR/CTNND1/RHOC |
| DOID:4074 | pancreatic adenocarcinoma | 30/860 | 201/10312 | 0.001231 | 0.00882 | 0.006016 | SPARC/THBS1/PALLD/PLAT/FAP/CXCR4/GLIPR2/JAK1/JUN/BCL2/TGFB1/C3/CAV1/LGALS1/IGF1/QSOX1/CD59/AKAP12/EFEMP1/CD151/CALR/CXCL12/MMP14/NRP2/PEBP1/ITGA6/RAC1/SOCS3/AKR7A2/BTF3 |
| DOID:1168 | familial hyperlipidemia | 21/860 | 124/10312 | 0.001325 | 0.009434 | 0.006435 | FN1/CCL5/ALOX5AP/TXNIP/IRF9/HSPA1B/TGFB1/APOE/C3/CD55/IGF1/APOC1/LRP1/CPE/APOA1/MMP14/VCAM1/LDLR/PLAUR/IRS1/PON2 |
| DOID:1192 | peripheral nervous system neoplasm | 52/860 | 409/10312 | 0.001347 | 0.009531 | 0.006501 | IGFBP2/MMP2/LGALS3BP/ITGB1/PDPN/APP/CXCR4/MALAT1/S100A4/NSD1/ALOX5AP/CD44/ARID1B/KLF2/ID2/ATM/JUN/DUSP1/BCL2/PIK3R1/LGALS1/CD55/IGF1/FHL2/MDK/VIM/TNXB/HSPA5/ID3/GSTP1/CXCL12/MMP14/PAWR/IGFBP5/DHCR24/TGFBI/IGF2/ITM2B/KLF4/NME1/ATP1B1/ID1/EGR1/ASPH/COMT/MYC/CDKN1A/EPAS1/CLU/CTNNB1/CDKN1C/HMOX1 |
| DOID:0060058 | lymphoma | 26/860 | 167/10312 | 0.001373 | 0.009654 | 0.006585 | MMP2/CXCR4/PRF1/PSMB9/NFKBIA/RBL2/BRD2/REL/KMT2A/IRF7/ICAM3/ATM/JUN/BCL2/PIK3R1/CFLAR/LGALS1/IL6ST/CALR/GSTP1/GPX3/EPHX1/PLS3/DLC1/MYC/CLU |
| DOID:0060036 | intrinsic cardiomyopathy | 48/860 | 371/10312 | 0.00141 | 0.009855 | 0.006722 | GJA1/MMP2/TPM2/TNFRSF12A/LMNA/MYL9/PDLIM3/TPM1/TIMP2/YME1L1/AKAP9/MDM4/ORAI1/RBPJ/HLA-DPB1/CALM1/ICAM3/BCL2/TGFB1/APOE/CAV1/LAMA4/IGF1/NR2F2/TIMP3/CTSL/MMP14/SLC25A3/TXN/VCL/NEXN/RPL5/RPS5/RPS3A/SLC25A6/JAG1/YWHAE/LAMP2/CRYAB/MAP1LC3A/SLC25A5/COX7A1/RPS17/PDLIM5/DAAM1/CCT3/DMD/GNAQ |
| DOID:289 | endometriosis | 21/860 | 125/10312 | 0.001471 | 0.010214 | 0.006967 | SERPINF1/IL1R1/MMP2/CFI/LAMB1/ACTN1/LAMC1/CCL5/PTPN22/IL2RB/IL32/NFKBIA/TNFRSF1B/BCL2/S100A13/IGF1/MDK/MMP14/EGR1/CTNNB1/AXL |
| DOID:3211 | lysosomal storage disease | 45/860 | 346/10312 | 0.001768 | 0.012206 | 0.008325 | COL1A1/SERPINF1/RARRES2/LMNA/LUM/CCL5/RPS27/GNPTAB/KLF6/IDS/UCP2/JUN/SOCS1/TGFB1/APOE/STAR/IGF1/APOC1/GRN/PGRMC1/HSPA5/APOA1/PSAP/GSTP1/CTSB/NPC2/LGALS3/FGFR1/INSR/TXN/CTSF/VCAM1/ADAMTS5/ALDH2/LAMP2/LDLR/SCARB1/EXTL2/IRS1/PON2/HADHB/GNS/SEC63/HMOX1/NFE2L2 |
| DOID:0050700 | cardiomyopathy | 56/860 | 454/10312 | 0.001811 | 0.012392 | 0.008453 | SPARC/GJA1/MMP2/TPM2/TNFRSF12A/LMNA/MYL9/PDLIM3/TPM1/TIMP2/ITGB2/HLA-B/YME1L1/AKAP9/MDM4/ORAI1/RBPJ/HLA-DPB1/CALM1/ICAM3/BCL2/TGFB1/APOE/SPARCL1/CAV1/LAMA4/COL15A1/COL18A1/IGF1/NR2F2/LRP1/TIMP3/CTSL/MMP14/SLC25A3/TXN/VCL/NEXN/RPL5/RPS5/RPS3A/SLC25A6/JAG1/YWHAE/LAMP2/CRYAB/PDLIM7/MAP1LC3A/SLC25A5/COX7A1/RPS17/PDLIM5/DAAM1/CCT3/DMD/GNAQ |
| DOID:3355 | fibrosarcoma | 9/860 | 35/10312 | 0.001817 | 0.012392 | 0.008453 | COL1A1/CREB3L1/CTSK/FUS/PDGFRA/APOD/PDGFRB/CD9/TNFRSF1A |
| DOID:10591 | pre-eclampsia | 41/860 | 309/10312 | 0.001911 | 0.012952 | 0.008835 | TIMP1/CFH/MMP2/CFI/VCAN/DCN/HTRA1/BGN/CCL5/LNPEP/TNFRSF1B/TAP1/TGFB1/APOE/C3/LGALS1/CST3/IGF1/ENG/HTRA3/HSPA5/IL6ST/CALR/GSTP1/GPX3/IGFBP5/EPHX1/GADD45A/IGF2/VCAM1/COMT/PRCP/EPAS1/DDAH2/CLU/SOCS3/PLAUR/APEX1/CDKN1C/HMOX1/NFE2L2 |
| DOID:8466 | retinal degeneration | 42/860 | 319/10312 | 0.001965 | 0.013236 | 0.009029 | TIMP1/IGFBP2/SERPINF1/LOXL1/CFH/MMP2/CFI/HTRA1/TIMP2/HLA-B/ATXN7/OFD1/TRA2B/RORA/BCL2/APOE/C3/CFD/CST3/SERPING1/IGF1/GSTM3/FBLN5/GSN/EFEMP1/ANXA5/TIMP3/GSTP1/GPX3/PDGFRB/ATP6AP2/CANX/CDKN1A/CLU/SCARB1/CRYAB/FDXR/ADAM9/NFE2L2/ARL3/DMD/CTNNA1 |
| DOID:11870 | Pick's disease | 7/860 | 23/10312 | 0.002044 | 0.013636 | 0.009301 | APP/APOE/PRDX2/PRDX6/PRDX1/HNMT/PRDX3 |
| DOID:1247 | blood coagulation disease | 33/860 | 235/10312 | 0.002048 | 0.013636 | 0.009301 | THBS1/CFH/CFI/PLAT/LMAN1/ACTN1/VKORC1/CCL5/CXCR4/PRF1/FYB1/PTPN22/HLA-A/ITGB2/HLA-B/WIPF1/RAB27A/TGFB1/LYST/C3/IGF1/ANXA2/CALR/ST3GAL4/HES1/JAG1/LDHA/PROS1/MCFD2/CTNNB1/LRPAP1/HMOX1/GNAQ |
| DOID:11446 | sciatic neuropathy | 20/860 | 120/10312 | 0.002081 | 0.013774 | 0.009395 | SERPINF1/PLAT/PMP22/CCL5/NFKBIA/TNFRSF1B/JAK1/APOE/C3/CAV1/AKAP12/PDGFRA/LRP1/TIMP3/APOA1/PDGFRB/FGFR1/LAMP2/TNFRSF1A/HMOX1 |
| DOID:4897 | bile duct carcinoma | 30/860 | 208/10312 | 0.002122 | 0.013963 | 0.009524 | THBS1/MMP2/CXCR4/RUNX3/TNFAIP3/ANXA1/MCL1/HSP90AA1/DUSP1/BCL2/TGFB1/APOE/CAV1/VIM/ANXA2/KRT19/LAPTM4B/APOA1/CD151/GSTP1/CXCL12/PAWR/LGALS3/ASPH/FSCN1/CDKN1A/SOCS3/LDLR/CTNND1/RHOC |
| DOID:9799 | eye degenerative disease | 42/860 | 321/10312 | 0.002213 | 0.014398 | 0.009821 | TIMP1/IGFBP2/SERPINF1/LOXL1/CFH/MMP2/CFI/HTRA1/TIMP2/HLA-B/ATXN7/OFD1/TRA2B/RORA/BCL2/APOE/C3/CFD/CST3/SERPING1/IGF1/GSTM3/FBLN5/GSN/EFEMP1/ANXA5/TIMP3/GSTP1/GPX3/PDGFRB/ATP6AP2/CANX/CDKN1A/CLU/SCARB1/CRYAB/FDXR/ADAM9/NFE2L2/ARL3/DMD/CTNNA1 |
| DOID:9675 | pulmonary emphysema | 12/860 | 57/10312 | 0.002214 | 0.014398 | 0.009821 | FBN1/MMP2/NFKBIA/JUN/TGFB1/GSTM3/GSTP1/MMP14/EPHX1/EGR1/HMOX1/NFE2L2 |
| DOID:231 | motor neuron disease | 37/860 | 274/10312 | 0.002292 | 0.014818 | 0.010108 | TIMP1/IGFBP2/SERPINF1/P4HB/MMP2/LOX/APP/LAMC1/EIF2AK2/SETX/BPTF/TNFRSF1B/TRA2B/RNF19A/PFN1/FUS/APOE/C3/CST3/IGF1/HSPG2/ENG/GRN/TIMP3/GSTP1/CTSB/LGALS3/RTN4/FGFR1/PLS3/FKBP1A/IGF2/TNFRSF1A/APEX1/PON2/VAPA/NFE2L2 |
| DOID:2352 | hemochromatosis | 8/860 | 30/10312 | 0.002524 | 0.016132 | 0.011004 | B2M/HLA-A/HLA-B/APOE/FTL/FTH1/HMOX1/SLC40A1 |
| DOID:2876 | laryngeal squamous cell carcinoma | 8/860 | 30/10312 | 0.002524 | 0.016132 | 0.011004 | MMP2/CCND3/BCL2/CAV1/LGALS1/MYC/CDKN1A/FOXL2 |
| DOID:0111962 | combined immunodeficiency | 17/860 | 97/10312 | 0.002538 | 0.016132 | 0.011004 | PTPRC/CD3D/CORO1A/CD3E/IL2RG/RAC2/LCK/ITGB2/ITGAL/IVNS1ABP/ADAR/ARPC1B/MSN/TAPBP/TAP1/PIK3R1/MYC |
| DOID:2723 | dermatitis | 34/860 | 248/10312 | 0.002641 | 0.016689 | 0.011384 | SERPINH1/COL1A1/TIMP1/MMP2/CCL5/HLA-B/DOCK8/IL32/MYO9B/NFKBIA/BRD2/REL/TNFRSF14/TGFB1/APOE/C3/VIM/APOC1/ANXA2/APOA1/CD151/IL6ST/GSTP1/DST/ITGA6/CDKN1A/SOCS3/FABP5/PLAUR/ALDH1A1/HNMT/IL13RA1/HMOX1/ENO1 |
| DOID:0050938 | breast lobular carcinoma | 7/860 | 24/10312 | 0.00268 | 0.016752 | 0.011427 | CD44/HSP90AA1/CCND2/CTNND1/PLAUR/CTNNB1/CTNNA1 |
| DOID:3457 | invasive lobular carcinoma | 7/860 | 24/10312 | 0.00268 | 0.016752 | 0.011427 | CD44/HSP90AA1/CCND2/CTNND1/PLAUR/CTNNB1/CTNNA1 |
| DOID:657 | adenoma | 44/860 | 344/10312 | 0.002734 | 0.016995 | 0.011593 | THBS2/THBS1/MMP2/COL11A1/ITGB1/CXCR4/CD44/NCOR1/CCND3/JUN/SOCS1/BCL2/TGFB1/AKAP13/OGN/MEG3/CDH11/GSN/TCF4/TNXB/PDGFRA/KRT19/EFEMP1/CTSL/IL6ST/GSTP1/CTSB/GPX3/CXCL12/EPHX1/IGF2/SELENOP/NME1/FSCN1/MYC/CDKN1A/ALDH2/CLU/PRDX1/CTNNB1/SLC9A3R2/HMOX1/NFE2L2/CTNNA1 |
| DOID:4610 | intestinal benign neoplasm | 13/860 | 66/10312 | 0.002764 | 0.017078 | 0.011649 | FBLN1/LOX/JUN/BCL2/CCND2/GSTP1/GPX3/PMEPA1/PTGIS/SELENOP/KLF4/RAC1/TNFRSF1A |
| DOID:2600 | laryngeal carcinoma | 10/860 | 44/10312 | 0.002802 | 0.017078 | 0.011649 | MMP2/CXCR4/CCND3/SOCS1/BCL2/CAV1/LGALS1/MYC/CDKN1A/FOXL2 |
| DOID:12528 | lesion of sciatic nerve | 20/860 | 123/10312 | 0.002808 | 0.017078 | 0.011649 | SERPINF1/PLAT/PMP22/CCL5/NFKBIA/TNFRSF1B/JAK1/APOE/C3/CAV1/AKAP12/PDGFRA/LRP1/TIMP3/APOA1/PDGFRB/FGFR1/LAMP2/TNFRSF1A/HMOX1 |
| DOID:9473 | mononeuritis of lower limb | 20/860 | 123/10312 | 0.002808 | 0.017078 | 0.011649 | SERPINF1/PLAT/PMP22/CCL5/NFKBIA/TNFRSF1B/JAK1/APOE/C3/CAV1/AKAP12/PDGFRA/LRP1/TIMP3/APOA1/PDGFRB/FGFR1/LAMP2/TNFRSF1A/HMOX1 |
| DOID:767 | muscular atrophy | 15/860 | 82/10312 | 0.002902 | 0.0175 | 0.011937 | COL1A2/TPM2/LMNA/EIF2AK2/JUND/CBLB/TRA2B/NR3C1/TGFB1/IGF1/HSPG2/GSN/CTSL/PLS3/DMD |
| DOID:3144 | cutis laxa | 5/860 | 13/10312 | 0.002909 | 0.0175 | 0.011937 | EFEMP2/PYCR1/FBLN5/LTBP1/LTBP4 |
| DOID:8502 | bullous skin disease | 8/860 | 31/10312 | 0.003158 | 0.018801 | 0.012824 | SERPINH1/COL1A1/MYO9B/TGFB1/VIM/CD151/DST/ITGA6 |
| DOID:9452 | fatty liver disease | 32/860 | 232/10312 | 0.003159 | 0.018801 | 0.012824 | COL1A1/SERPINF1/RARRES2/LMNA/LUM/CCL5/KLF6/UCP2/JUN/SOCS1/TGFB1/APOE/STAR/IGF1/APOC1/PGRMC1/HSPA5/APOA1/GSTP1/CTSB/LGALS3/FGFR1/INSR/TXN/ALDH2/LDLR/IRS1/PON2/HADHB/SEC63/HMOX1/NFE2L2 |
| DOID:2730 | epidermolysis bullosa | 7/860 | 25/10312 | 0.003459 | 0.020481 | 0.01397 | SERPINH1/COL1A1/TGFB1/VIM/CD151/DST/ITGA6 |
| DOID:1206 | Rett syndrome | 6/860 | 19/10312 | 0.00348 | 0.0205 | 0.013983 | MECP2/ID2/APOE/ID3/ID4/ID1 |
| DOID:11612 | polycystic ovary syndrome | 27/860 | 188/10312 | 0.003667 | 0.021437 | 0.014622 | TIMP1/SERPINF1/MMP2/RARRES2/LMNA/PLAT/NCOR1/LPIN1/TNFRSF1B/NR3C1/BCL2/PIK3R1/C3/STAR/IGF1/APOC1/IL6ST/INSR/IGF2/COMT/SOCS3/ITGAV/PLAUR/IRS1/NFIC/HMOX1/GNAQ |
| DOID:13580 | cholestasis | 24/860 | 161/10312 | 0.00369 | 0.021437 | 0.014622 | COL1A1/TIMP1/CFH/MMP2/HLA-E/CD44/MX1/UCP2/TGFB1/APOE/LGALS1/IGF1/ENG/GSTP1/PDGFRB/ID1/TJP1/JAG1/LAMP2/SPINT2/ITGAV/ADH5/HMOX1/NFE2L2 |
| DOID:3587 | pancreatic ductal carcinoma | 14/860 | 76/10312 | 0.003697 | 0.021437 | 0.014622 | THBS1/MMP2/PALLD/TGFB1/APOC1/ANXA2/ANXA5/CALR/GSTP1/NME1/FSCN1/CDKN1A/CTNND1/ADAM9 |
| DOID:3910 | lung adenocarcinoma | 35/860 | 263/10312 | 0.003777 | 0.021789 | 0.014862 | CD63/PLOD2/HTRA1/ITGB1/LUM/COL10A1/CCL5/CXCR4/CD3E/RUNX3/IKZF1/S100A4/CD44/ETS1/KLF6/MCL1/PIK3R1/TGFB1/CCND2/CFLAR/APOE/CAV1/IL6ST/CALR/GSTP1/GPX3/CXCL12/LGALS3/EGR1/MYC/ITGAV/CTNNB1/AXL/HMOX1/STMN1 |
| DOID:4896 | bile duct adenocarcinoma | 28/860 | 198/10312 | 0.003902 | 0.022282 | 0.015198 | THBS1/MMP2/CXCR4/RUNX3/TNFAIP3/ANXA1/MCL1/HSP90AA1/DUSP1/BCL2/TGFB1/CAV1/VIM/ANXA2/KRT19/LAPTM4B/APOA1/CD151/GSTP1/CXCL12/PAWR/LGALS3/ASPH/FSCN1/CDKN1A/SOCS3/CTNND1/RHOC |
| DOID:4947 | cholangiocarcinoma | 28/860 | 198/10312 | 0.003902 | 0.022282 | 0.015198 | THBS1/MMP2/CXCR4/RUNX3/TNFAIP3/ANXA1/MCL1/HSP90AA1/DUSP1/BCL2/TGFB1/CAV1/VIM/ANXA2/KRT19/LAPTM4B/APOA1/CD151/GSTP1/CXCL12/PAWR/LGALS3/ASPH/FSCN1/CDKN1A/SOCS3/CTNND1/RHOC |
| DOID:4079 | heart valve disease | 17/860 | 101/10312 | 0.003923 | 0.022289 | 0.015204 | COL3A1/COL1A2/FBN1/IL1R1/MMP2/LMNA/VKORC1/CXCR4/HLA-B/OGT/RBPJ/SOCS1/APOE/IGF1/APOA1/LGALS3/EGR1 |
| DOID:3969 | thyroid gland papillary carcinoma | 20/860 | 127/10312 | 0.004099 | 0.023171 | 0.015805 | FN1/CXCR4/RUNX3/ZFP36L2/ATM/TPM3/KRT19/LGALS3/TXN/CAMK2N1/RPL15/RPL7/MYC/CDKN1A/PRDX1/NDUFC2/CTNNB1/CEBPB/DAD1/S100A6 |
| DOID:5200 | urinary tract obstruction | 13/860 | 69/10312 | 0.004143 | 0.023175 | 0.015807 | COL1A2/COL1A1/FN1/LUM/B2M/TNFRSF1B/IRF1/TGFB1/HSPA5/RTN4/ADAMTS1/BNC2/TNFRSF1A |
| DOID:12306 | vitiligo | 15/860 | 85/10312 | 0.004144 | 0.023175 | 0.015807 | PTPN22/HLA-A/HLA-B/HLA-C/HSPA1A/PSMB9/PSMB8/TAP1/TGFB1/SMOC2/PDGFRA/GSTP1/COMT/MSRB2/NFE2L2 |
| DOID:3021 | acute kidney failure | 18/860 | 110/10312 | 0.004161 | 0.023175 | 0.015807 | FN1/PLAT/B2M/HSPA1A/CCL3/TNFRSF1B/ANXA1/BCL2/IL6ST/GSTP1/LGALS3/HES1/EPAS1/CLU/TNFRSF1A/AXL/HMOX1/NFE2L2 |
| DOID:1414 | ovarian dysfunction | 28/860 | 199/10312 | 0.004191 | 0.023228 | 0.015844 | TIMP1/SERPINF1/MMP2/RARRES2/LMNA/PLAT/NCOR1/LPIN1/TNFRSF1B/NR3C1/BCL2/PIK3R1/C3/STAR/IGF1/APOC1/IL6ST/INSR/IGF2/COMT/SOCS3/ITGAV/PLAUR/FOXL2/IRS1/NFIC/HMOX1/GNAQ |
| DOID:0060082 | breast benign neoplasm | 5/860 | 14/10312 | 0.004218 | 0.023259 | 0.015865 | GJA1/CD44/IGF1/CALR/TXN |
| DOID:3717 | gastric adenocarcinoma | 19/860 | 119/10312 | 0.004348 | 0.023861 | 0.016276 | COL1A2/MMP2/TAGLN/CD44/ANXA1/IRF1/JUN/BCL2/TGFB1/LGALS1/PMEPA1/PEBP1/ID4/SELENOP/KLF4/MYC/APEX1/CTNNB1/HMOX1 |
| DOID:1339 | Diamond-Blackfan anemia | 7/860 | 26/10312 | 0.004399 | 0.023905 | 0.016306 | RPS29/RPS27/RPS7/RPL5/RPL15/RPS17/RPL18 |
| DOID:2731 | vesiculobullous skin disease | 7/860 | 26/10312 | 0.004399 | 0.023905 | 0.016306 | SERPINH1/COL1A1/TGFB1/VIM/CD151/DST/ITGA6 |
| DOID:403 | mouth disease | 35/860 | 266/10312 | 0.004536 | 0.024536 | 0.016736 | SPARC/COL1A2/CKAP4/COL1A1/TIMP1/FN1/IL1R1/MMP2/FMOD/PLAT/PDPN/APP/HSP90B1/CCL5/HLA-A/HLA-B/IL32/CD44/CYBA/ADAR/MSN/CTSC/ATM/BCL2/TGFB1/HSPG2/CXCL12/CYB5A/FGFR1/VCAM1/CDKN1A/ALDH2/TNFRSF1A/PDLIM7/CEBPB |
| DOID:10825 | essential hypertension | 21/860 | 137/10312 | 0.004588 | 0.024695 | 0.016844 | MMP2/RARRES2/PLAT/EMILIN1/BGN/CYBA/HSPA1A/PSMB9/HSPA1B/TGFB1/CST3/IGF1/GSTM3/PTGIS/INSR/ATP1B1/ALDH2/CLU/APEX1/ACSM3/HMOX1 |
| DOID:9455 | lipid storage disease | 39/860 | 305/10312 | 0.004657 | 0.024948 | 0.017017 | COL1A1/SERPINF1/RARRES2/LMNA/LUM/CCL5/GNPTAB/KLF6/UCP2/JUN/SOCS1/TGFB1/APOE/STAR/IGF1/APOC1/GRN/PGRMC1/HSPA5/APOA1/PSAP/GSTP1/CTSB/NPC2/LGALS3/FGFR1/INSR/TXN/CTSF/VCAM1/ALDH2/LDLR/SCARB1/IRS1/PON2/HADHB/SEC63/HMOX1/NFE2L2 |
| DOID:13141 | uveitis | 14/860 | 78/10312 | 0.004715 | 0.025139 | 0.017147 | SERPINF1/CFH/CCL5/PTPN22/HLA-B/CCL3/ANXA1/NR3C1/TGFB1/C3/CD59/IL6ST/TJP1/TNFRSF1A |
| DOID:10976 | membranous glomerulonephritis | 9/860 | 40/10312 | 0.004831 | 0.025635 | 0.017486 | SPARC/COL3A1/CFH/APOE/C7/C3/A2M/CLU/LRPAP1 |
| DOID:9744 | type 1 diabetes mellitus | 27/860 | 192/10312 | 0.004909 | 0.025928 | 0.017686 | TIMP1/SERPINF1/IL1R1/PLAT/TIMP2/CCL5/PTPRC/PRF1/PTPN22/DOCK8/HLA-DPB1/ETS1/CBLB/TAP1/DUSP1/TGFB1/APOE/C3/IGF1/APOA1/GPX3/CXCL12/LGALS3/IGF2/VCAM1/ALDH2/IGHG4 |
| DOID:9835 | refractive error | 15/860 | 87/10312 | 0.005191 | 0.02729 | 0.018615 | COL1A1/CFH/MMP2/P4HA2/LUM/CD247/APOBEC3G/LPIN2/BCL2/TGFB1/IGF1/EGR1/LAMA2/LDLR/LRPAP1 |
| DOID:2055 | post-traumatic stress disorder | 7/860 | 27/10312 | 0.005519 | 0.028744 | 0.019606 | NR3C1/BCL2/APOE/ANXA2/HSPA5/PDIA3/COMT |
| DOID:2566 | corneal dystrophy | 7/860 | 27/10312 | 0.005519 | 0.028744 | 0.019606 | DCN/TGFB1/C3/TCF4/TGFBI/CDKN1A/CLU |
| DOID:3498 | pancreatic ductal adenocarcinoma | 19/860 | 122/10312 | 0.005731 | 0.02971 | 0.020265 | PLAT/GLIPR2/JAK1/TGFB1/CAV1/LGALS1/QSOX1/CD59/AKAP12/CD151/CALR/MMP14/NRP2/PEBP1/ITGA6/RAC1/SOCS3/AKR7A2/BTF3 |
| DOID:1040 | chronic lymphocytic leukemia | 31/860 | 232/10312 | 0.005833 | 0.030101 | 0.020532 | FMOD/PLAT/ITGB1/BGN/CCL4/CXCR4/CD2/CD52/PTPN22/B2M/EIF2AK2/FMNL1/HCLS1/HSPA1A/STAT1/CCL3/TNFAIP3/MCL1/HSP90AA1/ATM/BCL2/TGFB1/SFRP1/COL18A1/GSTP1/CXCL12/NRP1/PEG10/MYC/NFE2L2/DMD |
| DOID:4007 | bladder carcinoma | 12/860 | 64/10312 | 0.006002 | 0.030833 | 0.021031 | CKAP4/CXCR4/ANXA1/TGFB1/CFLAR/CAV1/VIM/GSTP1/ANGPTL4/MYC/PLAUR/SMTN |
| DOID:4138 | bile duct disease | 27/860 | 195/10312 | 0.006056 | 0.030969 | 0.021124 | COL1A1/TIMP1/CFH/MMP2/CCL5/HLA-E/HLA-C/CD44/MX1/UCP2/ATM/TGFB1/APOE/LGALS1/IGF1/ENG/GSTP1/PDGFRB/ID1/TJP1/JAG1/LAMP2/SPINT2/ITGAV/ADH5/HMOX1/NFE2L2 |
| DOID:0050557 | congenital muscular dystrophy | 7/860 | 28/10312 | 0.00684 | 0.034817 | 0.023749 | COL6A3/COL6A2/COL6A1/LMNA/COL12A1/COL4A1/LAMA2 |
| DOID:9741 | biliary tract disease | 27/860 | 197/10312 | 0.006938 | 0.03516 | 0.023983 | COL1A1/TIMP1/CFH/MMP2/CCL5/HLA-E/HLA-C/CD44/MX1/UCP2/ATM/TGFB1/APOE/LGALS1/IGF1/ENG/GSTP1/PDGFRB/ID1/TJP1/JAG1/LAMP2/SPINT2/ITGAV/ADH5/HMOX1/NFE2L2 |
| DOID:0050621 | respiratory system benign neoplasm | 14/860 | 82/10312 | 0.007429 | 0.037477 | 0.025563 | PLAT/ITGB1/RBL2/ANXA1/MCL1/BCL2/ANXA2/TM4SF1/MMP14/RHOB/TUBA1A/CDKN1A/YAP1/PLAUR |
| DOID:0060056 | hypersensitivity reaction disease | 22/860 | 152/10312 | 0.007515 | 0.03774 | 0.025743 | COL3A1/COL1A1/TIMP1/CFH/PLAT/CCL5/PTPN22/HLA-B/CYBA/STAT1/NFKBIA/CCL3/TNFRSF1B/NR3C1/TGFB1/C3/CFD/IGF1/IGKC/EGR1/CDKN1A/HMOX1 |
| DOID:5199 | ureteral obstruction | 11/860 | 58/10312 | 0.007651 | 0.038253 | 0.026092 | COL1A2/COL1A1/FN1/LUM/B2M/TNFRSF1B/IRF1/HSPA5/RTN4/ADAMTS1/TNFRSF1A |
| DOID:0060097 | thoracic benign neoplasm | 5/860 | 16/10312 | 0.007998 | 0.039815 | 0.027158 | GJA1/CD44/IGF1/CALR/TXN |
| DOID:1993 | rectum cancer | 7/860 | 29/10312 | 0.008381 | 0.040995 | 0.027963 | CXCR4/ADGRE5/BCL2/APOE/CD55/GPX3/CXCL12 |
| DOID:3151 | skin squamous cell carcinoma | 7/860 | 29/10312 | 0.008381 | 0.040995 | 0.027963 | THBS1/MMP2/DDX5/CD44/MMP14/CTNNB1/AXL |
| DOID:4798 | aggressive systemic mastocytosis | 7/860 | 29/10312 | 0.008381 | 0.040995 | 0.027963 | MMP2/CCL5/CXCR4/DUSP1/TGFB1/KLF4/TNFRSF1A |
| DOID:6195 | conjunctivitis | 7/860 | 29/10312 | 0.008381 | 0.040995 | 0.027963 | MMP2/CCL5/LCK/IRF1/C3/LAMP2/SOCS3 |
| DOID:8791 | breast carcinoma in situ | 10/860 | 51/10312 | 0.008493 | 0.041363 | 0.028214 | MMP2/TIMP2/ANXA1/SOCS1/BCL2/CCND2/CAV1/CDKN1A/SOCS3/BNIP3 |
| DOID:272 | hepatic vascular disease | 12/860 | 67/10312 | 0.008711 | 0.042239 | 0.028811 | TIMP1/SERPINF1/VKORC1/TIMP2/GRK2/HSP90AA1/DUSP1/PIK3R1/TGFB1/APOA1/CTNNB1/HMOX1 |
| DOID:1802 | mononeuritis | 20/860 | 136/10312 | 0.008846 | 0.042529 | 0.029009 | SERPINF1/PLAT/PMP22/CCL5/NFKBIA/TNFRSF1B/JAK1/APOE/C3/CAV1/AKAP12/PDGFRA/LRP1/TIMP3/APOA1/PDGFRB/FGFR1/LAMP2/TNFRSF1A/HMOX1 |
| DOID:2871 | endometrial carcinoma | 20/860 | 136/10312 | 0.008846 | 0.042529 | 0.029009 | SPARC/TIMP1/MMP2/TIMP2/RUNX3/S100A4/CCND3/IRF1/ATM/JUN/BCL2/PIK3R1/CFLAR/CD55/MDK/GPX3/MMP14/BSG/MYC/PLAUR |
| DOID:1307 | dementia | 27/860 | 201/10312 | 0.009023 | 0.043193 | 0.029462 | TIMP1/THBS1/MMP2/APP/CXCR4/GLS/HSPA1A/TNFRSF1B/NR4A2/FUS/BCL2/TGFB1/APOE/CST3/IGF1/GRN/ANXA5/APOA1/FGFR1/IGF2/ITM2B/COMT/TNFRSF1A/PLAUR/MAP1LC3A/PON2/LRPAP1 |
| DOID:0060116 | sensory system cancer | 22/860 | 155/10312 | 0.009412 | 0.044678 | 0.030475 | SERPINF1/MMP2/LMNA/HLA-B/MDM4/RBL2/HSP90AA1/ATM/BCL2/CDH11/IGF1/AKAP12/IGFBP5/NME1/ID1/MYC/CDKN1A/CLU/GPC6/IRS1/CDKN1C/AXL |
| DOID:2174 | ocular cancer | 22/860 | 155/10312 | 0.009412 | 0.044678 | 0.030475 | SERPINF1/MMP2/LMNA/HLA-B/MDM4/RBL2/HSP90AA1/ATM/BCL2/CDH11/IGF1/AKAP12/IGFBP5/NME1/ID1/MYC/CDKN1A/CLU/GPC6/IRS1/CDKN1C/AXL |
| DOID:1188 | mononeuropathy | 20/860 | 137/10312 | 0.009574 | 0.04469 | 0.030483 | SERPINF1/PLAT/PMP22/CCL5/NFKBIA/TNFRSF1B/JAK1/APOE/C3/CAV1/AKAP12/PDGFRA/LRP1/TIMP3/APOA1/PDGFRB/FGFR1/LAMP2/TNFRSF1A/HMOX1 |
| DOID:3744 | cervical squamous cell carcinoma | 15/860 | 93/10312 | 0.009667 | 0.04469 | 0.030483 | MMP2/PDPN/TIMP2/ADAR/IFITM1/IFI16/BCL2/CFLAR/IFI27/IL6ST/MYC/CDKN1A/RAC1/SOCS3/CEBPB |
| DOID:0050860 | colorectal adenoma | 6/860 | 23/10312 | 0.009716 | 0.04469 | 0.030483 | JUN/GSTP1/GPX3/PTGIS/SELENOP/TNFRSF1A |
| DOID:3008 | invasive ductal carcinoma | 10/860 | 52/10312 | 0.009752 | 0.04469 | 0.030483 | CCL5/RUNX3/CD44/CCND3/ANXA1/SOCS1/CAV1/ANXA2/CLU/SOCS3 |
| DOID:3907 | lung squamous cell carcinoma | 12/860 | 68/10312 | 0.009799 | 0.04469 | 0.030483 | PDPN/FAP/S100A4/CBLB/ATM/CCND2/PEBP1/BSG/DDR2/RPS3A/CDKN1A/CTNNB1 |
| DOID:0060443 | corneal endothelial dystrophy | 4/860 | 11/10312 | 0.009847 | 0.04469 | 0.030483 | C3/TCF4/CDKN1A/CLU |
| DOID:10966 | lipoid nephrosis | 4/860 | 11/10312 | 0.009847 | 0.04469 | 0.030483 | MAF/NR3C1/LGALS1/CDKN1A |
| DOID:11555 | Fuchs' endothelial dystrophy | 4/860 | 11/10312 | 0.009847 | 0.04469 | 0.030483 | C3/TCF4/CDKN1A/CLU |
| DOID:3507 | dermatofibrosarcoma protuberans | 4/860 | 11/10312 | 0.009847 | 0.04469 | 0.030483 | COL1A1/CTSK/APOD/PDGFRB |
| DOID:4226 | endometrial stromal sarcoma | 4/860 | 11/10312 | 0.009847 | 0.04469 | 0.030483 | MALAT1/PDGFRA/PDGFRB/EPAS1 |
| DOID:11260 | rabies | 11/860 | 60/10312 | 0.009891 | 0.04469 | 0.030483 | APP/VKORC1/IGFBP6/STAT1/IRF3/IRF7/BCL2/CAV1/RNASE1/CALR/PLIN3 |
| DOID:11830 | myopia | 11/860 | 60/10312 | 0.009891 | 0.04469 | 0.030483 | COL1A1/CFH/MMP2/P4HA2/LUM/LPIN2/TGFB1/IGF1/EGR1/LAMA2/LRPAP1 |
| DOID:8618 | oral cavity cancer | 20/860 | 138/10312 | 0.010349 | 0.046572 | 0.031767 | MMP2/CXCR4/GON4L/ARPC1B/ATM/BCL2/CALR/GSTP1/SOX4/LGALS3/CTNNAL1/PTGIS/DDR2/CAMK2N1/ID1/CDKN1A/BST2/MTUS1/CTNND1/CTNNB1 |
| DOID:14323 | Marfan syndrome | 5/860 | 17/10312 | 0.010567 | 0.046987 | 0.03205 | FBN1/MMP2/TIMP2/TGFB1/MMP14 |
| DOID:5166 | endometrial stromal tumor | 5/860 | 17/10312 | 0.010567 | 0.046987 | 0.03205 | MALAT1/PDGFRA/PDGFRB/EPAS1/CTNNB1 |
| DOID:9146 | visceral leishmaniasis | 5/860 | 17/10312 | 0.010567 | 0.046987 | 0.03205 | CCL4/IL2RB/IL32/HMOX1/NFE2L2 |
| DOID:255 | hemangioma | 9/860 | 45/10312 | 0.010768 | 0.047508 | 0.032405 | MMP2/ENG/NRP1/PDGFRB/IGF2/TUBA1A/EPAS1/CLU/CTNNB1 |
| DOID:3192 | neurilemmoma | 9/860 | 45/10312 | 0.010768 | 0.047508 | 0.032405 | CD44/TGFB1/CAV1/IGF1/PDGFRA/PDGFRB/FGFR1/CDKN1A/RAC1 |
| DOID:0050589 | inflammatory bowel disease | 33/860 | 262/10312 | 0.010912 | 0.047954 | 0.03271 | COL1A1/CCL5/CXCR4/PRF1/RUNX3/PTPN22/B2M/ITGB2/HLA-B/ITGAL/TXNIP/IL10RA/MYO9B/TNFAIP3/TNFRSF1B/CARD8/ANXA1/HSPA1B/TGFB1/COL18A1/HSPA5/IGF2/VCAM1/RHOB/CDKN1A/EPB41L2/SOCS3/TNFRSF1A/PLAUR/ACSM3/HNMT/RHOC/HMOX1 |
| DOID:9743 | diabetic neuropathy | 11/860 | 61/10312 | 0.011186 | 0.048715 | 0.033229 | CD63/PLAT/CCL5/MBP/UCP2/TGFB1/IGF1/INSR/TXN/VCAM1/ALDH2 |
| DOID:75 | lymphatic system disease | 18/860 | 121/10312 | 0.011238 | 0.048715 | 0.033229 | TIMP1/PRF1/HLA-E/HLA-A/HLA-B/HLA-C/ITGAL/HSPA1A/RAB27A/SOCS1/PIK3R1/TGFB1/CFLAR/ACTA2/LGMN/CLU/SOCS3/HMOX1 |
| DOID:2957 | pulmonary tuberculosis | 14/860 | 86/10312 | 0.011259 | 0.048715 | 0.033229 | CCL5/CCL4/CD247/PTPN22/IL32/CYBA/NFKBIA/CCL3/HLA-DPB1/TAP1/JUN/C3/ATF3/PLAUR |
| DOID:9261 | nasopharynx carcinoma | 14/860 | 86/10312 | 0.011259 | 0.048715 | 0.033229 | MMP2/CXCR4/RBL2/TNFAIP3/HSPA1B/ATM/BCL2/CCND2/CALR/PAWR/DDR2/MYC/CDKN1A/BCAT1 |
| DOID:5327 | retinal detachment | 6/860 | 24/10312 | 0.012061 | 0.051931 | 0.035422 | CFH/IL10RA/CXCL12/FGFR1/MAP1LC3A/BNIP3 |
| DOID:13241 | Behcet's disease | 17/860 | 113/10312 | 0.01214 | 0.051931 | 0.035422 | MMP2/LGALS3BP/PTPN22/HLA-E/HLA-A/HLA-B/NFKBIA/TNFRSF1B/IRF1/TGFB1/VIM/GSTP1/CXCL12/TNFRSF1A/PROS1/HMOX1/ENO1 |
| DOID:633 | myositis | 17/860 | 113/10312 | 0.01214 | 0.051931 | 0.035422 | THBS1/MMP2/FMOD/LMNA/APP/HLA-E/HLA-A/HLA-B/HLA-C/HLA-F/TNFRSF1B/APOE/CST3/SDC2/CLU/TNFRSF1A/MAP1LC3A |
| DOID:3429 | inclusion body myositis | 7/860 | 31/10312 | 0.012207 | 0.051972 | 0.035451 | THBS1/LMNA/APP/APOE/CST3/CLU/MAP1LC3A |
| DOID:1602 | lymphadenitis | 12/860 | 70/10312 | 0.012289 | 0.051972 | 0.035451 | TIMP1/PRF1/HLA-E/HLA-A/HLA-B/HLA-C/HSPA1A/SOCS1/TGFB1/CLU/SOCS3/HMOX1 |
| DOID:9942 | lymph node disease | 12/860 | 70/10312 | 0.012289 | 0.051972 | 0.035451 | TIMP1/PRF1/HLA-E/HLA-A/HLA-B/HLA-C/HSPA1A/SOCS1/TGFB1/CLU/SOCS3/HMOX1 |
| DOID:12930 | dilated cardiomyopathy | 30/860 | 235/10312 | 0.012359 | 0.052074 | 0.03552 | GJA1/MMP2/TPM2/TNFRSF12A/LMNA/TPM1/YME1L1/MDM4/ORAI1/RBPJ/ICAM3/BCL2/TGFB1/APOE/LAMA4/IGF1/NR2F2/TIMP3/CTSL/MMP14/TXN/VCL/NEXN/JAG1/LAMP2/CRYAB/COX7A1/PDLIM5/DMD/GNAQ |
| DOID:3480 | uveal disease | 14/860 | 87/10312 | 0.012422 | 0.052146 | 0.035569 | SERPINF1/CFH/CCL5/PTPN22/HLA-B/CCL3/ANXA1/NR3C1/TGFB1/C3/CD59/IL6ST/TJP1/TNFRSF1A |
| DOID:4914 | esophagus adenocarcinoma | 10/860 | 54/10312 | 0.01269 | 0.05307 | 0.036199 | MMP2/S100A4/BCL2/TGFB1/SFRP1/HSPA5/CTSB/GPX3/MMP14/MYC |
| DOID:2789 | parasitic protozoa infectious disease | 24/860 | 178/10312 | 0.012741 | 0.053086 | 0.03621 | COL1A1/MMP2/LMAN1/CCL5/CCL4/PTPN22/IL2RB/HLA-B/IL32/NFKBIA/CCL3/TNFRSF1B/IRF1/TGFB1/APOE/COL18A1/A2M/VIM/ENG/GSTP1/CTSB/TNFRSF1A/HMOX1/NFE2L2 |
| DOID:2320 | obstructive lung disease | 37/860 | 305/10312 | 0.013029 | 0.054087 | 0.036893 | COL1A1/FBN1/TIMP1/CD63/NNMT/MMP2/CCL5/CCL4/GZMA/ITGB2/ITGAL/CYBA/IL10RA/STAT1/NFKBIA/HLA-DPB1/TNFRSF1B/NR3C1/HSP90AA1/JUN/BCL2/TGFB1/SERPINE2/C3/GSTM3/A2M/GSTP1/MMP14/EPHX1/VCAM1/EGR1/TNFRSF1A/PLAUR/IL13RA1/BNIP3/HMOX1/NFE2L2 |
| DOID:0060060 | non-Hodgkin lymphoma | 17/860 | 114/10312 | 0.013198 | 0.054229 | 0.03699 | CXCR4/PRF1/NFKBIA/RBL2/BRD2/KMT2A/ICAM3/BCL2/LGALS1/IL6ST/CALR/GSTP1/GPX3/EPHX1/PLS3/MYC/CLU |
| DOID:341 | peripheral vascular disease | 17/860 | 114/10312 | 0.013198 | 0.054229 | 0.03699 | SPARC/SERPINF1/FN1/MMP2/PLAT/VKORC1/ATM/BCL2/CST3/IGF1/ENG/PLTP/CXCL12/MMP14/NRP1/VCAM1/CDKN1A |
| DOID:1824 | status epilepticus | 18/860 | 123/10312 | 0.013208 | 0.054229 | 0.03699 | PLAT/CCL4/HSPA1A/CCL3/NR3C1/ATM/SOCS1/BCL2/GRN/IL6ST/CXCL12/APOD/SOCS3/PLAUR/APEX1/ADAM9/HMOX1/DMD |
| DOID:200 | benign giant cell tumor | 5/860 | 18/10312 | 0.013646 | 0.055826 | 0.03808 | COL6A3/BSG/CLU/CTNNB1/CEBPB |
| DOID:11200 | T cell deficiency | 4/860 | 12/10312 | 0.01381 | 0.056289 | 0.038395 | TRAC/CD3G/CD247/ORAI1 |
| DOID:381 | arthropathy | 15/860 | 97/10312 | 0.014049 | 0.056911 | 0.038819 | SPARC/COL6A1/MMP2/RGS1/HLA-E/HLA-A/HLA-B/HLA-C/SAMHD1/NFKBIA/TNFRSF1B/HSPA5/PTGIS/TNFRSF1A/FTL |
| DOID:12449 | aplastic anemia | 19/860 | 133/10312 | 0.014164 | 0.056911 | 0.038819 | PRF1/RPS29/HLA-A/HLA-B/RPS27/MSN/HSP90AA1/ATM/TGFB1/CD55/CD59/CALR/GSTP1/RPS7/EPHX1/RPL5/RPL15/RPS17/RPL18 |
| DOID:0060074 | ductal carcinoma in situ | 9/860 | 47/10312 | 0.014266 | 0.056911 | 0.038819 | MMP2/TIMP2/ANXA1/SOCS1/BCL2/CCND2/CDKN1A/SOCS3/BNIP3 |
| DOID:0060123 | connective tissue benign neoplasm | 9/860 | 47/10312 | 0.014266 | 0.056911 | 0.038819 | SERPINH1/COL6A3/CTSK/HLA-A/PDGFRB/BSG/CLU/CTNNB1/CEBPB |
| DOID:2001 | neuroma | 9/860 | 47/10312 | 0.014266 | 0.056911 | 0.038819 | CD44/TGFB1/CAV1/IGF1/PDGFRA/PDGFRB/FGFR1/CDKN1A/RAC1 |
| DOID:437 | myasthenia gravis | 9/860 | 47/10312 | 0.014266 | 0.056911 | 0.038819 | PTPRC/PTPN22/HLA-E/IL2RB/NR3C1/TGFB1/C3/LGALS1/IL6ST |
| DOID:5183 | hereditary Wilms' tumor | 10/860 | 55/10312 | 0.014387 | 0.057156 | 0.038987 | HLA-A/HSP90AA1/IFI16/BCL2/PAWR/IGF2/EGR1/BASP1/CDKN1A/CTNNB1 |
| DOID:0080162 | lupus nephritis | 7/860 | 32/10312 | 0.01453 | 0.057156 | 0.038987 | COL1A1/CFH/TNFRSF1B/NR3C1/C3/VCAM1/TNFRSF1A |
| DOID:10588 | adrenoleukodystrophy | 7/860 | 32/10312 | 0.01453 | 0.057156 | 0.038987 | TIMP1/MMP2/PLAT/TNFRSF1B/ANXA1/ANXA2/TJP1 |
| DOID:9065 | leishmaniasis | 7/860 | 32/10312 | 0.01453 | 0.057156 | 0.038987 | COL1A1/MMP2/CCL4/IL2RB/IL32/HMOX1/NFE2L2 |
| DOID:12842 | Guillain-Barre syndrome | 6/860 | 25/10312 | 0.014777 | 0.057925 | 0.039511 | MMP2/PMP22/NR3C1/APOE/CST3/CTSB |
| DOID:11335 | sarcoidosis | 17/860 | 116/10312 | 0.01553 | 0.060664 | 0.041379 | COL3A1/COL1A1/CFH/CCL5/HLA-B/CYBA/STAT1/NFKBIA/CCL3/TNFRSF1B/NR3C1/TGFB1/C3/IGKC/EGR1/CDKN1A/HMOX1 |
| DOID:0050339 | commensal bacterial infectious disease | 13/860 | 81/10312 | 0.015991 | 0.062033 | 0.042313 | TIMP1/SERPINF1/CFH/ITGB2/HLA-B/STAT1/NFKBIA/HSPA1B/GRK2/TGFB1/SERPING1/A2M/HMOX1 |
| DOID:3247 | rhabdomyosarcoma | 13/860 | 81/10312 | 0.015991 | 0.062033 | 0.042313 | IGFBP6/CXCR4/MCL1/ATM/TGFB1/PDGFRA/CXCL12/FGFR1/IGF2/CDKN1A/APEX1/CTNNB1/DMD |
| DOID:10762 | portal hypertension | 10/860 | 56/10312 | 0.016248 | 0.062797 | 0.042834 | TIMP1/SERPINF1/TIMP2/GRK2/HSP90AA1/DUSP1/PIK3R1/TGFB1/CTNNB1/HMOX1 |
| DOID:0060091 | cardiovascular organ benign neoplasm | 9/860 | 48/10312 | 0.016299 | 0.062797 | 0.042834 | MMP2/ENG/NRP1/PDGFRB/IGF2/TUBA1A/EPAS1/CLU/CTNNB1 |
| DOID:865 | vasculitis | 21/860 | 154/10312 | 0.01687 | 0.064772 | 0.044181 | MMP2/LGALS3BP/PTPN22/HLA-E/HLA-A/HLA-B/NFKBIA/MX1/TNFAIP3/TNFRSF1B/IRF1/TGFB1/VIM/GSTP1/CXCL12/MFGE8/JAG1/TNFRSF1A/PROS1/HMOX1/ENO1 |
| DOID:12030 | panuveitis | 7/860 | 33/10312 | 0.017153 | 0.065413 | 0.044619 | CFH/CCL5/PTPN22/HLA-B/CCL3/C3/CD59 |
| DOID:12351 | alcoholic hepatitis | 7/860 | 33/10312 | 0.017153 | 0.065413 | 0.044619 | COL1A1/CCL5/NFKBIA/CCL3/TNFRSF1B/SOCS1/TNFRSF1A |
| DOID:0060094 | bone benign neoplasm | 5/860 | 19/10312 | 0.017277 | 0.065444 | 0.04464 | COL6A3/BSG/CLU/CTNNB1/CEBPB |
| DOID:90 | degenerative disc disease | 5/860 | 19/10312 | 0.017277 | 0.065444 | 0.04464 | COL1A1/TIMP1/COL10A1/ADAMTS5/MAP1LC3A |
| DOID:1100 | ovarian disease | 32/860 | 261/10312 | 0.017353 | 0.065512 | 0.044686 | TIMP1/SERPINF1/MMP2/RARRES2/LMNA/PLAT/NCOR1/LPIN1/TNFRSF1B/NR3C1/BCL2/PIK3R1/C3/STAR/IGF1/APOC1/PGRMC1/IL6ST/GSTP1/CTSB/CXCL12/INSR/IGF2/COMT/SOCS3/ITGAV/PLAUR/FOXL2/IRS1/NFIC/HMOX1/GNAQ |
| DOID:1485 | cystic fibrosis | 24/860 | 183/10312 | 0.017468 | 0.065726 | 0.044832 | COL1A1/TIMP1/CCL4/SLC4A7/KLF2/HSPA1A/STAT1/CCL3/FXYD5/TAP1/NR3C1/TGFB1/CSK/IGF1/GSTM3/ANXA5/GSTP1/TXN/RACK1/NME1/CANX/TNFRSF1A/HMOX1/NFE2L2 |
| DOID:0060033 | autoimmune disease of peripheral nervous system | 6/860 | 26/10312 | 0.017891 | 0.066868 | 0.045611 | MMP2/PMP22/NR3C1/APOE/CST3/CTSB |
| DOID:1407 | anterior uveitis | 6/860 | 26/10312 | 0.017891 | 0.066868 | 0.045611 | CFH/PTPN22/HLA-B/CCL3/C3/CD59 |
| DOID:3525 | middle cerebral artery infarction | 18/860 | 127/10312 | 0.017955 | 0.066884 | 0.045622 | SERPINF1/PLAT/APP/NFKBIA/BCL2/TGFB1/APOE/C3/PDGFRA/PAWR/PDGFRB/DYNLL1/EGR1/BNIP3L/TNFRSF1A/BNIP3/HMOX1/NFE2L2 |
| DOID:1459 | hypothyroidism | 20/860 | 146/10312 | 0.01852 | 0.068762 | 0.046903 | COL1A2/IGFBP2/PLOD2/PAM/PTPN22/NCOR1/MBP/ANXA1/UCP2/APOE/STAR/CAV1/IGF1/GSN/ANXA2/ANXA5/MAP1B/PLOD1/EGR1/LRPAP1 |
| DOID:0060318 | acute promyelocytic leukemia | 4/860 | 13/10312 | 0.018654 | 0.069033 | 0.047088 | PLAT/SENP7/KMT2E/MYC |
| DOID:1176 | bronchial disease | 40/860 | 344/10312 | 0.019409 | 0.071592 | 0.048833 | MMP2/DCN/CCL5/CCL4/PRF1/RUNX3/HLA-E/IL2RB/HLA-B/HLA-C/EIF2AK2/IL32/ALOX5AP/CYBA/PRMT2/HSPA1A/STAT1/NFKBIA/HLA-DPB1/MAF/PPP1R15A/TNFRSF14/IRF1/HSPA1B/TAP1/NR3C1/BCL2/TGFB1/MYLK/C3/GSTM3/GSTP1/EPHX1/LGALS3/EGR1/ADH5/HNMT/IL13RA1/HMOX1/NFE2L2 |
| DOID:234 | colon adenocarcinoma | 11/860 | 66/10312 | 0.019686 | 0.07222 | 0.049262 | LOX/PMP22/BCL2/TGFB1/CAV1/APOA1/CALR/NRP1/ITGA6/RAC1/CTNNB1 |
| DOID:4989 | pancreatitis | 24/860 | 185/10312 | 0.019708 | 0.07222 | 0.049262 | MMP2/CCL5/CD44/NFKBIA/IRF9/HSPA1B/DUSP1/TGFB1/SERPING1/A2M/ENG/CD59/CTSL/GAS6/GSTP1/CTSB/FDX1/VCAM1/EGR1/BNIP3L/ALDH2/LAMP2/TNFRSF1A/PROS1 |
| DOID:3083 | chronic obstructive pulmonary disease | 36/860 | 304/10312 | 0.019965 | 0.072925 | 0.049743 | COL1A1/FBN1/TIMP1/CD63/NNMT/MMP2/CCL5/CCL4/GZMA/ITGB2/ITGAL/CYBA/IL10RA/STAT1/NFKBIA/HLA-DPB1/TNFRSF1B/NR3C1/HSP90AA1/JUN/BCL2/TGFB1/SERPINE2/C3/GSTM3/GSTP1/MMP14/EPHX1/VCAM1/EGR1/TNFRSF1A/PLAUR/IL13RA1/BNIP3/HMOX1/NFE2L2 |
