# Supplementary material for: Glycosylation-related genes mediated prognostic signature contribute to prognostic prediction and treatment options in ovarian cancer: based on bulk and single‑cell RNA sequencing data
Source: BMC Cancer. 2024 Feb 14;24:207. doi: 10.1186/s12885-024-11908-4 (PMC10865697; doi:10.1186/s12885-024-11908-4)
Supplement: Supplementary file 11 — Supplementary Table 6. 16 genes in the GRGs signature. [file 12885_2024_11908_MOESM11_ESM.docx]

Supplementary Table 6. 16 genes in the GRGs signature.

|  | Gene | Coef | Type |
| --- | --- | --- | --- |
| 1 | IGFBP7 | -0.26114 | Protective |
| 2 | GBP5 | -0.2807 | Protective |
| 3 | PPP4R3A | -0.46842 | Protective |
| 4 | ARID1B | 0.53432 | Risk |
| 5 | GSTK1 | -0.25829 | Protective |
| 6 | ARL6IP5 | -0.383 | Protective |
| 7 | DDIT4 | 0.103247 | Risk |
| 8 | BTN3A1 | -0.22202 | Protective |
| 9 | TPM3 | -0.44702 | Protective |
| 10 | MAGED2 | -0.37166 | Protective |
| 11 | ANGPTL4 | 0.117919 | Risk |
| 12 | NSG1 | -0.22049 | Protective |
| 13 | RAB34 | 0.224858 | Risk |
| 14 | GAS1 | 0.233199 | Risk |
| 15 | CYBRD1 | 0.31204 | Risk |
| 16 | RAMP1 | 0.110688 | Risk |
