# Supplementary material for: Glycosylation-related genes mediated prognostic signature contribute to prognostic prediction and treatment options in ovarian cancer: based on bulk and single‑cell RNA sequencing data
Source: BMC Cancer. 2024 Feb 14;24:207. doi: 10.1186/s12885-024-11908-4 (PMC10865697; doi:10.1186/s12885-024-11908-4)
Supplement: Supplementary file 12 — Supplementary Table 7. Relationship between CYBRD1 expression and tumor characteristics in patients with ovarian cancer. Note?*p<0.05?**p<0.05?***p<0.001 [file 12885_2024_11908_MOESM12_ESM.docx]

### Supplementary Table 7. Relationship between *CYBRD1* expression and tumor characteristics in patients with ovarian cancer. Note：*p<0.05；**p<0.05；***p<0.001

|  |  | *CYBRD1* expression | | *p* value |
| --- | --- | --- | --- | --- |
|  |  | 低 | 高 |  |
| Number | 150 | 66 | 84 |  |
| Age (year) |  |  |  | 0.08 |
| ≤51 | 76 | 40 | 36 |  |
| ＞51 | 74 | 28 | 46 |  |
| Grade |  |  |  | 0.32 |
| I | 13 | 7 | 6 |  |
| II | 15 | 7 | 8 |  |
| III | 92 | 32 | 60 |  |
| T |  |  |  | 0.22 |
| T1 | 12 | 6 | 6 |  |
| T2 | 35 | 18 | 17 |  |
| T3 | 103 | 41 | 62 |  |
| Lymph node metastasis |  |  |  | 0.005** |
| N0 | 112 | 56 | 56 |  |
| N1 | 38 | 8 | 30 |  |
| Distant metastasis |  |  |  | 0.004** |
| M0 | 116 | 57 | 59 |  |
| M1 | 34 | 7 | 27 |  |
| FIGO |  |  |  | 0.02* |
| 1 | 8 | 4 | 4 |  |
| 2 | 35 | 18 | 17 |  |
| 3 | 75 | 35 | 40 |  |
| 4 | 32 | 6 | 26 |  |
| Tumor size |  |  |  | 0.06 |
| ≤12.4cm | 74 | 40 | 34 |  |
| ＞12.4cm | 76 | 30 | 46 |  |
| Tumor recurrence |  |  |  | 0.30 |
| no | 33 | 17 | 15 |  |
| yes | 117 | 50 | 67 |  |
